# Prognostic Value of High-Density Lipoprotein Cholesterol in Patients with Overt Hepatic Encephalopathy

**DOI:** 10.3390/biomedicines12081783

**Published:** 2024-08-06

**Authors:** Ke Shi, Yufei Bi, Xiaojing Wang, Yanqiu Li, Xuanwei Zeng, Ying Feng, Xianbo Wang

**Affiliations:** Center of Integrative Medicine, Beijing Ditan Hospital, Capital Medical University, Beijing 100015, China; 13253691368@163.com (K.S.); yufei.bi@outlook.com (Y.B.); wangxiaojingdt@163.com (X.W.); yanqiuli0317@163.com (Y.L.); zengxuanweizxw@163.com (X.Z.)

**Keywords:** high-density lipoprotein cholesterol, hepatic encephalopathy, transplant-free mortality, prognosis

## Abstract

Overt hepatic encephalopathy (OHE), a serious complication of liver cirrhosis, is associated with alterations in lipid and lipoprotein metabolism. We evaluated the correlation between high-density lipoprotein cholesterol (HDL-C) levels and transplant-free (TF) mortality in patients with OHE. Patients with OHE admitted to Beijing Ditan Hospital between January 2010 and August 2016 (n = 821) and between September 2016 and December 2020 (n = 480) were included in the training and validation sets, respectively. Independent predictors were explored by a multivariate Cox regression analysis, and the area under the receiver operating characteristic curve (AUC) was used to assess the prognostic value of these factors. The prognostic value of HDL-C was good (AUC at 1 year: 0.745) and was equivalent to that of the Model for End-Stage Liver Disease (MELD) score (AUC at 1 year: 0.788). The optimal threshold values for HDL-C and MELD were 0.5 mmol/L and 17, respectively. The 1-year TF mortality rates in the low-risk (HDL-C ≥ 0.5 mmol/L and MELD < 17) and high-risk (HDL-C < 0.5 mmol/L and MELD ≥ 17) groups were 7.5% and 51.5% in the training set and 10.1% and 48.2% in the validation set, respectively. HDL-C level < 0.5 mmol/L and MELD score > 17 can facilitate the identification of high-risk patients and provide a basis for timely treatment.

## 1. Introduction

Hepatic encephalopathy (HE) is a clinical syndrome characterized by metabolic disturbances and central nervous system dysfunction due to chronic liver failure and portosystemic shunts [1]. Severe cases are classified as overt hepatic encephalopathy (OHE) and can involve sleep disorders, personality changes, cognitive impairment, asterixis, and coma [2]. OHE occurs in 30–45% of patients with cirrhosis, and the mortality rate is extremely high, with 1- and 5-year survival rates of <50% and <25%, respectively [3]. The high rates of hospitalization and 30 d mortality due to extrahepatic failure can mostly be attributed to OHE [4]. Therefore, convenient and low-cost prognostic markers for OHE are urgently needed.

The liver is the main organ of lipid biosynthesis and metabolism and plays a central role in lipoprotein synthesis [5]. In the lipid profile, high-density lipoprotein cholesterol (HDL-C) has antiapoptotic, antioxidation, and anti-inflammatory functions [6]. Reduced HDL-C levels may contribute to systemic inflammation, and reduced levels of circulating lipoproteins reflect the severity of liver dysfunction [7]. Dysfunctional HDL may contribute to the development of inflammatory diseases, such as cardiovascular diseases, diabetes, and kidney diseases [8,9,10]. Patients with advanced cirrhosis have disorders of lipid metabolism, such as abnormalities in HDL-C levels [11].

Increasing evidence suggests that HDL-C is an independent risk factor for the prognosis of various liver diseases [12,13,14]. The HDL-C level has emerged as a novel prognostic biomarker for the development of hepatocellular carcinoma [12]. Additionally, HDL-C is related to increased mortality in patients with alcoholic hepatitis and noncholestatic cirrhosis [13,14]. Recent prognostic models for cirrhosis have suggested that HDL-C level <0.4 mmol/L is the most appropriate cutoff value for identifying patients with a high risk of mortality [15]. In patients with acute decompensated cirrhosis, HDL-C levels serve as a significant predictor of 90 d mortality [7]. A cohort study of 153 patients with hepatitis B virus-related decompensated cirrhosis has shown that an HDL-C level of 0.53 mmol/L is a risk factor for 30 d mortality [16]. Nonetheless, few studies have confirmed the long-term prognostic value of HDL-C for transplant-free (TF) mortality in patients with OHE. Furthermore, owing to demographic differences among studies, the critical threshold value is uncertain. Accordingly, optimal methods for identifying patients at high risk of mortality according to HDL-C levels have not been determined.

The Cox proportional hazards model is widely used in biomedical research for evaluating relationships between predictor variables and time-to-event data [17]. Receiver operating characteristic (ROC) curves indicate the overall discriminatory ability of tests and enable quantitative comparisons of multiple diagnostic tests [18]. In the current study, the relationship between 1-year TF mortality and HDL-C levels in patients with OHE was evaluated using a Cox regression analysis and ROC curve. Our aim was to provide clinicians with an insight in order to help improve the prognosis of patients.

## 2. Materials and Methods

### 2.1. Study Population

**Figure 1:**
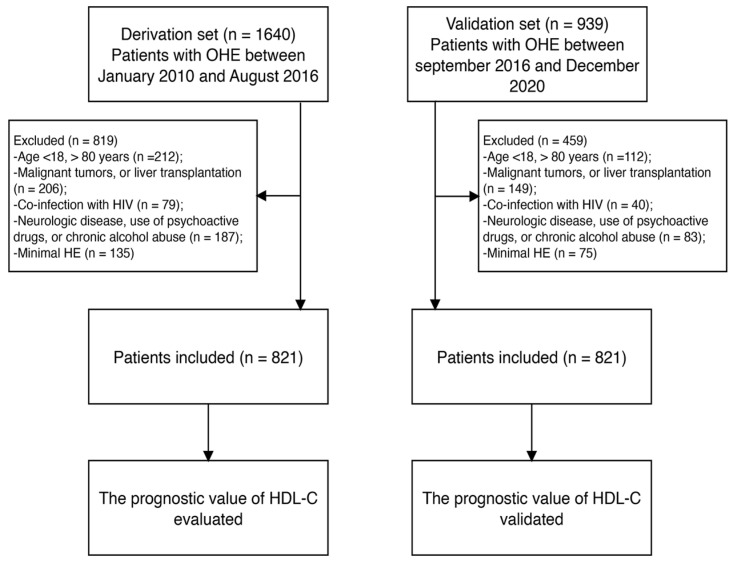
Study flow diagram. OHE, overt hepatic encephalopathy; HIV, human immunodeficiency virus; HDL-C, high-density lipoprotein cholesterol.

Patients with OHE (n = 1640) receiving medical care at Beijing Ditan Hospital from January 2010 to August 2016 were included in our analysis. The exclusion criteria were as follows: (i) age < 18 or >80 years; (ii) malignant tumors or liver transplantation; (iii) coinfection with human immunodeficiency virus; (iv) severe mental illness or use of medication for mental illness; (v) neurological diseases, use of psychoactive drugs, or chronic alcohol abuse; and (vi) minimal HE. Ultimately, 821 patients with OHE were included in the training set. Additionally, 480 patients recruited from September 2016 to December 2020 using the same criteria were included in the validation set (Figure 1).

### 2.2. Ethics and Consent

This study was approved by Beijing Ditan Hospital’s ethical committee (2021No. [002]-01) and adhered to the Declaration of Helsinki’s ethical principles. Written informed consent was obtained from each patient for the use of their data in this study.

### 2.3. Data Collection and Clinical Definitions

Baseline demographic and laboratory data were obtained from medical records, including age, sex, complications, and alanine aminotransferase (ALT), aspartate aminotransferase (AST), serum albumin (ALB), total bilirubin (TBIL), triglyceride (TG), total cholesterol (TC), serum creatinine (Cr), HDL-C, and low-density lipoprotein cholesterol (LDL-C) levels. In addition, the neutrophil–lymphocyte ratio (NLR), prothrombin time (PT), platelet count (PLT), prothrombin activity (PTA), and international normalized ratio (INR) were collected. Upon patient admission, all laboratory tests were completed within 48 h. These tests were performed by specialized technicians in our hospital’s laboratory department, using biochemical analyzers, hematology analyzers, hemagglutination analyzers, and other relevant equipment available. The occurrence of TF mortality within 1 year or at the end of the 1-year follow-up was defined as the endpoint. To determine the severity of liver disease, the Model for End-Stage Liver Disease (MELD) score was used [19]. HE was graded according to West–Haven criteria [2].

### 2.4. Statistical Analysis

IBM SPSS Statistics (IBM Corp., Armonk, NY, USA) and R version 4.2.3 (The R Foundation, Nashville, TN, USA) were used for all statistical analyses. Continuous variables are presented as the mean ± standard deviation, discrete variables are described as median values (interquartile range), and categorical variables are presented as frequencies or percentages. A Cox proportional risk regression analysis was performed to identify independent prognostic factors. Variables that were significant in univariate analyses were incorporated into a multivariate Cox proportional hazards model (backward stepwise selection, maximum likelihood ratio test). In addition, the area under the ROC curve (AUC) was used to evaluate the predictive value of independent risk indicators, and statistical significance was determined using the DeLong test [20]. Restricted cubic spline (RCS) was used to visualize the possible nonlinear dependency of the association between HDL-C and 1-, 3-, and 12-month TF mortality [21]. Kaplan–Meier curves and log-rank tests were used to evaluate survival. A forest plot was performed to determine the relationship between HDL-C and outcomes in different subgroups. Values with *p* < 0.05 were considered significant in all analyses.

## 3. Results

### 3.1. Baseline Characteristics of Patients with OHE

The training and validation groups included 821 and 480 patients, respectively. Table 1 presents characteristics of the two cohorts of patients with OHE. All patients had a median age of 55 years (range, 47–63 years), with 962 (73.9%) males and 339 (26.1%) females. In the training set, 679 patients (82.7%) were diagnosed with OHE grade II and 142 (17.3%) with grades III–IV. During the 1-year follow-up period, 200 (24.4%) deaths occurred in the training group and 126 (26.3%) in the validation group. No significant differences were found in baseline characteristics between the two subgroups.

We further compared the characteristics of patients who survived and died in the training cohort (Table 2). Patients who died were older and exhibited higher levels of ALT, TBIL, AST, and Cr; higher MELD scores, PTA, and INR (all *p* < 0.001); and higher frequencies of upper gastrointestinal bleeding (GIB), spontaneous bacterial peritonitis (SBP), and ascites than those of patients who survived. Notably, the patients who died had lower levels of LDL-C, TC, HDL-C, and ALB than those of patients who survived (all *p* < 0.001).

### 3.2. Identification of Independent Risk Factors for OHE

In the training cohort, univariate analyses revealed that age, sex, GIB, SBP, OHE grade, ascites, MELD score, and levels of AST, ALT, TBIL, ALB, TC, HDL-C, LDL-C, Cr, NLR, PT, and PTA were prognostic factors for 1-year TF mortality (all *p* < 0.05). These factors were included in a multivariate Cox regression analysis. Finally, age (adjusted hazard ratio [aHR], 1.030; 95% confidence interval [CI]: 1.016–1.044, *p* < 0.001), ascites (aHR, 1.974; 95% CI: 1.452–2.683, *p* < 0.001), MELD score (aHR, 1.107; 95% CI: 1.066–1.150, *p* < 0.001), HDL-C (aHR, 0.393; 95% CI: 0.218–0.711, *p* = 0.002), and NLR (aHR, 1.003; 95% CI: 1.014–1.053, *p* = 0.001) were identified as independent predictive biomarkers for patients with OHE (Table 3).

### 3.3. Prognosis Value of HDL-C in Patients with OHE

**Figure 2:**
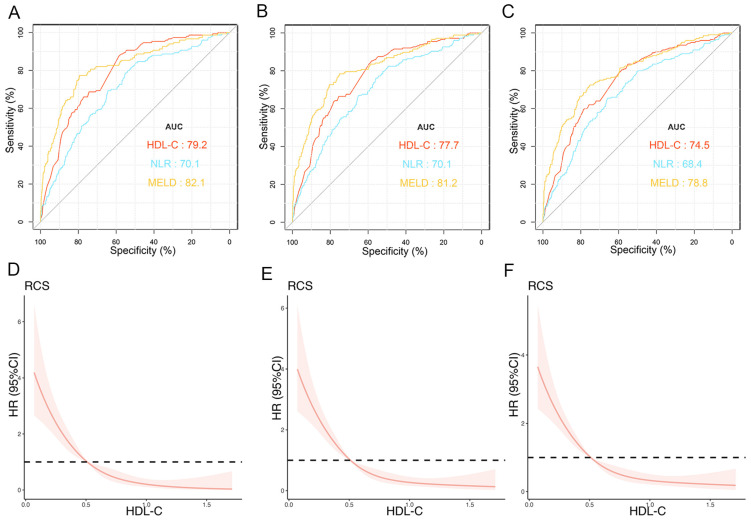
Predictive values of independent risk factors and relationship between HDL-C and prognosis in training cohort. AUCs of HDL-C, NLR, and MELD score predicting 1- (**A**), 3- (**B**), and 12-month (**C**) transplant-free (TF) mortality. Relationships between HDL-C and 1- (**D**), 3- (**E**), and 12-month (**F**) TF mortality in patients (unadjusted). Red lines represent references for HR, and red areas represent 95% CI. AUC, area under receiver operating characteristic curve; HDL-C, high-density lipoprotein cholesterol; MELD, Model for End-Stage Liver Disease; TF, transplant-free; NLR, neutrophil–lymphocyte ratio; HR, hazard ratio; CI, confidence interval.

The prognostic values of HDL-C at 1, 3, and 12 months were evaluated by ROC curves (Figure 2A–C). In the training cohort, the AUCs of HDL-C for TF mortality were 0.792 (95% CI: 0.755–0.829), 0.777 (95% CI: 0.735–0.810), and 0.745 (95% CI: 0.707–0.784) at 1, 3, and 12 months, respectively. The MELD score showed similar predictive abilities (AUCs at 1, 3, and 12 months: 0.821, 0.812, and 0.788, respectively). Furthermore, the performance of HDL-C was significantly higher than that of NLR at 1, 3, and 12 months (all *p* < 0.05). The RCS demonstrated that HDL-C levels and TF mortality displayed an L-shaped relationship, and an association was consistently observed at 1, 3, and 12 months (*p* for nonlinearity < 0.001). At lower HDL-C levels, the risk of mortality was elevated, whereas at higher levels, the mortality risk was reduced (Figure 2D–F). These findings suggest that HDL-C is an important factor influencing mortality outcomes.

### 3.4. Optimal Threshold Values for HDL-C and the MELD Score

**Figure 3:**
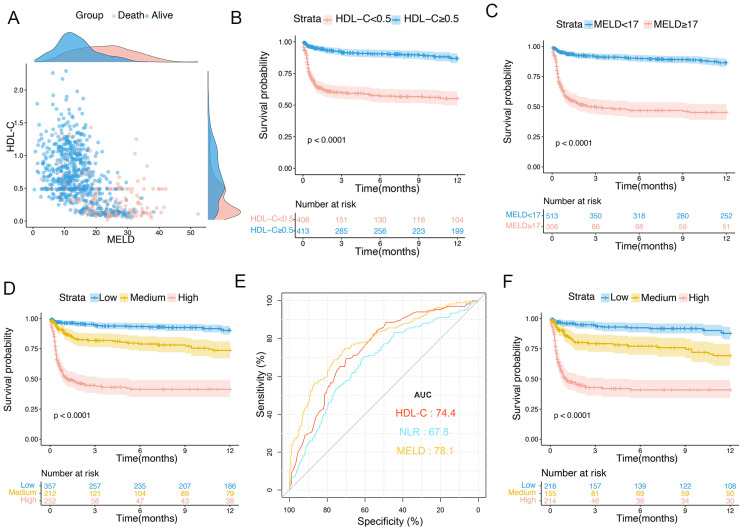
Scatter plot and risk stratification in patients with OHE in training set. (**A**) Distributions of patients with OHE who died and survived. (**B**) One-year survival probability in patients with HDL-C level < 0.5 mmol/L and ≥0.5 mmol/L. (**C**) One-year survival probability in patients with MELD score < 17 and ≥17. (**D**) Survival probability of patients in low-, medium-, and high-risk groups. (**E**) Predictive ability of different indicators for 1-year mortality in patients with ascites. (**F**) Survival probability of patients with ascites in low-, medium-, and high-risk groups. HDL-C, high-density lipoprotein cholesterol; MELD, Model for End-Stage Liver Disease; OHE, overt hepatic encephalopathy.

The optimal threshold values for HDL-C and the MELD score were identified as 0.5 mmol/L and 17, respectively. Scatter plots were produced to visualize the associations between HDL-C, MELD score, and 12-month TF mortality. As shown in Figure 3A, in the training set, patients with HDL-C level < 0.5 mmol/L and MELD score ≥ 17 had a worse prognosis than those of other groups. We further compared the characteristics of patients with HDL-C levels of <0.5 mmol/L and ≥0.5 mmol/L (Table 4). Patients with HDL-C < 0.5 mmol/L had higher MELD scores, rates of GIB, SBP, and ascites; higher levels of ALT, AST, TBIL, and Cr; and higher PLT, NLR, PT, and INR than those of patients with HDL-C level ≥ 0.5 mmol/L (all *p* < 0.001).

### 3.5. Risk Prediction Stratification for Patients with OHE

According to the optimal cutoff values for HDL-C and the MELD score, Kaplan–Meier curves were plotted to show the 1-year TF mortality in the training set. Patients with HDL-C < 0.5 mmol/L had a significantly higher 1-year TF mortality than those with HDL-C ≥ 0.5 mmol/L (38.7% vs. 10.3%, *p* < 0.0001; Figure 3B). Furthermore, the 1-year TF mortalities were 47% in the MELD score ≥ 17 group and 10.7% in the MELD score < 17 group (*p* < 0.0001; Figure 3C). Subsequently, the patients were divided into three groups: low- (HDL-C ≥ 0.5 mmol/L and MELD < 17), medium- (HDL-C < 0.5 mmol/L or MELD ≥ 17), and high-risk (HDL-C < 0.5 mmol/L and MELD ≥ 17). The 1-year TF mortalities were 7.5%, 20.2%, and 51.5% in the low-, medium-, and high-risk subgroups, respectively (*p* < 0.0001; Figure 3D).

In this study, ascites was an independent adverse prognostic factor for patients with OHE. We further evaluated the prognostic value of HDL-C levels in patients with ascites and evaluated risk stratification in these patients. The AUCs for the HDL-C levels, MELD scores, and NLR curves were 0.744 (95% CI: 0.701–0.787), 0.781 (95% CI: 0.738–0.824), and 0.678 (95% CI: 0.629–0.726), respectively (Figure 3E). The 1-year TF mortality rates for patients in the low-, medium-, and high-risk subgroups were 9.1%, 22.5%, and 51.8%, respectively (*p* < 0.0001; Figure 3F).

### 3.6. Subgroup Analysis

**Figure 4:**
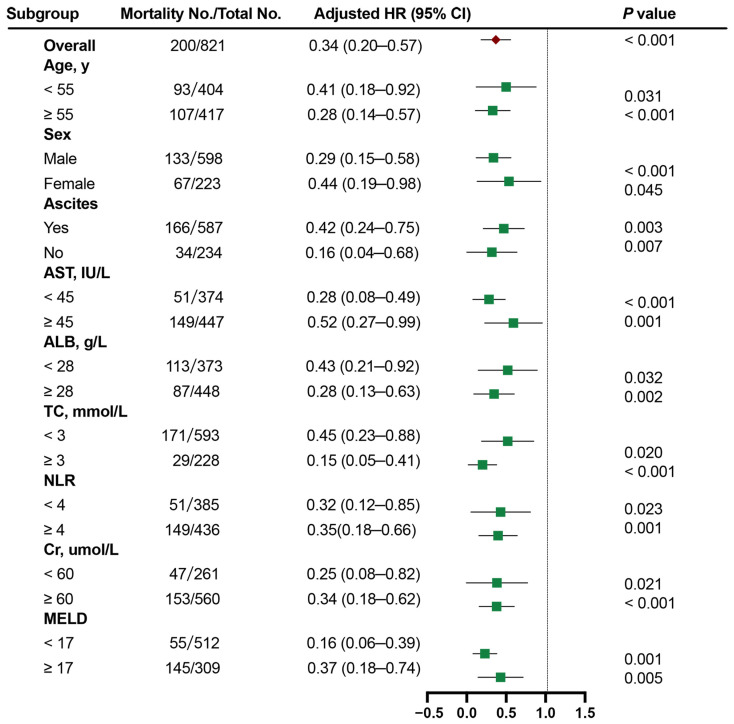
The Cox proportional hazards analysis of the prognostic value of HDL-C levels in various subgroups. HR was adjusted for age, sex, ascites, NLR, and the MELD score in the multivariate model. HDL-C, high-density lipoprotein cholesterol; MELD, Model for End-Stage Liver Disease; HR, hazard ratio.

To elucidate the relationship between HDL-C and 1-year TF mortality across patient subgroups, we conducted a comprehensive multivariate stratified analysis, accounting for a wide range of factors, including demographic factors (age and sex), complications (ascites), liver function parameters (AST and ALB), inflammatory markers (NLR), lipid indicators (TC), renal function (Cr), and the MELD score. Continuous variables were classified according to cutoff values. In stratified analyses, patients with low HDL-C had worse outcomes across various subgroups (all aHR > 1.0; Figure 4).

### 3.7. Validation of the Prognostic Value of the HDL-C Level and MELD Score

**Figure 5:**
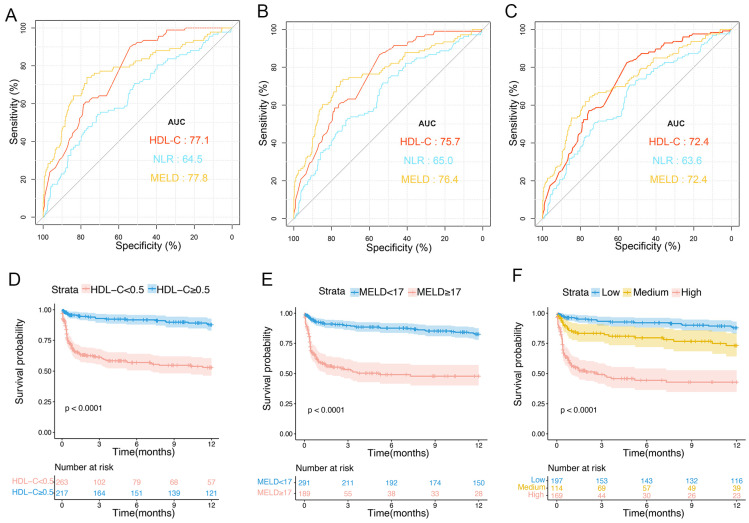
Predictive values of different risk factors and risk stratification in the validation cohort. ROC curves of HDL-C levels, NLR, and MELD scores in predicting 1- (**A**), 3- (**B**), and 12-month TF mortality (**C**). (**D**) Survival probability in patients with HDL-C values of <0.5 mmol/L and ≥0.5 mmol/L. (**E**) Survival probabilities for patients with MELD scores of <17 and ≥17. (**F**) Survival probabilities of patients in the low-, medium-, and high-risk groups. ROC, receiver operating characteristic; HDL-C, high-density lipoprotein cholesterol; NLR, neutrophil–lymphocyte ratio; MELD, Model for End-Stage Liver Disease; TF, transplant-free.

The AUCs of HDL-C in the validation set at 1, 3, and 12 months were 0.771 (95% CI: 0.724–0.818), 0.757 (95% CI: 0.710–0.806), and 0.724 (95% CI: 0.675–0.773), respectively. Additionally, the performances of HDL-C levels and MELD scores were similar (0.724; 95% CI: 0.671–0.787) but were higher than those of NLR (0.636; 95% CI: 0.579–0.693) at 1 year (Figure 5A–C). Patients with HDL-C levels < 0.5 mmol/L had a significantly higher 1-year TF mortality rate than that of patients with HDL-C levels ≥ 0.5 mmol/L (10% vs. 39.6%, *p* < 0.0001; Figure 5D). Patients with an MELD score ≥ 17 had a significantly higher 1-year TF mortality rate than that of patients with an MELD score <17 (44.2% vs. 14.3%, *p* < 0.0001; Figure 5E). The 1-year TF mortality rates for patients in the low- (HDL-C ≥ 0.5 mmol/L and MELD < 17), medium- (HDL-C < 0.5 mmol/L or MELD ≥17), and high-risk groups (HDL-C < 0.5 mmol/L and MELD ≥ 17) were 10.1%, 20.8%, and 48.2%, respectively (*p* < 0.001; Figure 5F).

## 4. Discussion

HE is a common complication and cause of death in patients with severe liver disease. The pathogenesis is unclear, and combined therapy is still the main treatment approach. The identification of simple, inexpensive, and rapid prognostic markers for OHE may reduce mortality and improve prognoses. Accordingly, associations between lipid-related markers and liver disease progression are of growing interest [22,23]. In the current study, we confirmed that baseline HDL-C levels are associated with short- and long-term prognoses in patients with OHE.

In this study, HDL-C levels were lower in patients who died than in those who survived. We found an L-shaped correlation between TF mortality and HDL-C levels in patients with OHE. HDL-C was identified as an independent prognostic factor, successfully predicting 1-, 3-, and 12-month TF mortality. The MELD score and NLR are recognized assessment tools to estimate the prognosis in liver disease [24,25]. We previously found that an elevated NLR is independently related to the prognosis of patients with OHE [26]. Furthermore, we constructed a dynamic nomogram model to predict TF mortality based on the MELD score and NLR [27]. The predictive performance of HDL-C levels was equal to that of the MELD score and higher than that of NLR in this study. The MELD score was initially devised to assess and predict the progression of cirrhosis by incorporating three key parameters: serum Cr levels, TBIL levels, and the INR. However, these parameters primarily reflect liver function, without considering disease pathogenesis or progression. In contrast, HDL-C levels represent a diverse array of pathophysiological processes and have the potential to improve the predictive performance of traditional scoring systems [7,28]. Consequently, the HDL-C level exhibits comparable prognostic value to that of the MELD score. Indeed, an increasing body of research has substantiated the superior prognostic ability of HDL-C for cirrhosis outcomes. For example, in a recent study [7], the HDL-C level was identified as an MELD-independent predictor of 90 d mortality in patients with acute decompensated cirrhosis. HDL-C and the MELD score had similar diagnostic accuracies for 90 d mortality. Another investigation demonstrated that incorporating HDL-C levels into the MELD score significantly improves the predictive accuracy for 30 d mortality over that using the MELD score alone [28]. Moreover, fluctuations in HDL-C levels have been identified as effective indicators of liver function and survival in cirrhosis. The underlying mechanism involves hepatocellular damage, which impairs the production and secretion of apolipoprotein A-I substantially, consequently leading to reduced HDL-C levels [29].

HDL is the main lipid component of serum and can bind cholesterol, phospholipids, and other lipid components [30]. Since the liver plays a crucial role in the synthesis, transport, and metabolism of lipids, patients with cirrhosis often exhibit abnormal lipid levels. Furthermore, lipid levels are correlated with the nutritional status, and hypoproteinemia and sarcopenia are common in patients with OHE [31]. Bories et al. found that nutritional supplementation in patients with cirrhosis improves indicators of liver function and increases HDL-C levels [32]. Furthermore, lipids play crucial roles in multiple processes linked to the pathogenesis of inflammatory diseases. Lipid-mediated signal transduction can regulate mechanisms closely associated with distal organ and cellular damage as well as inflammation and infection-related apoptosis and autophagy [33,34]. Numerous lipid molecules are involved in inflammatory processes [35]. The inflammatory reaction induced by infection is the main predisposing risk factor for OHE development. HDL-C plays an essential role in inhibiting endogenous inflammation, and reduced levels of HDL-C are related to systemic inflammatory responses [36]. HDL-C regulates cholesterol efflux from macrophage foam cells, thereby reducing lipid-induced proinflammatory cytokine secretion [37]. It potentially attenuates inflammatory signaling pathways and modulates cellular membrane cholesterol levels by facilitating the efflux of cholesterol and other lipids from cells [38]. Furthermore, HDL-C binds to and solubilizes lipopolysaccharides, reduces adhesion molecule expression, and inhibits macrophage-mediated cytokine secretion [39]. There is an inverse correlation between HDL-C and C-reactive protein levels, supporting the anti-inflammatory activity of HDL-C [40]. In patients with OHE, HDL-C may exhibit a loss of its characteristic anti-inflammatory properties. This dysfunction in HDL could potentially contribute to adverse patient outcomes.

To our knowledge, this study provides the first evidence for the relationship between HDL-C levels and prognoses of patients with OHE. In the training cohort, the 1-year TF mortality rate was significantly higher in patients at high risk (HDL-C < 0.5 mmol/L and MELD ≥ 17) than in those at low risk (HDL-C ≥ 0.5 mmol/L and MELD < 17) (51.5% vs. 7.5%, *p* < 0.0001). Based on the HDL-C levels and MELD scores, clinicians could identify patients at high risk, accurately predict outcomes, and ensure the timely implementation of therapeutic strategies. Nevertheless, a few limitations of this study should be acknowledged. First, this is a retrospective study conducted at a single institution. To address this limitation, we validated the value of HDL-C levels in an internal cohort. However, to definitively establish the prognostic significance of HDL-C in patients with OHE, conducting prospective, multicenter studies is imperative. Large-scale studies have the potential to generate high-quality evidence to substantiate the inclusion of the HDL-C level as a key indicator in future clinical management guidelines for OHE. This evidence-based approach would significantly enhance the standard of care and therapeutic decision-making. Second, we focused on clinical characteristics associated with HDL-C changes. Our research team is conducting ongoing investigations within our cohort to delineate the alterations in serum markers of lipid metabolism in patients with OHE. The aim of these efforts is to gain deeper insights into the pathophysiological mechanisms driving this complex disease process. Third, we only collected baseline data and did not analyze the dynamic changes in HDL-C levels and outcomes. Finally, although we adjusted for confounding factors, unmeasured factors may influence the results.

## 5. Conclusions

In summary, this study revealed that low HDL-C levels can be a robust indicator for predicting 1-year TF mortality in patients with OHE. Patients with HDL-C level < 0.5 mmol/L and MELD score ≥ 17 had a poor prognosis. Monitoring HDL-C levels may assist clinicians in identifying high-risk patients and administering early treatment to improve outcomes. To conclusively establish the prognostic value of HDL-C levels in patients with OHE, further prospective multicenter studies are essential. These future investigations will serve to validate and expand upon our current findings, ultimately providing a more comprehensive understanding of lipid-related indicators as potential therapeutic targets for patients with OHE.

## Figures and Tables

**Table 1:** Clinical baseline characteristics of patients with liver cirrhosis and overt hepatic encephalopathy in the training and validation sets.

Variables	Training Cohort (n = 821)	Validation Cohort (n = 480)	*p*-Value
Age (years)	54.0 (47.0,61.0)	55.0 (47.0,63.0)	0.896
Male	598 (72.8)	364 (75.8)	0.235
Diabetes (%)	198 (24.1)	126 (26.3)	0.391
GIB (%)	291 (35.4)	158 (32.9)	0.355
SBP (%)	50 (6.1)	27 (5.6)	0.732
Ascites (%)	587 (71.5)	348 (72.5)	0.698
Classification of OHE (%)			0.451
Grade II	679 (82.7)	389 (81.0)	
Grade III–IV	142 (17.3)	91 (19.0)	
ALT (IU/L)	31.0 (19.7,59.2)	30.7 (19.2,56.6)	0.742
AST (IU/L)	49.9 (32.7,103.2)	66.8 (34.9,169.8)	0.706
TBIL (µmol/L)	55.3 (27.2,140.2)	53.8 (32.2,102.9)	0.248
Albumin (g/L)	28.4 ± 4.9	28.0 ± 5.3	0.255
TC (mmol/L)	2.4 (1.8,3.1)	2.4 (2.0,2.9)	0.762
TG (mmol/L)	0.6 (0.4,0.8)	0.6 (0.4,0.8)	0.318
HDL-C (mmol/L)	0.5 (0.3,0.9)	0.5 (0.2,0.8)	0.123
LDL-C (mmol/L)	1.1 (0.8,1.6)	1.1 (0.8,1.5)	0.179
NLR	4.3 (2.1,7.3)	4.3 (2.3,7.9)	0.597
PLT (×10^9^/L)	66.9 (43.4,103.0)	69.2 (48.7,102.0)	0.432
PT (s)	17.6 (15.2,22.0)	17.7 (15.3,23.3)	0.322
PTA (%)	49.6 (36.3,62.3)	48.7 (34.0,62.0)	0.463
INR	1.5 (1.3,1.9)	1.5 (1.3,1.9)	0.160
Cr (µmol/L)	70.3 (56.7,96.3)	69.8 (56.0,97.6)	0.831
MELD score	14.5 (10.3,20.7)	14.4 (10.0,22.2)	0.455
Mortality (%)	200 (24.4)	126 (26.3)	0.448

Data are presented as n (%), mean ± SD, or median (interquartile range). GIB, upper gastrointestinal bleeding; SBP, spontaneous bacterial peritonitis; OHE, overt hepatic encephalopathy; ALT, alanine aminotransferase; AST, aspartate aminotransferase; TBIL, total bilirubin; TC, total cholesterol; TG, triglyceride; HDL-C, high-density lipoprotein cholesterol; LDL-C, low-density lipoprotein cholesterol; NLR, neutrophil–lymphocyte ratio; PLT, platelet; INR, international normalized ratio; PT, prothrombin time; PTA, prothrombin activity; Cr, serum creatinine; MELD, Model for End-Stage Liver Disease.

**Table 2:** Baseline characteristics of surviving and deceased patients in the training cohort.

Variables	Survived (n = 621)	Death (n = 200)	*p*-Value
Age (years)	54.0 (48.0,62.0)	56.5 (47.0,66.0)	0.001
Male	465 (74.9)	133 (66.5)	0.021
Diabetes (%)	148 (23.8)	50 (25.0)	0.737
GIB (%)	202 (32.5)	89 (44.5)	0.002
SBP (%)	26 (4.2)	24 (12.0)	0.001
Ascites (%)	421 (67.8)	166 (83.0)	<0.001
ALT (IU/L)	28.2 (18.5,47.3)	51.8 (25.5,142.0)	<0.001
AST (IU/L)	43.9 (30.2,76.5)	97.4 (44.6,241.8)	<0.001
TBIL (µmol/L)	44.8 (24.2,92.4)	174.1 (62.5,399.7)	<0.001
Albumin (g/L)	28.8 ± 4.8	26.9 ± 4.9	<0.001
TC (mmol/L)	2.5 (2.1,3.2)	1.9 (1.4,2.4)	<0.001
TG (mmol/L)	0.6 (0.4,0.8)	0.6 (0.4,0.7)	0.219
HDL-C (mmol/L)	0.6 (0.4,0.9)	0.3 (0.1,0.5)	<0.001
LDL-C (mmol/L)	1.2 (0.9,1.6)	0.8 (0.5,1.1)	<0.001
NLR	3.7 (2.2,6.2)	6.5 (3.9,10.6)	<0.001
PLT (×10^9^/L)	68.0 (44.2,103.0)	62.5 (40.5,105.7)	0.743
INR	1.5 (1.3,1.7)	1.9 (1.5,2.4)	0.219
Cr (µmol/L)	67.4 (55.7,87.0)	88.9 (61.0,146.6)	<0.001
MELD score	13.0 (9.4,17.1)	23.2 (15.9,29.1)	<0.001

Data are presented as n (%), mean ± SD, or median (interquartile range). GIB, upper gastrointestinal bleeding; SBP, spontaneous bacterial peritonitis; ALT, alanine aminotransferase; AST, aspartate aminotransferase; TBIL, total bilirubin; TC, total cholesterol; TG, triglyceride; HDL-C, high-density lipoprotein cholesterol; LDL-C, low-density lipoprotein cholesterol; PLT, platelet; NLR, neutrophil–lymphocyte ratio; INR, international normalized ratio; PT, prothrombin time; PTA, prothrombin activity; Cr, serum creatinine; MELD, Model for End-Stage Liver Disease.

**Table 3:** Univariate and multivariate Cox regression analyses for 1-year transplant-free mortality in patients with overt hepatic encephalopathy in the training set.

Variables	Univariate Analysis		Multivariate Analysis	
	HR (95% CI)	*p*-Value	HR (95% CI)	*p*-Value
Age (years)	1.012 (1.000,1.025)	0.048	1.030 (1.016,1.044)	<0.001
Male	0.708 (0.521,0.949)	0.021		
Diabetes	1.082 (0.786,1.490)	0.629		
GIB (Yes or no)	1.523 (1.152,2.012)	0.003		
SBP (Yes or no)	2.383 (1.552,3.657)	0.001		
Ascites (Yes or no)	2.172 (1.501,3.143)	<0.001	1.974 (1.452,2.683)	<0.001
Classification of OHE (%)				
Grade II	Reference			
Grade III–IV	1.447 (1.029,2.003)	0.033		
MELD score	1.124 (1.108,1.141)	<0.001	1.107 (1.066,1.150)	<0.001
ALT (IU/L)	1.001 (1.001,1.002)	<0.001		
AST (IU/L)	1.001 (1.001,1.001)	<0.001		
TBIL (µmol/L)	1.004 (1.003,1.005)	<0.001		
Albumin (g/L)	0.925 (0.899,0.952)	<0.001		
TC (mmol/L)	1.100 (1.006,1.135)	<0.001		
TG (mmol/L)	1.115 (0.960,1.391)	0.217		
HDL-C (mmol/L)	0.008 (0.049,0.113)	<0.001	0.393 (0.218,0.711)	0.002
LDL-C (mmol/L)	0.365 (0.274,0.486)	<0.001		
NLR	1.061 (1.046,1.076)	<0.001	1.003 (1.014,1.053)	0.001
PLT (×10^9^/L)	1.000 (0.997,1.002)	0.774		
PT (s)	1.077 (1.064,1.089)	<0.001		
PTA (%)	0.951 (0.943,0.959)	<0.001		
Cr (µmol/L)	1.004 (1.003,1.005)	<0.001		

CI, confidence interval; HR, hazard ratio; GIB, upper gastrointestinal bleeding; SBP, spontaneous bacterial peritonitis; OHE, overt hepatic encephalopathy; ALT, alanine aminotransferase; AST, aspartate aminotransferase; TBIL, total bilirubin; TC, total cholesterol; TG, triglyceride; HDL-C, high-density lipoprotein cholesterol; LDL-C, low-density lipoprotein cholesterol; NLR, neutrophil–lymphocyte ratio; PLT, platelet; PT, prothrombin time; PTA, prothrombin activity; Cr, serum creatinine.

**Table 4:** Clinical characteristics according to HDL-C levels in the training set.

Variables	HDL-C < 0.5 mmol/L (n = 408)	HDL-C ≥ 0.5 mmol/L (n = 413)	*p*-Value
Age (years)	53.0 (46.0,63.0)	56.0 (49.0,63.0)	0.008
Male	302 (74.0)	296 (71.7)	0.449
Diabetes (%)	89 (21.8)	109 (26.4)	0.125
GIB (%)	149 (36.5)	142 (34.4)	0.552
SBP (%)	39 (9.6)	11 (2.7)	<0.001
Ascites (%)	327 (80.1)	260 (63.0)	<0.001
ALT (IU/L)	42.3 (23.0,130.4)	25.8 (17.9,39.5)	<0.001
AST (IU/L)	81.2 (42.7,190.0)	38.5 (27.8,57.2)	<0.001
TBIL (µmol/L)	124.1 (51.5,286.6)	34.2 (20.3,59.7)	<0.001
Albumin (g/L)	27.1 ± 4.8	29.6 ± 4.7	<0.001
TC (mmol/L)	2.1 (1.5,2.4)	2.8 (2.3,3.5)	<0.001
TG (mmol/L)	0.6 (0.5,0.8)	0.6 (0.4,0.8)	0.129
LDL-C (mmol/L)	1.0 (0.6,1.1)	1.4 (1.0,1.8)	<0.001
NLR	5.1 (3.0,8.6)	3.5 (2.1,5.9)	<0.001
PLT (×10^9^/L)	70.5 (42.2,108.0)	65.2 (44.0,95.7)	0.407
INR	1.8 (1.4,2.2)	1.3 (1.2,1.5)	<0.001
Cr (µmol/L)	76.1 (59.6,113.2)	66.7 (54.0,84.9)	<0.001
MELD score	19.7 (13.5,26.1)	11.7 (9.0,14.9)	<0.001

Data are presented as n (%), mean ± SD, or median (interquartile range). GIB, upper gastrointestinal bleeding; SBP, spontaneous bacterial peritonitis; ALT, alanine aminotransferase; AST, aspartate aminotransferase; TBIL, total bilirubin; TC, total cholesterol; TG, triglyceride; LDL-C, low-density lipoprotein cholesterol; NLR, neutrophil–lymphocyte ratio; PT, prothrombin time; PTA, prothrombin activity; PLT, platelet; INR, international normalized ratio; Cr, serum creatinine; MELD, Model for End-Stage Liver Disease.

## Data Availability

The original contributions presented in the study are included in the article/Supplementary Material. Further inquiries can be directed to the corresponding author.

## Supplementary Materials

The following supporting information can be downloaded at: https://www.mdpi.com/article/10.3390/biomedicines12081783/s1.
