# Supplementary material for: Prognostic Value of High-Density Lipoprotein Cholesterol in Patients with Overt Hepatic Encephalopathy
Source: Biomedicines. 2024 Aug 6;12(8):1783. doi: 10.3390/biomedicines12081783 (PMC11351328; doi:10.3390/biomedicines12081783)
Supplement: Supplementary file 1 [file biomedicines-12-01783-s001.zip › biomedicines-3107357-supplementary.pdf]

ID	sex	age	30d	30out	90d	90out	360d	360out	cirrhosis
110808	1	58	1	1	1	1	1	1	1
114014	0	48	1	1	1	1	1	1	1
115639	1	56	1	1	1	1	1	1	1
121140	1	54	1	1	1	1	1	1	1
108966	0	71	1	1	1	1	1	1	1
127875	0	62	1	1	1	1	1	1	1
128718	0	66	1	1	1	1	1	1	1
133766	1	60	1	1	1	1	1	1	1
135224	1	50	1	1	1	1	1	1	1
143822	0	58	1	1	1	1	1	1	1
144084	1	64	1	1	1	1	1	1	1
154372	1	46	1	1	1	1	1	1	1
164091	1	54	1	1	1	1	1	1	1
179990	1	50	1	1	1	1	1	1	1
181647	1	52	1	1	1	1	1	1	1
187542	1	40	1	1	1	1	1	1	1
178636	1	51	1	1	1	1	1	1	1
198991	1	55	1	1	1	1	1	1	1
215802	1	58	1	1	1	1	1	1	1
207225	1	61	1	1	1	1	1	1	1
107744	0	77	2	1	2	1	2	1	1
109684	1	40	2	1	2	1	2	1	1
112169	0	66	2	1	2	1	2	1	1
122144	0	75	2	1	2	1	2	1	1
104828	0	65	2	1	2	1	2	1	1
135402	1	57	2	1	2	1	2	1	1
127563	1	73	2	1	2	1	2	1	1
139189	1	49	2	1	2	1	2	1	1
126145	0	66	2	1	2	1	2	1	1
147754	0	66	2	1	2	1	2	1	1
148176	0	64	2	1	2	1	2	1	1
169325	1	51	2	1	2	1	2	1	1
182357	1	44	2	1	2	1	2	1	1
190193	1	59	2	1	2	1	2	1	1
196825	1	57	2	1	2	1	2	1	1
212405	1	66	2	1	2	1	2	1	1
101952	1	51	5	1	5	1	5	1	1
106418	1	38	12	0	12	0	12	0	1
107148	1	57	6	0	6	0	6	0	1
90879	0	54	16	0	16	0	16	0	1
115036	1	26	25	0	25	0	25	0	1
89288	0	55	22	1	22	1	22	1	1
103163	1	70	13	0	13	0	13	0	1
103187	1	65	24	0	24	0	24	0	1

119723	1	32	16	1	16	1	16	1	1
103056	1	61	7	0	7	0	7	0	1
103115	1	72	9	1	9	1	9	1	1
103929	0	78	3	0	3	0	3	0	1
104203	0	56	18	0	18	0	18	0	1
104239	1	59	3	0	3	0	3	0	1
124272	1	37	9	1	9	1	9	1	1
87914	0	70	21	0	21	0	21	0	1
67082	1	47	6	1	6	1	6	1	1
128965	0	51	22	0	22	0	22	0	1
133784	1	54	10	1	10	1	10	1	1
108843	1	55	26	1	26	1	26	1	1
108580	0	60	23	0	23	0	23	0	1
94341	1	52	30	0	90	0	170	0	1
83925	1	47	8	1	8	1	8	1	1
110692	0	55	12	1	12	1	12	1	1
111331	1	45	25	0	25	0	25	0	1
111832	0	64	9	1	9	1	9	1	1
105510	1	39	19	1	19	1	19	1	1
112170	1	41	6	0	6	0	6	0	1
112310	1	61	9	1	9	1	9	1	1
112599	1	60	10	1	10	1	10	1	1
112691	1	63	20	0	20	0	20	0	1
112809	0	59	15	0	15	0	15	0	1
112988	1	58	8	1	8	1	8	1	1
114262	0	73	5	0	5	0	5	0	1
114723	1	43	8	1	8	1	8	1	1
114801	1	47	4	0	4	0	4	0	1
115190	0	68	17	0	17	0	17	0	1
116149	0	76	21	0	21	0	21	0	1
116210	1	36	7	1	7	1	7	1	1
116259	1	57	13	1	13	1	13	1	1
116880	1	32	17	1	17	1	17	1	1
124700	1	52	3	1	3	1	3	1	1
118611	1	28	20	0	20	0	20	0	1
119073	1	57	5	0	5	0	5	0	1
119341	0	60	12	1	12	1	12	1	1
208608	1	47	17	1	17	1	17	1	1
119620	0	70	24	0	24	0	24	0	1
120830	1	47	21	0	21	0	21	0	1
110097	1	44	20	1	20	1	20	1	1
121632	1	64	18	0	18	0	18	0	1
121974	1	65	20	0	20	0	20	0	1
122742	1	63	23	1	23	1	23	1	1
123097	1	59	30	0	90	0	360	0	1

292548	0	76	9	0	9	0	9	0	1
121360	0	91	3	0	3	0	3	0	1
123983	0	58	13	1	13	1	13	1	1
103574	0	58	24	0	24	0	24	0	1
124303	0	77	10	0	10	0	10	0	1
124305	0	74	17	0	17	0	17	0	1
124340	1	47	10	0	10	0	10	0	1
124445	1	54	5	0	5	0	5	0	1
218538	1	47	10	1	10	1	10	1	1
125172	1	23	3	0	3	0	3	0	1
125197	1	58	30	0	90	0	360	0	1
125534	1	34	13	1	13	1	13	1	1
125701	0	78	16	0	16	0	16	0	1
112384	1	64	5	0	5	0	5	0	1
125856	1	70	30	0	34	0	34	0	1
125911	1	44	3	0	3	0	3	0	1
126123	1	52	9	1	9	1	9	1	1
122995	1	39	30	0	90	0	360	0	1
126837	1	57	10	1	10	1	10	1	1
127273	0	59	8	0	8	0	8	0	1
127476	1	41	14	0	14	0	14	0	1
127614	1	53	11	1	11	1	11	1	1
129274	1	47	23	1	23	1	23	1	1
130056	1	64	15	0	15	0	15	0	1
130272	0	53	30	1	30	1	30	1	1
130437	1	43	3	0	3	0	3	0	1
129221	1	56	3	0	3	0	3	0	1
131062	1	34	11	1	11	1	11	1	1
131128	0	63	11	1	11	1	11	1	1
119972	1	56	2	0	2	0	2	0	1
131142	1	71	8	0	8	0	8	0	1
131462	1	39	3	0	3	0	3	0	1
123986	1	64	9	1	9	1	9	1	1
126802	1	43	25	0	25	0	25	0	1
133267	0	58	16	0	16	0	16	0	1
122657	0	54	18	0	18	0	18	0	1
133528	0	61	12	1	12	1	12	1	1
133859	1	55	5	0	5	0	5	0	1
134170	1	68	7	0	7	0	7	0	1
116823	1	37	9	0	9	0	9	0	1
135349	1	48	10	0	10	0	10	0	1
135948	1	48	13	0	13	0	13	0	1
136327	1	71	12	1	12	1	12	1	1
136431	1	74	17	0	17	0	17	0	1
136713	1	48	12	1	12	1	12	1	1

137008	0	49	9	1	9	1	9	1	1
137195	0	65	29	1	29	1	29	1	1
137508	0	44	6	0	6	0	6	0	1
134959	1	66	9	0	9	0	9	0	1
139443	1	58	6	0	6	0	6	0	1
113421	1	42	6	0	6	0	6	0	1
140364	1	52	16	1	16	1	16	1	1
140755	0	82	15	0	15	0	15	0	1
140825	1	74	16	1	16	1	16	1	1
140912	1	41	11	1	11	1	11	1	1
141095	1	26	26	1	26	1	26	1	1
141757	0	61	7	0	7	0	7	0	1
141150	0	63	26	0	26	0	26	0	1
142371	1	76	24	0	24	0	24	0	1
102064	0	73	23	0	23	0	23	0	1
142997	1	60	7	0	7	0	7	0	1
143630	1	47	5	0	5	0	5	0	1
143666	1	59	9	1	9	1	9	1	1
144562	0	72	14	1	14	1	14	1	1
145352	0	71	6	0	6	0	6	0	1
143440	1	78	12	1	12	1	12	1	1
145957	1	50	10	1	10	1	10	1	1
145991	1	61	9	1	9	1	9	1	1
130600	1	57	12	1	12	1	12	1	1
146162	1	41	9	0	9	0	9	0	1
146296	1	55	6	0	6	0	6	0	1
146779	1	73	3	0	3	0	3	0	1
147547	1	42	6	0	6	0	6	0	1
147677	1	59	19	0	19	0	19	0	1
147900	1	44	30	0	90	0	360	0	1
148079	0	50	18	1	18	1	18	1	1
148190	1	55	19	1	19	1	19	1	1
130710	0	73	8	0	8	0	8	0	1
145683	1	40	14	0	14	0	14	0	1
148976	1	48	21	0	21	0	21	0	1
149241	1	40	6	0	6	0	6	0	1
149421	1	55	9	0	9	0	9	0	1
149729	1	54	6	0	6	0	6	0	1
149761	1	52	22	0	22	0	22	0	1
150900	1	70	13	1	13	1	13	1	1
151181	1	48	8	1	8	1	8	1	1
151186	1	53	11	1	11	1	11	1	1
110339	1	63	15	1	15	1	15	1	1
127703	1	44	12	1	12	1	12	1	1
147662	1	58	16	0	16	0	16	0	1

112316	0	45	9	1	9	1	9	1	1
152919	1	44	16	0	16	0	16	0	1
139855	1	39	7	1	7	1	7	1	1
153723	1	74	13	1	13	1	13	1	1
154138	1	50	6	0	6	0	6	0	1
154356	0	83	13	0	13	0	13	0	1
154663	1	39	12	1	12	1	12	1	1
155259	1	61	23	0	23	0	23	0	1
145812	1	35	22	0	22	0	22	0	1
156619	1	66	13	1	13	1	13	1	1
156822	0	52	11	1	11	1	11	1	1
101119	0	65	4	0	4	0	4	0	1
156942	0	64	5	0	5	0	5	0	1
157018	1	48	8	0	8	0	8	0	1
157122	0	73	16	0	16	0	16	0	1
157294	1	50	7	0	7	0	7	0	1
155007	1	71	22	0	22	0	22	0	1
143815	1	54	4	0	4	0	4	0	1
157985	1	56	4	0	4	0	4	0	1
158829	1	50	18	0	18	0	18	0	1
147442	1	59	6	0	6	0	6	0	1
159897	1	64	13	0	13	0	13	0	1
160474	0	57	15	1	15	1	15	1	1
159011	1	54	11	1	11	1	11	1	1
162429	1	60	13	1	13	1	13	1	1
162462	0	87	21	0	21	0	21	0	1
162498	1	41	18	0	18	0	18	0	1
162512	0	65	16	0	16	0	16	0	1
162838	0	49	9	0	9	0	9	0	1
163294	1	51	13	1	13	1	13	1	1
163392	1	70	10	0	10	0	10	0	1
164084	0	93	15	1	15	1	15	1	1
164501	1	49	11	1	11	1	11	1	1
165207	1	70	16	0	16	0	16	0	1
165528	1	77	20	1	20	1	20	1	1
165734	1	52	6	0	6	0	6	0	1
165854	1	46	13	1	13	1	13	1	1
166116	1	47	11	1	11	1	11	1	1
166332	1	24	9	0	9	0	9	0	1
166637	1	46	16	0	16	0	16	0	1
167299	1	43	30	0	33	0	33	0	1
167348	1	49	24	0	24	0	24	0	1
164887	0	66	8	1	8	1	8	1	1
168473	1	41	12	0	12	0	12	0	1
168519	1	49	11	0	11	0	11	0	1

168917	1	47	30	0	33	0	33	0	1
131200	1	54	5	0	5	0	5	0	1
169635	0	38	20	0	20	0	20	0	1
169993	1	60	27	0	27	0	27	0	1
436008	1	75	3	1	3	1	3	1	1
170185	1	36	19	0	19	0	19	0	1
170377	1	63	2	1	2	1	2	1	1
170491	0	44	20	0	20	0	20	0	1
170517	1	53	7	0	7	0	7	0	1
170549	1	48	15	0	15	0	15	0	1
171074	0	57	17	0	17	0	17	0	1
171300	1	41	1	1	1	1	1	1	1
172155	0	76	2	0	2	0	2	0	1
172960	1	43	30	0	90	0	360	0	1
167481	1	52	12	1	12	1	12	1	1
173341	1	52	7	0	7	0	7	0	1
174005	1	43	12	1	12	1	12	1	1
174072	1	53	25	1	25	1	25	1	1
170579	1	74	7	1	7	1	7	1	1
174496	1	52	6	0	6	0	6	0	1
174883	1	35	10	1	10	1	10	1	1
171151	0	77	4	0	4	0	4	0	1
175361	1	63	17	0	17	0	17	0	1
175994	0	58	12	1	12	1	12	1	1
151073	0	68	5	1	5	1	5	1	1
176998	1	42	1	1	1	1	1	1	1
177157	1	57	19	0	19	0	19	0	1
94893	0	51	21	1	21	1	21	1	1
177666	1	41	21	0	21	0	21	0	1
178301	1	42	5	0	5	0	5	0	1
178662	1	56	12	1	12	1	12	1	1
166967	0	81	14	0	14	0	14	0	1
169552	1	59	8	0	8	0	8	0	1
179529	1	45	10	1	10	1	10	1	1
174286	0	78	28	1	28	1	28	1	1
179857	1	59	18	1	18	1	18	1	1
180373	1	48	11	0	11	0	11	0	1
180753	1	46	8	0	8	0	8	0	1
181135	1	46	21	0	21	0	21	0	1
181255	1	41	27	0	27	0	27	0	1
181948	1	52	13	0	13	0	13	0	1
181987	1	58	6	0	6	0	6	0	1
182698	1	42	7	0	7	0	7	0	1
183017	1	41	8	1	8	1	8	1	1
183276	1	47	17	1	17	1	17	1	1

146589	1	43	22	1	22	1	22	1	1
184092	0	62	7	0	7	0	7	0	1
184118	0	47	24	0	24	0	24	0	1
184232	1	74	8	1	8	1	8	1	1
184388	0	70	1	1	1	1	1	1	1
185539	1	64	8	1	8	1	8	1	1
185689	1	66	5	0	5	0	5	0	1
184902	0	65	13	1	13	1	13	1	1
185744	1	46	11	1	11	1	11	1	1
172344	1	47	10	1	10	1	10	1	1
165768	0	71	11	0	11	0	11	0	1
184178	1	58	10	1	10	1	10	1	1
187265	0	63	23	1	23	1	23	1	1
187312	1	40	8	0	8	0	8	0	1
152567	0	62	10	0	10	0	10	0	1
188289	1	47	29	1	29	1	29	1	1
188365	1	53	24	1	24	1	24	1	1
188699	1	56	12	1	12	1	12	1	1
188731	0	58	3	0	3	0	3	0	1
190329	0	64	10	1	10	1	10	1	1
190714	0	64	9	1	9	1	9	1	1
177756	0	67	8	1	8	1	8	1	1
191636	1	58	11	1	11	1	11	1	1
191731	1	69	13	1	13	1	13	1	1
191813	0	70	17	1	17	1	17	1	1
139868	1	82	15	1	15	1	15	1	1
192245	1	40	19	0	19	0	19	0	1
192526	1	53	30	0	90	0	360	0	1
192665	1	52	10	0	10	0	10	0	1
192686	1	68	25	1	25	1	25	1	1
192986	1	61	15	0	15	0	15	0	1
159610	1	57	3	0	3	0	3	0	1
193350	1	52	23	0	23	0	23	0	1
194241	0	65	26	0	26	0	26	0	1
194573	1	47	30	0	90	0	360	0	1
195059	1	47	11	1	11	1	11	1	1
179004	1	80	13	0	13	0	13	0	1
73199	1	48	7	0	7	0	7	0	1
196437	1	59	2	0	2	0	2	0	1
196700	1	72	2	0	2	0	2	0	1
194578	0	70	15	0	15	0	15	0	1
197237	0	53	25	1	25	1	25	1	1
197302	0	52	23	0	23	0	23	0	1
197550	0	64	7	0	7	0	7	0	1
197746	1	66	12	0	12	0	12	0	1

197995	0	60	6	0	6	0	6	0	1
197996	1	53	8	1	8	1	8	1	1
124812	1	42	30	0	90	0	360	0	1
198593	1	54	12	0	12	0	12	0	1
198822	1	52	14	0	14	0	14	0	1
166349	1	41	12	1	12	1	12	1	1
199300	1	52	11	0	11	0	11	0	1
193882	1	65	12	0	12	0	12	0	1
199423	1	76	11	1	11	1	11	1	1
199794	1	34	15	1	15	1	15	1	1
178190	0	66	10	0	10	0	10	0	1
179099	0	71	13	1	13	1	13	1	1
179789	1	64	10	0	10	0	10	0	1
201659	1	43	4	0	4	0	4	0	1
201727	1	54	30	0	32	0	32	0	1
202044	1	51	15	1	15	1	15	1	1
202159	1	31	7	0	7	0	7	0	1
202317	1	66	9	1	9	1	9	1	1
202997	0	71	8	1	8	1	8	1	1
128406	0	78	14	1	14	1	14	1	1
204330	1	50	7	0	7	0	7	0	1
204480	1	54	5	0	5	0	5	0	1
204744	1	65	13	1	13	1	13	1	1
206424	1	62	11	1	11	1	11	1	1
206961	1	38	2	0	2	0	2	0	1
207310	1	63	4	0	4	0	4	0	1
207524	1	47	10	1	10	1	10	1	1
208041	0	54	4	1	4	1	4	1	1
141333	0	79	14	1	14	1	14	1	1
208846	1	68	12	1	12	1	12	1	1
206887	0	52	7	0	7	0	7	0	1
210311	0	66	12	1	12	1	12	1	1
152073	0	71	4	0	4	0	4	0	1
210356	1	62	4	0	4	0	4	0	1
210368	1	61	1	0	1	0	1	0	1
155119	1	50	5	0	5	0	5	0	1
211077	1	54	24	1	24	1	24	1	1
211381	0	65	14	0	14	0	14	0	1
159971	1	63	30	0	90	0	360	0	1
211579	1	53	13	0	13	0	13	0	1
211705	1	75	3	0	3	0	3	0	1
211925	1	47	19	0	19	0	19	0	1
184530	1	64	5	0	5	0	5	0	1
212206	0	71	9	0	9	0	9	0	1
212286	0	41	17	0	17	0	17	0	1

212747	1	52	13	1	13	1	13	1	1
212818	1	41	8	0	8	0	8	0	1
212876	1	41	30	0	31	0	31	0	1
184676	1	52	10	0	10	0	10	0	1
207837	1	54	16	0	16	0	16	0	1
213973	1	75	16	0	16	0	16	0	1
186031	1	46	7	0	7	0	7	0	1
214090	0	72	21	1	21	1	21	1	1
214213	1	63	13	0	13	0	13	0	1
215111	1	51	18	0	18	0	18	0	1
110561	1	85	16	0	16	0	16	0	1
93480	0	64	28	0	28	0	28	0	1
146590	1	34	28	0	28	0	28	0	1
68858	1	60	28	1	28	1	28	1	1
167606	1	44	28	1	28	1	28	1	1
187731	0	70	28	0	28	0	28	0	1
103785	0	65	28	0	28	0	28	0	1
117368	1	40	29	0	29	0	29	0	1
136569	0	78	29	1	29	1	29	1	1
174419	1	49	29	0	29	0	29	0	1
207393	1	35	29	0	29	0	29	0	1
214679	1	56	29	0	29	0	29	0	1
95972	1	39	30	0	30	0	30	0	1
152970	0	47	30	0	30	0	30	0	1
181426	1	74	30	0	30	0	30	0	1
183775	1	61	30	0	30	0	30	0	1
153557	1	54	30	1	30	1	30	1	1
138544	1	51	30	0	31	0	31	0	1
152187	0	31	30	0	31	0	31	0	1
200184	0	62	30	0	31	1	31	1	1
209119	1	43	30	0	31	1	31	1	1
102270	1	45	30	0	32	1	32	1	1
113013	0	69	30	0	32	1	32	1	1
168065	0	62	30	0	32	0	32	0	1
190736	0	66	30	0	32	0	32	0	1
169074	1	48	30	0	32	0	32	0	1
114252	1	53	30	0	33	0	33	0	1
105707	0	75	30	0	33	0	33	0	1
171982	1	50	30	0	33	0	33	0	1
205577	1	47	30	0	33	0	33	0	1
180517	1	45	30	0	34	0	34	0	1
36495	1	78	30	0	34	1	34	1	1
207608	0	71	30	0	34	1	34	1	1
114938	1	59	30	0	35	0	35	0	1
130887	0	63	30	0	35	0	35	0	1

186837	0	49	30	0	35	0	35	0	1
172079	1	58	30	0	35	0	35	0	1
134346	0	57	30	0	36	1	36	1	1
136692	1	56	30	0	36	0	36	0	1
156331	1	68	30	0	36	0	36	0	1
172529	1	49	30	0	36	0	36	0	1
190422	1	45	30	0	36	0	36	0	1
193102	0	66	30	0	36	1	36	1	1
192100	1	44	30	0	36	1	36	1	1
155637	1	61	30	0	37	0	37	0	1
202120	1	63	30	0	37	0	37	0	1
209213	1	50	30	0	37	0	37	0	1
126199	0	60	30	0	37	1	37	1	1
150419	1	41	30	0	38	0	38	0	1
125315	1	47	30	0	38	0	38	0	1
157077	1	38	30	0	39	0	39	0	1
156883	1	52	30	0	39	0	39	0	1
191402	1	48	30	0	39	0	39	0	1
192723	1	36	30	0	39	0	39	0	1
203767	1	51	30	0	39	0	39	0	1
107109	1	78	30	0	39	1	39	1	1
182623	0	51	30	0	40	0	40	0	1
95784	1	71	30	0	41	0	41	0	1
114247	0	57	30	0	41	0	41	0	1
137210	1	57	30	0	41	0	41	0	1
157598	0	59	30	0	41	0	41	0	1
145281	1	41	30	0	41	1	41	1	1
174893	1	44	30	0	41	0	41	0	1
105689	0	76	30	0	42	1	42	1	1
149073	1	60	30	0	42	0	42	0	1
161844	0	57	30	0	42	0	42	0	1
194947	1	34	30	0	42	0	42	0	1
103828	1	66	30	0	43	0	43	0	1
106962	1	47	30	0	43	1	43	1	1
111667	1	30	30	0	43	0	43	0	1
114435	1	58	30	0	43	0	43	0	1
127254	1	46	30	0	43	1	43	1	1
134522	0	36	30	0	43	0	43	0	1
215489	1	33	30	0	43	0	43	0	1
111467	0	77	30	0	44	1	44	1	1
128933	1	67	30	0	44	0	44	0	1
154086	1	53	30	0	44	1	44	1	1
109902	1	40	30	0	45	0	45	0	1
126422	1	57	30	0	45	0	45	0	1
126667	1	51	30	0	45	1	45	1	1

125099	0	48	30	0	45	1	45	1	1
206703	1	38	30	0	45	0	45	0	1
184176	1	60	30	0	46	1	46	1	1
101785	1	51	30	0	47	0	47	0	1
136103	1	48	30	0	47	0	47	0	1
144989	1	70	30	0	47	0	47	0	1
96525	1	68	30	0	48	1	48	1	1
128352	1	48	30	0	48	0	48	0	1
210047	1	60	30	0	48	0	48	0	1
84937	0	69	30	0	49	1	49	1	1
111006	0	69	30	0	50	1	50	1	1
112891	1	45	30	0	50	0	50	0	1
133466	1	54	30	0	50	0	50	0	1
157962	1	47	30	0	50	0	50	0	1
171201	1	61	30	0	50	0	50	0	1
215011	1	68	30	0	50	0	50	0	1
102781	1	47	30	0	56	1	56	1	1
114719	0	48	30	0	51	0	51	0	1
111092	0	76	30	0	51	1	51	1	1
150349	1	53	30	0	51	1	51	1	1
168551	0	75	30	0	52	1	52	1	1
192213	1	64	30	0	52	0	52	0	1
132850	1	43	30	0	53	0	53	0	1
183861	0	65	30	0	54	0	54	0	1
188574	1	49	30	0	54	0	54	0	1
172431	1	46	30	0	55	0	55	0	1
185576	0	51	30	0	55	0	55	0	1
171709	0	78	30	0	55	0	55	0	1
99993	1	50	30	0	56	0	56	0	1
184947	1	32	30	0	56	0	56	0	1
128621	1	61	30	0	57	0	57	0	1
131577	1	50	30	0	57	0	57	0	1
170490	1	52	30	0	57	0	57	0	1
208619	0	64	30	0	57	0	57	0	1
102050	0	78	30	0	58	1	58	1	1
103873	1	56	30	0	58	1	58	1	1
134845	1	52	30	0	58	0	58	0	1
145451	0	51	30	0	59	0	59	0	1
206686	1	27	30	0	59	0	59	0	1
147671	1	64	30	0	60	0	60	0	1
135438	0	18	30	0	60	0	60	0	1
146435	1	58	30	0	61	0	61	0	1
158670	1	60	30	0	61	0	61	0	1
71738	0	31	30	0	62	0	62	0	1
107286	1	57	30	0	62	1	62	1	1

127888	0	48	30	0	62	0	62	0	1
153467	0	65	30	0	62	0	62	0	1
208584	1	30	30	0	63	0	63	0	1
124708	0	68	30	0	64	0	64	0	1
203417	1	49	30	0	64	0	64	0	1
207974	0	53	30	0	64	0	64	0	1
211626	1	51	30	0	64	0	64	0	1
134714	1	51	30	0	65	0	65	0	1
214824	1	65	30	0	65	0	65	0	1
101960	1	37	30	0	67	0	67	0	1
137471	0	54	30	0	67	0	67	0	1
107697	1	65	30	0	68	0	68	0	1
114651	0	67	30	0	68	0	68	0	1
175403	0	69	30	0	68	1	68	1	1
108797	1	53	30	0	68	0	68	0	1
169226	1	66	30	0	69	0	69	0	1
110178	1	40	30	0	70	1	70	1	1
122933	0	70	30	0	70	0	70	0	1
133412	1	44	30	0	70	0	70	0	1
165552	0	59	30	0	70	0	70	0	1
119393	1	52	30	0	71	0	71	0	1
201810	1	56	30	0	71	1	71	1	1
174396	0	73	30	0	71	0	71	0	1
200439	1	53	30	0	73	0	73	0	1
209659	1	46	30	0	74	0	74	0	1
133446	0	51	30	0	75	1	75	1	1
107188	0	50	30	0	75	1	75	1	1
97905	1	53	30	0	76	0	76	0	1
210595	1	59	30	0	77	0	77	0	1
186181	1	52	30	0	78	0	78	0	1
191334	0	53	30	0	78	0	78	0	1
180359	1	62	30	0	79	0	79	0	1
109552	1	31	30	0	80	0	80	0	1
122880	0	52	30	0	80	1	80	1	1
103282	1	51	30	0	81	1	81	1	1
196190	1	58	30	0	81	0	81	0	1
175479	1	54	30	0	82	1	82	1	1
182110	0	71	30	0	83	0	83	0	1
214266	1	60	30	0	86	0	86	0	1
101704	0	57	30	0	87	0	87	0	1
188916	1	35	30	0	87	0	87	0	1
121427	1	74	30	0	88	1	88	1	1
176189	0	69	30	0	88	1	88	1	1
193552	1	51	30	0	89	0	89	0	1
160258	1	51	30	0	90	0	90	0	1

206150	1	49	30	0	90	0	90	0	1
167274	1	47	30	0	90	0	91	1	1
214699	1	61	30	0	90	0	91	0	1
146339	1	60	30	0	90	0	92	0	1
111032	0	57	30	0	90	0	93	0	1
165880	1	52	30	0	90	0	93	0	1
210644	0	65	30	0	90	0	94	0	1
112732	0	67	30	0	90	0	95	0	1
154988	1	54	30	0	90	0	95	1	1
137173	0	59	30	0	90	0	96	1	1
206302	1	42	30	0	90	0	96	0	1
134173	1	56	30	0	90	0	99	0	1
183918	0	49	30	0	90	0	100	1	1
174715	0	53	30	0	90	0	102	0	1
76500	1	75	30	0	90	0	103	1	1
141254	1	46	30	0	90	0	104	0	1
112935	1	51	30	0	90	0	104	1	1
173271	0	52	30	0	90	0	104	0	1
102764	0	78	30	0	90	0	104	1	1
142708	1	62	30	0	90	0	107	1	1
162142	1	52	30	0	90	0	107	0	1
178506	1	55	30	0	90	0	107	0	1
150234	0	55	30	0	90	0	109	1	1
162038	0	65	30	0	90	0	109	0	1
127302	1	39	30	0	90	0	111	0	1
150920	1	50	30	0	90	0	111	0	1
201344	1	54	30	0	90	0	111	0	1
126003	0	58	30	0	90	0	114	0	1
111796	1	57	30	0	90	0	116	0	1
163201	1	49	30	0	90	0	116	0	1
208550	1	62	30	0	90	0	117	0	1
210367	1	78	30	0	90	0	117	0	1
215249	1	60	30	0	90	0	121	0	1
118642	1	53	30	0	90	0	122	0	1
129042	1	47	30	0	90	0	126	1	1
111294	1	49	30	0	90	0	126	0	1
139226	0	59	30	0	90	0	127	0	1
103726	1	45	30	0	90	0	127	0	1
202559	1	59	30	0	90	0	129	0	1
145521	1	62	30	0	90	0	129	0	1
170069	1	56	30	0	90	0	130	0	1
160855	0	51	30	0	90	0	130	0	1
195783	1	55	30	0	90	0	130	0	1
150817	1	39	30	0	90	0	135	0	1
170180	0	65	30	0	90	0	135	0	1

203328	1	54	30	0	90	0	135	0	1
188902	1	47	30	0	90	0	137	0	1
145788	1	38	30	0	90	0	139	0	1
202474	1	71	30	0	90	0	139	0	1
135196	1	57	30	0	90	0	140	0	1
215682	0	72	30	0	90	0	140	0	1
107187	1	45	30	0	90	0	141	0	1
145507	0	61	30	0	90	0	142	1	1
169436	1	56	30	0	90	0	143	0	1
172891	1	50	30	0	90	0	149	0	1
200908	1	77	30	0	90	0	150	0	1
211699	1	46	30	0	90	0	150	0	1
151759	0	57	30	0	90	0	150	0	1
133070	0	50	30	0	90	0	152	0	1
141583	1	51	30	0	90	0	152	0	1
107862	1	28	30	0	90	0	153	1	1
170463	1	55	30	0	90	0	153	0	1
193590	1	52	30	0	90	0	153	0	1
148473	1	54	30	0	90	0	155	1	1
108135	1	49	30	0	90	0	156	1	1
179234	0	62	30	0	90	0	156	1	1
190021	1	76	30	0	90	0	156	1	1
130986	1	58	30	0	90	0	157	0	1
145889	1	37	30	0	90	0	157	0	1
178673	1	48	30	0	90	0	157	1	1
102780	1	70	30	0	90	0	158	0	1
91155	0	57	30	0	90	0	160	1	1
160790	1	59	30	0	90	0	162	0	1
112862	1	53	30	0	90	0	163	0	1
117385	1	51	30	0	90	0	167	0	1
204747	1	54	30	0	90	0	168	0	1
163870	1	70	30	0	90	0	171	0	1
172852	1	47	30	0	90	0	171	0	1
131186	1	52	30	0	90	0	173	0	1
196206	0	72	30	0	90	0	173	0	1
170139	1	76	30	0	90	0	176	0	1
184611	1	60	30	0	90	0	177	1	1
213540	1	26	30	0	90	0	179	0	1
208530	1	48	30	0	90	0	180	0	1
104438	0	46	30	0	90	0	183	0	1
180478	1	42	30	0	90	0	183	0	1
140432	1	42	30	0	90	0	186	0	1
204712	1	43	30	0	90	0	186	0	1
117297	1	56	30	0	90	0	196	0	1
150756	0	60	30	0	90	0	197	0	1

119649	0	72	30	0	90	0	198	1	1
215586	1	66	30	0	90	0	198	0	1
213387	0	61	30	0	90	0	201	0	1
135383	1	48	30	0	90	0	202	0	1
161419	0	62	30	0	90	0	202	0	1
103528	1	50	30	0	90	0	204	0	1
160640	1	52	30	0	90	0	204	0	1
174975	1	78	30	0	90	0	205	0	1
215846	1	47	30	0	90	0	206	0	1
210307	1	44	30	0	90	0	210	0	1
185565	1	52	30	0	90	0	210	0	1
209224	1	64	30	0	90	0	212	0	1
172179	1	52	30	0	90	0	213	0	1
97035	1	72	30	0	90	0	214	1	1
170205	1	47	30	0	90	0	214	1	1
178016	1	59	30	0	90	0	214	0	1
202139	1	63	30	0	90	0	216	0	1
207456	0	60	30	0	90	0	217	0	1
214613	1	54	30	0	90	0	217	0	1
106914	1	60	30	0	90	0	218	0	1
213917	0	54	30	0	90	0	218	0	1
105445	0	54	30	0	90	0	219	0	1
177558	1	63	30	0	90	0	219	1	1
181397	1	54	30	0	90	0	220	0	1
164389	1	62	30	0	90	0	220	0	1
185149	1	52	30	0	90	0	222	0	1
143142	1	74	30	0	90	0	225	0	1
143748	0	63	30	0	90	0	225	0	1
211282	0	52	30	0	90	0	225	0	1
141606	0	76	30	0	90	0	226	1	1
357562	0	37	30	0	90	0	226	0	1
114877	1	46	30	0	90	0	227	0	1
113118	1	53	30	0	90	0	227	0	1
109609	0	58	22	0	22	0	22	0	1
104920	1	54	30	0	90	0	228	0	1
40218	0	50	30	0	90	0	228	0	1
141660	1	41	30	0	90	0	229	0	1
138692	1	72	30	0	90	0	229	0	1
101322	1	51	30	0	90	0	91	1	1
100266	0	59	30	0	90	0	230	0	1
214409	1	25	30	0	90	0	231	0	1
170910	0	60	30	0	90	0	237	0	1
203404	1	45	30	0	90	0	241	0	1
213038	0	70	30	0	90	0	241	0	1
164795	1	58	30	0	90	0	243	0	1

190232	1	57	30	0	90	0	243	0	1
195184	0	54	30	0	90	0	244	0	1
127852	1	64	30	0	90	0	246	0	1
103813	1	41	30	0	90	0	247	0	1
161382	1	66	30	0	90	0	248	0	1
113455	0	70	30	0	90	0	249	1	1
108798	1	56	30	0	90	0	251	1	1
206757	1	51	30	0	90	0	253	0	1
112031	1	71	30	0	90	0	253	0	1
190839	1	61	30	0	90	0	256	0	1
212015	0	80	30	0	90	0	258	0	1
130184	0	70	30	0	90	0	260	0	1
213519	1	40	30	0	90	0	264	0	1
109355	1	52	30	0	90	0	267	0	1
202840	1	55	30	0	90	0	268	0	1
210397	0	59	30	0	90	0	268	0	1
139977	1	79	30	0	90	0	269	0	1
211277	0	55	30	0	90	0	269	0	1
96497	1	29	30	0	90	0	271	1	1
143534	0	67	30	0	90	0	274	0	1
139308	1	48	30	0	90	0	277	0	1
211198	1	57	30	0	90	0	277	0	1
207229	0	56	30	0	90	0	279	0	1
195034	1	50	30	0	90	0	280	0	1
135592	0	66	30	0	90	0	281	0	1
210390	1	48	30	0	90	0	282	0	1
149437	0	74	30	0	90	0	283	1	1
182578	1	57	30	0	90	0	283	1	1
134325	1	59	30	0	90	0	285	0	1
188491	1	54	30	0	90	0	286	0	1
179235	1	61	30	0	90	0	290	0	1
194541	1	58	30	0	90	0	291	0	1
143429	1	57	30	0	90	0	292	0	1
179115	0	37	30	0	90	0	292	0	1
210192	1	46	30	0	90	0	293	0	1
165357	1	54	30	0	90	0	295	0	1
137385	1	60	30	0	90	0	296	0	1
201884	1	45	30	0	90	0	296	0	1
129018	0	62	30	0	90	0	304	1	1
194811	1	70	30	0	90	0	304	0	1
121073	0	64	30	0	90	0	304	1	1
126209	1	46	30	0	90	0	305	0	1
210385	1	35	30	0	90	0	306	0	1
183518	1	30	30	0	90	0	308	1	1
203624	1	60	30	0	90	0	308	0	1

202695	0	50	30	0	90	0	308	0	1
187955	1	63	30	0	90	0	309	0	1
154902	1	47	30	0	90	0	310	0	1
171308	1	52	30	0	90	0	311	1	1
139557	1	64	30	0	90	0	312	0	1
186123	1	56	30	0	90	0	316	0	1
120006	1	50	30	0	90	0	317	1	1
194038	1	54	30	0	90	0	318	0	1
150506	1	51	30	0	90	0	320	0	1
101086	1	56	30	0	90	0	322	0	1
211080	1	45	30	0	90	0	322	0	1
199796	1	63	30	0	90	0	326	1	1
201483	1	46	30	0	90	0	326	0	1
121890	1	57	30	0	90	0	327	0	1
202658	0	52	30	0	90	0	329	0	1
191362	1	46	30	0	90	0	329	0	1
210572	0	73	30	0	90	0	330	0	1
210080	1	49	30	0	90	0	334	0	1
91958	1	54	30	0	90	0	335	0	1
94277	1	53	30	0	90	0	360	0	1
200204	1	71	30	0	90	0	335	0	1
206561	1	62	30	0	90	0	336	0	1
114403	0	63	30	0	90	0	339	1	1
180103	1	57	30	0	90	0	340	0	1
156967	0	59	30	0	90	0	343	1	1
183639	1	64	30	0	90	0	343	1	1
145937	0	73	30	0	90	0	343	0	1
109854	1	46	30	0	90	0	347	1	1
200348	0	77	30	0	90	0	347	0	1
118965	0	61	30	0	90	0	349	1	1
166681	0	63	30	0	90	0	351	0	1
101739	1	50	30	0	90	0	352	1	1
123563	1	55	30	0	90	0	354	0	1
197663	0	75	30	0	90	0	355	0	1
111300	1	65	30	0	90	0	357	0	1
146679	1	55	30	0	90	0	357	0	1
203284	1	55	30	0	90	0	357	0	1
209648	1	62	30	0	90	0	357	0	1
117506	1	59	30	0	90	0	359	0	1
111352	0	57	30	0	90	0	360	0	1
87789	1	44	30	0	90	0	360	0	1
139242	1	78	30	0	90	0	360	0	1
182953	1	53	30	0	90	0	360	0	1
187514	0	79	30	0	90	0	360	0	1
200433	1	56	30	0	90	0	360	0	1

120189	1	35	30	0	90	0	360	0	1
206625	1	47	30	0	90	0	360	0	1
85855	1	66	30	0	90	0	360	0	1
161653	1	56	30	0	90	0	360	0	1
167880	1	66	30	0	90	0	360	0	1
192821	0	61	30	0	90	0	360	0	1
159358	1	61	30	0	90	0	360	0	1
100053	1	49	30	0	90	0	360	0	1
110885	1	56	30	0	90	0	360	0	1
133685	1	56	30	0	90	0	360	0	1
205968	1	52	30	0	90	0	360	0	1
120511	1	34	30	0	90	0	360	0	1
194344	0	71	30	0	90	0	360	0	1
164906	1	52	30	0	90	0	360	0	1
101847	1	70	30	0	90	0	360	0	1
119796	1	75	30	0	90	0	360	0	1
201325	1	38	30	0	90	0	360	0	1
147174	1	49	30	0	90	0	360	0	1
169700	1	46	30	0	90	0	360	0	1
188915	1	76	30	0	90	0	360	0	1
180126	1	32	30	0	90	0	360	0	1
112717	1	62	30	0	90	0	360	0	1
153992	1	51	30	0	90	0	360	0	1
204886	1	59	30	0	90	0	360	0	1
206988	1	68	30	0	90	0	360	0	1
186792	0	50	30	0	90	0	360	0	1
152335	1	46	30	0	90	0	360	0	1
127395	0	58	30	0	90	0	360	0	1
156670	1	43	30	0	90	0	360	0	1
166145	1	54	30	0	90	0	360	0	1
206163	1	43	30	0	90	0	360	0	1
151288	1	44	30	0	90	0	360	0	1
126617	0	70	30	0	90	0	360	0	1
77254	1	68	30	0	90	0	360	0	1
169427	1	45	30	0	90	0	360	0	1
171424	1	53	30	0	90	0	360	0	1
206967	1	57	30	0	90	0	360	0	1
206626	1	55	30	0	90	0	360	0	1
181968	1	42	30	0	90	0	360	0	1
131286	1	60	30	0	90	0	360	0	1
197405	1	44	30	0	90	0	360	0	1
146478	1	50	30	0	90	0	360	0	1
171777	1	71	30	0	90	0	360	0	1
200505	0	66	30	0	90	0	360	0	1
179085	1	38	30	0	90	0	360	0	1

205141	1	38	30	0	90	0	360	0	1
206741	0	53	30	0	90	0	360	0	1
144670	1	53	30	0	90	0	360	0	1
205706	1	42	30	0	90	0	360	0	1
202273	1	52	30	0	90	0	360	0	1
132755	1	51	30	0	90	0	360	0	1
131679	0	71	30	0	90	0	360	0	1
102340	1	51	30	0	90	0	360	0	1
103594	0	77	30	0	90	0	230	1	1
140194	1	43	30	0	90	0	360	0	1
139475	0	74	30	0	90	0	360	0	1
142785	1	58	30	0	90	0	360	0	1
111538	0	51	30	0	90	0	360	0	1
148590	1	50	30	0	90	0	360	0	1
199577	1	44	30	0	90	0	360	0	1
132485	1	54	30	0	90	0	360	0	1
109099	1	49	30	0	90	0	360	0	1
198731	0	62	30	0	90	0	360	0	1
184881	1	57	30	0	90	0	360	0	1
161529	1	51	30	0	90	0	360	0	1
156725	1	45	30	0	90	0	360	0	1
71085	1	54	30	0	90	0	360	0	1
200719	1	48	30	0	90	0	360	0	1
195665	1	37	30	0	90	0	360	0	1
36464	0	53	30	0	90	0	360	0	1
156486	1	59	30	0	90	0	360	0	1
182811	0	71	30	0	90	0	360	0	1
169191	1	49	30	0	90	0	360	0	1
139846	1	52	30	0	90	0	360	0	1
163625	1	29	30	0	90	0	360	0	1
197815	0	70	30	0	90	0	360	0	1
154511	1	62	30	0	90	0	360	0	1
132469	1	43	30	0	90	0	360	0	1
165151	1	47	30	0	90	0	360	0	1
156743	1	46	30	0	90	0	360	0	1
194943	0	74	30	0	90	0	360	0	1
192591	1	54	30	0	90	0	360	0	1
169747	1	60	30	0	90	0	360	0	1
213075	1	68	30	0	90	0	360	0	1
118911	1	49	30	0	90	0	360	0	1
139149	0	68	30	0	90	0	360	0	1
143188	1	51	30	0	90	0	360	0	1
118533	1	45	30	0	90	0	360	0	1
201144	0	75	30	0	90	0	360	0	1
202368	1	55	30	0	90	0	360	0	1

165180	1	80	30	0	90	0	360	0	1
114814	1	60	30	0	90	0	360	0	1
107748	1	54	30	0	90	0	360	0	1
194282	1	46	30	0	90	0	360	0	1
100910	1	55	30	0	90	0	360	0	1
161892	1	52	30	0	90	0	360	0	1
115758	1	63	30	0	90	0	360	0	1
111556	1	45	30	0	90	0	360	0	1
127166	1	59	30	0	90	0	360	0	1
191633	1	38	30	0	90	0	360	0	1
148788	1	64	30	0	90	0	360	0	1
184255	0	65	30	0	90	0	360	0	1
191826	1	45	30	0	90	0	360	0	1
156365	1	80	30	0	90	0	360	0	1
133042	1	46	30	0	90	0	360	0	1
198493	1	51	30	0	90	0	360	0	1
103633	1	44	30	0	90	0	360	0	1
197598	0	60	30	0	90	0	360	0	1
170938	1	55	30	0	90	0	360	0	1
162001	0	58	30	0	90	0	360	0	1
192053	0	70	30	0	90	0	360	0	1
129099	0	55	30	0	90	0	360	0	1
139935	1	41	30	0	90	0	360	0	1
192156	1	38	30	0	90	0	360	0	1
188231	1	53	30	0	90	0	360	0	1
130089	0	50	30	0	90	0	360	0	1
148347	1	55	30	0	90	0	360	0	1
140373	1	64	30	0	90	0	360	0	1
175761	1	37	30	0	90	0	360	0	1
198052	1	49	30	0	90	0	360	0	1
65986	1	66	30	0	90	0	360	0	1
188677	1	35	30	0	90	0	360	0	1
159931	0	69	30	0	90	0	360	0	1
106768	1	51	30	0	90	0	360	0	1
115813	1	41	30	0	90	0	360	0	1
54895	1	59	30	0	90	0	360	0	1
112627	1	61	30	0	90	0	360	0	1
127347	1	67	30	0	90	0	360	0	1
99620	0	66	30	0	90	0	360	0	1
180206	0	56	30	0	90	0	360	0	1
111154	1	49	30	0	90	0	360	0	1
196131	1	45	30	0	90	0	360	0	1
197736	1	35	30	0	90	0	360	0	1
103400	0	80	30	0	90	0	360	0	1
148777	1	52	30	0	90	0	360	0	1

173324	1	47	30	0	90	0	360	0	1
164082	1	44	30	0	90	0	360	0	1
179974	1	56	30	0	90	0	360	0	1
104151	1	54	30	0	90	0	360	0	1
189094	1	31	30	0	90	0	360	0	1
178360	1	72	30	0	90	0	360	0	1
96557	0	79	30	0	90	0	360	0	1
169744	1	48	30	0	90	0	360	0	1
113915	1	62	30	0	90	0	360	0	1
195860	1	48	30	0	90	0	360	0	1
125340	0	69	30	0	90	0	360	0	1
89609	1	45	30	0	90	0	360	0	1
129085	0	70	30	0	90	0	360	0	1
121795	1	53	30	0	90	0	360	0	1
192643	1	49	30	0	90	0	360	0	1
102692	1	49	30	0	90	0	360	0	1
187659	1	48	30	0	90	0	360	0	1
118141	0	74	30	0	90	0	360	0	1
193530	0	56	30	0	90	0	360	0	1
188754	1	63	30	0	90	0	360	0	1
33378	1	49	30	0	90	0	360	0	1
189053	0	71	30	0	90	0	360	0	1
166908	1	53	30	0	90	0	360	0	1
116888	1	64	30	0	90	0	360	0	1
92619	1	53	30	0	90	0	360	0	1
192951	1	46	30	0	90	0	360	0	1
192898	1	62	30	0	90	0	360	0	1
192945	1	35	30	0	90	0	360	0	1
151963	1	48	30	0	90	0	360	0	1
132898	1	64	30	0	90	0	360	0	1
154246	1	59	30	0	90	0	360	0	1
127301	1	46	30	0	90	0	360	0	1
190659	1	39	30	0	90	0	360	0	1
192305	1	50	30	0	90	0	360	0	1
192382	0	62	30	0	90	0	360	0	1
159604	1	47	30	0	90	0	360	0	1
189872	0	71	30	0	90	0	360	0	1
175950	1	41	30	0	90	0	360	0	1
120489	1	51	30	0	90	0	360	0	1
179952	0	77	30	0	90	0	360	0	1
189178	1	53	30	0	90	0	360	0	1
122365	1	60	30	0	90	0	360	0	1
156529	1	53	30	0	90	0	360	0	1
140085	0	72	30	0	90	0	360	0	1
175469	1	52	30	0	90	0	360	0	1

151319	0	77	30	0	90	0	360	0	1
145256	1	53	30	0	90	0	360	0	1
181060	1	45	30	0	90	0	360	0	1
190072	0	62	30	0	90	0	360	0	1
129153	1	41	30	0	90	0	360	0	1
188261	1	52	30	0	90	0	360	0	1
134439	1	54	30	0	90	0	360	0	1
148876	1	43	30	0	90	0	360	0	1
87138	0	74	30	0	90	0	360	0	1
99826	1	56	30	0	90	0	360	0	1
162477	0	73	30	0	90	0	360	0	1
115449	1	71	30	0	90	0	360	0	1
142960	1	43	30	0	90	0	360	0	1
144282	1	37	30	0	90	0	360	0	1
175689	1	70	30	0	90	0	360	0	1
177180	1	56	30	0	90	0	360	0	1
130692	1	70	30	0	90	0	360	0	1
132762	1	62	30	0	90	0	360	0	1
85357	1	72	30	0	90	0	360	0	1
174306	1	65	30	0	90	0	360	0	1
155679	1	41	30	0	90	0	360	0	1
101600	0	64	30	0	90	0	360	0	1
103418	1	48	30	0	90	0	360	0	1
174950	1	50	30	0	90	0	360	0	1
170019	1	56	30	0	90	0	360	0	1
144227	1	63	30	0	90	0	360	0	1
160295	0	54	30	0	90	0	360	0	1
152772	0	63	30	0	90	0	360	0	1
94420	0	74	30	0	90	0	360	0	1
125266	1	65	30	0	90	0	360	0	1
144221	1	71	30	0	90	0	360	0	1
153571	1	46	30	0	90	0	360	0	1
104722	1	53	30	0	90	0	360	0	1
99141	1	78	30	0	90	0	360	0	1
106339	0	64	30	0	90	0	360	0	1
98668	0	68	30	0	90	0	360	0	1
140640	1	48	30	0	90	0	360	0	1
169254	1	40	30	0	90	0	360	0	1
98095	1	47	30	0	90	0	360	0	1
185015	1	46	30	0	90	0	360	0	1
150752	1	63	30	0	90	0	360	0	1
179227	1	47	30	0	90	0	360	0	1
157654	1	66	30	0	90	0	360	0	1
183336	1	55	30	0	90	0	360	0	1
151021	1	56	30	0	90	0	360	0	1

171743	1	30	30	0	90	0	360	0	1
129492	0	52	30	0	90	0	360	0	1
93376	1	43	30	0	90	0	360	0	1
105059	1	66	30	0	90	0	360	0	1
172542	1	62	30	0	90	0	360	0	1
146666	1	42	30	0	90	0	360	0	1
114410	1	61	30	0	90	0	360	0	1
125609	1	33	30	0	90	0	360	0	1
174935	0	60	30	0	90	0	360	0	1
165406	0	61	30	0	90	0	360	0	1
123551	1	51	30	0	90	0	360	0	1
101411	1	47	30	0	90	0	360	0	1
168209	1	59	30	0	90	0	360	0	1
101900	1	57	30	0	90	0	360	0	1
155375	0	65	30	0	90	0	360	0	1
140752	1	39	30	0	90	0	360	0	1
181918	1	56	30	0	90	0	360	0	1
99612	0	66	30	0	90	0	360	0	1
170393	1	33	30	0	90	0	360	0	1
164976	1	32	30	0	90	0	360	0	1
175945	1	29	30	0	90	0	360	0	1
104425	1	60	30	0	90	0	360	0	1
158125	1	31	30	0	90	0	360	0	1
171206	1	40	30	0	90	0	360	0	1
131127	1	60	30	0	90	0	360	0	1
134254	1	40	30	0	90	0	360	0	1
176225	0	74	30	0	90	0	360	0	1
126568	1	48	30	0	90	0	360	0	1
128203	1	61	30	0	90	0	360	0	1
128161	1	63	30	0	90	0	360	0	1
152178	0	58	30	0	90	0	360	0	1
95005	1	51	30	0	90	0	360	0	1
173143	0	69	30	0	90	0	360	0	1
122823	1	44	30	0	90	0	360	0	1
175026	1	53	30	0	90	0	360	0	1
172332	1	47	30	0	90	0	360	0	1
144791	1	58	30	0	90	0	360	0	1
173072	0	68	30	0	90	0	360	0	1
146735	1	47	30	0	90	0	360	0	1
178302	1	46	30	0	90	0	360	0	1
170485	1	40	30	0	90	0	360	0	1
157663	0	58	30	0	90	0	360	0	1
144164	0	64	30	0	90	0	360	0	1
123952	1	39	30	0	90	0	360	0	1
144148	1	55	30	0	90	0	360	0	1

121148	1	35	30	0	90	0	360	0	1
156361	0	71	30	0	90	0	360	0	1
170239	0	65	30	0	90	0	360	0	1
167975	0	64	30	0	90	0	360	0	1
156840	1	58	30	0	90	0	360	0	1
106248	1	68	30	0	90	0	234	1	1
132949	1	57	30	0	90	0	360	0	1
162863	1	38	30	0	90	0	360	0	1
99881	1	43	30	0	90	0	360	0	1
169627	1	37	30	0	90	0	360	0	1
131763	0	74	30	0	90	0	360	0	1
108110	1	57	30	0	90	0	360	0	1
111867	1	57	30	0	90	0	360	0	1
145527	0	61	30	0	90	0	360	0	1
160558	1	45	30	0	90	0	360	0	1
135084	1	45	30	0	90	0	360	0	1
168531	1	60	30	0	90	0	360	0	1
170071	1	64	30	0	90	0	360	0	1
113203	1	46	30	0	90	0	360	0	1
141145	1	68	30	0	90	0	360	0	1
146475	1	58	30	0	90	0	360	0	1
111937	1	50	30	0	90	0	360	0	1
155355	0	68	30	0	90	0	360	0	1
154922	1	40	30	0	90	0	360	0	1
164307	1	56	30	0	90	0	360	0	1
115900	0	70	30	0	90	0	360	0	1
111396	1	61	30	0	90	0	360	0	1
131662	1	62	30	0	90	0	360	0	1
357952	1	53	30	0	90	0	360	0	1
166814	1	44	30	0	90	0	360	0	1
167036	1	57	30	0	90	0	360	0	1
139771	1	66	30	0	90	0	360	0	1
164741	1	55	30	0	90	0	360	0	1
142036	1	55	30	0	90	0	360	0	1
110907	1	56	30	0	90	0	360	0	1
126026	1	58	30	0	90	0	360	0	1
151745	1	37	30	0	90	0	360	0	1
163726	0	62	30	0	90	0	360	0	1
98807	1	50	30	0	90	0	360	0	1
120097	0	56	30	0	90	0	360	0	1
163355	0	74	30	0	90	0	360	0	1
157187	1	51	30	0	90	0	360	0	1
146483	1	63	30	0	90	0	360	0	1
112892	1	65	30	0	90	0	360	0	1
157029	1	56	30	0	90	0	360	0	1

108715	1	63	30	0	90	0	360	0	1
163059	1	39	30	0	90	0	360	0	1
102222	0	67	30	0	90	0	360	0	1
88349	1	60	30	0	90	0	360	0	1
121234	1	45	30	0	90	0	360	0	1
117747	1	55	30	0	90	0	360	0	1
112807	1	66	30	0	90	0	360	0	1
101163	1	69	30	0	90	0	360	0	1
89520	1	70	30	0	90	0	360	0	1
163249	1	44	30	0	90	0	360	0	1
155071	1	60	30	0	90	0	360	0	1
105228	1	68	30	0	90	0	360	0	1
144408	1	51	30	0	90	0	360	0	1
129011	1	41	30	0	90	0	360	0	1
115853	1	62	30	0	90	0	360	0	1
158646	1	53	30	0	90	0	360	0	1
155457	1	48	30	0	90	0	360	0	1
138096	1	52	30	0	90	0	360	0	1
91106	1	46	30	0	90	0	360	0	1
132343	1	29	30	0	90	0	360	0	1
154831	1	59	30	0	90	0	360	0	1
161966	1	44	30	0	90	0	360	0	1
121379	0	62	30	0	90	0	360	0	1
131216	0	61	30	0	90	0	360	0	1
161111	1	45	30	0	90	0	360	0	1
100847	0	54	30	0	90	0	360	0	1
143378	1	32	30	0	90	0	360	0	1
156703	1	54	30	0	90	0	360	0	1
123202	0	52	30	0	90	0	360	0	1
123923	1	48	30	0	90	0	360	0	1
101400	1	55	30	0	90	0	360	0	1
159502	1	52	30	0	90	0	360	0	1
153739	1	49	30	0	90	0	360	0	1
110882	1	58	30	0	90	0	360	0	1
156371	1	43	30	0	90	0	360	0	1
103968	1	37	30	0	90	0	360	0	1
146561	1	50	30	0	90	0	360	0	1
145820	1	42	30	0	90	0	360	0	1
156384	1	58	30	0	90	0	360	0	1
154717	1	64	30	0	90	0	360	0	1
49212	1	53	30	0	90	0	360	0	1
111816	0	55	30	0	90	0	360	0	1
156328	1	57	30	0	90	0	360	0	1
157817	1	57	30	0	90	0	360	0	1
134294	1	56	30	0	90	0	360	0	1

103243	1	52	30	0	90	0	360	0	1
104327	1	52	30	0	90	0	360	0	1
107021	1	48	30	0	90	0	360	0	1
128794	1	53	30	0	90	0	360	0	1
135789	1	79	30	0	90	0	360	0	1
144997	1	63	30	0	90	0	360	0	1
154702	1	53	30	0	90	0	360	0	1
129908	1	41	30	0	90	0	360	0	1
115725	1	64	30	0	90	0	360	0	1
154704	1	62	30	0	90	0	360	0	1
108662	1	36	30	0	90	0	360	0	1
107162	1	43	30	0	90	0	360	0	1
112944	1	39	30	0	90	0	360	0	1
97943	1	39	30	0	90	0	360	0	1
139519	0	48	30	0	90	0	360	0	1
152457	1	43	30	0	90	0	360	0	1
102215	1	53	30	0	90	0	360	0	1
152287	0	52	30	0	90	0	360	0	1
133000	1	53	30	0	90	0	360	0	1
118900	1	71	30	0	90	0	360	0	1
116997	1	62	30	0	90	0	360	0	1
99491	1	59	30	0	90	0	360	0	1
100322	1	56	30	0	90	0	360	0	1
110540	1	47	30	0	90	0	360	0	1
130045	1	60	30	0	90	0	360	0	1
102277	1	41	30	0	90	0	360	0	1
109402	0	67	30	0	90	0	360	0	1
147142	1	57	30	0	90	0	360	0	1
117986	1	49	30	0	90	0	360	0	1
102176	1	37	30	0	90	0	360	0	1
112128	1	58	30	0	90	0	360	0	1
147133	1	45	30	0	90	0	360	0	1
139684	0	73	30	0	90	0	360	0	1
130082	0	52	30	0	90	0	360	0	1
146336	1	35	30	0	90	0	360	0	1
109957	1	46	30	0	90	0	360	0	1
145191	1	65	30	0	90	0	360	0	1
138711	1	53	30	0	90	0	360	0	1
107052	1	47	30	0	90	0	360	0	1
124565	1	51	30	0	90	0	360	0	1
129566	0	47	30	0	90	0	360	0	1
140040	1	54	30	0	90	0	360	0	1
115041	1	44	30	0	90	0	360	0	1
142224	0	59	30	0	90	0	360	0	1
103622	1	42	30	0	90	0	360	0	1

91635	0	69	30	0	90	0	360	0	1
122838	1	54	30	0	90	0	360	0	1
124205	1	39	30	0	90	0	360	0	1
132326	0	59	30	0	90	0	360	0	1
132933	1	34	30	0	90	0	360	0	1
112690	0	72	30	0	90	0	360	0	1
105045	1	66	30	0	90	0	360	0	1
98024	0	59	30	0	90	0	360	0	1
134851	1	52	30	0	90	0	360	0	1
132555	1	62	30	0	90	0	360	0	1
136840	1	49	30	0	90	0	360	0	1
118293	1	52	30	0	90	0	360	0	1
101495	0	65	30	0	90	0	360	0	1
130568	1	40	30	0	90	0	360	0	1
132165	1	55	30	0	90	0	360	0	1
111056	1	51	30	0	90	0	360	0	1
228124	1	42	30	0	90	0	360	0	1
131460	1	51	30	0	90	0	360	0	1
59337	1	61	30	0	90	0	360	0	1
129335	1	49	30	0	90	0	360	0	1
128736	1	59	30	0	90	0	360	0	1
123253	1	51	30	0	90	0	360	0	1
129523	1	52	30	0	90	0	360	0	1
118606	1	37	30	0	90	0	360	0	1
112927	1	45	30	0	90	0	360	0	1
112235	0	36	30	0	90	0	360	0	1
111201	1	49	30	0	90	0	360	0	1
128849	1	46	30	0	90	0	360	0	1
111992	1	49	30	0	90	0	360	0	1
127967	0	68	30	0	90	0	360	0	1
121518	1	44	30	0	90	0	360	0	1
125254	0	72	30	0	90	0	360	0	1
124667	0	47	30	0	90	0	360	0	1
124446	1	48	30	0	90	0	360	0	1
123823	1	49	30	0	90	0	360	0	1
111834	1	46	30	0	90	0	360	0	1
92322	1	47	30	0	90	0	360	0	1
115851	1	58	30	0	90	0	360	0	1
118739	1	54	30	0	90	0	360	0	1
117815	1	75	30	0	90	0	360	0	1
116829	1	55	30	0	90	0	360	0	1
107643	1	42	30	0	90	0	360	0	1
99144	1	46	30	0	90	0	360	0	1
110048	1	49	30	0	90	0	360	0	1
115915	1	42	30	0	90	0	360	0	1

103113	1	58	30	0	90	0	360	0	1
110153	0	63	30	0	90	0	360	0	1
114552	1	47	30	0	90	0	360	0	1
111142	1	62	30	0	90	0	360	0	1
110530	1	49	30	0	90	0	360	0	1
112574	1	43	30	0	90	0	360	0	1
109986	1	46	30	0	90	0	360	0	1
108970	1	55	30	0	90	0	360	0	1
108386	0	54	30	0	90	0	360	0	1
141007	1	34	30	0	90	0	360	0	1
137313	1	44	30	0	90	0	360	0	1
139092	1	57	30	0	90	0	360	0	1
104311	0	50	30	0	90	0	360	0	1
142389	0	52	30	0	90	0	360	0	1
135892	1	80	3	1	3	1	3	1	1
136454	1	38	3	1	3	1	3	1	1
164771	1	40	3	1	3	1	3	1	1
129327	1	56	3	1	3	1	3	1	1
179261	1	75	3	1	3	1	3	1	1
187839	1	49	3	1	3	1	3	1	1
202287	1	79	3	1	3	1	3	1	1
109977	1	28	3	1	3	1	3	1	1
123257	1	58	4	1	4	1	4	1	1
154911	1	46	4	1	4	1	4	1	1
167418	1	59	4	1	4	1	4	1	1
102974	1	51	5	1	5	1	5	1	1
142292	1	40	5	1	5	1	5	1	1
178464	0	67	5	1	5	1	5	1	1
146079	1	53	5	1	5	1	5	1	1
117655	1	44	6	1	6	1	6	1	1
186542	1	48	6	1	6	1	6	1	1
198571	1	71	6	1	6	1	6	1	1
117894	1	55	6	1	6	1	6	1	1
111780	1	55	7	1	7	1	7	1	1
111113	1	70	30	0	90	0	360	0	1
129634	1	54	7	1	7	1	7	1	1
117418	0	56	7	1	7	1	7	1	1
139497	1	53	7	1	7	1	7	1	1
167539	1	60	7	1	7	1	7	1	1
198351	0	79	7	1	7	1	7	1	1
101611	1	44	8	1	8	1	8	1	1
100430	1	73	8	1	8	1	8	1	1
130941	0	62	8	1	8	1	8	1	1
178208	0	61	8	1	8	1	8	1	1
140849	1	54	9	1	9	1	9	1	1

154549	1	44	9	1	9	1	9	1	1
191001	1	54	9	1	9	1	9	1	1
196202	0	77	9	1	9	1	9	1	1
103390	0	64	10	1	10	1	10	1	1
113583	1	57	10	1	10	1	10	1	1
119851	1	35	10	1	10	1	10	1	1
133364	0	48	10	1	10	1	10	1	1
206145	1	54	10	1	10	1	10	1	1
209427	1	61	10	1	10	1	10	1	1
142593	1	46	11	1	11	1	11	1	1
183906	0	57	11	1	11	1	11	1	1
141031	1	48	12	1	12	1	12	1	1
112113	1	71	12	1	12	1	12	1	1
146559	1	45	12	1	12	1	12	1	1
101941	0	30	12	1	12	1	12	1	1
103893	0	55	13	1	13	1	13	1	1
138392	0	59	14	1	14	1	14	1	1
142756	1	60	14	1	14	1	14	1	1
106312	0	60	15	1	15	1	15	1	1
140705	1	44	16	1	16	1	16	1	1
98310	0	59	18	1	18	1	18	1	1
120374	1	68	18	1	18	1	18	1	1
145409	1	75	18	1	18	1	18	1	1
103020	0	68	20	1	20	1	20	1	1
133205	1	59	20	1	20	1	20	1	1
155468	0	53	20	1	20	1	20	1	1
92897	0	54	21	1	21	1	21	1	1
143259	1	59	21	1	21	1	21	1	1
209951	0	69	23	1	23	1	23	1	1
154879	1	54	24	1	24	1	24	1	1
144584	0	33	24	1	24	1	24	1	1
94099	1	42	25	1	25	1	25	1	1
209967	1	43	25	1	25	1	25	1	1
116046	0	48	25	1	25	1	25	1	1
143001	0	69	26	1	26	1	26	1	1
133338	1	70	26	1	26	1	26	1	1
111188	1	36	27	1	27	1	27	1	1
139765	1	30	27	1	27	1	27	1	1
166830	1	44	27	1	27	1	27	1	1
167606	1	44	28	1	28	1	28	1	1
174955	0	57	30	0	90	0	360	0	1
129639	1	48	30	0	90	0	360	0	1

GIB	SBP	ascites	hypertensi	diabetes	HE	ALT	AST	TBILumo	ALB
1	0	1	1	0	2	53.7	151.5	75.5	26.2
1	0	1	0	0	1	22.4	30.7	115.4	16.8
0	0	1	0	0	1	29.1	56.1	43.0	22.5
1	0	0	0	0	1	32.5	35.6	20.6	12.7
0	0	0	0	0	2	652.8	1548.5	53.3	9.6
1	0	1	0	0	2	10.8	23.3	8.1	12.6
1	0	1	0	0	1	15.4	46.6	149.4	26.4
1	0	1	0	0	2	12.7	24.1	85.4	23.0
1	0	1	0	0	2	35.0	80.9	230.4	25.7
1	0	1	0	1	1	110.1	151.3	18.1	14.7
1	0	1	1	0	1	14.5	27.0	8.3	25.1
1	0	1	0	1	1	62.9	244.2	74.2	29.7
0	1	1	0	1	1	67.9	208.1	265.6	19.1
1	0	1	0	0	2	425.1	280.3	328.5	24.3
1	1	1	0	0	1	21.6	99.5	177.4	21.1
1	0	0	0	1	2	332.3	704.4	29.3	27.4
1	0	1	0	0	2	29.2	63.3	336.5	23.2
0	0	1	0	0	2	41.4	116.1	137.4	23.6
1	0	0	1	1	1	31.4	21.0	10.5	28.5
0	0	1	0	0	1	52.7	55.3	373.2	40.7
0	0	1	0	0	1	60.4	137.9	466.4	29.6
1	0	1	0	0	1	271.4	401.0	184.0	18.1
1	0	1	0	0	2	22.6	37.6	50.5	11.3
1	0	0	1	1	1	1491.9	1554.6	84.1	21.8
1	0	1	0	1	2	22.3	42.6	124.1	18.6
1	0	1	0	1	2	67.8	172.1	318.4	32.0
0	0	0	1	1	2	51.6	66.7	17.0	28.0
0	0	1	1	0	2	64.5	115.9	154.8	17.1
0	1	1	1	1	1	19.8	39.0	10.9	29.0
0	0	1	1	1	1	121.0	309.8	515.9	32.7
1	0	1	0	0	1	66.6	41.7	272.5	28.1
1	0	1	0	0	1	99.0	95.5	252.2	21.6
1	0	0	0	1	2	21.2	36.4	158.1	26.7
1	1	1	0	1	1	18.1	42.6	235.1	23.1
0	0	1	1	1	1	68.5	273.1	129.1	22.6
0	0	0	0	1	1	159.1	620.4	303.7	29.0
0	0	1	0	0	2	686.5	752.7	498.9	41.0
1	0	1	0	0	1	126.4	176.9	202.7	23.3
0	0	0	0	0	2	17.6	24.5	22.5	33.1
0	0	1	0	0	1	48.6	118.6	35.2	19.2
0	0	0	0	0	1	148.4	105.7	344.6	29.9
1	0	1	0	0	2	321.6	628.2	469.4	30.6
1	0	0	0	0	1	161.2	106.5	83.9	27.2
0	0	1	0	1	1	23.3	43.9	55.6	37.8

0	0	1	0	0	1	206.6	253.2	447.5	27.5
0	0	1	0	0	1	20.9	22.3	26.4	35.5
0	0	1	0	0	1	1157.3	2429.7	56.0	23.6
0	0	1	1	0	2	19.1	33.7	45.4	31.5
0	0	1	0	0	2	25.8	33.8	52.0	42.6
1	0	1	0	0	1	31.3	117.1	95.2	31.7
0	1	1	0	0	1	85.2	128.7	530.6	28.1
1	0	1	0	1	1	17.4	29.6	30.2	30.6
1	0	1	0	1	1	44.3	82.6	505.1	22.4
0	0	1	1	0	1	52.2	103.1	433.5	25.6
0	0	0	0	0	2	54.0	64.2	47.1	35.2
0	1	1	0	0	2	14.4	40.1	554.0	22.7
0	0	1	1	0	1	39.5	45.8	52.9	29.4
0	1	1	0	0	1	27.2	51.2	141.2	25.3
0	0	1	0	0	2	350.6	693.9	541.8	21.8
0	0	1	0	0	1	99.9	143.1	460.8	24.6
0	0	1	0	0	1	30.3	58.1	133.4	20.2
0	0	1	1	0	1	129.3	384.8	368.7	25.5
0	0	1	0	0	2	43.9	90.0	515.8	15.4
0	0	1	0	1	2	77.1	101.5	53.6	18.1
0	1	1	0	0	2	64.3	161.9	409.7	19.7
0	0	1	0	0	1	73.2	60.7	170.4	22.4
0	0	1	0	1	1	75.1	109.8	39.3	20.3
0	0	1	0	0	1	38.1	74.6	156.7	22.4
1	0	1	0	0	1	31.2	176.7	433.4	14.8
0	0	0	0	0	1	21.6	66.8	75.3	25.2
0	0	1	0	0	1	98.2	161.2	189.7	30.8
0	0	1	0	0	1	19.6	31.1	30.9	27.1
0	0	1	0	0	1	204.5	341.2	560.3	33.6
0	0	0	0	0	1	36.4	57.6	92.1	28.3
1	0	1	0	0	1	76.9	231.6	557.2	20.0
0	0	1	0	1	1	272.3	255.8	955.4	34.0
1	0	1	0	0	1	30.1	45.1	389.2	18.2
1	0	0	0	0	2	41.8	36.9	38.2	25.4
0	0	1	0	0	1	26.2	62.4	30.6	23.8
0	0	1	0	0	1	206.3	381.8	557.7	21.0
0	0	0	0	0	1	198.4	124.3	203.1	24.6
1	0	1	0	0	1	27.7	124.5	262.7	21.8
0	0	0	1	0	1	472.0	339.2	549.6	30.2
0	1	1	0	0	1	28.2	22.1	44.0	27.3
0	0	1	0	0	1	22.3	42.7	184.7	27.6
0	0	1	0	0	1	9.9	13.1	8.0	31.7
0	0	0	0	0	1	23.5	25.4	61.1	33.9
0	1	1	0	0	1	443.8	658.0	405.8	31.4
0	0	0	1	0	1	16.6	22.9	35.6	29.0

0	0	0	1	1	1	19.2	19.1	35.5	32.2
0	0	1	1	0	1	43.8	71.9	19.9	29.4
0	0	1	0	1	1	152.3	234.7	286.7	29.1
0	0	1	0	0	1	7.5	16.8	17.5	26.3
0	0	0	0	0	1	30.7	63.8	23.2	28.4
0	0	0	0	0	2	19.1	33.9	52.1	28.2
0	0	1	0	0	1	17.7	23.4	29.1	32.8
0	0	1	0	0	1	24.8	44.7	26.2	18.7
1	0	1	0	1	1	215.6	192.1	43.1	20.5
1	0	1	0	0	1	17.9	61.2	40.7	24.1
0	0	0	0	1	1	47.4	52.1	17.7	39.0
0	0	1	0	0	1	209.7	369.8	602.3	34.5
0	0	1	0	0	1	11.3	25.2	74.0	34.2
1	0	1	0	0	2	15.8	21.9	26.0	22.6
1	0	1	1	0	1	120.1	219.8	360.4	27.5
0	0	0	0	0	1	44.5	63.1	87.3	32.0
0	0	1	0	0	1	134.7	117.0	663.0	31.8
1	0	1	0	0	2	16.6	16.7	31.7	28.7
0	0	1	1	0	1	76.8	125.3	172.2	24.3
0	0	0	0	0	2	41.9	61.3	36.1	19.8
0	0	1	0	0	1	911.2	738.0	257.1	33.0
0	0	1	1	0	1	282.9	176.1	576.6	34.0
0	0	1	0	0	1	135.2	147.6	735.8	32.9
0	0	1	0	0	1	21.6	46.1	58.8	23.9
0	0	1	1	0	1	486.0	633.2	294.9	30.2
0	0	1	0	0	1	26.3	37.3	70.0	28.3
0	0	1	0	0	2	48.0	44.7	21.0	35.1
1	0	1	0	0	1	1793.9	1121.7	475.5	32.5
0	0	1	1	0	1	58.1	185.6	622.5	28.5
0	0	1	0	0	2	26.4	87.8	124.1	21.2
1	0	1	0	0	2	20.7	53.4	56.4	20.2
1	0	1	0	0	1	18.7	91.3	46.1	26.4
1	1	1	0	0	2	101.1	121.7	102.5	25.9
1	1	1	0	0	1	26.1	30.8	56.8	29.5
0	0	1	0	0	1	25.8	36.3	49.5	23.4
1	0	1	0	0	1	37.9	116.6	205.0	17.0
1	0	0	0	0	2	138.1	299.4	253.8	25.4
1	0	1	0	1	2	27.0	31.2	24.7	28.3
0	0	0	0	0	1	31.5	41.5	33.5	28.0
0	0	1	0	0	1	26.3	53.7	130.2	25.1
1	0	1	0	1	1	16.5	23.5	51.0	25.4
0	0	0	0	1	1	30.1	126.5	55.2	26.6
0	0	1	0	0	1	2310.3	1482.4	338.5	35.3
0	1	1	1	1	1	10.5	34.1	103.8	23.5
0	0	0	0	1	1	52.8	98.8	145.9	20.2

0	0	1	0	0	2	25.5	80.5	827.3	33.5
1	1	1	1	0	1	629.2	630.0	258.5	24.1
0	0	1	0	0	1	39.5	77.3	53.2	22.5
0	0	1	0	0	1	41.7	84.7	58.9	22.0
0	0	1	0	0	2	35.3	75.9	80.2	17.8
0	0	1	0	0	1	47.1	130.6	16.2	23.1
1	0	1	0	1	1	379.1	179.4	400.6	26.8
0	0	1	1	0	1	11.6	24.7	43.7	25.9
0	0	1	0	1	1	151.8	259.5	490.0	30.5
1	0	1	0	0	1	32.4	57.2	650.8	26.2
0	0	0	0	0	1	817.5	373.1	113.7	40.3
0	0	1	1	0	1	22.8	75.6	40.7	25.1
0	0	1	0	1	1	71.6	54.7	95.4	34.7
0	0	1	0	1	1	10.6	21.4	11.8	24.2
0	0	1	0	0	1	11.0	18.8	22.1	22.0
0	0	0	0	1	1	24.3	21.8	42.7	32.6
1	0	1	1	1	1	46.9	94.1	32.1	29.5
1	0	1	1	0	2	37.2	58.1	8.7	30.5
0	0	1	0	1	1	24.4	141.3	19.3	27.6
0	0	1	0	0	1	17.8	56.2	116.7	20.4
1	0	1	1	0	1	11.9	26.7	14.4	32.2
0	0	1	0	1	1	56.2	102.2	611.3	31.3
1	0	1	0	0	2	15.8	25.2	29.3	29.8
1	0	1	0	1	2	15.4	40.3	10.8	18.6
0	0	0	0	1	1	14.9	19.0	20.4	34.2
0	0	0	0	0	2	64.8	66.9	47.0	25.2
0	0	1	0	1	2	39.4	47.9	88.9	29.9
0	0	0	0	1	1	54.2	77.9	51.4	27.2
0	0	1	0	0	1	64.1	143.3	227.6	22.2
0	0	1	0	0	1	15.5	26.9	11.7	22.0
1	0	1	0	0	1	35.7	82.6	442.1	29.1
0	0	1	0	0	1	390.0	377.3	471.5	35.7
1	0	1	0	0	1	31.5	29.3	37.1	28.1
0	0	1	1	1	1	103.1	85.2	109.1	29.1
0	0	1	0	0	1	42.7	53.2	57.2	27.3
0	0	1	0	0	1	370.8	618.1	374.6	31.0
0	0	0	0	0	1	22.8	26.1	34.1	32.9
0	0	0	0	0	2	28.5	56.0	21.6	26.5
0	0	0	1	0	1	18.0	32.2	34.5	27.6
0	0	1	0	0	1	77.9	101.9	687.4	36.7
1	0	1	0	0	1	761.1	1607.0	30.0	29.8
0	0	1	0	0	1	522.8	217.4	329.2	24.0
0	1	1	0	0	1	50.9	180.9	113.6	31.8
1	1	1	0	0	2	55.2	323.8	115.3	29.2
1	0	1	0	0	1	15.6	22.1	22.8	28.0

0	0	1	0	1	1	31.3	82.1	138.1	25.6
0	0	0	0	0	1	34.2	55.7	16.4	20.5
0	0	1	0	0	1	67.2	94.4	155.3	26.4
1	0	1	0	0	1	47.9	104.4	46.0	28.0
0	0	1	0	0	1	99.3	203.3	449.6	29.2
0	0	1	0	0	1	7.4	18.8	54.5	28.4
0	0	1	0	0	1	128.9	143.4	552.2	26.1
0	0	1	0	0	1	22.5	36.4	105.1	29.1
1	0	1	0	0	1	26.1	31.0	13.9	27.7
0	0	1	0	0	1	1193.0	1861.4	385.1	26.1
0	0	1	0	0	1	47.4	112.5	361.3	31.6
0	0	0	1	1	1	17.8	31.9	9.2	28.9
1	0	1	0	0	1	25.9	53.3	44.0	29.7
1	0	1	0	0	2	41.6	45.7	52.4	23.9
0	0	1	1	0	1	131.6	134.1	146.1	31.1
0	0	1	1	0	1	98.5	85.3	84.0	30.7
0	0	0	1	1	1	5.8	15.4	2.8	24.7
1	0	0	0	1	1	53.8	72.6	23.9	25.4
1	0	1	0	0	1	39.8	44.5	33.3	23.8
0	0	1	0	0	1	34.4	53.6	37.1	21.7
1	0	1	0	1	2	19.1	20.3	13.6	30.6
0	0	0	1	1	2	16.6	27.2	24.2	28.8
1	1	1	0	0	1	149.7	141.6	104.2	25.4
0	0	1	0	0	1	91.8	94.9	207.4	34.7
0	0	1	1	1	1	84.7	134.3	444.3	28.0
0	0	1	1	1	2	5.7	13.5	25.8	28.5
0	0	1	0	0	1	31.1	52.7	28.0	35.0
0	0	0	0	0	1	55.8	80.4	526.6	27.9
0	0	1	0	0	1	34.2	36.6	8.3	34.6
0	0	0	0	0	2	429.8	565.9	340.4	25.4
0	0	0	1	1	1	31.0	39.3	33.5	27.7
0	0	1	1	1	2	8.4	16.5	12.9	29.0
1	0	1	0	0	1	142.7	152.8	278.1	23.9
0	0	1	0	1	1	9.7	18.0	16.5	31.1
0	0	1	0	0	1	50.2	91.1	133.0	29.5
1	0	1	0	0	2	91.4	46.4	18.9	20.5
1	0	1	0	0	1	1638.3	1520.7	362.8	30.9
0	0	1	0	0	1	125.7	796.7	180.2	20.8
1	0	1	0	0	2	28.3	65.6	34.8	21.4
0	1	1	0	0	1	320.7	171.2	513.2	31.4
1	0	1	0	1	1	45.4	221.7	243.5	27.0
0	0	1	0	0	1	27.6	41.8	157.9	32.5
0	0	1	0	0	1	13.9	37.6	206.8	25.7
1	0	1	0	0	1	1223.5	1203.1	44.4	28.4
0	0	1	0	0	1	16.2	61.9	95.9	26.1

0	0	1	0	0	1	166.6	180.4	833.6	35.5
1	0	1	0	1	1	20.4	34.5	17.8	25.7
0	0	1	0	1	2	83.0	103.6	93.5	20.2
0	0	1	1	0	2	30.4	43.6	49.0	34.2
0	0	1	0	0	2	20.1	44.7	170.7	22.8
1	0	1	1	1	2	55.1	53.0	42.9	19.5
0	0	0	1	1	1	2127.8	2335.7	397.2	28.1
0	0	1	0	0	1	49.6	73.6	183.4	26.8
1	0	1	0	1	1	828.6	768.2	179.6	31.5
0	0	1	0	0	1	25.8	53.4	73.6	24.6
0	0	1	1	1	1	58.4	91.3	85.5	40.4
1	0	0	0	0	2	231.0	1153.0	82.2	19.9
1	0	0	0	0	1	4.5	19.2	12.8	25.5
0	0	0	0	0	1	48.9	70.1	33.5	28.3
1	1	1	0	0	1	23.4	39.4	99.5	20.1
0	0	0	0	1	2	25.6	33.5	130.4	28.6
0	0	1	0	0	1	1081.0	1729.1	313.2	25.6
0	0	0	0	0	1	381.2	301.3	397.7	33.9
1	0	1	1	0	2	19.5	23.6	73.2	36.9
0	0	1	0	0	1	67.4	111.6	46.7	23.2
0	0	1	0	0	1	41.9	227.8	118.0	16.8
0	0	1	0	0	1	34.1	59.4	45.1	31.4
0	0	1	0	1	1	5.3	30.5	47.5	28.8
0	0	1	1	0	1	231.7	288.5	401.1	33.4
0	0	1	1	1	2	8.6	22.4	34.7	19.2
0	1	1	1	0	1	74.1	96.8	219.4	21.9
0	0	0	0	1	1	274.2	338.4	97.4	30.6
1	1	1	0	0	1	27.3	41.6	32.1	24.6
0	0	1	0	0	1	8.4	21.5	14.9	25.5
0	0	1	0	0	1	100.7	112.1	404.9	27.5
0	0	1	0	1	1	32.6	37.6	85.8	26.0
0	0	1	1	0	1	17.2	27.6	15.8	29.2
1	0	1	1	0	2	596.7	592.9	51.4	26.4
1	0	1	0	0	2	128.8	222.9	138.7	28.4
0	0	1	0	0	1	4.8	8.4	2.9	19.7
1	0	1	1	0	1	1053.6	335.2	231.9	26.8
0	0	0	0	1	2	26.2	35.3	31.4	22.1
0	0	0	0	0	2	34.9	62.5	43.4	30.5
0	0	1	0	0	2	16.5	34.0	51.4	20.6
0	0	1	0	0	1	41.2	76.8	509.8	31.4
0	0	1	1	1	1	28.2	27.9	106.7	28.6
0	1	1	0	0	1	17.8	61.1	244.0	28.2
1	0	0	0	0	1	23.1	45.7	33.4	28.4
0	0	1	1	0	1	852.9	617.6	221.5	28.0
1	0	1	0	0	1	140.0	261.7	490.1	33.4

1	0	1	0	0	2	29.7	57.4	199.7	20.3
0	0	1	0	1	1	40.1	39.0	28.0	29.2
0	0	1	0	0	1	30.7	151.2	358.2	24.2
1	0	1	0	1	1	19.5	29.7	19.6	21.7
0	0	0	1	0	2	20.0	56.5	83.3	24.0
1	0	1	0	0	2	63.2	104.1	17.8	27.3
1	0	1	0	1	1	13.5	31.5	6.8	25.6
1	0	1	0	0	1	27.2	47.5	46.0	25.8
0	0	1	0	0	1	53.2	65.0	105.1	26.9
1	0	1	0	0	1	40.7	204.1	342.9	23.6
0	0	0	0	0	1	19.7	38.6	68.2	30.2
0	0	1	0	0	1	17.2	47.8	146.9	19.6
1	0	1	0	0	2	5.4	16.3	136.1	17.0
0	0	1	0	0	2	114.1	136.0	365.4	33.4
0	0	1	1	0	1	21.2	36.8	87.5	22.9
0	0	0	1	0	1	1758.1	1126.8	424.3	31.3
0	0	1	0	1	1	12.7	56.1	412.4	31.8
0	0	1	1	1	1	1232.5	873.6	334.4	32.2
0	0	1	1	0	1	28.8	68.4	48.2	21.8
0	0	1	0	0	1	1290.6	1937.3	327.4	28.2
1	0	1	1	0	1	21.5	36.0	10.1	24.4
0	0	1	0	0	2	19.2	36.7	230.3	20.5
0	0	1	1	0	1	57.8	146.2	456.6	24.0
0	0	1	1	0	1	119.5	179.3	89.3	36.6
0	0	0	1	1	1	17.9	33.8	62.3	31.4
0	0	0	0	0	1	431.7	616.3	202.2	32.8
0	0	1	0	0	1	20.9	81.5	120.9	28.9
0	0	0	0	0	1	28.2	100.8	181.4	26.8
0	0	1	0	0	1	43.0	38.5	32.1	33.6
0	0	1	1	1	1	653.7	252.0	101.6	30.6
0	1	1	0	0	2	27.7	37.9	45.2	22.9
0	0	1	1	0	1	29.2	131.2	100.7	26.2
0	0	1	0	0	1	17.7	30.2	113.2	30.2
0	0	0	0	0	1	25.5	51.0	249.3	38.1
0	0	1	0	0	1	101.7	115.1	65.7	23.8
1	0	1	0	0	1	108.2	233.5	555.9	22.0
1	0	0	0	0	1	29.8	45.8	36.4	21.2
1	0	1	0	0	1	27.9	38.8	36.8	26.8
0	0	1	0	0	2	130.2	99.3	180.2	30.4
0	0	1	0	0	1	23.6	39.3	76.2	21.2
0	0	1	0	0	1	15.0	27.6	49.2	30.0
1	1	1	0	0	1	11.7	16.2	15.8	33.0
0	0	0	0	1	1	16.7	20.7	101.8	26.7
0	0	1	1	1	1	8.6	21.3	27.6	19.6
1	0	0	0	0	1	7.8	14.3	17.2	29.1

0	0	0	1	0	1	10.8	24.6	30.5	29.4
0	0	1	0	1	1	686.3	588.6	250.9	36.1
0	0	1	0	1	2	37.2	67.5	213.7	21.7
0	0	1	0	0	1	31.4	90.5	56.5	27.0
1	0	1	0	1	1	12.3	12.0	21.2	24.6
1	0	1	0	0	2	57.0	175.8	479.5	24.0
0	0	1	0	0	1	49.0	28.1	126.5	27.3
1	0	1	0	0	1	32.1	35.2	36.3	21.0
1	0	1	1	1	2	362.9	396.9	100.3	30.1
0	0	1	1	0	1	67.1	76.5	644.9	34.8
1	0	1	1	0	1	36.1	112.0	124.2	28.2
0	0	1	0	0	1	23.4	38.7	144.4	29.6
1	0	0	0	0	1	27.4	39.0	11.5	28.8
1	0	1	0	0	1	18.0	88.6	130.8	32.8
0	0	1	0	1	1	43.4	118.2	44.0	24.9
0	0	1	0	0	1	25.4	74.4	93.0	27.6
0	0	1	0	0	1	424.0	772.3	193.4	40.0
0	0	0	0	0	2	23.0	37.0	44.3	38.4
1	0	0	1	1	1	14.2	16.5	13.2	26.8
0	0	1	0	0	1	14.5	41.9	321.2	27.5
0	0	1	1	1	1	27.2	38.5	28.6	30.7
0	0	0	0	1	1	56.8	51.7	99.9	36.6
1	0	1	1	1	1	1694.7	1195.3	281.4	30.2
0	0	1	0	0	2	26.0	70.4	560.7	25.7
0	0	0	0	0	2	32.6	60.4	162.6	32.3
1	0	1	0	0	2	27.4	26.2	12.5	30.5
1	0	1	0	0	1	11.7	17.0	18.9	31.2
0	0	1	0	0	2	87.8	62.0	164.6	37.0
1	1	1	1	1	2	32.1	46.0	140.6	25.6
0	0	0	1	0	1	22.7	64.8	58.1	23.4
0	0	1	0	0	1	18.8	96.5	127.5	19.8
0	0	0	0	0	1	1409.7	3324.2	65.2	34.1
0	0	1	1	1	1	14.8	42.7	44.0	26.2
0	0	1	1	0	1	58.1	91.0	33.8	38.2
0	0	1	0	0	2	228.4	426.8	196.9	23.2
1	0	0	0	0	1	23.7	38.3	87.8	26.7
1	0	1	0	1	2	29.0	78.7	24.0	16.6
1	0	1	1	1	1	9.3	45.4	51.1	28.1
1	0	1	0	1	1	21.0	20.1	17.4	35.0
1	0	0	0	0	2	191.4	200.4	18.9	26.6
1	0	1	0	1	1	8.6	17.0	72.8	23.9
1	0	1	0	0	1	349.4	156.9	18.3	21.6
0	0	0	0	1	1	16.8	24.7	27.1	32.9
0	0	1	0	0	1	10.5	31.5	46.4	28.5
0	0	1	0	0	1	18.4	40.7	29.1	24.9

0	0	1	1	0	2	31.9	74.4	609.9	38.9
0	0	1	0	0	2	97.0	249.2	17.5	23.8
0	0	1	1	0	1	62.1	91.2	119.4	29.7
0	0	0	1	0	1	15.9	32.3	15.4	33.1
0	0	1	0	0	1	11.3	51.5	153.0	35.6
0	0	1	0	0	1	109.9	131.3	119.1	31.5
0	0	0	0	0	2	39.6	40.6	94.4	33.3
0	0	1	0	0	1	45.9	68.2	174.5	30.1
0	0	1	0	0	1	15.7	23.8	9.3	32.5
0	0	0	0	0	1	36.4	94.4	329.0	22.8
0	0	0	1	0	1	10.9	21.5	17.7	27.4
0	0	1	0	0	1	96.2	169.8	522.8	27.4
0	1	1	0	0	1	19.5	63.1	117.7	20.5
1	1	1	0	1	1	20.9	38.8	54.3	24.4
1	0	1	0	0	1	243.0	277.6	19.0	22.6
0	0	0	0	0	2	7.2	16.0	59.8	37.8
0	0	1	1	0	1	10.9	27.6	23.9	36.6
0	0	1	0	0	1	59.0	369.9	747.0	27.0
0	1	1	1	0	1	58.6	65.9	93.1	32.3
0	1	1	0	0	1	25.4	45.3	44.3	39.3
1	0	0	0	0	2	247.8	113.2	39.3	32.3
1	0	1	0	0	1	24.3	24.9	16.8	25.2
0	0	1	0	0	1	76.1	95.9	90.4	24.3
1	0	0	0	0	1	20.1	47.2	100.4	26.4
0	0	0	0	0	1	1705.6	1205.7	204.4	27.2
0	0	1	0	0	1	46.1	52.4	343.0	32.1
1	0	1	0	0	1	37.5	56.1	10.2	26.6
1	0	1	0	0	1	16.1	29.9	39.2	26.3
0	0	1	0	0	1	12.7	13.4	32.0	31.0
0	1	1	0	1	1	31.1	37.0	11.0	29.5
0	0	1	0	1	1	18.6	39.5	174.2	34.3
1	0	1	0	0	1	50.8	90.7	427.0	20.6
0	0	1	0	0	1	38.0	64.3	146.1	29.2
0	0	0	0	1	1	15.5	23.4	86.2	28.7
0	0	1	0	0	2	34.7	56.7	65.1	20.2
1	0	1	0	0	1	18.8	44.6	9.8	29.3
0	0	1	0	1	2	23.9	45.6	26.4	25.4
1	0	1	0	0	1	21.5	29.4	115.3	22.4
0	0	1	0	1	1	38.1	53.1	259.6	30.2
0	0	1	1	0	1	22.8	69.6	483.3	24.3
0	0	1	0	0	1	28.2	65.6	61.6	24.8
1	0	1	1	0	2	21.7	81.7	26.3	17.1
0	0	1	0	0	1	32.3	51.4	521.3	28.6
0	0	1	0	0	2	25.5	58.0	96.2	30.4
0	0	0	1	1	1	421.9	615.8	182.3	29.1

0	0	0	0	0	2	605.3	719.8	316.4	29.5
0	0	1	0	0	2	12.5	23.7	29.5	27.3
0	0	1	1	1	1	59.2	72.1	90.6	26.1
0	0	1	0	0	1	161.4	169.8	133.7	23.9
0	0	1	0	0	1	12.5	64.3	54.1	30.2
0	0	1	0	0	2	284.1	111.4	226.1	33.0
0	0	0	0	0	1	1751.8	1390.4	187.3	37.3
0	0	1	1	1	1	43.5	63.9	42.8	30.4
1	0	1	0	0	2	40.5	111.6	105.7	24.5
0	0	1	0	0	2	14.2	37.8	69.3	37.0
0	0	1	1	0	1	17.5	57.7	66.9	25.2
0	0	0	1	0	1	475.7	402.7	153.2	33.9
0	0	1	0	0	2	58.1	61.1	136.7	21.1
0	0	1	0	0	2	26.0	59.5	143.3	21.7
0	1	1	1	0	1	44.6	135.8	355.9	30.4
0	0	1	0	0	1	142.6	129.5	290.1	29.5
1	0	1	0	0	1	19.0	27.3	13.5	31.0
0	0	1	0	0	1	42.8	56.7	19.8	26.1
0	0	0	0	1	1	31.5	36.2	209.9	27.7
0	0	1	1	1	1	187.2	330.7	325.4	31.1
1	0	1	1	0	2	57.0	108.6	64.4	27.2
0	0	1	0	0	1	377.0	154.7	388.4	31.5
0	1	1	0	1	1	17.9	30.6	50.9	24.6
0	0	0	0	0	1	29.7	47.2	230.8	34.1
0	1	1	0	0	1	32.3	47.7	130.4	28.4
1	1	1	1	0	2	497.6	362.3	320.1	28.3
1	0	0	1	1	2	21.9	53.6	23.2	30.2
1	0	1	0	0	2	9.7	36.7	77.5	28.8
0	0	1	1	0	1	41.1	30.7	24.2	28.7
0	1	1	0	1	2	36.5	46.9	161.3	24.1
0	0	1	0	0	2	36.9	70.4	29.1	28.1
1	0	0	0	0	1	69.2	68.8	7.0	29.8
0	0	1	0	0	1	132.2	148.6	497.9	32.4
1	0	1	1	1	1	20.5	82.4	200.4	28.1
1	0	1	0	0	1	529.7	1265.7	81.6	21.2
0	1	1	0	0	1	19.1	33.2	26.8	32.2
1	0	1	0	0	1	88.1	117.8	615.6	30.5
0	0	0	0	1	1	45.7	47.5	39.7	32.6
1	0	1	0	0	1	78.0	98.8	176.4	29.9
0	0	1	0	0	1	15.9	42.9	136.6	31.9
0	0	1	0	0	2	16.9	32.9	44.7	30.9
1	1	1	0	0	2	45.0	114.0	541.0	24.8
0	0	1	0	0	1	670.6	1056.6	470.8	24.1
1	1	1	0	0	1	54.2	74.7	129.2	35.8
0	0	1	1	1	1	27.8	40.8	212.5	27.1

1	0	1	0	0	1	28.9	185.8	269.2	27.8
0	0	0	0	0	1	49.6	118.3	548.2	36.5
1	1	1	0	0	1	32.4	96.0	65.5	19.9
0	0	1	0	0	1	32.6	85.3	40.3	26.4
1	1	1	0	0	1	194.6	247.6	267.6	20.9
0	0	0	1	1	1	20.6	28.0	25.4	40.4
1	1	1	1	1	1	26.9	43.6	37.6	28.4
0	0	1	0	0	1	39.2	92.5	154.1	21.1
0	0	1	0	0	1	25.4	54.9	273.4	29.5
1	0	1	1	0	1	50.8	85.1	81.9	26.4
1	0	0	1	1	1	9.0	14.4	33.3	26.3
0	0	1	0	0	1	42.2	100.9	80.0	28.1
0	0	1	0	0	1	236.5	231.0	463.7	27.6
1	0	1	0	0	1	46.2	44.7	20.2	36.6
0	0	1	0	0	1	8.7	31.6	16.5	28.6
0	0	1	0	0	1	24.2	38.9	58.7	28.2
0	0	0	0	0	1	40.1	55.6	406.9	22.0
0	0	1	0	1	1	499.4	647.0	363.9	25.8
0	0	1	0	1	1	15.7	14.4	22.4	27.0
1	0	1	1	0	1	17.2	62.2	241.3	26.6
1	0	1	0	0	1	139.3	284.2	437.0	29.8
1	0	1	1	1	1	18.2	30.0	44.6	25.5
0	0	1	0	0	1	150.1	151.9	402.3	26.9
0	0	1	0	1	1	21.8	30.2	18.3	28.2
0	0	1	0	0	1	28.8	70.7	179.4	34.8
1	1	1	0	0	1	10.1	23.1	90.6	20.3
0	0	1	0	0	1	107.6	157.4	306.8	35.7
1	0	1	0	0	1	19.8	34.3	20.6	22.8
1	1	1	0	1	1	11.6	33.5	18.2	23.6
0	0	1	0	0	1	46.0	63.0	464.8	33.2
0	0	1	0	0	1	104.1	128.5	525.2	33.5
0	0	0	0	1	1	82.0	109.2	40.3	28.0
0	0	0	0	0	1	20.7	44.1	59.8	25.3
0	1	1	1	1	1	52.0	38.9	81.4	29.9
0	0	1	1	1	1	42.7	110.5	107.6	17.3
1	1	1	0	0	1	101.7	163.0	551.7	33.0
0	1	1	0	0	1	43.2	55.3	395.4	22.1
0	0	0	0	1	2	58.3	61.8	70.8	27.9
0	0	0	0	0	1	120.9	196.0	594.0	34.7
0	1	1	0	0	2	39.7	72.7	333.7	30.0
0	0	1	0	0	1	137.4	560.8	339.3	28.6
0	0	1	1	0	1	41.7	41.7	37.0	28.8
0	0	1	1	0	1	25.8	59.8	371.5	30.0
0	0	1	0	0	1	784.9	608.7	357.3	30.2
1	0	1	1	0	1	19.3	46.9	164.9	18.9

0	0	0	0	0	1	30.9	67.7	240.9	29.3
1	0	1	1	1	1	39.7	37.7	4.8	31.1
0	0	1	0	0	2	20.3	46.7	103.9	22.9
0	0	1	0	0	2	17.9	21.8	28.8	28.4
1	0	1	1	1	2	87.8	187.6	18.8	25.1
1	0	1	0	1	2	38.4	145.8	16.7	21.2
1	0	1	0	0	1	16.8	23.9	9.0	31.2
0	1	1	0	0	1	27.7	51.3	195.5	23.7
1	0	1	0	1	2	18.5	34.5	28.0	24.4
0	0	1	0	0	1	22.5	37.4	33.6	30.1
0	0	0	0	0	1	30.9	27.9	56.1	31.6
0	0	0	0	0	1	9.9	17.2	61.1	28.3
0	0	0	0	1	1	28.7	44.3	222.4	30.7
0	0	0	0	0	1	28.6	44.6	44.9	30.2
0	0	0	0	0	1	40.9	67.5	88.1	32.7
0	0	1	0	0	2	30.7	34.4	51.9	26.6
0	0	1	0	0	2	37.0	51.6	899.0	19.9
0	0	1	1	0	1	207.8	281.1	324.5	28.1
0	0	0	0	1	1	25.2	32.8	45.9	28.1
0	0	0	0	0	1	34.4	63.2	61.7	27.4
0	0	1	0	0	1	57.2	112.4	556.7	22.5
0	0	1	1	1	1	83.7	159.4	429.4	33.7
0	0	1	0	0	1	10.9	18.1	44.8	33.0
0	0	1	1	0	1	49.3	73.2	35.0	25.5
0	0	1	0	0	1	27.8	42.4	59.9	23.5
0	0	0	0	0	1	155.8	360.1	507.1	28.2
0	0	1	0	0	1	523.3	569.5	325.9	35.3
1	0	1	0	0	1	32.8	51.4	150.9	28.3
0	0	1	0	0	1	39.1	128.4	95.5	23.6
0	0	1	0	1	1	968.8	743.0	218.7	31.8
0	0	1	0	1	1	329.6	685.9	433.9	28.5
0	0	1	0	0	1	33.0	65.0	92.0	20.9
0	1	1	0	0	1	234.8	104.4	599.3	36.0
0	0	0	0	0	1	21.1	49.7	32.6	28.3
0	0	1	0	0	1	132.4	194.3	134.2	25.3
0	0	0	0	0	1	22.4	38.0	85.3	33.3
0	0	1	0	0	1	43.7	67.9	221.1	30.3
0	1	1	1	1	1	7.0	30.7	18.5	29.6
0	0	1	1	0	1	38.2	48.0	16.5	25.9
0	0	1	0	0	1	167.9	116.6	418.8	27.7
1	0	1	0	0	1	21.7	22.3	14.4	29.1
0	0	0	0	0	1	34.4	53.0	42.2	27.6
0	0	1	0	1	1	13.5	20.2	19.6	29.7
0	0	0	0	0	1	43.5	103.0	72.4	24.5
0	1	1	0	1	1	41.2	41.8	20.2	28.2

0	0	1	0	0	1	20.6	54.6	386.2	30.3
0	0	0	1	1	1	43.0	64.7	432.0	29.2
0	0	1	0	0	1	22.6	34.9	61.8	34.0
1	0	0	0	0	2	52.0	58.0	119.6	29.1
1	0	1	0	0	2	22.8	51.3	48.4	39.9
1	0	1	0	0	1	25.4	43.3	33.6	19.3
1	0	1	0	0	2	17.6	25.5	57.9	37.8
0	0	1	1	1	1	8.1	24.4	22.5	26.7
0	0	1	1	0	1	18.5	55.7	47.6	33.5
0	0	0	0	1	1	36.6	42.9	84.9	26.0
0	0	0	0	1	2	13.6	23.8	59.6	27.7
1	0	1	0	0	1	33.0	45.1	22.9	21.2
1	0	1	0	0	1	12.7	16.2	140.5	32.9
0	0	1	0	1	1	172.0	225.8	96.6	24.8
1	0	1	0	0	2	63.4	193.4	16.4	28.0
0	0	1	0	0	1	63.4	193.4	16.4	28.0
0	1	1	0	0	1	47.1	75.3	49.2	28.8
0	0	0	1	0	2	26.7	58.4	57.4	26.6
0	1	1	0	0	1	54.6	58.6	78.7	25.5
0	0	1	0	0	1	27.2	56.4	78.4	37.1
0	0	1	0	0	1	8.7	30.7	16.1	25.7
0	0	0	0	0	1	34.3	41.9	80.2	28.2
0	0	1	0	0	1	18.9	79.8	103.0	31.4
0	0	1	0	0	1	28.7	77.9	54.8	26.1
0	0	0	0	0	1	24.0	33.1	86.4	31.3
0	0	0	0	0	1	81.7	82.3	10.6	30.6
0	0	1	0	0	1	36.6	81.2	150.4	30.7
0	0	1	1	0	1	42.9	85.2	163.2	25.7
0	0	1	0	1	1	51.3	69.2	106.2	30.7
0	0	1	1	1	2	50.9	55.1	24.2	31.0
1	0	1	0	0	1	53.7	122.4	37.6	25.8
1	0	0	1	0	1	9.6	19.7	35.7	28.1
1	0	1	0	1	1	13.5	33.8	25.4	28.0
0	0	1	0	0	1	64.1	104.5	153.0	23.4
0	0	1	0	0	1	77.7	78.6	57.1	20.9
1	0	1	0	0	1	41.7	54.5	186.9	18.9
0	0	1	0	0	1	37.8	73.4	202.7	27.6
0	0	0	1	0	1	57.9	110.9	16.5	31.1
0	0	1	1	0	1	16.5	57.8	208.0	28.5
0	0	1	1	0	1	9.9	29.3	169.9	27.3
1	0	1	0	0	1	21.7	42.0	17.3	32.4
0	0	1	0	0	1	26.4	88.2	165.5	23.4
0	0	1	1	0	1	16.6	18.7	37.4	38.0
0	0	0	0	0	1	114.7	57.9	664.9	32.3
0	0	1	0	0	2	11.0	16.2	22.3	22.9

0	0	1	0	0	1	46.3	80.8	76.5	36.8
0	0	1	1	0	1	32.3	73.5	85.5	25.0
0	0	1	1	0	1	33.2	39.2	409.5	27.2
0	0	1	0	0	1	31.0	23.0	32.8	32.0
0	0	1	0	0	2	59.2	76.3	175.6	30.5
1	0	0	0	0	1	11.7	19.8	10.3	34.1
0	0	1	0	0	1	53.4	91.6	306.6	17.8
0	0	0	1	1	1	30.3	42.7	37.8	26.0
1	0	1	0	0	1	61.1	32.7	8.7	25.8
0	0	1	0	0	1	259.6	72.9	489.9	29.9
0	0	1	1	1	1	13.6	28.3	23.4	28.6
0	0	1	0	0	1	22.9	31.0	50.8	29.2
0	0	1	0	0	1	39.2	52.8	19.5	25.7
0	0	0	0	0	1	63.3	96.9	358.5	34.5
0	0	1	0	0	2	15.4	18.1	20.0	25.9
0	0	0	0	0	1	54.2	166.1	434.1	26.1
1	0	1	0	0	1	20.5	31.7	28.1	20.3
1	0	1	0	0	1	12.1	21.4	26.9	25.5
0	0	1	0	0	1	16.9	99.1	159.0	36.6
0	0	1	1	0	1	118.6	238.6	634.6	26.8
0	0	1	0	0	2	19.9	40.7	31.5	26.5
0	0	1	1	0	1	25.7	49.4	21.9	34.5
1	0	1	0	0	1	16.5	35.7	38.7	30.5
1	0	1	0	0	2	103.1	75.4	143.3	34.5
0	0	1	0	0	1	52.9	65.6	70.0	22.9
0	0	0	0	0	1	18.1	21.0	26.4	31.2
1	1	1	0	0	1	16.6	24.2	213.1	23.2
0	1	1	1	0	1	32.7	37.3	26.9	25.3
0	1	1	0	0	1	184.8	227.7	298.4	24.4
1	0	0	0	1	1	120.8	107.9	68.0	25.7
0	0	0	1	0	1	397.8	664.5	121.7	22.0
0	0	0	0	1	1	29.1	74.3	31.4	31.1
0	0	1	0	0	1	14.3	16.1	118.3	33.3
0	0	0	1	0	1	79.4	76.3	30.4	34.4
0	0	1	0	0	1	34.5	39.1	64.2	30.1
1	0	1	0	0	1	111.7	164.6	21.4	22.0
0	0	0	1	1	1	10.8	44.2	92.8	29.8
0	0	0	0	0	1	906.3	311.0	198.9	38.5
0	0	1	0	1	1	35.5	64.1	206.5	25.9
0	0	1	0	0	1	31.3	51.2	70.8	23.5
0	0	0	0	0	1	26.2	32.4	44.7	34.8
0	0	0	0	0	1	86.4	320.8	24.6	40.9
1	0	0	0	0	1	21.6	27.5	13.8	34.4
0	0	1	0	0	1	22.2	64.3	53.6	23.5
1	0	1	0	0	2	29.2	39.2	128.5	27.0

0	0	1	0	0	1	36.5	38.1	67.6	32.8
0	0	0	1	1	1	18.9	44.7	46.9	28.3
0	0	1	0	0	1	31.6	36.1	21.8	28.6
0	0	1	0	1	1	24.5	40.9	41.8	25.0
0	0	1	0	0	1	31.0	43.0	43.8	27.8
0	0	1	0	1	2	73.4	85.2	17.2	33.5
0	0	1	0	0	1	36.3	62.7	38.3	22.0
0	0	0	1	0	1	20.1	51.4	25.0	37.0
1	0	0	1	0	1	13.7	26.9	33.1	32.1
0	0	1	0	0	1	147.3	92.3	367.9	28.1
1	0	0	0	0	1	18.4	28.0	10.5	24.3
0	0	1	0	0	1	13.3	18.9	11.1	30.8
0	0	1	0	1	1	295.5	206.0	32.1	25.4
0	0	1	0	1	1	16.3	22.6	52.4	24.2
0	0	1	0	0	1	22.5	110.2	61.2	28.6
1	0	1	0	0	1	22.4	38.9	30.3	30.4
1	0	0	0	0	1	82.1	41.0	53.0	28.5
1	0	0	0	0	1	10.9	16.8	10.3	34.7
1	0	0	0	0	1	9.0	8.5	9.8	29.1
0	0	1	0	0	1	26.9	35.6	281.2	26.4
0	0	0	1	0	1	16.0	44.5	35.5	29.3
0	0	0	0	0	1	22.8	53.2	93.6	21.9
1	0	0	0	0	1	32.5	72.7	87.8	32.2
0	0	1	0	0	1	12.2	32.8	72.8	29.7
1	0	1	0	0	2	15.7	15.8	21.9	22.2
1	0	1	0	0	1	20.6	21.4	11.1	30.8
0	0	0	0	0	2	22.9	77.5	38.9	21.9
0	0	1	0	1	1	3.0	11.3	9.3	28.6
0	0	1	0	1	1	913.1	1457.4	135.7	29.2
0	0	1	0	0	1	9.0	17.4	42.1	35.1
1	0	1	0	0	1	22.9	22.3	11.7	30.5
0	0	1	0	1	2	27.5	97.0	76.9	29.5
0	0	0	0	0	1	69.2	72.4	25.6	24.7
1	0	1	0	1	1	38.2	31.5	7.0	34.7
0	0	1	0	0	2	55.9	119.7	105.6	32.4
0	0	1	0	0	1	17.8	34.5	55.5	27.2
0	0	1	0	0	1	41.8	62.1	55.3	31.7
1	0	0	0	0	1	11.3	1724.0	11.1	20.8
0	0	1	0	1	1	14.8	30.4	71.9	29.9
0	0	1	0	1	1	31.2	49.3	83.9	23.2
0	0	0	0	0	1	91.5	150.7	172.5	46.0
1	0	0	0	0	2	14.4	17.6	9.3	33.4
1	0	1	0	0	1	17.5	30.3	24.8	24.9
1	0	1	1	1	2	21.1	23.6	6.4	26.2
0	0	1	0	0	1	19.8	40.6	64.4	24.9

0	0	1	1	0	1	36.1	66.3	106.1	27.9
0	0	1	0	0	2	28.2	47.2	34.2	30.1
0	0	1	0	1	1	15.3	23.4	7.9	19.1
0	0	1	0	1	2	31.1	41.4	73.5	31.5
0	0	1	0	1	1	35.5	52.8	47.6	30.3
1	0	1	0	0	1	16.2	14.7	8.9	30.8
0	0	1	1	1	2	30.2	71.3	74.5	26.1
0	0	1	1	1	1	13.5	21.7	33.2	30.6
1	0	0	0	1	1	46.4	42.5	17.7	33.1
0	0	0	1	0	1	51.4	94.6	90.9	31.7
0	0	1	1	0	1	196.5	280.6	7.2	26.7
0	0	1	0	0	1	23.0	26.8	79.8	31.1
1	0	1	1	1	1	48.4	99.9	209.4	25.0
0	0	1	1	0	1	10.1	18.2	25.6	25.5
0	0	0	0	1	1	29.7	69.3	405.9	32.9
1	0	1	0	0	1	10.6	16.9	12.8	31.6
0	0	1	0	0	1	8.8	20.2	23.8	22.0
0	0	0	0	0	1	25.4	40.3	110.9	35.1
1	0	1	0	0	1	23.8	33.2	41.3	22.5
0	0	1	1	1	2	13.7	49.2	23.0	25.3
1	0	1	0	0	1	12.0	34.8	20.7	34.6
1	0	1	0	0	1	12.6	12.8	19.4	26.6
0	0	1	1	0	1	138.7	192.1	186.0	30.1
0	0	1	0	0	1	77.5	121.9	49.9	21.7
0	0	0	0	1	1	32.1	48.9	61.6	23.9
1	0	1	1	0	1	49.8	64.2	15.8	32.2
0	0	1	0	0	1	29.6	37.1	30.0	28.9
0	0	1	0	0	2	7.8	35.2	61.9	26.4
1	0	1	1	0	1	31.1	41.9	28.5	29.5
0	0	1	0	0	1	16.0	38.5	26.9	29.6
0	0	0	0	0	1	36.2	64.1	105.5	25.5
1	0	1	0	0	1	15.0	10.2	8.4	35.9
0	0	0	0	1	1	34.8	59.8	87.6	27.0
0	0	1	0	1	1	17.8	21.1	21.4	31.9
0	0	1	0	0	1	34.3	58.1	54.9	31.3
0	0	1	0	0	1	83.8	341.2	211.5	24.5
0	0	0	0	1	1	31.4	40.6	89.6	30.1
0	0	1	0	0	2	16.5	41.5	74.8	25.1
1	0	1	1	0	1	48.2	62.4	104.6	31.4
1	0	1	0	1	1	7.8	9.7	13.9	29.2
0	0	1	0	0	2	42.9	59.3	80.5	23.2
1	0	0	0	0	1	22.2	19.9	25.6	40.2
0	0	1	0	0	1	55.4	72.6	44.4	25.2
0	0	1	0	0	1	174.8	365.5	17.4	28.2
1	0	1	0	0	1	13.9	27.4	24.3	26.0

1	0	0	0	0	1	30.4	31.3	59.6	26.6
1	0	1	0	0	1	85.8	90.4	24.9	23.5
0	0	1	0	0	1	553.0	252.1	432.9	28.8
1	0	1	0	1	2	13.9	20.4	22.1	20.2
1	1	1	0	0	1	26.4	36.7	87.7	21.2
0	0	1	0	0	1	18.7	48.2	115.4	32.0
0	0	1	0	0	1	46.4	155.2	310.9	28.2
1	0	0	0	0	2	11.7	14.5	39.8	34.2
1	0	1	1	0	1	54.4	153.1	41.0	21.3
0	0	0	0	0	1	46.5	63.6	92.8	34.8
1	0	1	0	1	1	53.9	75.5	17.4	30.6
0	0	1	0	0	1	22.1	37.1	31.7	24.8
1	0	1	0	1	1	140.5	139.0	7.2	29.7
0	0	1	0	1	1	56.3	83.8	72.3	21.3
1	0	1	0	0	2	10.2	15.3	5.6	35.7
0	0	0	0	0	1	11.2	29.5	91.4	35.3
0	0	0	1	0	1	19.9	39.0	41.3	29.2
0	0	0	0	0	1	2013.5	2787.0	97.3	33.6
0	0	1	0	0	1	48.5	74.4	55.3	18.9
0	0	1	0	0	1	39.1	39.3	42.3	29.7
0	0	1	0	1	1	18.8	30.4	37.6	27.3
0	0	0	0	0	1	19.7	35.3	34.0	27.8
0	0	1	0	0	1	49.3	103.5	38.2	32.3
1	0	0	0	0	1	33.7	25.2	18.9	41.1
0	0	1	0	0	2	16.3	31.5	60.8	32.3
0	0	1	0	1	1	15.6	30.7	180.9	27.3
0	0	1	0	0	1	22.8	37.7	8.1	24.0
0	0	1	1	1	1	25.4	38.7	61.4	30.3
1	0	1	0	0	2	17.3	32.8	17.6	28.4
0	0	0	1	0	1	21.2	39.9	47.5	32.4
1	0	1	0	1	2	25.1	32.5	63.8	32.5
1	0	1	0	0	2	25.8	40.7	26.4	23.0
0	0	0	0	1	1	25.9	27.9	17.1	27.2
1	0	1	0	0	1	6.2	21.8	6.6	29.8
1	0	0	0	0	1	110.0	144.0	18.4	22.6
0	0	1	0	1	1	34.0	40.8	29.0	32.2
0	0	1	0	0	1	76.0	80.5	31.4	29.9
0	0	1	0	0	1	21.4	20.9	6.5	23.0
0	0	0	0	0	1	35.1	46.1	70.6	27.3
0	0	1	0	1	2	16.6	20.7	33.8	27.9
0	0	1	0	0	1	22.4	45.1	44.7	34.0
1	0	0	0	0	2	46.3	63.7	29.0	30.6
1	0	1	0	1	1	22.2	34.0	22.0	36.2
0	0	1	1	1	1	10.4	32.8	38.2	29.4
1	0	0	1	1	2	17.9	16.9	9.6	29.6

0	1	1	0	0	1	54.8	236.9	187.0	26.6
0	0	1	0	1	1	50.7	44.3	185.1	32.5
0	0	0	0	0	1	18.7	38.2	42.5	23.6
1	0	0	0	1	1	7.5	12.2	7.9	32.6
0	0	1	0	0	1	60.2	104.2	60.4	24.4
0	0	1	1	1	1	14.0	20.2	21.0	36.6
0	0	0	0	0	1	32.3	81.4	245.2	35.2
0	0	0	0	0	1	38.0	45.9	39.2	30.9
0	0	1	1	0	1	91.7	120.9	65.8	24.8
1	0	1	0	0	1	20.3	29.3	68.5	25.0
0	0	1	0	0	1	34.3	42.8	110.0	31.8
0	0	1	0	0	1	112.1	165.6	373.2	27.8
0	0	0	1	1	1	34.0	24.2	45.5	34.1
0	0	0	0	0	1	33.7	26.0	21.2	29.0
0	0	1	0	0	1	105.4	162.6	46.0	25.1
0	0	1	0	1	1	39.4	53.4	27.9	29.9
0	0	1	0	0	1	469.3	177.8	367.7	30.6
0	0	1	0	0	2	44.9	51.4	18.9	26.2
0	0	1	0	0	2	25.4	33.4	87.4	20.6
0	0	1	1	1	1	9.3	22.9	35.0	29.3
0	0	1	0	0	1	41.7	31.8	52.2	25.9
0	0	1	0	0	1	48.4	57.2	164.6	25.1
1	0	1	0	0	1	11.5	47.0	67.1	19.8
0	0	0	0	0	1	48.6	94.3	40.8	23.8
0	0	1	1	0	1	6.6	40.5	24.2	36.2
1	0	1	0	0	1	9.7	9.6	23.3	32.6
0	0	1	1	0	1	539.1	445.8	280.1	33.6
0	0	1	0	0	1	29.8	38.5	35.8	26.0
1	0	0	0	0	1	28.7	77.8	69.1	37.1
0	0	1	0	1	1	50.8	65.3	44.2	25.8
0	0	0	0	0	2	24.4	86.9	142.5	21.1
1	0	1	0	1	1	37.0	109.2	50.7	31.2
0	0	1	0	0	1	23.7	37.8	30.4	30.4
0	0	1	1	0	1	12.4	39.1	26.1	26.0
0	0	1	0	0	1	27.0	50.3	22.6	36.8
0	0	1	1	0	2	21.1	43.6	46.9	24.7
1	0	0	0	1	1	33.1	39.2	26.2	37.0
0	0	1	1	1	1	27.5	38.5	25.3	40.5
1	0	0	1	0	1	15.9	26.4	33.2	28.5
1	0	1	0	0	1	10.2	20.5	7.9	35.1
0	0	0	1	0	2	29.3	59.3	101.6	25.6
1	0	0	0	1	1	18.4	23.9	40.4	24.6
1	0	0	1	1	1	6.9	13.4	18.7	28.6
1	0	1	0	1	1	14.2	35.0	27.1	25.3
1	0	1	0	0	1	12.9	34.6	28.2	27.3

0	0	1	0	0	1	131.7	150.6	130.0	24.0
0	0	0	0	0	2	38.0	39.0	94.9	37.3
1	0	1	0	0	1	46.9	100.9	178.1	25.2
1	0	1	0	0	1	32.4	23.2	10.4	29.7
0	0	1	0	1	1	22.5	49.0	134.6	31.0
0	0	1	0	0	1	35.4	68.5	43.6	26.9
0	0	0	0	0	1	48.3	41.6	16.9	41.1
0	0	0	0	0	1	20.6	27.5	28.6	30.0
0	0	0	0	0	2	34.3	45.8	51.6	29.6
0	0	0	0	0	1	47.2	56.6	47.8	26.1
1	0	1	1	0	1	16.8	44.1	148.3	25.9
0	0	1	0	0	1	24.1	42.4	37.8	24.2
1	0	1	0	0	1	13.4	26.6	14.2	28.1
1	0	1	0	0	1	42.2	83.8	13.6	22.0
1	0	0	0	0	2	14.3	20.8	48.1	32.5
0	0	1	0	1	2	20.2	28.1	38.2	30.1
0	0	0	0	1	1	32.1	169.8	107.6	31.0
1	0	1	0	0	1	25.0	30.8	10.2	25.2
0	0	0	0	0	1	18.1	30.7	35.6	29.2
0	0	1	0	0	1	14.4	21.0	35.0	33.8
0	0	1	0	0	1	7.6	21.1	19.4	30.8
1	0	0	0	1	2	40.3	58.2	38.5	26.6
1	0	1	1	1	1	419.0	1361.1	58.0	23.6
1	0	1	0	0	1	14.4	17.6	17.3	29.9
0	0	1	0	0	2	29.5	49.4	36.7	30.1
1	0	1	0	0	1	18.0	21.0	27.7	20.5
0	0	1	1	1	2	23.2	39.4	27.1	31.5
0	0	1	0	0	1	14.5	52.9	107.1	31.4
1	0	1	1	1	1	24.2	45.1	58.8	28.1
0	0	0	1	0	1	39.4	81.0	57.1	20.9
1	0	1	0	0	1	16.5	24.8	3.4	25.0
0	0	1	1	0	1	19.2	34.3	89.4	29.9
1	0	0	0	0	1	26.3	155.7	69.2	23.8
0	1	1	0	0	1	237.4	205.9	346.9	26.1
0	0	1	0	0	1	23.0	41.5	67.6	28.0
1	0	1	1	0	1	45.9	86.7	24.9	25.8
1	0	1	0	0	1	18.1	41.8	11.1	32.8
0	0	0	0	0	2	66.9	63.2	67.0	25.5
0	0	1	0	0	1	26.5	37.7	53.9	24.5
1	0	1	0	1	1	33.2	31.6	15.9	22.6
0	0	0	0	1	1	18.5	31.2	133.9	30.3
0	0	1	0	1	1	18.8	30.6	43.9	24.1
1	0	1	0	0	1	30.3	50.3	45.7	24.8
1	0	0	0	0	1	119.4	103.4	9.9	29.4
1	1	1	0	0	1	11.0	21.9	72.2	19.1

0	0	0	1	0	2	4.9	30.8	39.7	27.0
0	0	1	0	0	1	60.3	87.6	42.7	29.7
1	0	1	0	0	2	35.9	64.2	45.0	25.9
0	0	1	0	0	2	31.5	32.3	155.3	35.4
1	0	1	0	0	1	16.5	24.1	27.8	32.7
0	0	0	1	0	2	34.0	58.8	15.4	30.7
1	0	1	1	1	1	139.5	354.0	70.2	22.8
1	0	1	1	1	1	42.2	45.7	240.8	23.6
0	0	1	1	1	1	35.3	40.9	83.3	33.2
1	0	1	0	0	1	10.6	17.8	11.0	30.2
1	0	0	0	0	1	25.6	46.8	24.8	24.2
1	0	1	0	0	1	8.3	9.4	12.2	30.4
0	0	0	1	1	1	30.4	36.0	93.0	36.1
0	0	0	0	0	1	13.4	18.1	9.2	39.6
0	0	0	1	0	2	39.1	54.1	63.4	31.9
1	0	0	1	0	1	22.6	33.5	26.2	26.0
0	0	1	0	0	2	38.2	114.0	70.1	24.7
0	0	1	1	0	1	389.8	399.0	300.6	30.3
0	0	0	0	0	1	40.6	47.3	58.4	22.8
1	0	1	0	0	1	24.9	43.8	12.1	28.2
0	0	1	0	0	1	8.7	12.9	22.4	27.1
0	0	0	0	1	1	39.1	94.3	59.5	24.6
0	0	0	0	0	1	38.9	52.3	197.1	25.4
1	0	1	0	0	1	24.5	26.7	22.9	29.7
1	1	1	0	0	1	37.6	61.4	85.4	28.8
1	0	1	0	0	2	39.0	53.4	6.0	25.7
1	0	1	1	0	1	22.5	37.0	24.7	30.9
0	0	1	0	1	2	27.4	42.8	31.0	22.4
0	0	1	0	0	1	52.2	79.5	74.4	25.8
1	0	1	0	0	1	25.9	77.9	83.2	21.2
0	0	0	0	0	1	11.4	16.3	46.1	34.3
1	0	1	0	0	1	32.9	85.9	62.4	25.0
0	0	1	1	0	1	60.9	80.2	14.2	36.2
1	0	0	0	0	2	27.4	43.3	52.9	34.1
1	0	0	0	1	1	26.0	29.3	28.9	28.1
0	0	1	1	1	1	18.1	36.5	25.5	29.5
0	0	1	0	0	2	31.1	44.0	42.7	31.1
1	0	1	0	0	1	34.3	42.3	50.9	26.5
0	0	0	1	0	1	17.5	28.9	16.9	28.9
1	0	1	1	1	1	5.6	23.3	7.0	27.1
0	0	1	0	0	1	21.0	42.1	18.4	27.8
0	0	0	0	0	1	1617.7	1259.3	186.4	34.1
0	0	1	0	0	1	1398.2	591.4	162.1	32.9
0	0	0	0	0	2	34.7	42.1	53.3	33.2
0	0	1	1	0	1	21.0	53.1	59.0	33.4

0	0	1	0	0	1	27.7	44.0	38.3	28.5
0	0	0	0	1	1	121.0	115.7	26.1	28.6
1	0	0	1	0	1	7.6	20.7	15.2	24.3
0	0	1	1	1	1	10.4	18.1	58.8	32.3
0	0	1	0	0	1	751.4	246.4	337.6	33.2
0	0	0	0	0	1	36.4	53.0	13.9	37.4
0	0	1	1	1	1	17.6	32.1	82.0	28.5
1	0	1	0	0	1	36.5	59.0	56.1	25.4
1	0	1	0	0	1	22.6	67.8	175.8	29.0
1	0	1	0	1	1	65.0	318.8	103.6	23.8
0	0	1	0	0	1	28.6	36.3	49.1	32.5
0	0	0	0	0	1	22.0	29.9	48.1	36.9
1	0	1	0	0	1	50.6	72.2	36.9	26.8
0	0	0	0	0	1	21.4	44.5	91.4	30.5
0	0	0	0	0	1	52.4	140.9	254.9	36.7
0	0	0	0	0	1	23.4	31.6	31.8	32.2
1	0	1	1	0	1	23.8	31.6	15.1	22.2
1	0	1	0	1	1	13.6	23.2	7.6	25.6
0	0	1	0	1	1	39.5	68.9	158.1	24.0
0	0	1	0	0	1	24.6	58.6	31.5	38.7
0	0	0	0	1	1	42.2	41.8	22.4	29.0
1	1	1	1	1	1	12.7	28.6	33.4	23.0
0	1	1	0	0	1	29.9	31.8	30.8	27.1
0	0	1	0	0	2	17.3	28.2	51.5	25.0
0	0	1	0	0	1	79.6	89.4	250.6	25.4
1	0	1	0	0	1	35.7	55.0	16.2	30.1
1	0	1	0	1	1	13.5	16.8	36.9	33.3
0	0	1	0	0	1	85.7	66.5	376.4	29.0
0	0	1	0	0	2	17.9	16.6	18.1	33.2
0	0	1	1	0	2	30.7	34.2	18.8	33.7
1	0	1	0	0	1	21.4	26.4	14.8	38.3
1	0	1	1	1	1	13.1	37.4	36.9	29.1
0	0	1	0	0	1	7.7	30.8	110.8	22.3
1	0	1	0	0	1	19.6	35.8	79.4	24.0
0	0	0	0	1	1	18.6	25.3	31.2	28.2
0	0	1	0	0	1	22.9	101.3	115.1	23.7
1	0	0	0	0	1	20.7	60.6	24.5	26.3
0	0	0	0	0	1	57.0	145.4	73.3	39.6
0	0	1	0	0	1	14.3	22.9	43.2	30.2
0	0	1	0	1	1	22.9	30.1	35.8	35.4
0	0	1	1	0	1	19.1	35.6	48.7	23.1
0	0	0	0	0	1	24.2	39.1	24.7	33.7
0	0	1	0	1	1	16.6	28.1	23.0	31.4
0	0	1	1	0	1	29.3	114.4	94.3	27.9
1	0	1	0	1	1	556.3	487.0	8.0	31.4

1	0	0	0	1	1	13.2	18.2	6.5	32.0
0	0	0	0	0	1	274.1	187.8	406.9	30.7
0	0	0	0	0	1	60.0	61.8	25.1	32.3
0	0	0	1	0	1	30.8	47.6	31.0	29.4
0	0	0	0	0	1	29.7	48.8	69.7	25.2
0	0	0	1	0	1	40.8	69.3	61.5	28.5
0	0	1	1	0	1	16.9	31.6	47.5	24.3
1	0	1	1	0	1	32.4	128.4	118.4	20.3
0	0	0	1	0	1	1560.1	1670.2	91.1	45.6
1	0	1	1	1	1	13.9	16.1	18.1	35.7
0	0	0	1	0	2	17.4	27.5	26.3	31.5
1	0	1	0	0	1	16.2	15.7	8.3	24.5
1	0	1	0	1	1	17.2	21.5	13.7	28.9
0	0	1	0	0	1	47.2	62.9	75.3	25.8
0	0	1	0	0	1	33.4	41.6	47.2	28.3
1	0	1	0	0	1	34.0	36.0	16.3	30.6
1	0	0	0	1	1	53.7	53.6	45.9	30.7
0	0	1	0	1	1	18.6	26.3	70.8	31.0
0	0	0	0	1	2	14.6	23.8	33.3	37.1
1	0	1	0	0	1	258.6	325.2	22.9	30.2
0	0	1	0	0	1	14.5	24.5	66.2	29.1
0	0	1	0	0	1	26.9	33.3	44.2	29.4
1	0	1	0	0	1	11.7	17.7	18.9	21.8
1	0	1	0	0	1	16.4	24.9	33.0	31.0
0	0	1	0	0	1	40.8	69.5	120.7	26.1
1	0	1	0	0	1	18.5	37.9	27.4	12.2
0	0	0	0	0	1	32.6	20.3	66.0	30.9
0	0	0	0	0	1	10.7	37.2	50.4	30.3
0	0	0	0	0	1	48.2	70.7	33.5	34.6
0	0	0	0	0	2	13.8	28.5	41.7	30.4
0	0	1	1	0	1	7.2	12.0	10.1	28.1
0	0	1	0	0	2	34.9	38.6	35.1	32.2
0	0	1	0	0	1	20.8	30.8	60.5	27.9
1	0	0	0	1	1	25.1	26.1	17.7	34.9
0	0	0	1	0	2	69.7	80.6	20.1	27.4
0	0	0	0	1	1	30.0	36.3	47.6	32.6
1	0	0	0	0	1	30.2	43.9	12.9	29.4
1	0	1	0	0	1	26.1	104.4	112.1	37.6
0	0	1	0	0	1	9.1	25.0	89.2	31.8
1	0	1	0	0	1	45.6	51.7	246.7	26.6
1	0	1	1	0	1	20.5	31.4	20.4	28.5
0	0	0	0	0	1	20.1	22.1	60.7	30.3
0	0	0	0	0	1	1002.0	627.0	444.3	28.8
0	0	0	1	0	1	12.2	26.1	10.3	27.9
0	0	1	0	1	1	10.5	31.2	5.4	25.4

1	1	1	0	0	1	175.2	243.5	88.5	17.0
0	0	1	0	0	1	45.7	41.9	29.1	24.9
0	0	1	0	0	2	46.2	104.1	52.4	32.1
0	0	0	0	0	1	34.8	47.3	58.2	29.5
0	0	1	1	0	1	22.7	28.8	72.1	25.8
1	0	1	0	0	1	36.0	65.7	31.2	34.8
0	0	0	1	0	1	24.4	39.4	27.0	32.2
0	0	1	0	0	2	41.3	40.4	125.0	31.3
0	0	1	0	1	1	7.8	12.3	46.8	27.0
0	0	1	0	0	1	29.2	36.7	70.0	28.0
0	0	1	0	0	1	22.3	39.9	45.1	26.5
0	0	0	0	0	2	189.1	430.2	81.7	28.7
0	0	1	1	0	1	22.7	39.5	25.0	25.5
0	0	1	0	0	1	51.7	57.8	118.2	27.5
0	0	0	0	1	1	37.0	70.1	85.8	22.3
0	1	1	0	0	1	25.5	26.9	51.9	25.9
0	0	1	0	1	1	25.8	35.1	75.1	29.8
0	0	0	1	0	1	39.4	72.5	69.0	26.0
0	0	1	0	0	1	1032.9	343.1	307.3	32.0
1	0	1	0	0	1	17.9	19.3	14.0	32.6
1	0	1	0	0	2	211.3	128.8	50.8	25.8
0	0	1	0	0	1	42.4	107.8	65.3	21.8
0	0	1	0	1	1	12.7	14.4	31.3	26.0
1	0	1	1	1	1	24.0	39.7	75.8	25.5
0	0	1	0	0	1	15.2	22.7	31.9	35.4
0	0	1	0	1	2	21.0	54.0	130.7	29.6
0	0	0	0	1	1	28.4	27.8	14.1	36.0
0	0	0	0	1	1	20.2	30.1	47.6	30.9
1	0	1	1	0	1	10.8	17.0	14.6	25.6
0	0	1	0	0	1	30.9	64.0	47.3	28.9
0	0	0	0	0	2	31.5	56.2	22.6	27.9
1	0	1	0	1	1	30.2	37.9	30.5	29.3
0	0	1	0	0	1	22.1	45.6	54.5	26.3
0	0	1	1	0	2	36.6	50.8	44.7	35.4
1	0	1	1	1	1	10.6	21.0	9.6	33.5
1	0	1	0	1	1	41.3	93.6	238.0	29.0
1	0	1	1	1	1	30.8	33.0	10.6	28.2
0	0	1	0	0	1	67.0	93.5	173.7	28.1
1	0	0	0	0	1	59.5	108.6	27.1	34.2
1	0	0	1	0	1	12.9	22.7	31.5	27.3
1	0	1	0	0	2	25.1	49.0	27.9	27.0
1	0	0	0	0	1	115.2	34.2	17.1	24.2
0	0	1	1	1	1	33.0	36.0	39.3	38.6
0	0	1	0	0	1	47.5	50.9	37.5	29.5
1	0	1	0	0	1	16.2	19.8	72.8	25.1

0	0	1	0	1	1	94.5	669.1	85.2	40.3
0	0	1	0	0	1	21.2	53.8	145.8	32.7
0	0	0	0	1	1	43.2	60.9	35.8	29.6
0	0	1	0	0	1	41.8	56.2	36.5	31.5
1	0	1	0	0	1	316.1	185.8	143.1	20.8
1	0	1	0	1	1	55.4	343.4	29.5	19.0
0	0	0	0	0	1	71.3	76.9	52.5	33.4
1	0	0	0	0	2	40.5	50.5	20.3	25.6
0	0	1	0	0	1	38.6	52.2	83.6	26.8
1	0	1	1	1	1	769.1	875.0	53.4	23.6
0	0	0	0	0	1	23.1	34.8	28.6	30.2
0	0	1	0	0	1	23.1	23.4	50.4	31.5
0	0	0	0	1	1	27.1	34.1	34.5	32.1
0	0	0	0	0	1	20.0	32.8	33.7	33.7
0	0	1	0	0	1	64.5	144.3	475.2	31.9
0	0	0	0	1	1	18.7	11.4	35.2	29.6
1	0	1	0	0	1	18.2	25.7	24.7	26.6
1	0	1	0	0	2	26.5	43.3	32.2	18.9
0	0	1	0	0	1	31.5	62.9	111.4	25.7
1	0	1	0	0	2	24.8	56.8	104.8	31.2
0	0	1	0	0	1	19.7	61.8	95.7	30.1
0	0	0	1	0	1	44.5	151.0	107.5	30.8
1	0	1	1	1	1	16.0	20.3	13.0	32.4
1	0	0	0	0	1	24.2	28.6	7.5	29.7
0	0	0	0	0	2	25.2	25.9	11.6	37.1
0	0	1	0	0	2	11.5	19.1	25.1	29.7
1	0	1	1	0	2	174.9	376.5	190.4	28.4
1	0	1	1	1	2	18.2	29.8	35.2	33.2
0	0	1	0	0	1	24.9	44.6	48.6	28.7
0	0	0	0	0	1	128.8	160.4	12.7	29.5
1	0	1	0	0	1	125.1	62.4	121.7	33.5
1	0	1	0	0	1	28.5	36.4	22.7	19.8
0	0	1	0	0	1	231.4	109.5	269.9	27.0
0	0	0	0	0	1	23.7	56.9	48.8	35.5
1	0	1	0	0	1	33.5	48.0	28.3	23.8
1	0	0	0	1	1	10.7	15.6	18.0	19.8
0	0	0	0	0	1	92.3	96.2	76.0	31.5
0	0	1	0	0	2	27.5	44.8	50.5	32.9
0	0	0	0	0	1	57.0	85.9	16.8	31.4
0	0	0	0	0	2	21.1	49.6	12.3	31.9
0	0	1	0	0	1	13.9	39.9	16.3	26.4
0	0	1	0	0	1	12.5	25.2	106.6	31.2
0	0	0	0	1	1	20.9	25.0	24.0	35.9
0	0	0	1	1	1	14.5	29.4	27.0	32.9
0	0	0	0	1	1	30.7	71.6	75.1	18.2

0	0	0	0	0	1	17.0	22.6	52.7	34.3
0	0	1	0	0	1	279.5	182.2	379.8	32.3
1	0	1	0	0	1	9.5	13.9	4.9	35.0
1	0	1	0	0	1	32.0	81.5	632.0	25.2
0	0	1	0	0	1	693.4	545.1	213.3	30.3
0	1	1	0	0	1	17.9	56.2	79.9	23.7
0	0	0	0	0	2	129.0	155.9	34.3	30.4
0	0	1	0	0	1	24.0	31.0	27.7	34.6
1	0	1	0	1	1	19.9	39.1	19.3	28.0
1	0	1	0	0	2	22.3	54.1	94.9	28.2
1	0	0	0	1	1	24.5	29.7	17.0	34.2
0	0	0	0	0	1	19.5	31.3	32.9	31.5
0	0	0	1	1	1	703.4	145.0	368.8	30.3
0	0	1	0	0	1	45.3	56.4	34.4	27.7
0	0	0	0	0	1	23.6	36.9	34.3	28.5
0	0	1	0	1	1	71.6	77.5	88.7	33.1
0	0	0	0	0	2	35.7	54.5	104.2	26.3
0	0	0	0	0	1	25.5	43.3	21.7	33.1
0	0	1	0	1	1	33.4	54.2	23.8	24.6
1	0	0	0	0	1	20.5	25.6	30.0	34.1
1	0	1	0	0	1	20.7	32.7	24.5	28.2
0	0	1	0	0	1	113.6	187.0	244.8	31.9
1	0	0	0	0	1	14.3	19.5	29.7	22.1
1	0	0	1	0	1	7.3	34.3	19.7	30.3
1	0	1	0	1	1	75.6	116.7	217.4	37.1
0	0	0	0	0	1	12.2	19.5	3.3	28.1
0	0	1	0	0	1	85.2	201.8	29.1	30.8
0	0	1	0	0	1	100.7	71.3	261.7	34.9
0	0	0	0	0	2	19.3	27.2	44.9	34.2
0	0	0	1	1	2	27.3	42.5	67.1	38.5
0	0	0	1	1	1	50.5	112.4	150.4	32.9
0	0	0	0	0	1	10.2	14.3	27.1	35.9
1	0	1	0	0	1	19.9	33.7	12.4	35.7
0	0	0	0	1	1	22.4	47.5	28.1	35.1
0	0	1	0	1	1	21.8	19.5	10.2	31.7
1	0	1	0	0	2	61.9	90.9	31.7	31.6
0	0	1	0	0	1	31.7	91.1	34.5	30.5
1	0	1	0	0	2	11.7	12.5	16.1	25.9
1	0	0	1	1	1	12.6	19.7	8.6	29.1
1	0	1	0	0	1	10.8	28.2	30.5	23.1
1	0	1	0	1	1	20.4	22.8	32.4	31.6
0	0	0	0	0	1	20.9	26.6	50.5	25.5
0	0	0	0	0	1	466.0	373.7	314.4	31.1
1	0	1	0	0	1	31.0	97.3	49.8	18.4
0	0	0	0	1	2	55.9	45.2	20.2	28.5

1	0	0	1	1	1	22.2	28.7	30.6	37.8
0	0	1	0	0	1	43.1	138.1	312.1	37.3
1	0	1	0	0	1	29.6	39.8	89.7	26.5
1	0	1	0	1	1	33.3	34.7	21.0	17.9
0	0	1	1	1	1	43.5	57.3	38.4	37.2
1	0	1	1	0	1	11.2	16.7	7.2	16.5
0	0	0	0	0	1	16.5	24.5	22.9	31.6
0	0	0	0	0	1	44.0	85.9	76.3	31.0
1	0	1	0	0	1	19.2	21.3	12.3	28.3
0	0	0	0	1	1	218.8	248.4	30.8	27.8
0	0	0	0	0	1	23.6	46.0	11.9	17.9
1	0	1	0	0	1	14.5	35.2	66.1	23.0
1	0	1	0	0	1	63.9	52.8	18.6	21.1
0	0	0	0	0	1	25.2	31.3	27.9	37.4
0	0	1	0	0	1	350.1	268.6	367.0	26.5
0	0	0	0	1	1	83.4	98.8	130.3	32.9
0	0	1	0	0	1	34.2	44.0	73.7	32.0
0	0	0	0	0	1	39.3	54.1	37.0	23.6
1	0	1	0	0	1	23.4	70.7	27.8	32.2
0	0	1	1	0	1	48.6	64.9	35.5	33.3
0	0	0	0	0	2	48.9	54.6	93.3	38.5
0	0	0	0	1	1	1607.5	1696.9	226.6	31.0
0	0	1	0	0	1	19.0	33.7	37.6	30.6
1	0	0	0	0	1	18.1	46.5	27.7	32.7
0	0	0	1	0	1	41.1	74.5	63.4	30.2
0	0	1	0	0	2	47.8	122.6	64.2	24.0
0	0	0	0	0	1	28.1	43.1	31.9	31.3
0	0	0	0	1	1	25.4	61.3	52.5	37.8
1	0	1	0	0	1	31.7	79.9	57.7	14.8
0	0	1	0	0	1	35.1	67.7	452.0	28.0
0	0	1	0	0	2	40.8	37.0	60.2	19.9
1	0	0	1	1	1	27.7	35.5	27.4	25.5
0	0	1	1	1	1	17.6	21.0	17.0	30.6
0	0	0	1	0	1	92.6	119.0	18.8	32.9
1	0	1	0	1	1	67.7	49.9	176.0	24.6
1	0	1	0	1	1	10.4	11.8	12.0	34.0
1	0	1	0	0	1	27.2	29.3	21.7	25.7
0	0	1	0	0	1	232.1	197.0	294.3	29.7
0	0	1	0	0	1	19.1	33.4	18.6	23.5
1	0	0	0	1	1	46.4	51.9	78.9	29.0
1	0	1	0	0	1	34.1	37.3	11.6	25.1
0	0	1	0	0	2	23.8	30.9	35.5	29.9
1	0	1	0	1	1	14.9	28.7	33.6	22.9
1	0	0	0	0	1	19.1	16.7	14.0	38.4
1	0	0	0	1	1	22.7	23.8	24.2	26.7

0	0	1	0	0	1	9.0	14.2	11.5	34.8
0	0	0	0	0	1	539.5	334.8	429.5	36.3
0	0	1	0	1	1	26.1	29.7	58.0	32.0
1	0	0	0	0	1	38.7	60.4	23.6	25.9
0	0	1	0	0	1	52.4	99.9	30.9	25.2
1	0	1	0	1	1	17.3	25.4	54.1	25.5
0	0	1	0	1	2	6.0	21.4	35.8	35.7
0	0	0	0	0	1	13.1	61.2	58.5	26.7
0	0	1	0	0	1	26.9	50.9	73.7	25.8
0	0	0	0	0	1	28.2	48.9	44.6	27.5
0	0	0	0	0	1	34.5	44.6	33.8	28.8
1	0	0	0	1	1	30.4	34.0	23.9	29.4
0	0	0	0	1	1	18.4	33.8	24.2	27.2
0	0	0	0	0	1	813.9	250.2	220.9	31.0
0	0	0	0	0	1	17.6	22.7	8.6	35.6
0	0	0	0	0	1	17.9	86.3	15.0	44.7
0	0	1	0	1	1	20.8	26.6	69.5	26.6
1	0	1	0	0	1	18.1	38.6	54.3	29.8
1	0	1	0	0	1	27.9	33.3	29.4	26.9
0	0	1	0	0	1	27.0	61.2	20.1	35.1
0	0	1	0	1	1	18.4	23.0	18.3	34.6
0	0	0	0	0	1	50.3	131.4	57.7	33.3
0	0	0	1	0	1	17.1	29.3	90.1	47.3
0	0	1	0	1	1	32.1	64.5	133.6	35.3
0	0	0	0	0	1	41.0	74.6	231.8	27.3
0	0	1	0	0	1	7.5	29.3	170.4	28.1
0	0	0	0	0	1	30.0	43.4	16.8	29.5
0	0	1	0	0	1	2141.9	2070.8	306.9	37.4
0	0	0	0	0	1	31.1	43.3	66.0	36.2
1	0	1	0	0	1	11.0	9.8	31.1	21.2
0	0	0	0	1	1	129.3	114.0	50.3	27.4
0	0	0	0	0	1	24.5	37.7	54.5	26.7
1	0	1	0	0	1	29.2	30.2	46.4	24.7
1	0	0	0	0	1	22.6	27.1	20.7	30.5
1	1	1	0	0	1	130.5	585.7	82.6	18.2
1	0	1	0	0	1	40.0	85.6	19.3	17.3
1	0	1	0	0	1	111.5	380.1	414.4	25.9
1	0	1	0	1	2	24.9	34.1	29.5	39.7
1	0	0	1	0	1	17.9	39.7	38.0	34.4
0	0	1	0	0	1	151.1	329.8	165.4	26.9
1	0	1	0	0	1	34.4	37.6	41.7	28.4
1	0	0	0	0	1	20.9	29.5	15.0	37.0
1	0	0	0	1	1	22.0	19.8	6.7	30.2
1	0	1	0	0	1	19.0	28.5	15.8	28.8
0	0	1	1	1	1	22.1	34.2	132.9	29.6

1	0	0	0	1	1	18.4	25.0	12.3	31.5
1	0	1	0	0	1	34.3	67.2	15.5	28.2
0	0	0	0	1	2	2991.7	1205.0	253.4	31.4
1	0	1	0	0	2	21.3	41.5	17.0	18.0
1	0	1	0	0	2	21.1	30.8	41.1	22.8
0	0	1	0	0	1	36.4	47.4	25.6	31.8
0	0	1	0	0	2	38.1	49.2	39.8	23.7
0	1	1	0	0	1	19.2	61.5	48.8	23.7
1	0	0	0	0	1	86.9	96.6	9.7	32.7
1	0	0	0	0	1	19.6	31.9	28.5	25.9
0	0	0	0	0	1	61.0	86.6	83.6	25.5
0	0	0	0	0	2	29.4	29.9	16.1	40.1
1	0	0	0	0	1	43.0	44.8	14.7	28.7
0	0	1	0	0	2	28.9	46.9	22.5	23.6
0	0	1	0	1	2	14.6	27.9	15.7	29.3
1	0	1	0	0	1	351.7	2229.8	81.8	19.6
1	0	0	0	0	1	71.1	90.3	378.2	22.4
1	0	0	0	0	1	13.2	18.3	7.3	28.1
1	0	1	1	0	1	2204.6	756.7	269.5	27.0
1	0	1	0	0	1	33.1	214.0	154.0	16.0
0	0	1	1	0	1	66.9	86.9	64.7	13.8
0	0	0	0	0	2	291.7	374.9	616.9	20.3
1	0	1	0	0	1	188.6	520.4	442.8	28.2
0	0	0	1	0	1	32.1	72.7	263.1	29.3
1	0	1	1	0	1	45.7	33.4	244.1	31.6
1	0	1	0	1	1	15.1	21.8	22.2	25.2
0	0	1	0	0	1	253.1	474.7	294.4	28.6
1	0	1	0	0	1	1236.5	1202.2	214.1	25.7
0	0	1	0	0	1	17.5	27.4	35.5	26.9
1	1	1	0	0	1	162.9	529.4	502.9	33.4
1	0	1	1	1	2	2440.7	2238.0	435.3	32.4
0	0	1	0	0	1	4.0	23.0	149.5	31.2
0	0	1	0	0	1	151.2	149.8	854.5	27.3
1	0	1	0	0	1	55.0	208.1	343.5	32.8
0	1	1	1	0	1	12.4	26.3	30.5	25.3
1	0	1	0	0	1	14.7	32.0	60.5	13.3
0	0	1	0	0	1	12.5	49.1	106.4	25.1
0	0	0	0	1	2	48.7	77.2	300.6	16.8
1	0	1	1	0	1	58.8	70.6	234.6	31.4
0	0	1	1	1	1	746.5	670.1	192.3	29.2
1	0	1	0	1	1	2194.0	2537.0	116.2	26.5
1	0	1	0	0	1	38.0	229.1	366.1	30.6
1	0	0	0	0	1	60.5	190.4	158.3	21.7
1	0	1	0	0	1	34.3	45.9	286.3	29.4
1	0	1	0	0	1	149.3	151.7	488.9	33.4

1	0	1	0	0	1	62.7	170.9	425.9	27.4
0	0	1	0	0	1	109.4	284.1	221.8	16.7
0	0	1	1	1	1	118.0	224.9	251.6	24.2
1	0	1	0	0	1	93.2	139.0	418.3	22.8
0	0	1	0	0	1	279.6	197.8	674.0	28.5
0	0	1	1	1	1	1381.2	864.7	433.3	34.4
0	0	1	0	0	1	78.7	123.5	576.3	27.6
1	1	1	0	0	1	25.0	47.5	21.9	30.2
1	1	1	1	0	1	110.6	120.5	185.2	32.6
1	0	1	1	0	1	8.4	14.6	64.5	30.4
0	0	1	0	0	1	65.7	123.0	239.2	30.5
0	0	1	0	0	1	72.4	103.3	359.9	19.8
1	0	0	1	0	2	29.5	51.4	52.4	31.2
0	0	0	0	0	1	375.6	243.1	285.2	26.7
0	0	1	0	0	1	90.0	228.7	296.7	24.9
0	0	1	0	1	1	72.3	64.8	226.9	29.9
1	0	0	1	0	1	32.6	49.3	47.6	32.4
0	0	1	0	0	2	25.0	43.6	42.3	24.4
1	0	1	0	0	1	35.1	59.6	151.8	26.2
1	0	0	0	0	1	19.5	35.4	139.8	20.7
0	1	1	0	0	1	25.4	39.3	174.1	22.2
1	1	1	0	0	1	448.5	433.1	419.1	25.3
0	0	1	1	1	1	12.9	27.3	147.0	29.2
0	0	1	0	0	1	56.5	106.8	237.4	20.0
1	0	1	0	1	2	111.2	157.6	17.2	22.2
1	0	1	0	0	1	56.6	98.4	728.7	31.6
1	0	1	0	0	1	24.1	39.9	464.9	22.9
1	1	1	0	0	1	29.9	40.4	23.5	30.1
0	0	1	0	0	1	14.2	26.4	13.1	34.4
0	0	0	1	0	1	273.6	165.2	554.0	29.3
1	0	1	0	0	1	390.2	547.7	72.2	19.7
0	0	1	0	1	1	77.0	96.5	703.8	26.3
0	1	1	1	0	1	222.9	383.0	523.9	25.8
0	0	1	0	0	1	146.9	275.8	775.6	32.0
1	0	1	0	1	1	17.0	14.3	58.9	16.3
0	1	1	1	0	1	776.9	867.9	78.3	28.5
1	0	1	0	0	1	43.0	188.8	606.5	25.2
0	0	1	0	1	1	25.2	41.9	111.4	24.0
0	0	0	0	0	1	2026.7	892.0	324.0	36.2
1	0	1	0	0	1	243.0	277.6	19.0	22.6
1	0	1	0	0	1	11.5	20.9	14.5	37.3
1	0	0	0	1	1	45.1	37.0	17.1	39.0

GLO	GGT	ALP	TC	TG	HDL-C	group1	LDL	WBC	NE#
28.7	629.60	92.3	3.72	1.98	0.54	1.00	2.62	14.80	13.000
12.4	145.50	201.3	1.25	0.39	0.09	0.00	0.48	2.52	1.900
31.1	13.50	89.2	3.98	0.65	0.74	1.00	2.67	6.00	4.900
9.7	13.10	24.2	0.96	0.34	0.30	0.00	0.54	5.30	4.500
17.4	3.80	25.8	0.91	4.37	0.15	0.00	0.43	6.56	2.200
7.4	6.90	19.2	0.52	0.08	0.24	0.00	0.17	9.70	6.120
19.8	8.90	72.3	0.70	0.22	0.05	0.00	0.17	25.34	22.250
29.4	15.40	43.5	1.14	0.15	0.29	0.00	0.67	11.36	9.340
27.2	47.85	99.0	2.40	0.56	0.49	0.00	1.14	13.03	10.890
12.0	47.85	99.0	2.40	0.56	0.49	0.00	1.14	20.12	17.220
31.0	47.85	99.0	2.40	0.56	0.49	0.00	1.14	8.12	5.470
29.7	348.80	49.5	3.18	7.05	0.09	0.00	0.39	4.97	4.110
23.2	47.85	99.0	2.40	0.56	0.49	0.00	1.14	2.26	1.320
23.9	47.85	99.0	2.40	0.56	0.49	0.00	1.14	10.28	7.770
25.4	47.85	99.0	2.40	0.56	0.49	0.00	1.14	21.42	20.030
14.4	47.85	99.0	2.40	0.56	0.49	0.00	1.14	11.54	8.810
13.9	47.85	99.0	2.40	0.56	0.49	0.00	1.14	8.51	7.410
26.9	47.85	99.0	2.40	0.56	0.49	0.00	1.14	13.59	8.740
20.3	35.60	61.6	2.95	0.70	0.63	1.00	1.71	8.74	8.020
17.8	47.85	99.0	2.40	0.56	0.49	0.00	1.14	11.22	10.420
41.3	16.20	142.2	2.40	0.56	0.49	0.00	1.14	3.82	3.600
28.7	47.85	99.0	2.40	0.56	0.49	0.00	1.14	14.41	12.800
31.1	6.90	51.2	0.72	0.10	0.08	0.00	0.36	4.90	3.600
29.7	198.20	356.4	3.11	1.15	0.11	0.00	1.49	7.40	6.200
18.5	98.30	225.2	2.48	0.72	0.08	0.00	1.36	4.54	3.790
32.8	357.80	92.3	2.02	0.69	0.15	0.00	1.03	13.99	13.110
25.6	207.20	212.3	2.67	1.22	0.25	0.00	0.76	5.72	4.070
27.6	90.30	281.0	0.30	0.23	0.03	0.00	0.03	16.70	13.930
18.0	71.20	109.1	0.51	0.22	0.17	0.00	0.13	5.86	5.200
15.4	135.30	203.4	1.35	0.54	0.04	0.00	0.11	30.89	27.700
19.7	47.85	99.0	2.40	0.56	0.49	0.00	1.14	7.22	6.170
15.8	46.30	58.4	1.21	0.35	0.07	0.00	0.30	9.72	8.210
23.6	88.60	111.9	1.60	0.65	0.12	0.00	0.68	32.35	28.610
28.1	8.60	77.9	1.75	0.30	0.15	0.00	0.98	8.37	6.990
26.5	251.30	60.6	2.58	0.75	0.27	0.00	1.56	8.84	6.690
33.8	1407.90	939.8	9.03	2.44	0.25	0.00	8.88	7.51	3.690
26.8	74.20	207.5	2.61	0.67	0.88	1.00	1.06	4.03	2.710
26.3	17.70	107.7	0.92	0.32	0.32	0.00	0.42	5.23	4.480
30.1	13.40	61.0	3.14	0.43	1.51	1.00	1.73	1.63	0.940
28.3	43.90	175.1	1.38	0.32	0.45	0.00	0.98	2.40	0.900
23.9	202.50	186.7	2.89	1.15	0.33	0.00	1.37	5.10	3.100
46.1	70.80	209.7	2.40	0.56	0.49	0.00	1.14	4.70	3.500
34.8	179.40	124.6	2.23	1.23	0.28	0.00	1.72	8.10	5.700
40.9	21.90	105.9	2.02	0.45	1.22	1.00	0.76	4.73	2.220

32.3	105.80	154.5	2.36	0.45	0.12	0.00	1.10	14.40	10.300
29.7	47.85	99.0	2.40	0.56	0.49	0.00	1.14	4.00	3.200
16.6	64.30	151.9	0.41	0.15	0.12	0.00	0.29	10.80	9.500
39.5	129.50	146.8	2.15	0.43	1.02	1.00	1.28	3.69	1.980
48.9	12.20	77.1	2.38	0.43	1.05	1.00	1.74	2.72	2.000
40.2	305.50	116.4	2.18	0.90	0.56	1.00	1.65	6.50	4.600
23.4	26.90	74.2	1.06	0.44	0.07	0.00	0.17	17.61	15.040
42.5	40.10	146.5	2.97	0.76	1.14	1.00	1.79	3.12	2.330
27.0	18.20	100.9	1.11	0.50	0.19	0.00	0.45	8.60	7.900
29.0	88.00	127.7	2.61	1.18	0.12	0.00	1.05	9.75	7.250
36.8	47.85	99.0	2.40	0.56	0.49	0.00	1.14	14.60	12.900
44.5	21.20	73.1	1.34	0.41	0.21	0.00	0.63	6.13	5.100
36.2	44.80	73.6	2.17	0.25	1.11	1.00	1.07	7.90	4.200
29.7	47.85	99.0	2.40	0.56	0.49	0.00	1.14	15.41	13.900
41.8	11.00	96.5	1.17	0.44	0.36	0.00	0.34	14.81	12.400
13.0	25.50	73.5	0.86	0.43	0.14	0.00	0.22	7.80	6.500
36.4	43.30	107.8	1.35	0.79	0.20	0.00	0.61	4.70	3.200
47.3	144.10	195.6	0.96	0.36	0.39	0.00	0.21	17.32	15.630
36.3	47.85	99.0	2.40	0.56	0.49	0.00	1.14	7.60	6.800
39.9	62.70	114.7	1.96	0.49	0.71	1.00	1.00	5.20	2.700
27.0	19.20	25.4	0.84	0.30	0.27	0.00	0.21	7.82	7.240
29.5	74.30	142.4	1.78	0.42	0.42	0.00	1.10	5.30	4.800
40.9	40.80	103.6	3.01	0.63	1.28	1.00	1.27	5.60	4.100
51.1	21.60	100.0	2.06	0.71	0.65	1.00	0.90	10.84	8.800
16.3	17.10	37.7	0.77	0.49	0.19	0.00	0.22	3.92	3.600
40.9	25.80	158.4	2.79	1.18	0.62	1.00	1.65	3.42	3.000
29.7	47.85	99.0	2.40	0.56	0.49	0.00	1.14	4.20	3.600
29.4	20.80	174.5	2.62	0.61	1.37	1.00	1.00	3.42	2.300
40.2	74.30	128.7	1.84	0.94	0.17	0.00	0.80	6.50	5.400
35.1	9.20	79.5	2.10	0.46	0.89	1.00	1.09	1.72	1.000
29.8	23.30	88.4	0.93	0.42	0.21	0.00	0.22	15.41	12.900
28.0	57.90	29.7	1.58	0.59	0.29	0.00	0.73	5.50	4.600
24.5	14.00	58.5	1.72	0.36	0.51	1.00	0.79	2.80	2.000
23.7	80.40	98.6	0.83	0.08	0.52	1.00	0.51	3.02	2.300
29.3	70.40	195.4	1.90	0.80	0.59	1.00	1.01	1.72	1.100
51.4	103.30	191.5	2.47	1.68	0.11	0.00	0.96	14.81	12.500
52.3	157.90	301.1	4.26	1.59	0.16	0.00	2.29	10.84	8.700
30.4	473.90	144.7	4.42	3.20	0.12	0.00	2.07	11.78	9.380
34.0	22.50	202.4	1.34	0.48	0.10	0.00	0.64	6.00	4.300
18.7	7.60	48.9	1.24	0.40	0.43	0.00	0.45	17.01	15.900
34.8	14.90	108.2	2.40	0.56	0.49	0.00	1.14	4.00	3.100
36.8	97.90	118.0	2.59	0.81	0.74	1.00	1.16	3.54	3.110
26.9	31.80	121.3	3.57	0.66	1.58	1.00	1.47	5.64	3.570
32.1	98.30	250.9	3.52	3.33	0.15	0.00	1.42	5.70	4.700
26.9	26.00	88.4	3.31	0.59	1.26	1.00	1.64	2.51	1.670

34.5	35.40	71.0	4.69	1.21	0.69	1.00	3.10	6.45	4.670
31.5	21.60	107.7	3.52	1.08	0.60	1.00	2.19	2.88	2.050
42.5	118.80	197.0	1.52	0.47	0.11	0.00	0.59	12.74	9.000
40.2	7.00	80.1	1.21	0.28	0.48	0.00	0.61	1.19	0.730
36.3	57.60	183.5	2.57	0.90	0.62	1.00	1.28	2.67	1.170
36.1	37.30	239.6	3.01	0.54	0.96	1.00	1.35	4.16	2.180
34.3	38.30	94.3	2.87	0.60	1.09	1.00	1.19	3.58	2.380
48.0	51.00	135.1	1.99	0.63	0.78	1.00	0.43	2.12	1.360
22.3	80.20	137.6	1.34	0.19	0.49	0.00	0.61	5.93	5.550
27.9	61.30	61.6	1.86	0.20	0.60	1.00	0.92	2.11	1.430
36.4	38.80	102.9	3.62	0.95	1.45	1.00	1.44	4.10	3.120
32.1	29.80	124.1	1.21	0.54	0.06	0.00	0.21	12.44	9.910
30.4	21.10	68.6	2.40	0.56	0.49	0.00	1.14	4.07	2.530
11.8	9.30	29.7	2.40	0.56	0.49	0.00	1.14	9.48	8.440
32.4	417.20	136.4	2.07	0.82	0.11	0.00	1.07	8.44	6.540
31.6	22.00	81.9	3.71	0.77	1.08	1.00	1.72	2.14	1.280
12.8	129.70	99.1	2.00	0.60	0.14	0.00	1.09	12.58	11.500
16.3	33.50	40.5	1.69	0.62	0.53	1.00	0.69	6.28	3.820
40.2	40.40	26.0	0.91	0.38	0.09	0.00	0.34	5.02	4.420
37.8	25.00	100.8	3.16	0.48	0.42	0.00	1.64	10.28	8.830
30.8	95.40	124.1	1.30	0.39	0.11	0.00	0.52	8.01	5.220
39.4	46.60	137.0	1.59	0.53	0.17	0.00	0.59	7.12	4.760
35.2	75.90	147.0	1.65	0.67	0.14	0.00	0.77	6.02	4.350
38.6	14.90	62.0	3.01	0.37	0.37	0.00	2.00	3.20	1.360
34.1	356.70	161.1	2.99	0.99	0.13	0.00	1.61	5.28	3.060
23.7	16.70	220.3	2.10	0.53	0.38	0.00	0.79	2.52	1.880
29.8	46.90	141.7	2.92	0.84	1.01	1.00	1.20	3.13	2.240
24.0	69.70	141.8	1.47	0.43	0.11	0.00	0.41	9.23	7.010
26.2	23.60	88.7	1.28	1.11	0.04	0.00	0.14	7.14	6.110
45.7	36.00	35.0	2.53	6.88	0.15	0.00	0.73	3.89	3.080
24.7	41.80	51.9	2.53	0.12	0.22	0.00	1.69	11.92	9.960
26.8	648.30	91.7	2.79	0.76	0.42	0.00	2.04	10.15	7.270
41.5	47.85	99.0	2.40	0.56	0.49	0.00	1.14	6.87	5.960
40.0	47.85	99.0	2.40	0.56	0.49	0.00	1.14	6.44	5.040
36.8	13.30	109.7	1.98	0.45	0.90	1.00	0.53	1.90	0.920
19.5	58.70	69.2	2.28	1.67	0.09	0.00	0.83	24.15	20.650
31.2	79.50	76.7	1.37	0.60	0.12	0.00	0.69	5.13	3.660
34.1	34.50	51.2	2.35	0.77	0.54	1.00	1.13	6.02	4.890
32.5	38.00	82.2	5.01	0.46	1.95	1.00	1.57	3.76	1.320
39.4	47.85	99.0	2.40	0.56	0.49	0.00	1.14	5.66	4.150
47.7	47.85	99.0	2.40	0.56	0.49	0.00	1.14	8.51	5.350
43.8	277.30	102.2	2.63	1.27	0.53	1.00	1.29	4.58	2.990
20.8	162.00	132.0	3.41	0.70	0.21	0.00	2.02	7.17	6.000
42.6	180.70	81.7	1.74	1.39	0.06	0.00	0.80	7.69	4.810
33.7	16.50	120.7	2.17	0.47	0.26	0.00	0.69	8.78	4.450

26.3	8.10	12.3	2.13	1.08	0.02	0.00	0.02	10.32	5.270
38.5	102.00	109.8	1.61	0.46	0.11	0.00	0.64	4.38	2.500
39.4	34.50	96.0	2.12	0.75	0.27	0.00	1.07	4.33	2.120
45.1	28.20	99.0	2.37	0.66	0.18	0.00	1.21	5.00	2.410
40.7	29.50	85.7	2.80	0.47	0.22	0.00	1.04	4.38	2.870
27.5	50.80	103.0	3.01	0.34	1.14	1.00	1.33	5.85	3.750
48.6	44.10	114.5	1.11	0.47	0.09	0.00	0.32	9.18	5.290
24.9	19.60	79.1	2.19	0.51	0.85	1.00	0.79	2.29	1.450
35.4	171.40	213.1	1.97	0.45	0.13	0.00	0.73	13.91	11.300
17.9	46.60	83.9	1.76	0.57	0.14	0.00	0.53	6.49	4.820
28.4	40.20	145.1	2.94	1.01	0.81	1.00	1.18	5.62	3.590
45.9	182.60	201.1	3.44	0.93	0.99	1.00	1.35	5.97	3.600
44.5	156.10	210.0	5.75	0.86	1.08	1.00	2.63	4.26	3.960
28.7	10.70	72.2	3.00	0.83	0.51	1.00	1.95	2.07	1.200
28.0	13.70	51.7	2.98	1.28	0.69	1.00	1.46	2.12	1.820
32.5	29.40	71.0	2.45	0.52	1.07	1.00	0.91	2.94	1.520
23.4	79.60	66.2	1.69	0.24	0.61	1.00	0.72	3.50	2.330
22.2	28.90	57.8	2.37	0.87	0.64	1.00	1.28	11.27	8.320
34.9	96.60	109.1	2.07	1.86	0.23	0.00	1.04	4.38	2.370
35.1	45.90	152.1	3.21	0.82	0.18	0.00	1.70	2.03	1.240
20.4	47.85	99.0	2.40	0.56	0.49	0.00	1.14	3.48	1.820
23.0	143.60	99.9	2.06	0.51	0.14	0.00	0.84	4.92	3.780
29.8	19.80	47.5	2.88	0.51	1.03	1.00	0.83	18.39	16.380
19.8	12.40	33.6	1.49	0.65	0.32	0.00	0.69	2.16	1.670
31.5	25.60	112.1	4.19	0.70	1.18	1.00	2.25	8.57	5.160
35.7	43.70	102.4	2.04	0.27	0.72	1.00	0.82	4.68	2.530
34.2	72.20	96.4	5.46	1.96	0.76	1.00	2.40	4.38	2.740
37.4	29.50	141.1	2.36	0.72	0.85	1.00	0.62	3.28	1.640
48.9	322.10	67.5	1.49	0.50	0.09	0.00	0.54	13.94	10.370
31.8	33.20	75.7	5.79	0.73	1.47	1.00	3.31	7.49	3.020
38.7	26.00	133.8	1.25	0.48	0.09	0.00	0.33	5.28	3.450
34.3	170.00	146.5	2.81	0.95	0.16	0.00	1.29	7.78	5.850
26.0	47.85	99.0	2.40	0.56	0.49	0.00	1.14	5.03	4.290
34.9	55.90	199.4	3.41	1.25	0.68	1.00	1.28	4.65	2.090
26.6	16.20	68.4	2.09	0.59	0.59	1.00	0.86	2.58	1.770
30.9	112.20	157.5	1.61	0.46	0.14	0.00	0.70	8.45	5.430
30.1	20.20	77.0	2.12	0.33	0.95	1.00	0.85	2.20	1.530
36.4	77.10	196.5	2.76	1.86	0.64	1.00	0.86	2.93	1.400
32.5	22.90	77.0	1.70	0.57	0.48	0.00	0.69	4.36	2.910
46.1	27.90	91.3	1.47	0.61	0.12	0.00	0.63	4.97	3.840
19.3	62.90	241.8	1.42	0.23	0.56	1.00	0.48	8.92	7.060
16.2	155.60	94.1	5.43	0.53	0.21	0.00	3.48	19.75	17.150
29.5	150.50	15.6	1.09	0.90	0.14	0.00	0.32	10.72	8.860
30.5	161.80	54.0	2.19	2.48	0.10	0.00	0.56	10.35	8.120
31.2	16.90	55.5	2.20	0.52	0.93	1.00	0.64	3.43	2.300

26.0	19.70	83.8	1.18	0.65	0.08	0.00	0.27	11.53	9.670
42.5	20.90	114.0	2.94	0.54	0.86	1.00	1.57	7.56	2.780
19.6	47.85	99.0	2.40	0.56	0.49	0.00	1.14	3.40	2.520
41.8	27.40	105.1	2.81	0.67	0.67	1.00	1.46	2.21	1.270
30.3	149.10	164.6	1.70	0.85	0.12	0.00	0.38	5.20	4.180
40.4	3.50	68.2	3.10	0.60	0.48	0.00	1.92	4.26	3.530
31.0	23.50	53.8	1.44	0.43	0.11	0.00	0.33	3.88	3.000
33.0	16.90	41.7	2.01	0.43	0.41	0.00	1.23	3.23	2.360
33.7	56.40	69.6	2.69	0.74	0.97	1.00	1.21	5.66	3.720
18.9	46.80	103.5	1.09	0.32	0.09	0.00	0.27	5.85	5.130
38.3	27.80	67.0	0.99	0.39	0.08	0.00	0.26	12.51	8.640
42.1	24.40	56.4	3.63	0.74	0.81	1.00	2.18	5.94	4.790
45.7	22.50	92.0	3.98	0.53	1.08	1.00	2.09	6.45	3.720
27.1	29.30	71.4	1.49	0.35	0.39	0.00	0.66	4.92	3.080
37.3	154.00	186.9	2.26	1.33	0.14	0.00	0.96	8.15	6.280
26.5	41.40	92.9	4.56	0.74	1.20	1.00	2.13	8.35	4.310
45.4	41.80	55.8	5.90	2.10	0.62	1.00	3.72	5.41	3.720
40.2	21.70	73.4	2.99	0.21	0.59	1.00	2.11	4.24	2.980
19.9	9.40	47.2	1.68	0.38	0.52	1.00	0.54	3.12	2.310
33.3	47.60	178.6	2.85	0.48	1.03	1.00	1.13	4.62	2.820
32.6	37.10	85.2	2.40	0.56	0.49	0.00	1.14	11.28	7.430
41.0	16.30	107.0	4.43	0.50	1.50	1.00	2.59	3.40	1.620
22.8	50.70	40.4	0.70	0.22	0.12	0.00	0.49	2.66	1.690
25.2	148.20	153.1	2.29	0.32	0.29	0.00	1.02	12.09	10.480
18.9	57.90	87.1	1.40	0.38	0.10	0.00	0.42	9.51	7.170
20.2	20.40	70.4	4.26	2.60	0.32	0.00	2.71	8.10	7.100
22.3	92.20	94.3	3.49	0.70	0.91	1.00	1.82	9.94	8.880
21.4	62.80	113.3	2.55	0.49	0.17	0.00	1.43	16.86	15.660
23.8	56.90	84.0	2.75	0.95	1.13	1.00	0.87	1.04	0.390
29.4	250.60	204.8	2.18	0.82	0.14	0.00	0.79	8.34	6.190
32.3	28.40	155.4	2.20	1.32	0.68	1.00	0.51	2.57	1.450
43.5	145.70	105.4	1.78	1.64	0.15	0.00	0.70	2.44	0.960
44.4	52.90	103.2	1.50	0.34	0.11	0.00	0.50	11.57	9.340
25.8	22.10	56.2	2.40	0.56	0.49	0.00	1.14	2.71	1.680
17.6	166.40	175.4	1.39	0.39	0.10	0.00	0.61	11.49	10.150
35.1	67.40	122.3	3.21	1.98	0.38	0.00	2.30	6.05	4.850
22.8	82.80	143.5	1.94	0.50	0.15	0.00	0.84	7.85	5.680
38.3	26.30	101.9	2.56	0.50	0.23	0.00	0.83	11.36	7.990
22.7	132.10	146.4	2.72	0.16	0.64	1.00	1.67	6.09	4.930
22.6	38.80	98.4	1.95	0.43	0.13	0.00	1.08	4.70	4.050
28.5	985.50	76.2	2.69	1.60	0.16	0.00	1.21	6.49	4.700
36.4	10.40	124.2	1.82	0.36	0.72	1.00	0.55	7.43	5.450
35.8	11.50	88.9	0.90	0.32	0.06	0.00	0.21	6.15	4.430
13.9	29.80	64.8	1.24	0.04	0.50	0.00	0.62	10.62	9.030
33.7	17.40	50.7	1.32	0.17	0.18	0.00	0.67	3.78	2.560

21.2	59.10	161.0	1.88	0.53	0.15	0.00	0.26	20.18	16.930
17.3	12.60	40.2	1.87	0.23	0.52	1.00	0.97	8.62	6.880
26.4	79.90	68.7	2.36	0.38	0.22	0.00	1.27	7.43	5.730
37.4	47.85	99.0	2.40	0.56	0.49	0.00	1.14	6.89	4.800
41.6	42.40	40.1	1.22	0.24	0.12	0.00	0.58	1.83	0.940
30.9	54.40	67.5	2.22	1.33	0.31	0.00	1.28	2.13	1.220
37.6	120.80	86.5	1.86	0.31	0.17	0.00	0.72	4.97	3.450
27.7	205.80	130.0	1.46	0.26	0.11	0.00	0.76	3.92	2.410
12.9	186.60	87.4	1.65	0.37	0.61	1.00	0.64	36.21	31.540
36.9	84.50	46.9	3.88	0.76	0.97	1.00	2.11	9.46	8.470
38.8	367.30	183.1	5.54	1.20	0.73	1.00	3.32	13.50	8.890
22.9	47.85	99.0	2.40	0.56	0.49	0.00	1.14	9.95	9.240
25.7	47.85	99.0	2.40	0.56	0.49	0.00	1.14	3.84	1.740
28.0	32.90	82.0	3.12	0.63	1.29	1.00	1.05	4.19	2.280
34.6	36.00	138.6	1.00	0.22	0.26	0.00	0.49	12.42	10.690
31.4	46.70	58.4	4.02	1.23	0.88	1.00	1.56	5.64	3.270
44.5	34.20	97.8	2.40	0.56	0.49	0.00	1.14	9.81	3.320
27.3	61.30	176.8	1.63	0.45	0.15	0.00	0.79	6.55	4.360
27.6	10.80	35.7	0.98	0.10	0.31	0.00	0.67	8.32	6.570
43.0	187.00	128.3	2.74	0.67	0.54	1.00	1.05	4.91	3.030
47.5	47.85	99.0	2.40	0.56	0.49	0.00	1.14	5.28	3.990
27.7	91.90	93.0	3.44	0.42	1.00	1.00	1.85	2.39	1.570
33.8	19.60	100.2	3.69	0.51	1.21	1.00	1.93	3.71	2.500
22.6	253.30	105.7	1.73	0.36	0.26	0.00	0.85	6.35	3.040
43.2	20.20	71.0	1.27	0.53	0.10	0.00	0.41	14.45	12.840
38.5	47.85	99.0	2.40	0.56	0.49	0.00	1.14	10.00	9.330
22.2	597.70	73.2	2.83	1.66	0.25	0.00	1.25	8.14	6.620
36.0	39.40	79.3	6.00	0.59	1.33	1.00	4.08	7.15	4.300
19.2	5.90	35.2	2.23	0.56	0.69	1.00	1.22	1.47	1.110
22.2	110.70	60.4	1.58	0.45	0.10	0.00	0.54	17.45	16.050
30.2	30.20	84.9	1.57	0.50	0.39	0.00	0.80	2.35	1.720
25.0	34.90	86.3	3.05	0.96	0.74	1.00	1.75	5.08	4.330
20.3	32.70	40.2	1.73	0.25	0.61	1.00	0.73	13.04	10.420
38.4	64.80	94.1	2.73	0.54	0.16	0.00	1.30	4.75	3.250
41.6	47.85	99.0	2.40	0.56	0.49	0.00	1.14	4.40	3.420
27.9	94.10	87.4	1.46	0.36	0.10	0.00	0.43	7.44	4.900
29.8	35.50	96.3	2.47	0.91	0.74	1.00	0.71	2.35	0.800
30.4	59.30	130.0	2.40	0.56	0.49	0.00	1.14	1.87	0.730
20.2	15.20	30.2	0.53	0.21	0.19	0.00	0.07	6.07	5.570
27.1	27.60	117.0	1.49	0.45	0.11	0.00	0.36	2.99	2.240
35.9	47.85	99.0	2.40	0.56	0.49	0.00	1.14	6.86	5.250
28.9	82.50	230.7	0.85	0.27	0.07	0.00	0.20	7.69	6.560
24.7	211.20	51.9	2.28	0.59	0.54	1.00	1.37	17.76	15.380
33.4	87.80	87.2	1.47	0.46	0.08	0.00	0.65	11.01	7.950
27.4	73.50	111.0	1.30	0.49	0.08	0.00	0.28	2.39	2.050

34.6	50.90	202.9	1.26	0.47	0.09	0.00	0.50	12.02	9.390
22.3	8.50	99.0	3.25	0.36	1.47	1.00	1.21	1.93	1.160
37.9	431.60	85.9	2.56	1.45	0.13	0.00	0.82	12.98	11.710
28.6	47.85	99.0	2.40	0.56	0.49	0.00	1.14	5.33	4.530
38.8	8.50	42.1	1.53	0.84	0.12	0.00	0.81	8.31	6.350
18.3	34.60	50.6	2.06	1.48	0.64	1.00	0.73	20.43	17.020
39.1	81.10	132.3	2.70	0.50	0.52	1.00	1.91	7.00	3.800
33.9	47.85	99.0	2.40	0.56	0.49	0.00	1.14	4.01	2.850
35.2	28.50	74.8	2.18	0.79	0.26	0.00	0.98	12.69	10.450
26.6	118.60	144.2	0.99	0.46	0.08	0.00	0.62	6.00	4.650
50.9	9.00	61.5	2.51	0.34	0.61	1.00	1.40	3.01	1.760
41.1	11.20	40.1	0.91	0.22	0.11	0.00	0.36	10.72	8.960
23.4	47.85	99.0	2.40	0.56	0.49	0.00	1.14	2.23	1.160
36.8	37.20	88.1	2.40	0.56	0.49	0.00	1.14	14.29	8.510
35.4	19.60	172.4	2.39	0.43	0.53	1.00	1.10	3.66	2.380
20.8	136.80	122.8	3.15	0.59	0.18	0.00	1.99	7.15	4.810
19.5	134.70	58.4	1.86	0.72	0.11	0.00	0.67	3.70	2.600
33.5	83.90	212.1	2.40	0.33	0.22	0.00	1.37	6.89	5.230
31.5	21.00	125.3	1.20	0.37	0.19	0.00	0.56	9.71	7.230
24.6	222.10	178.8	2.11	0.35	0.13	0.00	0.79	8.92	7.290
22.4	8.10	47.0	2.19	1.28	0.72	1.00	1.00	4.52	2.340
23.6	47.85	99.0	2.40	0.56	0.49	0.00	1.14	8.71	5.630
26.0	205.10	56.2	1.43	0.97	0.11	0.00	0.45	10.92	8.760
23.8	59.40	80.7	2.81	0.54	0.51	1.00	1.68	7.05	5.040
32.8	47.85	99.0	2.40	0.56	0.49	0.00	1.14	2.53	0.930
33.5	81.20	84.5	2.99	0.59	0.19	0.00	1.69	6.11	4.000
42.3	262.70	159.9	3.09	1.74	0.15	0.00	2.13	7.37	5.450
33.4	290.10	103.6	2.28	0.68	0.48	0.00	1.35	3.09	2.100
16.1	14.70	50.0	1.67	0.15	0.60	1.00	1.00	15.02	13.880
23.3	97.70	91.2	1.40	0.57	0.14	0.00	0.72	9.51	7.220
31.2	40.40	53.8	2.87	0.47	0.81	1.00	1.01	7.45	6.270
19.6	391.20	146.7	1.73	0.17	0.64	1.00	0.85	6.50	4.630
22.8	23.80	101.0	2.69	0.34	0.59	1.00	0.72	1.71	0.930
24.0	21.30	119.8	2.19	0.35	0.16	0.00	1.30	3.30	1.960
32.9	123.50	105.3	2.67	0.69	0.59	1.00	0.83	4.45	2.010
29.1	215.40	54.6	1.59	1.06	0.11	0.00	0.52	24.06	22.060
24.4	47.85	99.0	2.40	0.56	0.49	0.00	1.14	8.17	6.960
20.6	169.50	52.1	1.59	0.47	0.32	0.00	1.21	11.18	9.400
36.7	111.40	129.8	3.01	0.46	0.42	0.00	1.45	18.36	11.660
39.0	47.85	99.0	2.40	0.56	0.49	0.00	1.14	5.05	3.470
35.1	18.80	71.8	2.62	0.81	0.71	1.00	1.16	2.84	1.880
23.4	18.10	48.7	3.22	1.01	0.62	1.00	2.08	3.27	2.270
22.1	11.10	115.2	1.46	0.26	0.75	1.00	0.51	2.23	1.310
22.7	24.10	77.2	6.18	1.46	1.07	1.00	2.83	5.53	4.100
19.8	47.85	99.0	2.40	0.56	0.49	0.00	1.14	5.09	3.500

23.8	16.00	71.2	2.57	0.32	0.73	1.00	1.42	6.81	2.080
34.3	47.85	99.0	2.40	0.56	0.49	0.00	1.14	7.69	5.520
30.9	38.30	434.9	2.51	0.48	0.17	0.00	1.43	12.05	10.750
38.6	47.85	99.0	2.40	0.56	0.49	0.00	1.14	29.06	25.130
15.2	18.90	48.2	1.59	0.17	0.46	0.00	1.02	11.75	10.700
20.3	195.60	69.2	2.12	1.91	0.13	0.00	0.75	8.33	6.620
29.4	23.50	75.4	1.74	0.24	0.29	0.00	1.12	6.11	4.950
37.4	38.90	65.1	2.40	0.56	0.49	0.00	1.14	24.67	18.750
27.5	66.70	83.4	2.46	0.39	0.20	0.00	0.65	5.90	4.310
23.0	41.10	175.9	2.19	0.66	0.15	0.00	0.86	26.79	22.920
22.1	204.10	284.1	3.82	1.11	0.18	0.00	2.41	16.13	13.220
35.0	47.85	99.0	2.40	0.56	0.49	0.00	1.14	3.87	2.860
21.4	47.85	99.0	2.40	0.56	0.49	0.00	1.14	0.98	0.640
33.4	624.40	144.5	3.27	1.98	0.21	0.00	1.56	4.67	3.100
37.0	47.40	234.4	1.48	0.77	0.45	0.00	0.39	3.90	2.650
42.9	42.70	65.1	1.36	0.48	0.18	0.00	0.79	4.62	3.610
39.9	158.80	125.7	3.09	1.20	0.15	0.00	1.38	6.19	4.510
31.7	55.00	100.2	3.69	0.82	1.01	1.00	1.96	4.46	3.300
17.2	36.80	50.2	2.45	1.25	0.59	1.00	1.37	3.95	2.600
26.4	100.30	121.6	1.61	0.72	0.10	0.00	1.05	7.17	5.300
30.8	53.80	136.9	3.32	0.60	1.02	1.00	1.68	4.43	2.410
45.3	1474.50	859.9	11.69	1.36	0.48	0.00	10.23	6.67	4.020
31.3	190.70	121.5	2.89	2.21	0.13	0.00	1.29	9.53	6.610
24.9	14.40	62.4	1.49	0.60	0.11	0.00	0.69	19.93	15.140
28.0	57.10	123.9	3.82	0.98	0.89	1.00	1.87	2.86	1.300
15.5	16.90	67.6	2.14	0.38	0.69	1.00	1.20	4.06	2.550
22.0	47.85	99.0	2.40	0.56	0.49	0.00	1.14	13.32	12.390
17.2	102.10	118.3	1.98	0.32	0.17	0.00	1.06	13.64	11.490
25.9	25.90	33.6	2.40	0.56	0.49	0.00	1.14	2.37	2.130
51.5	35.50	68.4	1.21	0.28	0.14	0.00	0.46	2.35	1.310
33.7	47.85	99.0	2.40	0.56	0.49	0.00	1.14	9.28	7.630
22.7	483.50	151.5	5.76	1.37	0.14	0.00	4.28	10.73	6.000
27.4	9.40	63.9	1.25	0.60	0.30	0.00	0.54	4.66	3.040
30.7	80.90	98.5	2.57	0.85	0.75	1.00	1.38	3.83	2.620
33.9	47.85	99.0	2.40	0.56	0.49	0.00	1.14	17.91	16.990
26.6	28.90	73.9	2.81	0.56	0.84	1.00	1.46	3.77	2.130
41.9	47.85	99.0	2.40	0.56	0.49	0.00	1.14	14.07	10.830
23.2	13.30	51.8	1.19	0.30	0.27	0.00	0.53	4.28	3.360
44.4	36.00	98.5	5.12	0.38	1.05	1.00	3.45	5.36	2.080
15.9	4.30	44.7	1.69	0.83	0.47	0.00	0.72	18.16	14.670
18.3	47.85	99.0	2.40	0.56	0.49	0.00	1.14	5.03	3.910
29.3	44.20	180.2	2.26	0.39	0.51	1.00	0.13	6.34	4.240
46.9	157.50	160.0	5.09	2.54	0.33	0.00	3.66	16.47	14.570
37.1	18.50	152.1	2.95	0.79	0.49	0.00	1.29	6.28	2.360
30.9	7.40	53.4	1.35	0.28	0.39	0.00	0.64	4.03	2.690

21.4	41.60	91.7	1.42	0.58	0.14	0.00	0.55	7.50	5.450
16.2	165.20	52.3	1.90	0.47	0.49	0.00	0.83	8.67	5.440
39.2	28.50	128.1	3.02	0.61	0.30	0.00	1.00	4.94	2.350
32.5	14.30	53.8	2.81	1.25	0.73	1.00	1.74	4.66	1.120
34.5	48.70	127.1	4.98	0.25	0.77	1.00	1.70	9.55	7.210
22.4	58.40	117.2	1.42	0.33	0.31	0.00	0.95	9.26	7.730
20.7	8.50	92.5	2.32	0.20	1.10	1.00	0.91	2.02	0.650
16.2	11.90	187.0	1.33	0.31	0.09	0.00	0.90	4.36	3.170
24.7	111.60	131.2	2.54	0.42	0.91	1.00	1.25	4.14	3.460
29.0	30.30	130.9	1.47	0.43	0.07	0.00	0.56	4.67	3.480
29.3	49.20	130.2	1.64	0.45	0.33	0.00	1.24	9.46	8.550
33.7	66.00	249.7	2.53	0.99	0.42	0.00	2.22	17.40	14.800
60.1	97.90	106.3	4.79	0.60	0.49	0.00	1.63	13.22	9.430
27.8	85.40	122.4	1.59	0.95	0.23	0.00	0.54	11.68	11.110
12.4	17.60	38.5	1.50	0.51	0.33	0.00	0.93	17.86	12.840
29.2	13.50	60.3	2.97	0.35	1.33	1.00	1.44	10.27	9.210
27.4	9.10	43.6	2.35	0.46	0.62	1.00	1.35	2.27	1.720
33.3	62.80	97.7	1.02	0.51	0.11	0.00	0.22	7.80	6.200
31.8	47.85	99.0	2.40	0.56	0.49	0.00	1.14	12.83	10.630
23.0	11.70	37.9	0.95	0.29	0.23	0.00	0.53	5.19	4.270
17.9	130.90	84.1	2.89	0.90	0.67	1.00	1.69	11.71	9.390
19.2	20.80	93.2	2.41	0.33	0.70	1.00	1.42	4.88	2.940
35.7	39.30	165.3	1.65	0.60	0.68	1.00	0.54	6.75	4.840
31.2	16.80	58.9	1.49	0.28	0.28	0.00	0.75	1.27	0.850
31.0	111.00	163.7	2.09	0.87	0.11	0.00	0.67	11.54	9.300
26.0	56.20	151.3	1.65	0.42	0.14	0.00	0.73	9.10	5.500
12.8	47.85	99.0	2.40	0.56	0.49	0.00	1.14	10.90	9.180
21.8	9.20	45.7	0.98	0.71	0.32	0.00	0.32	1.45	1.050
21.3	15.50	50.0	2.40	0.56	0.49	0.00	1.14	0.91	0.660
35.2	47.85	99.0	2.40	0.56	0.49	0.00	1.14	6.16	3.680
45.4	27.00	109.4	2.00	0.29	0.20	0.00	0.97	6.29	4.610
46.2	65.60	103.3	1.30	0.42	0.35	0.00	0.84	13.01	12.400
29.8	23.20	92.8	0.94	0.36	0.20	0.00	0.45	5.00	4.400
34.1	7.50	77.7	1.61	0.28	0.75	1.00	0.44	2.04	1.220
27.6	12.10	122.9	1.44	0.27	0.13	0.00	0.64	4.10	3.520
27.6	19.80	82.3	4.21	0.61	1.02	1.00	2.67	12.02	10.040
49.3	26.20	103.9	3.28	0.53	1.10	1.00	1.89	3.40	1.600
32.2	16.90	118.0	1.37	0.85	0.06	0.00	0.70	14.20	11.560
27.6	79.30	90.1	1.31	0.36	0.19	0.00	0.56	3.26	2.700
29.1	199.30	67.2	1.58	1.16	0.13	0.00	0.48	13.82	11.840
30.1	155.80	54.1	1.61	0.62	0.26	0.00	0.75	13.30	9.590
27.1	53.80	117.9	2.15	8.43	0.44	0.00	0.63	5.38	4.550
18.6	48.70	88.2	1.27	0.66	0.05	0.00	0.26	3.38	2.670
46.9	45.50	69.2	1.94	0.57	0.35	0.00	1.05	2.42	1.400
30.3	62.30	153.4	1.58	0.71	0.21	0.00	0.43	8.64	3.710

26.1	39.70	77.5	2.56	1.58	0.12	0.00	1.31	5.08	3.130
41.4	20.60	67.2	3.82	0.36	1.12	1.00	2.08	5.26	2.880
44.4	173.60	116.4	4.12	1.29	0.57	1.00	1.72	6.56	3.850
37.9	73.10	81.1	2.33	0.76	0.19	0.00	1.12	8.87	5.950
33.0	295.20	101.0	2.64	1.19	0.15	0.00	1.57	11.60	9.640
23.5	76.40	101.8	2.02	0.91	0.13	0.00	0.89	3.10	1.650
26.7	108.20	143.5	2.40	0.56	0.49	0.00	1.14	5.01	3.580
21.7	56.30	60.3	2.22	0.49	0.60	1.00	1.42	5.38	3.990
46.3	588.90	231.6	2.33	1.44	0.20	0.00	1.23	3.18	2.380
17.3	21.20	102.4	1.07	0.33	0.12	0.00	0.54	3.11	1.410
33.4	267.10	77.1	2.50	1.10	0.26	0.00	1.45	3.94	2.340
24.2	131.10	154.5	2.46	1.28	0.13	0.00	1.22	5.75	3.370
33.3	45.90	125.1	3.99	0.45	0.32	0.00	2.84	2.92	2.090
45.0	83.10	206.5	3.48	1.10	0.46	0.00	1.07	8.71	7.510
52.4	11.10	64.8	1.16	0.38	0.13	0.00	0.41	6.54	4.870
23.9	112.10	126.7	2.08	0.90	0.11	0.00	0.84	10.40	7.340
23.1	41.40	83.7	2.43	0.59	0.83	1.00	1.31	6.11	4.450
29.8	28.30	124.0	3.26	0.59	1.29	1.00	1.29	3.32	1.850
13.5	17.40	88.3	2.24	1.06	0.11	0.00	1.25	2.75	1.820
28.9	123.20	133.5	2.84	1.39	0.14	0.00	1.51	5.92	4.330
37.7	331.80	162.1	4.42	1.14	1.07	1.00	2.40	3.93	2.600
22.2	135.80	121.4	1.91	0.46	0.16	0.00	1.17	3.81	2.640
34.3	34.60	29.3	1.50	0.84	0.81	1.00	0.37	5.90	5.200
41.0	33.70	181.5	2.40	0.56	0.49	0.00	1.14	2.02	1.200
44.3	47.85	99.0	2.40	0.56	0.49	0.00	1.14	17.62	14.930
20.1	109.10	99.9	1.49	0.34	0.11	0.00	0.44	11.33	9.410
31.7	54.90	48.6	1.45	0.97	0.15	0.00	0.97	6.95	5.560
32.2	57.80	63.2	2.26	0.28	0.95	1.00	1.04	4.28	3.550
24.9	68.50	73.1	3.84	0.43	0.81	1.00	2.54	11.80	10.940
30.1	166.40	144.9	2.57	1.02	0.09	0.00	1.13	9.97	8.280
25.2	33.90	58.9	2.85	0.27	0.84	1.00	1.66	6.77	5.660
21.0	84.00	55.4	2.75	4.23	0.37	0.00	0.69	4.79	2.940
22.7	75.70	182.0	1.37	0.72	0.18	0.00	0.84	5.80	3.600
49.3	1030.40	156.9	4.60	2.46	0.27	0.00	3.92	8.17	6.580
20.6	77.20	40.1	0.29	0.13	0.07	0.00	0.39	17.33	12.940
41.6	34.50	56.7	2.72	0.84	0.63	1.00	1.77	4.60	3.930
33.5	43.10	96.9	1.94	1.58	0.13	0.00	0.53	3.87	2.800
38.2	45.80	116.3	4.10	0.86	1.29	1.00	1.60	2.32	1.360
27.0	602.80	83.5	2.40	0.56	0.49	0.00	1.14	4.75	2.690
33.8	14.30	75.4	0.98	0.51	0.34	0.00	0.43	3.72	3.000
39.7	10.90	113.9	2.15	0.22	0.75	1.00	1.12	2.16	1.610
25.7	96.50	91.8	1.58	0.49	0.12	0.00	0.58	15.56	12.470
27.1	100.40	148.4	1.02	0.42	0.35	0.00	0.23	8.43	6.070
39.6	20.50	140.4	2.10	0.34	0.80	1.00	0.90	7.55	6.520
43.1	18.00	83.3	1.28	0.40	0.13	0.00	0.59	5.38	3.720

33.8	1131.40	59.6	3.77	1.75	0.15	0.00	1.97	8.39	6.170
19.6	38.60	131.8	1.48	0.47	0.12	0.00	0.20	5.08	3.520
54.4	144.40	111.0	4.33	1.27	0.32	0.00	1.38	11.27	7.750
33.6	275.60	140.7	2.91	1.28	0.55	1.00	1.82	2.62	1.200
33.9	59.90	163.7	2.04	0.89	0.10	0.00	1.05	6.92	5.070
24.2	28.10	71.7	3.08	0.57	1.20	1.00	1.57	2.49	1.110
27.6	35.70	92.6	2.73	0.82	0.78	1.00	1.71	7.70	6.500
41.4	13.40	119.8	1.37	0.33	0.10	0.00	0.59	4.35	2.520
33.0	64.60	143.1	3.07	0.59	0.15	0.00	1.73	9.61	8.320
34.8	24.50	87.1	2.10	0.49	0.92	1.00	0.87	2.82	1.700
30.8	26.10	73.0	2.93	1.36	0.36	0.00	1.84	23.00	21.900
41.2	63.40	251.7	2.40	0.56	0.49	0.00	1.14	4.10	2.500
35.2	175.00	133.5	1.65	0.64	0.11	0.00	0.58	11.77	10.340
28.8	14.70	79.6	3.23	0.40	1.17	1.00	1.40	1.36	0.960
58.1	47.85	99.0	2.40	0.56	0.49	0.00	1.14	3.96	2.360
23.1	16.90	95.8	1.16	0.30	0.34	0.00	0.59	6.15	4.190
46.7	17.40	130.1	1.28	0.45	0.61	1.00	0.35	5.55	3.960
35.9	131.80	81.1	2.53	0.90	0.31	0.00	1.37	3.12	2.000
29.6	21.60	110.0	2.47	0.26	1.24	1.00	0.97	6.42	5.780
36.8	45.20	36.2	1.73	0.53	0.27	0.00	0.65	4.80	3.330
31.2	99.30	88.4	1.78	0.37	0.12	0.00	0.97	6.58	5.230
21.9	31.00	60.3	1.64	0.90	0.12	0.00	1.09	4.21	3.460
31.3	85.00	149.6	1.21	0.30	0.12	0.00	0.55	5.13	3.240
30.2	19.80	53.3	2.85	1.47	0.51	1.00	1.48	1.60	1.100
38.1	334.90	104.2	2.44	0.88	0.23	0.00	1.08	20.15	17.230
34.8	9.20	27.9	1.26	0.37	0.13	0.00	0.58	6.57	3.580
29.0	140.90	261.9	1.77	0.40	0.14	0.00	0.99	6.87	4.660
17.2	38.00	156.9	1.93	0.35	0.38	0.00	0.13	3.62	2.830
31.4	40.50	83.4	2.75	0.54	0.59	1.00	1.54	10.04	9.100
17.3	26.90	155.6	2.02	0.48	0.16	0.00	0.93	6.76	4.360
31.5	45.10	103.3	2.93	0.44	0.19	0.00	1.77	12.04	9.510
33.4	15.40	191.2	2.17	0.43	0.93	1.00	0.82	8.16	4.050
29.8	27.80	120.1	2.84	0.32	1.05	1.00	1.15	2.54	1.400
17.3	47.85	99.0	2.40	0.56	0.49	0.00	1.14	5.37	4.450
61.1	22.90	101.1	1.25	0.38	0.26	0.00	0.59	4.50	3.400
28.6	44.30	115.2	2.01	0.80	0.34	0.00	1.39	6.90	5.000
30.3	11.40	54.3	1.73	0.38	0.13	0.00	0.35	11.14	9.280
49.2	45.60	141.9	1.30	0.19	0.13	0.00	0.62	4.67	2.900
22.2	46.40	195.8	1.81	0.53	0.15	0.00	0.76	12.55	9.030
23.0	13.90	46.4	0.88	0.40	0.07	0.00	0.15	5.91	4.230
32.3	64.60	181.8	1.23	0.60	0.09	0.00	0.25	2.70	1.690
28.5	100.10	280.2	5.17	0.37	1.31	1.00	2.59	0.78	0.510
38.5	175.20	107.3	2.13	0.50	0.11	0.00	0.97	4.47	3.160
21.8	52.30	128.4	1.45	0.38	0.46	0.00	0.90	3.82	2.700
31.5	26.50	52.5	3.44	0.48	1.82	1.00	0.65	12.21	8.800

27.6	20.60	200.6	1.01	0.33	0.08	0.00	0.31	4.14	2.480
20.9	38.60	66.2	2.39	1.06	0.64	1.00	1.28	7.76	5.960
19.9	4.70	45.6	1.31	0.51	0.25	0.00	0.35	6.93	5.990
43.0	10.40	82.8	2.07	0.43	0.65	1.00	1.16	1.38	0.580
17.1	102.00	78.6	2.13	1.91	0.36	0.00	0.67	12.21	9.900
12.5	47.85	99.0	2.40	0.56	0.49	0.00	1.14	14.81	12.940
21.1	3.00	41.5	2.20	0.20	0.63	1.00	1.38	2.22	1.250
44.3	20.40	153.7	2.57	0.34	0.33	0.00	1.03	10.80	8.410
34.2	44.80	71.6	3.98	0.65	0.70	1.00	2.73	6.28	5.090
29.1	20.70	98.8	3.46	0.51	0.60	1.00	2.27	4.10	2.620
25.4	21.90	113.1	4.17	0.42	1.35	1.00	2.03	2.40	2.140
27.3	10.50	122.2	1.48	0.38	0.80	1.00	0.79	2.12	1.200
36.3	47.85	99.0	2.40	0.56	0.49	0.00	1.14	2.67	1.790
35.8	58.50	199.3	6.50	0.73	1.29	1.00	3.23	3.82	2.550
32.0	192.10	228.6	3.76	1.03	1.71	1.00	1.15	5.80	4.600
33.9	57.00	113.1	2.71	0.33	1.27	1.00	1.02	2.97	2.230
41.2	26.60	79.4	1.11	0.43	0.17	0.00	0.22	4.37	3.680
38.0	46.90	179.8	1.67	0.73	0.09	0.00	0.55	4.02	2.540
34.2	14.80	146.3	4.58	1.14	1.00	1.00	1.83	4.11	2.330
36.8	33.30	140.6	3.71	1.31	1.08	1.00	1.57	3.12	1.580
50.3	121.80	188.3	2.40	0.94	0.13	0.00	1.07	8.40	6.900
27.5	86.40	234.6	1.96	0.96	0.11	0.00	0.59	4.31	3.340
25.3	164.20	88.1	4.77	0.26	1.85	1.00	0.98	6.28	2.690
24.1	65.70	111.6	2.31	0.47	0.49	0.00	1.46	6.84	4.100
30.7	47.85	99.0	2.40	0.56	0.49	0.00	1.14	4.46	1.200
37.6	109.90	686.3	3.63	1.29	0.39	0.00	3.41	4.10	2.800
25.0	141.30	146.8	1.58	0.54	0.52	1.00	1.03	2.80	1.400
35.4	17.60	120.1	0.88	0.27	0.26	0.00	0.38	3.96	2.940
43.4	98.60	76.7	2.38	0.48	0.16	0.00	1.27	4.61	2.700
27.3	269.60	232.2	2.32	0.74	0.17	0.00	0.86	3.37	2.020
24.8	123.80	93.9	2.78	1.30	0.16	0.00	1.37	6.61	4.890
38.7	21.60	102.5	2.73	0.43	0.26	0.00	1.30	5.52	2.880
35.7	88.40	114.9	2.06	0.38	0.44	0.00	1.10	7.40	5.300
30.3	53.20	104.0	2.89	0.55	0.87	1.00	1.16	3.74	1.430
46.8	137.90	105.2	2.23	0.53	0.56	1.00	1.35	7.50	6.600
33.8	44.20	116.7	2.96	0.39	0.22	0.00	1.62	4.91	1.580
28.9	47.85	99.0	2.40	0.56	0.49	0.00	1.14	9.83	7.460
29.2	60.90	109.6	2.96	0.92	0.78	1.00	1.54	3.79	2.970
34.3	15.70	181.9	4.43	0.59	1.57	1.00	1.79	3.60	4.650
39.1	20.60	175.8	1.40	0.46	0.27	0.00	0.90	8.70	7.000
17.4	15.90	33.3	1.58	0.46	0.45	0.00	0.77	7.31	5.710
45.9	63.30	143.5	3.56	0.51	1.29	1.00	1.71	8.79	4.360
29.2	79.00	159.9	3.74	0.75	0.92	1.00	2.35	8.46	7.060
43.8	210.90	131.3	3.44	0.67	0.63	1.00	1.87	2.91	1.310
35.9	37.00	63.8	2.26	0.67	0.57	1.00	1.02	13.24	12.300

35.4	60.50	94.0	2.03	0.42	0.11	0.00	0.78	6.86	4.527
38.9	47.85	99.0	2.40	0.56	0.49	0.00	1.14	4.13	2.690
28.8	34.40	80.2	2.79	1.15	0.69	1.00	1.50	1.88	1.080
22.6	17.00	96.9	2.79	0.65	0.70	1.00	1.25	4.56	3.360
40.0	92.10	153.4	4.21	0.66	1.42	1.00	2.24	2.52	1.900
28.9	12.90	56.1	1.28	0.36	0.27	0.00	0.69	3.17	1.760
19.5	201.30	146.7	2.29	0.61	0.53	1.00	1.12	3.11	1.990
46.1	52.30	70.0	2.78	0.50	1.15	1.00	1.34	4.30	2.700
26.9	47.85	99.0	2.40	0.56	0.49	0.00	1.14	2.66	2.250
37.6	17.80	78.4	1.04	0.27	0.30	0.00	0.32	3.14	1.720
34.9	23.30	230.1	2.69	0.55	0.97	1.00	1.19	3.38	1.890
20.2	17.60	54.6	1.36	0.03	0.37	0.00	1.21	3.53	2.700
22.3	17.40	65.7	1.84	0.32	0.48	0.00	1.29	2.00	1.300
44.1	70.90	119.2	3.33	0.73	0.26	0.00	2.30	5.50	3.430
23.3	272.60	55.5	2.57	1.34	0.68	1.00	1.37	7.55	6.190
23.3	272.60	55.5	2.57	1.34	0.68	1.00	1.37	5.63	4.090
31.9	47.85	99.0	2.40	0.56	0.49	0.00	1.14	5.63	4.090
27.5	47.85	99.0	2.40	0.56	0.49	0.00	1.14	2.57	1.810
31.9	45.10	30.1	1.57	0.46	0.10	0.00	0.55	4.87	4.270
44.1	39.90	55.4	3.40	0.55	1.18	1.00	1.62	6.97	1.720
47.0	77.10	97.0	2.99	1.13	0.33	0.00	2.25	3.43	2.320
27.7	36.00	178.9	3.89	0.54	0.86	1.00	1.70	10.13	8.260
38.9	131.60	162.4	4.78	1.40	0.36	0.00	2.92	3.13	2.040
26.1	71.90	191.0	4.31	0.52	0.83	1.00	2.42	3.06	2.130
26.9	33.70	104.9	2.00	0.35	1.01	1.00	0.40	2.87	1.680
26.3	348.60	97.8	4.90	0.83	1.05	1.00	3.09	6.79	4.840
36.0	66.50	78.9	3.17	0.73	0.18	0.00	1.83	5.45	3.480
49.6	47.85	99.0	2.40	0.56	0.49	0.00	1.14	3.93	3.010
36.5	114.70	109.2	3.11	1.25	1.17	1.00	1.36	4.91	3.200
51.4	164.50	225.3	3.77	0.35	0.80	1.00	2.43	4.61	3.750
23.6	532.20	94.6	2.53	0.62	0.53	1.00	1.70	3.91	2.890
38.4	15.00	63.4	1.60	0.46	0.21	0.00	1.20	8.91	6.750
23.0	10.00	59.3	2.40	0.56	0.49	0.00	1.14	2.19	1.400
53.2	87.40	125.6	1.56	0.65	0.09	0.00	0.81	13.61	9.900
27.5	17.40	72.8	2.14	0.37	0.70	1.00	0.88	7.30	5.400
34.8	54.40	140.0	2.05	0.80	0.52	1.00	0.68	6.30	4.600
23.5	84.10	166.7	2.97	0.55	0.12	0.00	1.42	3.39	2.470
25.6	93.40	86.8	4.07	0.47	1.79	1.00	1.78	7.79	3.230
31.3	221.80	91.6	2.32	0.71	0.15	0.00	1.15	13.66	10.520
33.0	23.70	98.2	2.54	0.31	0.94	1.00	1.13	5.13	3.570
23.5	57.30	90.4	1.46	0.12	0.67	1.00	0.81	3.61	2.480
37.3	58.40	207.7	2.58	1.17	0.10	0.00	1.57	4.14	2.830
26.9	174.20	76.5	4.57	0.92	0.68	1.00	3.41	4.01	2.480
32.2	64.10	85.6	2.01	0.66	0.15	0.00	0.91	6.58	4.880
39.8	21.10	95.8	2.18	0.32	0.63	1.00	1.26	8.40	7.050

53.8	47.85	99.0	2.40	0.56	0.49	0.00	1.14	7.09	5.350
21.9	16.80	106.0	4.20	0.68	0.40	0.00	2.35	5.66	3.480
28.2	16.70	65.9	1.40	0.45	0.12	0.00	0.54	2.81	1.690
24.6	57.60	135.6	2.73	0.50	0.88	1.00	1.52	4.49	3.410
41.3	49.80	113.4	1.91	0.29	0.19	0.00	1.09	9.78	7.620
20.7	43.30	58.8	2.08	1.32	0.53	1.00	1.02	8.89	6.710
40.1	23.10	173.7	1.85	0.87	0.23	0.00	0.84	7.40	5.000
36.2	53.60	58.5	3.02	0.64	0.24	0.00	2.20	5.18	4.140
16.5	16.50	38.0	1.38	0.59	0.36	0.00	1.01	22.96	19.080
27.9	64.60	112.9	1.52	0.39	0.12	0.00	0.65	18.55	16.680
35.1	65.50	140.9	2.57	0.22	1.41	1.00	0.63	3.15	1.980
26.4	69.80	85.6	3.35	0.41	0.90	1.00	1.88	5.31	4.190
21.0	39.50	105.1	2.15	0.10	0.75	1.00	1.03	4.56	3.750
26.3	58.30	100.9	2.99	0.44	0.22	0.00	1.50	3.09	2.080
63.8	30.20	83.3	1.94	0.22	0.65	1.00	1.26	3.08	2.210
38.9	242.80	146.1	1.40	0.38	0.43	0.00	0.80	9.90	6.600
19.9	55.10	47.6	2.08	1.02	0.51	1.00	1.04	6.78	5.290
18.0	5.70	66.9	2.40	0.56	0.49	0.00	1.14	3.46	1.610
41.3	374.70	132.9	4.58	3.29	0.64	1.00	2.56	8.41	6.210
44.1	280.70	115.0	1.58	1.10	0.17	0.00	0.80	14.41	11.620
42.2	7.60	93.0	2.40	0.56	0.49	0.00	1.14	5.60	2.530
38.8	78.30	132.4	4.18	0.39	1.20	1.00	2.39	4.10	3.120
26.2	45.50	64.9	1.43	0.11	0.44	0.00	0.74	10.76	8.990
28.6	97.60	117.4	2.43	0.57	0.54	1.00	1.11	7.08	6.640
24.4	27.80	205.1	2.45	1.14	0.36	0.00	1.15	4.11	2.600
35.8	15.00	69.2	2.76	0.40	1.12	1.00	1.17	1.99	1.370
40.6	14.70	54.2	1.16	0.27	0.27	0.00	0.49	5.60	4.800
41.4	44.10	90.4	3.64	0.53	0.86	1.00	2.00	4.75	3.070
33.9	21.90	51.7	1.49	0.47	0.09	0.00	0.48	5.93	5.270
33.5	76.60	64.2	1.45	0.61	0.54	1.00	0.53	10.00	8.800
47.9	76.10	99.2	2.77	1.19	0.12	0.00	1.60	3.98	2.220
49.4	213.60	61.5	2.52	1.09	0.61	1.00	1.44	4.54	3.020
18.6	20.60	44.3	4.02	0.41	0.51	1.00	1.91	3.06	2.240
28.4	140.70	124.4	3.35	2.14	0.81	1.00	1.92	6.85	2.660
33.7	70.70	128.1	6.32	0.38	1.64	1.00	3.45	9.68	8.110
38.1	13.20	62.5	2.40	0.56	0.49	0.00	1.14	5.97	4.400
53.5	65.00	78.1	2.64	1.57	0.30	0.00	1.88	3.66	1.920
22.3	59.00	183.6	2.75	0.37	0.48	0.00	1.46	15.11	13.270
30.3	27.90	111.4	2.40	0.56	0.49	0.00	1.14	7.47	5.360
35.5	18.20	102.4	2.05	0.46	1.06	1.00	0.79	2.90	1.800
29.3	22.20	66.3	2.67	0.43	1.10	1.00	1.13	3.15	2.190
32.6	669.20	112.6	1.72	2.72	1.12	1.00	0.23	4.11	2.290
34.9	46.10	112.4	2.14	0.40	0.78	1.00	1.12	3.36	2.040
23.3	99.90	51.5	2.05	0.43	0.53	1.00	1.47	4.80	2.000
32.1	62.90	71.1	3.01	0.19	0.70	1.00	2.01	10.82	8.210

38.0	66.70	153.0	5.41	0.89	1.10	1.00	2.63	5.05	4.140
28.3	65.20	67.1	3.54	0.62	1.06	1.00	1.71	3.37	1.850
25.4	19.30	130.2	3.11	0.48	0.94	1.00	1.38	2.44	1.650
45.7	70.50	181.3	3.84	1.83	0.48	0.00	1.93	6.35	4.990
26.2	47.85	99.0	2.40	0.56	0.49	0.00	1.14	4.77	3.600
27.0	166.20	166.0	3.09	0.55	1.55	1.00	1.35	2.94	1.770
37.8	63.30	98.8	2.55	0.80	0.62	1.00	1.14	3.35	1.050
36.9	120.30	79.7	5.62	1.30	1.28	1.00	2.94	4.16	1.850
27.1	47.85	99.0	2.40	0.56	0.49	0.00	1.14	4.83	3.260
31.4	58.90	144.1	2.62	1.51	0.12	0.00	1.29	6.36	4.390
11.2	15.40	45.2	1.87	0.46	0.48	0.00	0.89	5.57	4.910
23.4	17.50	42.9	2.54	1.23	0.42	0.00	1.45	4.63	3.210
28.7	63.00	57.3	2.61	0.93	0.64	1.00	0.95	7.49	6.930
44.3	47.85	99.0	2.40	0.56	0.49	0.00	1.14	2.14	1.460
31.7	120.20	151.7	3.78	2.82	0.32	0.00	1.76	2.97	1.110
23.9	78.10	76.2	3.31	0.35	1.34	1.00	1.21	3.09	2.370
27.7	48.20	107.3	2.37	0.38	0.73	1.00	1.43	7.76	5.360
37.2	47.30	132.7	3.25	0.41	1.08	1.00	1.64	6.83	5.490
15.4	3.20	27.2	1.44	1.71	0.30	0.00	0.52	1.86	1.450
32.7	21.60	136.4	2.49	0.47	0.99	1.00	1.08	5.70	4.500
36.1	18.30	51.0	3.56	0.96	1.03	1.00	1.82	5.82	4.400
39.5	35.80	262.2	2.19	0.51	0.60	1.00	1.11	3.60	1.900
67.1	102.20	102.2	2.13	0.52	0.24	0.00	1.27	13.79	11.810
20.4	37.20	94.3	3.47	0.73	1.13	1.00	1.52	2.90	1.670
26.2	9.20	35.1	1.39	0.47	0.31	0.00	0.79	14.37	12.780
22.2	17.00	43.1	2.61	0.38	0.53	1.00	1.70	6.06	3.890
38.5	27.80	151.6	1.73	0.38	0.68	1.00	0.57	3.31	2.110
32.4	15.40	70.5	3.12	1.25	0.61	1.00	1.72	3.01	2.360
23.4	67.00	187.9	1.66	0.53	0.10	0.00	0.56	8.42	5.080
24.5	82.90	69.7	3.59	0.24	1.44	1.00	1.83	3.43	2.620
22.5	15.50	47.7	4.63	0.17	1.26	1.00	2.76	3.39	1.920
30.9	335.20	74.7	3.66	0.78	1.66	1.00	2.17	3.52	2.500
23.1	15.90	96.0	2.40	0.56	0.49	0.00	1.14	2.00	1.100
28.8	25.00	128.7	3.70	0.83	1.35	1.00	1.85	3.90	2.900
44.3	1.90	110.9	3.36	0.69	1.57	1.00	1.35	9.60	8.600
63.9	28.40	66.5	1.66	0.90	0.43	0.00	0.83	2.35	1.330
25.6	45.70	93.8	3.04	0.41	1.23	1.00	1.04	3.13	2.050
17.8	17.80	49.0	2.11	0.35	0.77	1.00	1.19	4.97	4.280
27.4	22.20	137.6	1.13	0.36	0.69	1.00	0.44	2.38	1.750
29.9	67.80	138.6	3.35	0.62	1.01	1.00	2.02	4.30	2.200
32.9	1466.50	74.4	7.00	2.40	0.41	0.00	5.36	3.61	2.070
17.6	11.60	52.4	2.45	0.64	0.77	1.00	1.32	1.63	0.990
24.7	47.85	99.0	2.40	0.56	0.49	0.00	1.14	1.88	0.990
13.5	27.50	62.9	2.12	1.45	0.41	0.00	0.89	9.10	7.140
33.4	54.00	59.7	3.39	0.97	0.79	1.00	1.41	2.99	1.840

31.7	51.90	80.0	2.27	0.36	0.28	0.00	1.36	7.34	6.100
41.6	103.80	60.9	5.71	0.99	0.82	1.00	3.23	6.97	5.490
19.1	42.40	52.4	1.29	0.81	0.60	1.00	0.56	8.79	6.080
41.7	42.20	212.8	3.39	0.54	1.63	1.00	1.88	4.30	2.000
37.1	85.80	132.4	3.18	0.30	0.41	0.00	2.45	4.74	2.860
18.3	13.00	81.6	2.52	0.97	0.57	1.00	1.29	2.20	1.300
38.4	234.20	110.3	2.32	0.50	0.94	1.00	0.89	3.94	2.860
23.6	11.50	68.8	3.30	0.52	1.17	1.00	1.64	1.96	1.180
18.6	16.80	42.4	2.43	0.77	0.51	1.00	1.38	14.41	13.000
44.5	40.40	212.8	4.48	1.02	0.36	0.00	2.69	5.30	3.400
32.1	105.40	128.9	3.11	0.96	0.65	1.00	1.67	3.07	1.280
32.7	16.10	45.2	3.96	0.37	1.32	1.00	2.09	2.46	1.510
31.7	266.70	98.3	3.22	1.83	0.12	0.00	2.03	6.25	5.430
23.8	47.85	99.0	2.40	0.56	0.49	0.00	1.14	2.67	2.040
30.3	58.50	126.9	2.65	1.07	0.14	0.00	1.55	7.25	4.690
26.1	19.40	45.6	2.38	0.44	0.69	1.00	1.29	2.51	1.780
28.5	5.80	40.2	2.60	0.25	0.95	1.00	1.08	4.87	2.460
23.4	18.00	95.6	2.00	0.26	0.80	1.00	0.80	1.48	1.080
36.8	24.60	41.6	1.85	0.28	0.57	1.00	0.48	16.27	13.510
21.2	16.50	61.6	3.00	0.66	0.85	1.00	1.59	4.40	3.080
31.6	47.85	99.0	2.40	0.56	0.49	0.00	1.14	1.90	1.460
12.3	108.70	49.1	1.43	0.33	0.28	0.00	0.96	3.22	2.290
18.9	113.40	123.7	1.18	0.34	0.08	0.00	0.39	3.07	2.010
29.6	29.20	115.2	3.40	0.68	0.54	1.00	1.57	3.01	1.990
27.2	22.90	92.5	3.72	0.59	0.95	1.00	1.77	4.32	3.440
20.9	51.50	41.4	2.75	1.26	0.51	1.00	1.24	18.33	13.060
32.5	43.90	139.3	3.65	0.55	1.43	1.00	1.60	3.14	2.400
24.8	44.60	175.8	2.27	0.91	0.29	0.00	1.12	2.88	1.440
20.6	15.50	41.3	1.56	0.46	0.57	1.00	0.63	9.42	8.230
31.4	17.10	86.7	2.45	0.40	0.71	1.00	1.16	7.49	5.540
22.7	21.70	73.2	2.66	0.40	0.52	1.00	1.38	9.38	7.700
17.3	15.50	42.0	3.06	0.80	0.88	1.00	1.67	7.91	6.700
34.3	29.70	105.3	3.45	0.73	0.68	1.00	1.30	3.17	1.880
23.5	8.70	54.3	3.56	0.84	0.98	1.00	1.96	1.38	0.830
19.0	30.90	217.6	3.11	0.76	0.59	1.00	1.53	3.09	1.830
26.0	571.80	152.7	1.27	0.37	0.13	0.00	0.54	8.87	6.330
28.1	10.70	112.6	2.55	0.53	0.98	1.00	0.94	1.80	0.770
36.3	37.50	133.4	3.48	0.93	0.32	0.00	2.12	7.41	6.060
26.6	69.70	160.2	2.82	0.42	0.74	1.00	1.41	10.07	8.260
16.6	10.70	41.6	2.17	0.83	0.33	0.00	1.50	4.13	2.490
29.3	27.20	143.6	3.09	0.47	0.71	1.00	1.86	5.54	4.570
20.8	22.40	68.2	3.00	1.02	1.27	1.00	0.51	7.33	6.230
25.4	107.40	114.6	2.99	0.58	0.76	1.00	1.29	2.65	1.400
24.8	40.30	85.8	3.25	0.53	1.00	1.00	1.66	7.70	5.300
26.1	11.40	57.4	3.05	0.34	0.87	1.00	2.01	6.44	2.960

20.7	47.85	99.0	2.40	0.56	0.49	0.00	1.14	5.82	4.420
15.4	9.90	39.6	1.46	0.60	0.42	0.00	0.57	9.42	7.000
37.7	83.90	120.9	2.57	0.49	0.15	0.00	1.53	10.96	8.280
17.2	20.00	47.4	1.22	0.97	0.34	0.00	0.48	7.96	6.570
38.1	18.20	37.7	2.40	0.56	0.49	0.00	1.14	7.21	6.070
32.4	27.40	88.6	2.01	0.45	0.25	0.00	0.92	3.51	1.600
39.2	832.30	123.6	3.14	3.29	0.12	0.00	1.09	4.84	3.630
21.8	27.90	50.1	2.74	0.78	0.78	1.00	1.45	7.95	5.760
16.0	44.00	44.9	2.10	0.40	0.30	0.00	1.01	4.72	2.880
44.6	40.90	72.9	3.84	0.61	1.01	1.00	2.20	6.98	4.410
24.3	360.00	55.8	0.70	1.48	1.01	1.00	1.64	4.93	3.290
30.3	34.90	91.2	3.67	0.73	0.42	0.00	2.27	5.75	4.100
18.1	21.20	57.8	2.50	1.23	0.56	1.00	1.45	10.94	8.110
42.7	37.40	119.8	2.25	0.64	0.29	0.00	1.14	3.14	2.040
21.4	14.30	47.2	3.30	1.61	0.84	1.00	1.55	2.11	1.710
29.8	32.70	114.4	3.81	0.36	1.04	1.00	1.90	2.70	1.350
28.8	23.60	89.5	3.44	0.46	1.47	1.00	1.18	2.49	1.320
20.3	424.20	117.9	2.10	0.60	0.44	0.00	0.93	8.79	8.380
40.5	43.70	134.4	3.08	0.63	0.45	0.00	2.06	8.40	6.600
47.9	30.90	93.0	2.73	0.45	0.93	1.00	1.17	6.89	4.780
26.4	44.90	104.8	4.43	0.48	0.89	1.00	2.92	5.14	3.700
27.3	26.00	102.8	2.53	0.40	0.93	1.00	0.89	3.13	1.650
51.4	47.85	99.0	2.40	0.56	0.49	0.00	1.14	4.50	3.700
25.7	31.20	82.7	2.37	0.91	0.66	1.00	1.30	2.34	1.880
41.4	22.80	92.5	1.73	0.34	0.56	1.00	0.80	4.35	2.720
27.8	24.00	174.3	2.72	0.29	0.71	1.00	0.88	5.43	3.490
33.9	25.40	133.0	3.25	0.57	1.43	1.00	1.21	4.00	1.360
30.3	211.20	175.2	3.68	0.80	1.34	1.00	2.00	4.20	2.100
28.0	24.50	54.7	4.54	0.37	1.23	1.00	2.79	2.87	2.090
33.0	38.00	115.8	4.66	0.43	0.98	1.00	2.82	4.02	2.290
27.1	47.85	99.0	2.40	0.56	0.49	0.00	1.14	5.83	4.050
20.2	61.90	87.0	2.02	0.62	0.88	1.00	0.97	19.91	18.100
33.1	17.90	77.5	1.75	0.51	0.75	1.00	0.62	2.58	1.460
23.4	8.40	70.2	2.40	0.56	0.49	0.00	1.14	6.08	5.490
25.1	22.20	41.7	2.04	0.43	1.02	1.00	0.81	1.56	0.940
34.2	47.85	99.0	2.40	0.56	0.49	0.00	1.14	4.48	3.190
38.3	253.20	154.1	3.53	1.11	0.63	1.00	2.13	4.10	2.110
25.2	114.10	103.6	4.50	1.85	0.66	1.00	3.07	10.07	8.460
34.8	24.50	67.0	3.27	0.50	1.33	1.00	1.76	3.36	2.160
32.8	47.85	99.0	2.40	0.56	0.49	0.00	1.14	1.82	1.000
35.0	457.80	267.3	3.32	0.65	0.98	1.00	1.69	3.72	2.800
22.8	40.80	70.3	3.56	0.60	1.07	1.00	1.92	14.27	12.080
32.0	120.20	59.7	4.02	0.60	1.42	1.00	2.05	2.41	1.430
30.0	16.50	101.9	2.44	0.44	0.86	1.00	1.18	4.32	2.810
17.2	8.80	53.2	2.30	1.63	0.62	1.00	1.01	3.78	2.850

39.4	136.50	97.3	1.83	0.60	0.13	0.00	0.98	5.03	1.320
28.3	115.40	203.3	3.32	1.08	0.37	0.00	1.53	7.39	4.690
38.3	29.30	95.9	2.34	0.40	1.04	1.00	1.04	4.53	2.330
23.0	8.50	63.3	3.38	0.46	0.79	1.00	2.16	5.26	3.050
35.8	97.10	171.1	3.41	0.30	0.60	1.00	2.37	6.06	2.230
34.5	12.90	86.6	3.42	0.41	1.09	1.00	1.84	2.23	1.350
39.7	23.20	129.7	3.56	0.37	0.63	1.00	1.29	7.17	6.710
34.0	70.60	133.2	3.91	0.99	1.60	1.00	1.73	2.45	1.300
37.1	59.00	101.5	2.39	1.00	0.79	1.00	0.97	3.60	1.900
20.5	20.80	72.1	3.96	0.11	0.99	1.00	1.52	13.23	11.870
33.8	18.30	92.2	2.85	0.41	0.37	0.00	1.47	1.77	0.750
23.5	76.60	117.0	3.43	1.57	0.13	0.00	1.58	5.64	3.650
25.6	121.60	71.2	2.40	0.56	0.49	0.00	1.14	4.37	2.960
30.9	24.90	79.9	1.71	0.92	0.45	0.00	0.77	8.16	5.390
38.3	199.50	130.1	3.52	0.57	0.77	1.00	2.11	5.34	2.800
46.6	34.10	65.4	2.55	0.55	1.16	1.00	1.02	3.32	2.000
28.7	98.40	272.4	2.90	1.13	0.16	0.00	1.38	9.45	5.800
56.0	47.85	99.0	2.40	0.56	0.49	0.00	1.14	5.37	2.030
38.7	12.40	60.6	2.10	0.35	0.61	1.00	1.01	3.94	2.390
17.6	16.90	70.2	2.99	0.89	1.03	1.00	1.45	2.52	1.480
29.2	47.85	99.0	2.40	0.56	0.49	0.00	1.14	6.36	4.660
38.1	25.60	152.5	2.26	0.46	0.71	1.00	0.88	3.22	2.300
37.6	25.40	94.6	3.10	0.56	0.74	1.00	1.39	10.63	7.400
42.7	79.40	204.3	3.67	0.54	1.04	1.00	1.97	4.19	1.220
37.0	454.00	217.3	3.80	1.57	1.32	1.00	1.40	3.32	2.200
17.7	11.20	51.7	2.34	0.56	0.64	1.00	1.36	1.68	1.270
25.1	126.80	160.9	1.93	0.50	0.12	0.00	0.74	14.76	12.960
41.1	36.10	127.5	2.85	0.41	1.20	1.00	1.78	3.43	2.320
35.0	753.70	74.9	4.09	0.90	1.45	1.00	2.21	7.69	6.090
42.6	71.40	73.4	3.36	0.53	0.94	1.00	1.73	5.88	2.870
38.9	150.50	125.8	2.51	1.33	0.32	0.00	0.68	4.95	3.330
35.3	456.30	120.5	2.78	0.71	0.70	1.00	1.80	4.83	2.640
30.3	15.80	162.0	3.03	0.28	1.18	1.00	1.31	2.29	1.820
44.0	66.60	201.0	1.91	0.50	0.34	0.00	1.39	5.60	4.140
24.8	27.60	146.2	3.74	0.61	1.43	1.00	1.57	2.64	1.390
35.2	63.60	36.0	2.29	0.78	0.30	0.00	1.53	10.97	9.550
21.5	86.80	62.7	3.34	1.29	0.76	1.00	2.04	3.69	2.410
26.6	787.20	84.5	4.04	1.70	0.85	1.00	2.73	5.50	3.440
19.3	44.00	50.3	3.32	0.66	0.77	1.00	2.09	9.75	6.660
26.8	47.85	99.0	2.40	0.56	0.49	0.00	1.14	3.55	2.450
28.7	93.30	120.4	5.89	1.10	0.70	1.00	3.08	5.09	2.150
33.6	29.20	122.5	2.22	0.49	0.78	1.00	1.01	3.06	2.250
39.3	9.00	57.6	2.15	0.70	0.40	0.00	1.41	2.47	1.900
25.7	15.80	65.3	2.20	0.40	0.73	1.00	1.12	7.94	5.750
30.1	238.60	103.2	1.62	0.47	0.42	0.00	1.08	7.18	5.250

28.0	17.70	112.7	2.54	0.52	0.30	0.00	1.17	2.19	1.180
18.2	10.00	47.5	1.58	0.47	0.33	0.00	0.86	3.00	1.570
39.6	58.20	87.6	2.40	0.56	0.49	0.00	1.14	5.06	3.210
15.1	26.50	39.7	1.73	0.49	0.49	0.00	1.05	7.21	5.290
37.4	21.50	104.7	2.51	0.56	0.50	0.00	0.92	9.97	8.030
31.4	28.50	101.3	3.05	0.36	1.16	1.00	1.28	2.84	1.600
32.4	47.85	99.0	2.40	0.56	0.49	0.00	1.14	4.05	3.110
26.7	51.50	107.6	2.79	0.48	1.29	1.00	1.32	1.64	1.080
28.6	16.40	156.0	2.07	0.38	0.78	1.00	1.36	2.62	1.800
20.7	50.30	81.1	2.66	0.95	0.87	1.00	1.15	6.99	5.310
36.3	209.30	252.2	5.05	2.25	0.16	0.00	3.27	4.17	2.360
37.4	44.80	102.5	3.72	0.69	1.11	1.00	1.87	5.34	3.230
32.5	8.70	66.6	1.67	0.05	0.83	1.00	0.91	6.84	5.860
21.6	87.00	67.3	1.28	0.15	0.62	1.00	0.39	4.32	3.180
25.1	33.80	114.7	3.32	0.53	1.05	1.00	2.25	13.40	9.500
41.0	86.40	87.8	4.05	1.63	0.82	1.00	2.35	4.94	3.370
44.3	987.80	71.4	7.38	1.88	0.82	1.00	5.62	5.80	4.400
18.3	27.80	119.5	3.33	0.36	1.24	1.00	1.32	9.12	6.140
20.2	56.80	87.2	2.97	0.71	1.03	1.00	1.33	2.84	1.700
28.3	7.20	144.9	2.49	0.41	1.06	1.00	0.99	2.00	1.020
27.7	262.80	115.8	2.76	0.67	0.82	1.00	1.34	3.80	2.910
50.9	31.60	87.1	3.23	0.63	1.21	1.00	1.50	3.32	2.700
24.5	198.10	52.8	2.37	0.99	0.40	0.00	1.09	9.93	8.490
22.7	11.50	54.3	2.25	0.46	0.33	0.00	1.47	3.33	2.250
33.6	12.40	100.8	2.62	0.37	1.19	1.00	1.04	1.90	1.560
13.2	6.30	39.9	1.11	0.06	0.65	1.00	0.51	15.22	13.220
35.5	85.60	109.4	4.03	0.70	1.09	1.00	2.46	2.90	2.070
29.0	73.30	168.5	6.47	0.76	1.35	1.00	2.19	5.68	3.150
28.9	129.20	73.5	3.44	1.19	0.39	0.00	2.07	15.75	12.950
30.3	14.60	11.6	1.94	1.07	0.35	0.00	0.43	7.33	6.350
17.2	16.20	36.5	1.69	0.81	0.69	1.00	0.56	5.46	4.450
33.1	18.20	109.0	1.86	0.30	0.39	0.00	1.72	3.55	2.520
34.1	500.20	114.5	3.15	1.40	0.47	0.00	1.65	7.66	6.210
33.7	115.60	122.5	2.40	0.56	0.49	0.00	1.14	11.92	7.790
47.7	72.90	187.8	2.93	0.81	0.45	0.00	1.22	8.48	4.740
37.5	149.60	328.7	2.80	0.17	0.62	1.00	2.00	4.50	3.300
22.0	29.60	43.6	2.79	1.12	0.85	1.00	1.33	3.74	2.810
30.8	36.20	74.9	2.15	0.39	1.09	1.00	0.78	5.63	4.210
18.7	11.20	76.3	2.11	0.39	1.10	1.00	0.83	4.50	3.300
17.4	19.80	49.8	1.50	0.44	0.33	0.00	0.93	5.40	4.640
27.6	129.40	118.8	2.38	0.98	0.39	0.00	0.97	9.69	8.940
33.6	35.50	74.7	3.47	0.62	0.41	0.00	1.15	5.57	4.390
27.9	33.40	113.8	3.13	0.41	1.09	1.00	1.36	3.99	2.900
23.0	8.50	54.1	2.42	1.32	0.68	1.00	1.07	8.16	7.090
45.2	24.30	54.6	1.72	0.53	0.18	0.00	0.92	7.00	5.530

23.6	18.60	83.3	2.72	0.48	0.66	1.00	1.82	4.01	2.160
32.5	40.30	102.2	2.79	0.71	0.68	1.00	1.49	18.57	17.340
25.9	78.70	98.2	2.09	0.56	0.66	1.00	1.08	9.70	7.400
35.2	30.80	79.0	3.33	0.55	0.86	1.00	1.46	7.21	5.640
26.6	8.90	41.8	1.93	0.51	0.91	1.00	0.69	2.40	1.640
45.5	64.20	99.4	3.32	0.63	1.07	1.00	1.59	4.70	3.060
15.9	392.30	68.3	2.20	1.07	0.32	0.00	0.87	6.20	4.390
32.3	180.63	192.5	2.49	0.89	0.42	0.00	1.24	10.20	8.600
27.9	59.90	52.4	4.21	0.52	1.54	1.00	1.94	5.42	3.880
37.9	47.85	99.0	2.40	0.56	0.49	0.00	1.14	8.91	6.050
27.1	21.30	63.3	2.19	0.39	0.79	1.00	0.93	6.68	5.230
28.5	35.90	80.3	1.70	0.33	0.48	0.00	1.14	4.62	3.380
26.7	967.70	68.2	3.24	1.14	1.25	1.00	1.59	8.31	4.610
29.7	56.10	58.9	3.61	0.94	0.76	1.00	2.32	6.91	4.870
45.2	21.50	65.7	3.20	1.12	1.03	1.00	1.52	3.52	2.480
22.7	47.85	99.0	2.40	0.56	0.49	0.00	1.14	5.99	4.500
39.0	494.70	114.2	2.58	0.61	0.55	1.00	1.56	4.49	2.940
36.6	131.70	136.7	2.26	0.81	0.12	0.00	1.12	9.35	8.030
24.7	20.60	89.1	3.01	0.38	1.18	1.00	0.46	2.84	1.840
29.2	5.80	39.3	1.52	0.11	0.63	1.00	0.78	18.96	15.090
25.9	11.60	36.0	2.52	0.46	0.66	1.00	1.09	12.95	11.270
57.8	33.30	378.5	2.40	0.56	0.49	0.00	1.14	3.77	1.920
27.7	18.10	93.1	2.20	0.29	0.17	0.00	1.02	6.03	3.920
17.7	30.10	55.6	2.29	0.51	0.66	1.00	1.23	5.39	4.040
26.9	98.40	82.2	2.27	0.53	0.17	0.00	1.53	13.39	10.130
24.4	21.20	29.7	3.16	0.33	0.84	1.00	1.83	8.52	7.540
21.7	21.40	49.5	2.46	0.43	0.77	1.00	1.54	4.10	3.170
33.9	55.70	62.0	2.04	0.88	0.57	1.00	0.97	7.30	5.900
31.2	59.70	130.4	2.50	0.39	0.37	0.00	1.51	6.30	2.800
37.8	16.00	100.5	3.02	0.43	0.19	0.00	1.59	3.53	2.230
27.6	15.90	66.8	3.52	0.35	1.54	1.00	1.49	4.78	3.160
25.2	150.40	56.6	1.91	0.47	0.17	0.00	1.38	5.51	3.390
26.3	25.10	60.6	3.08	0.92	0.83	1.00	1.67	5.13	4.000
18.6	11.20	40.4	2.41	1.00	0.92	1.00	1.25	8.80	7.000
18.5	45.80	160.7	2.25	0.73	0.80	1.00	0.83	4.57	3.440
33.1	18.40	62.1	2.68	0.34	1.16	1.00	1.36	3.91	3.190
30.0	61.90	83.0	2.45	0.30	1.02	1.00	1.00	8.00	7.350
33.3	40.30	49.3	2.46	3.21	0.78	1.00	0.88	6.02	4.710
34.2	43.40	102.2	4.64	0.35	1.55	1.00	2.33	5.28	1.870
29.5	13.20	58.6	2.70	0.95	0.65	1.00	1.55	5.91	4.660
71.8	165.00	330.6	3.57	0.49	0.94	1.00	1.91	8.54	6.300
33.0	72.90	208.4	2.20	1.36	0.12	0.00	0.97	4.73	2.900
23.3	68.70	81.5	2.06	1.16	0.10	0.00	0.84	5.62	3.130
40.0	38.40	124.5	3.21	0.55	1.45	1.00	1.88	4.70	3.400
26.0	13.40	86.6	1.32	0.58	0.56	1.00	0.22	2.20	1.760

31.8	52.80	79.1	4.48	0.42	1.22	1.00	2.63	7.03	4.990
33.7	32.80	89.3	2.23	0.74	0.57	1.00	1.17	4.01	2.170
24.5	11.70	71.3	1.61	0.22	0.76	1.00	0.70	5.33	3.940
44.0	32.70	62.4	2.46	0.43	0.77	1.00	1.31	5.91	4.050
28.7	84.30	108.6	2.06	1.12	0.12	0.00	1.08	6.31	4.360
26.6	47.85	99.0	2.40	0.56	0.49	0.00	1.14	3.04	2.110
34.7	59.30	101.3	3.01	0.52	1.35	1.00	1.08	6.66	5.880
24.0	18.10	49.2	1.65	0.80	0.43	0.00	0.64	4.99	3.700
35.8	163.70	66.8	2.02	0.79	0.88	1.00	1.03	7.40	5.160
30.2	188.70	95.3	2.25	0.99	0.39	0.00	1.07	6.59	5.260
38.5	14.50	73.8	4.62	0.78	1.23	1.00	2.14	3.51	2.460
20.9	84.30	113.7	2.49	0.39	0.92	1.00	1.25	2.71	1.500
29.3	182.10	138.8	3.28	0.21	0.94	1.00	1.77	10.15	9.290
42.5	26.00	205.2	3.17	0.67	1.17	1.00	1.19	4.81	3.140
32.0	541.50	77.6	3.49	1.29	0.18	0.00	1.69	4.23	3.790
20.9	51.30	78.5	3.45	0.42	1.64	1.00	1.19	2.47	1.600
16.0	7.40	30.1	1.52	0.38	0.60	1.00	0.72	8.42	7.200
26.1	14.80	56.2	2.40	0.56	0.49	0.00	1.14	1.71	1.300
23.6	23.40	99.7	1.55	0.35	0.07	0.00	0.71	10.18	8.290
43.1	47.85	99.0	2.40	0.56	0.49	0.00	1.14	4.06	2.980
24.4	24.60	72.9	3.09	0.41	2.29	1.00	0.78	2.00	1.300
25.9	47.85	99.0	2.40	0.56	0.49	0.00	1.14	8.53	7.040
34.3	166.20	49.0	3.69	1.29	0.36	0.00	2.50	13.36	11.760
38.6	66.20	102.0	3.09	1.06	0.90	1.00	1.27	7.44	5.040
29.1	189.50	527.8	2.01	1.18	0.11	0.00	0.89	31.72	27.190
13.9	50.40	64.9	1.10	0.17	0.36	0.00	0.73	2.39	1.900
21.2	19.90	50.6	2.85	0.93	0.62	1.00	1.73	7.02	6.410
16.6	36.70	22.8	1.34	0.36	0.14	0.00	0.48	13.62	12.510
27.2	30.80	55.9	2.44	0.42	1.04	1.00	1.16	3.78	3.090
32.2	39.80	105.9	4.85	0.63	1.71	1.00	2.28	5.26	2.450
21.4	16.00	40.6	2.68	0.44	0.74	1.00	1.71	2.98	2.370
24.1	90.70	72.4	2.65	0.53	0.40	0.00	1.84	8.84	7.500
25.9	21.90	55.5	1.91	0.59	0.11	0.00	1.34	5.39	3.920
16.8	26.00	56.0	2.38	0.85	0.40	0.00	1.32	3.19	2.530
35.0	17.40	163.6	2.51	0.63	0.86	1.00	1.19	3.96	2.550
29.8	317.90	45.7	2.61	1.01	0.18	0.00	1.05	12.66	10.650
29.9	47.85	99.0	2.40	0.56	0.49	0.00	1.14	7.57	5.480
28.2	1393.70	101.7	5.55	2.09	0.50	0.00	4.05	5.05	2.530
29.1	42.80	79.6	2.62	0.89	0.72	1.00	0.98	13.01	10.620
39.7	47.85	99.0	2.40	0.56	0.49	0.00	1.14	5.48	4.100
28.2	68.60	165.8	5.01	0.59	1.78	1.00	2.19	3.23	2.370
33.6	18.00	125.3	2.46	0.25	1.04	1.00	0.98	3.75	2.150
44.4	32.60	116.0	2.19	0.35	1.09	1.00	0.74	2.08	1.310
31.4	256.60	298.9	5.23	0.51	1.27	1.00	2.95	7.40	5.630
19.4	214.60	203.3	2.25	1.20	0.67	1.00	0.87	11.84	10.600

27.6	18.20	68.8	3.14	1.03	0.67	1.00	2.01	2.57	2.080
25.1	120.20	134.4	2.11	0.82	0.14	0.00	0.68	10.57	9.370
36.5	57.30	177.4	3.44	0.94	1.15	1.00	1.79	4.06	2.190
28.7	30.00	94.8	3.23	0.50	1.21	1.00	1.60	4.34	1.860
29.2	21.90	66.3	3.04	0.36	0.88	1.00	1.55	1.42	0.800
31.1	114.50	126.4	2.30	0.51	0.27	0.00	1.52	14.31	12.040
48.5	18.60	52.1	2.74	0.49	0.64	1.00	1.67	14.25	12.290
25.6	40.90	47.8	0.95	0.38	0.14	0.00	0.40	5.97	4.620
31.5	144.90	125.6	3.80	0.86	0.89	1.00	1.80	4.49	2.140
20.4	54.30	54.8	2.40	2.01	0.74	1.00	1.27	6.20	5.400
48.5	18.80	91.0	2.59	0.29	0.92	1.00	1.33	4.11	1.900
25.8	11.80	72.6	2.00	0.41	0.95	1.00	0.95	6.00	4.700
23.8	13.80	53.0	1.46	0.74	0.47	0.00	0.71	8.32	5.630
31.1	29.90	113.8	3.91	0.69	0.51	1.00	1.81	2.42	1.200
31.2	21.50	69.0	2.20	0.35	1.04	1.00	0.76	3.22	2.680
27.5	16.00	67.4	2.06	0.82	0.58	1.00	1.08	3.54	2.610
43.6	143.30	200.2	3.26	0.39	0.93	1.00	1.80	2.88	2.100
29.8	44.90	148.7	3.55	0.61	0.61	1.00	2.39	2.57	1.920
21.8	18.80	148.9	2.50	0.34	0.88	1.00	0.91	1.65	1.090
21.0	60.30	54.2	1.99	0.61	0.56	1.00	0.89	13.79	10.900
29.3	26.70	66.8	2.21	0.29	0.95	1.00	0.86	4.19	3.670
36.0	22.40	176.1	2.97	0.40	1.16	1.00	1.51	5.93	4.510
21.2	47.85	99.0	2.40	0.56	0.49	0.00	1.14	7.20	6.000
26.6	26.20	36.0	2.32	0.11	0.80	1.00	1.37	5.81	4.180
26.2	18.70	125.8	3.47	0.36	0.78	1.00	1.48	5.60	3.770
28.6	47.85	99.0	2.40	0.56	0.49	0.00	1.14	4.10	2.850
16.7	8.60	55.8	2.30	0.33	0.89	1.00	0.98	4.83	4.330
29.1	12.10	82.8	2.40	0.56	0.49	0.00	1.14	1.66	1.100
44.5	138.80	177.3	4.34	1.22	1.39	1.00	2.72	5.83	3.980
33.9	18.90	68.6	3.29	0.68	1.01	1.00	1.69	4.33	3.070
23.5	38.70	61.3	3.86	1.17	0.77	1.00	2.33	7.86	5.740
31.6	72.60	54.4	4.52	0.58	1.37	1.00	2.48	10.23	7.890
34.1	46.10	122.6	2.50	0.52	1.09	1.00	1.20	6.70	5.200
30.9	46.90	107.1	2.00	0.61	0.80	1.00	1.95	2.90	2.200
34.2	16.70	85.1	2.57	0.48	0.78	1.00	1.20	3.46	1.380
34.6	51.40	145.4	2.67	0.84	1.04	1.00	1.73	1.82	1.400
29.5	25.50	109.9	2.82	0.36	1.00	1.00	1.36	12.73	11.200
36.6	356.10	82.1	8.66	0.87	1.23	1.00	6.15	4.26	2.660
29.5	30.60	83.8	3.94	1.14	1.65	1.00	1.39	7.83	5.330
16.7	47.85	99.0	2.40	0.56	0.49	0.00	1.14	18.93	15.010
33.3	36.20	88.5	3.19	0.58	0.67	1.00	2.12	7.29	3.880
23.6	13.50	95.9	1.95	0.27	0.91	1.00	0.68	1.68	1.240
31.6	39.20	61.8	1.88	0.71	0.13	0.00	0.83	5.41	4.520
18.3	317.60	91.8	2.65	1.57	0.37	0.00	1.48	17.90	12.300
35.7	50.10	172.6	2.58	0.59	1.06	1.00	1.20	3.42	1.900

18.9	17.20	18.5	1.09	0.28	0.10	0.00	0.16	15.08	11.280
29.7	11.10	67.9	2.38	0.40	0.80	1.00	1.32	1.70	1.100
56.1	35.00	83.1	2.72	0.49	1.20	1.00	1.59	2.72	2.100
29.7	47.85	99.0	2.40	0.56	0.49	0.00	1.14	2.70	1.300
35.8	27.10	62.7	2.03	0.34	0.70	1.00	1.13	4.84	2.860
25.4	332.20	94.5	2.32	1.04	1.05	1.00	0.93	3.62	2.640
36.5	72.70	118.2	5.38	2.63	2.17	1.00	2.11	3.82	2.100
40.1	25.70	168.0	5.78	0.55	0.37	0.00	3.17	10.46	8.230
31.1	17.20	47.8	2.17	0.46	0.75	1.00	0.85	2.49	2.120
28.9	15.40	69.7	3.11	0.78	1.24	1.00	1.25	2.91	1.660
35.0	12.20	114.7	4.29	0.40	1.98	1.00	1.46	7.20	4.220
39.6	81.40	286.0	1.69	0.58	0.46	0.00	0.57	3.00	1.300
29.4	13.30	104.3	3.16	0.58	1.10	1.00	1.58	5.32	2.800
41.4	31.40	131.3	2.73	0.56	1.29	1.00	1.38	5.90	4.400
49.4	40.30	92.3	1.53	0.42	0.07	0.00	0.74	4.51	3.590
31.3	47.85	99.0	2.40	0.56	0.49	0.00	1.14	14.09	11.750
36.1	21.60	163.3	3.82	0.66	0.99	1.00	1.57	5.03	3.070
36.6	32.10	147.3	3.07	0.90	1.14	1.00	1.53	3.14	1.540
24.8	77.70	123.5	2.73	1.05	0.13	0.00	1.11	7.69	4.550
16.3	20.30	91.7	2.62	1.02	0.57	1.00	1.47	7.54	5.260
18.4	95.70	61.2	1.18	0.72	0.20	0.00	0.47	7.81	5.840
36.4	46.30	96.3	4.22	0.83	0.65	1.00	2.43	4.50	2.900
28.1	17.60	46.4	2.60	0.70	0.63	1.00	1.55	9.78	8.180
39.9	73.40	91.8	3.41	0.91	0.68	1.00	1.63	5.96	4.930
38.0	47.85	99.0	2.40	0.56	0.49	0.00	1.14	2.37	1.820
46.6	59.10	119.2	4.61	1.36	0.55	1.00	2.14	5.17	3.240
31.7	27.00	165.6	3.58	0.85	1.09	1.00	0.99	2.80	1.900
36.4	35.50	65.4	2.55	0.75	0.49	0.00	1.31	1.34	0.880
23.7	7.10	35.2	1.59	0.14	0.70	1.00	0.92	5.30	4.470
44.3	90.60	138.7	2.92	1.09	0.37	0.00	1.80	5.28	3.090
32.0	62.60	127.3	3.34	0.72	0.85	1.00	1.68	4.44	1.900
28.7	47.85	99.0	2.40	0.56	0.49	0.00	1.14	5.57	3.620
34.3	15.20	77.5	1.21	0.85	0.13	0.00	0.46	9.63	8.100
38.0	54.20	111.9	2.56	0.36	0.98	1.00	1.15	3.73	2.860
24.7	34.50	70.3	1.70	0.49	0.60	1.00	0.90	5.03	4.730
30.7	538.50	65.5	2.70	1.14	0.12	0.00	1.49	12.64	10.590
19.2	73.50	64.8	2.19	0.80	0.56	1.00	1.30	12.83	10.690
19.0	282.70	65.6	2.69	0.59	0.13	0.00	1.52	10.85	8.790
21.2	88.00	72.0	2.51	0.42	1.01	1.00	1.09	9.61	7.590
16.7	7.10	32.7	2.02	0.45	0.69	1.00	1.08	5.28	3.600
28.4	47.85	99.0	2.40	0.56	0.49	0.00	1.14	15.61	12.750
18.0	6.70	43.9	1.31	0.30	0.52	1.00	0.37	4.97	4.590
27.6	40.60	108.4	4.27	0.83	1.45	1.00	1.11	4.64	3.240
32.9	27.80	119.5	2.79	0.54	1.08	1.00	1.16	3.90	1.790
24.8	21.50	52.9	1.97	0.40	0.61	1.00	0.81	2.91	2.150

35.3	47.85	99.0	2.40	0.56	0.49	0.00	1.14	22.05	18.870
37.9	21.80	49.5	0.87	0.25	0.09	0.00	0.24	4.31	3.300
52.1	38.90	59.4	2.88	0.27	0.93	1.00	1.68	3.62	3.060
32.4	25.20	78.5	4.06	0.79	1.24	1.00	1.91	2.99	1.490
27.9	24.90	92.5	1.56	0.23	0.13	0.00	0.39	5.81	4.990
26.8	126.60	41.5	1.63	0.37	0.22	0.00	1.17	9.12	7.030
39.1	73.00	215.8	3.18	0.62	1.19	1.00	1.09	7.07	3.500
28.2	47.85	99.0	2.40	0.56	0.49	0.00	1.14	14.35	10.650
26.8	21.40	114.9	2.22	0.55	1.31	1.00	0.99	2.92	2.000
33.1	39.00	65.0	2.06	0.98	0.13	0.00	0.82	3.62	2.510
24.3	14.00	74.1	2.41	0.40	0.94	1.00	0.95	1.97	0.890
29.3	8.60	68.0	3.98	0.33	1.16	1.00	2.14	2.34	1.500
33.0	51.00	215.8	2.91	0.85	1.12	1.00	1.07	4.56	2.510
29.8	11.60	52.0	2.72	0.38	0.97	1.00	1.38	5.30	3.250
42.2	47.85	99.0	2.40	0.56	0.49	0.00	1.14	4.06	2.600
30.0	106.40	99.8	3.29	2.51	1.19	1.00	1.43	8.19	5.400
18.4	12.30	55.4	2.08	0.46	0.70	1.00	1.09	8.78	6.490
12.4	11.80	35.3	1.20	0.13	0.52	1.00	0.67	13.61	11.610
45.6	15.90	197.2	1.55	0.36	0.41	0.00	0.86	3.10	1.800
24.5	47.85	99.0	2.40	0.56	0.49	0.00	1.14	2.45	1.830
40.3	305.70	135.1	4.35	1.01	1.23	1.00	2.41	7.44	4.600
45.0	47.85	99.0	2.40	0.56	0.49	0.00	1.14	10.50	7.800
42.1	101.10	57.3	5.40	1.49	1.18	1.00	3.57	3.37	1.800
22.8	11.60	58.0	2.65	0.75	0.73	1.00	1.24	6.69	4.590
21.5	36.80	62.1	4.30	0.70	1.06	1.00	2.79	7.57	5.650
35.4	23.80	113.1	3.24	0.44	0.78	1.00	2.13	3.95	3.020
31.5	320.80	99.4	2.55	0.83	0.28	0.00	1.05	11.13	9.740
46.8	38.10	53.0	3.22	0.60	1.40	1.00	1.35	5.81	4.320
30.9	75.00	70.6	2.69	0.81	0.73	1.00	1.09	1.96	0.990
26.7	128.20	59.5	2.50	1.40	0.36	0.00	1.55	3.75	1.880
35.1	87.20	137.4	2.65	0.79	0.15	0.00	1.63	8.24	6.180
16.6	6.90	63.4	1.45	0.11	0.61	1.00	0.55	26.87	23.670
37.8	116.90	129.4	1.93	0.54	0.11	0.00	0.89	7.03	5.500
40.1	30.60	108.8	2.45	0.37	1.08	1.00	0.48	2.60	1.540
26.4	9.90	56.7	3.73	0.83	1.48	1.00	1.68	7.10	5.600
20.4	54.00	50.4	1.33	0.49	0.40	0.00	0.63	9.71	5.280
30.8	52.00	104.8	2.06	0.62	0.43	0.00	1.07	3.32	2.010
30.0	96.50	171.2	2.50	0.42	0.68	1.00	1.48	3.33	2.410
30.8	550.80	169.8	10.39	5.87	0.48	0.00	3.45	5.60	3.460
40.2	13.30	47.9	2.60	0.88	1.04	1.00	1.37	1.56	1.140
41.8	87.30	127.1	2.99	0.60	0.51	1.00	2.28	7.15	3.330
20.9	18.40	90.6	3.03	0.37	0.64	1.00	1.16	4.22	3.000
31.3	22.10	50.7	3.46	1.39	0.89	1.00	1.79	2.49	1.490
36.6	75.40	87.4	4.41	1.63	1.17	1.00	2.30	3.87	2.180
32.1	108.40	139.6	2.90	0.73	0.61	1.00	1.78	4.31	1.960

23.3	19.10	109.2	3.21	0.42	1.85	1.00	1.51	3.12	1.430
23.5	97.90	119.5	1.77	0.42	0.14	0.00	0.56	6.22	4.750
35.8	9.70	65.8	2.60	0.19	0.64	1.00	1.68	3.95	3.610
26.2	383.70	214.7	2.61	0.56	1.00	1.00	1.42	6.60	5.500
35.4	55.50	131.1	1.68	0.70	0.13	0.00	0.68	7.54	6.340
41.6	10.30	69.8	2.22	0.37	0.56	1.00	1.21	3.69	2.250
40.5	98.20	119.3	2.83	0.79	0.98	1.00	1.53	4.10	2.800
30.9	37.00	74.4	3.29	0.85	1.90	1.00	1.20	4.00	2.900
32.3	51.30	90.5	3.71	0.75	0.74	1.00	2.49	7.80	5.900
35.8	181.60	64.3	4.15	1.49	0.63	1.00	1.68	8.97	8.010
30.9	143.10	89.2	3.33	0.58	1.23	1.00	1.84	3.53	2.810
52.5	30.80	100.8	3.13	0.42	1.48	1.00	1.82	6.31	4.800
32.5	219.60	115.7	2.37	0.40	0.18	0.00	1.30	9.58	8.250
30.3	36.70	75.7	5.62	0.47	1.37	1.00	3.55	3.11	1.380
34.4	24.00	58.3	3.29	0.47	0.91	1.00	1.95	5.65	3.770
40.8	193.80	143.5	2.84	0.66	0.55	1.00	1.31	3.69	2.180
37.0	31.70	110.2	2.35	0.37	0.69	1.00	1.28	6.54	4.370
38.1	15.80	119.9	2.29	0.33	0.87	1.00	0.84	5.26	1.760
26.3	222.40	70.5	4.67	0.45	1.78	1.00	2.18	15.62	13.560
23.6	16.30	56.7	2.79	0.51	0.84	1.00	1.49	3.65	3.260
18.9	21.00	62.4	2.71	0.51	0.81	1.00	1.48	9.89	8.300
32.7	119.50	84.1	1.29	0.39	0.13	0.00	0.54	7.64	4.720
22.8	14.20	45.2	1.28	0.41	0.65	1.00	0.41	2.46	1.900
26.2	17.90	78.3	2.27	0.74	0.84	1.00	0.68	4.73	4.150
36.7	54.60	226.1	2.40	0.56	0.49	0.00	1.14	1.60	0.530
28.9	5.90	97.7	2.40	0.56	0.49	0.00	1.14	3.62	1.990
36.9	1820.80	276.3	5.80	2.27	0.65	1.00	3.59	11.34	8.980
42.3	47.10	106.5	2.71	0.42	0.23	0.00	1.66	6.60	4.660
32.5	9.70	104.5	2.96	0.74	1.31	1.00	1.23	1.89	1.200
41.4	49.70	174.3	3.56	1.50	0.95	1.00	1.35	3.81	1.940
45.6	117.00	80.2	2.40	0.56	0.49	0.00	1.14	8.70	5.100
31.4	104.10	91.6	2.60	0.53	0.85	1.00	1.40	2.78	1.740
41.1	25.30	80.9	2.44	0.12	1.14	1.00	1.01	8.59	7.240
48.9	38.80	97.6	3.72	1.23	0.81	1.00	2.48	6.60	4.900
24.8	15.60	53.2	2.57	0.36	0.88	1.00	1.56	2.38	1.490
30.9	63.60	67.5	1.84	0.73	0.67	1.00	0.98	4.60	3.000
51.5	47.85	99.0	2.40	0.56	0.49	0.00	1.14	3.34	1.690
19.3	9.80	48.9	2.40	0.56	0.49	0.00	1.14	7.29	5.960
33.8	106.00	70.7	2.93	1.56	0.73	1.00	1.51	4.73	3.220
38.8	7.70	50.7	1.15	0.04	0.22	0.00	0.89	3.59	2.270
22.2	14.90	65.7	2.86	0.77	0.81	1.00	1.61	5.50	4.150
22.3	5.10	128.0	1.92	0.28	0.79	1.00	0.72	1.44	0.880
50.0	84.30	168.6	3.02	1.49	0.17	0.00	1.44	4.09	3.750
19.8	146.90	40.5	2.40	0.56	0.49	0.00	1.14	5.27	3.620
33.5	84.50	70.8	6.53	1.24	1.29	1.00	3.72	5.98	2.710

33.9	22.00	77.3	4.49	1.08	0.59	1.00	2.65	13.35	11.910
32.0	2439.10	322.9	4.89	3.17	0.25	0.00	4.97	10.55	8.530
35.7	14.10	111.1	1.83	0.16	0.61	1.00	0.75	7.40	5.150
22.3	27.70	72.5	2.00	0.48	0.66	1.00	0.76	5.89	4.760
45.6	48.50	116.7	4.71	0.60	1.70	1.00	1.95	5.67	3.960
13.3	17.70	24.6	1.22	0.22	0.39	0.00	0.65	4.80	4.110
29.7	17.40	44.5	4.46	1.13	1.46	1.00	1.94	4.09	2.860
25.8	54.00	142.7	3.97	0.59	1.02	1.00	2.10	3.33	1.770
25.5	23.40	66.4	2.03	0.45	0.68	1.00	0.79	3.42	2.770
30.0	59.30	189.1	3.23	0.63	1.07	1.00	1.37	5.36	3.190
25.9	24.80	74.9	5.23	0.52	1.46	1.00	3.40	10.40	3.700
25.8	14.10	56.9	2.05	0.71	0.36	0.00	0.85	11.31	8.340
31.9	47.85	99.0	2.40	0.56	0.49	0.00	1.14	6.80	4.300
40.4	18.10	82.4	4.70	0.65	1.20	1.00	2.79	4.08	1.000
26.1	46.70	112.9	1.48	0.53	0.11	0.00	0.45	13.08	10.370
36.6	59.30	167.8	4.08	0.56	1.12	1.00	1.70	3.36	2.360
25.9	24.50	99.6	3.24	0.83	1.10	1.00	1.73	2.90	2.100
35.5	22.00	145.1	3.46	0.35	1.21	1.00	1.36	2.09	1.050
32.3	108.20	71.7	3.08	1.16	0.89	1.00	1.62	4.17	2.620
30.6	111.80	81.9	4.32	1.00	0.89	1.00	2.28	4.40	2.600
37.4	39.20	95.4	3.23	0.33	1.47	1.00	1.42	3.52	2.650
36.1	87.60	94.0	2.55	0.98	0.15	0.00	1.07	5.18	3.350
26.0	254.00	169.0	2.94	0.66	1.22	1.00	1.68	3.87	2.060
22.5	69.20	67.1	2.83	1.30	0.87	1.00	1.50	14.81	10.000
29.9	47.85	99.0	2.40	0.56	0.49	0.00	1.14	6.44	5.190
26.6	17.70	76.5	2.67	0.61	1.17	1.00	1.45	6.80	4.500
30.6	15.20	157.0	3.89	0.60	1.51	1.00	1.62	4.02	2.780
48.6	48.10	82.0	6.76	1.20	0.83	1.00	4.77	6.43	3.080
17.9	156.50	46.6	0.28	0.09	0.09	0.00	0.24	7.40	5.650
45.1	60.90	171.3	1.26	0.49	0.34	0.00	0.56	12.71	10.400
40.9	19.80	106.9	3.62	0.43	1.39	1.00	1.67	5.50	1.500
19.6	156.90	46.5	3.34	0.73	0.71	1.00	1.98	6.50	4.520
13.3	90.60	210.9	1.55	1.08	0.33	0.00	0.59	2.44	1.710
31.2	34.90	99.3	3.99	0.97	1.05	1.00	2.42	3.83	2.070
19.1	60.10	58.2	1.41	0.55	0.09	0.00	0.66	6.11	3.290
24.8	11.70	40.1	2.17	0.55	0.58	1.00	1.35	4.98	3.770
20.6	20.40	45.7	2.25	0.49	0.69	1.00	1.14	10.03	7.480
21.0	110.50	106.5	1.63	0.41	0.10	0.00	0.57	12.96	9.800
24.4	15.50	87.0	2.71	0.37	1.14	1.00	1.10	2.89	1.890
18.9	40.30	40.0	1.83	0.48	0.51	1.00	0.71	12.52	9.400
19.2	24.60	55.9	2.72	0.83	0.65	1.00	1.44	3.75	2.670
27.4	135.90	119.3	3.59	0.92	0.60	1.00	2.14	5.50	3.690
16.0	30.10	44.3	2.40	0.56	0.49	0.00	1.14	9.60	7.420
32.1	82.50	51.2	2.40	0.56	0.49	0.00	1.14	5.59	4.230
28.8	22.50	96.7	1.18	0.44	0.36	0.00	0.54	10.71	9.400

29.7	12.30	99.4	2.62	0.67	0.90	1.00	1.27	1.80	1.120
19.8	80.20	185.4	1.91	0.94	0.14	0.00	0.82	5.18	3.300
33.4	40.00	146.9	3.69	0.49	1.52	1.00	1.47	3.27	1.940
34.5	12.20	85.6	3.04	0.31	0.96	1.00	1.47	7.49	5.740
30.2	37.10	122.9	3.40	0.44	1.17	1.00	1.53	11.27	9.160
24.8	6.20	61.7	1.42	0.46	0.79	1.00	0.57	2.99	1.950
31.2	18.80	77.7	2.78	0.99	1.21	1.00	1.60	6.30	4.900
28.5	40.50	87.1	3.37	0.61	0.98	1.00	1.35	5.67	3.700
43.4	38.90	199.9	1.95	0.55	0.41	0.00	0.72	8.73	4.940
28.0	19.50	307.2	3.25	0.64	1.03	1.00	1.50	4.97	1.850
34.9	70.50	47.0	4.13	0.50	1.18	1.00	2.31	7.28	3.770
32.3	42.90	114.4	2.10	0.68	0.37	0.00	1.26	7.08	5.820
40.9	23.40	86.8	3.72	0.57	0.82	1.00	2.42	3.92	3.000
25.0	48.80	123.9	2.23	0.41	0.21	0.00	1.01	14.29	12.700
24.6	124.60	134.1	3.86	0.73	1.11	1.00	2.16	8.35	6.390
27.7	166.20	66.9	5.07	1.46	2.33	1.00	1.97	4.66	1.970
21.3	12.10	80.8	2.96	0.38	1.30	1.00	1.28	5.10	3.720
30.7	123.10	66.6	2.50	0.73	0.46	0.00	1.42	5.46	4.340
21.0	49.70	49.3	2.10	0.65	0.62	1.00	0.87	7.07	5.190
43.7	47.85	99.0	2.40	0.56	0.49	0.00	1.14	1.97	0.810
39.9	27.60	53.9	3.26	0.46	0.99	1.00	1.78	4.68	3.530
51.2	512.70	116.2	4.39	2.05	1.29	1.00	1.22	9.17	7.220
34.9	108.00	148.8	4.74	0.88	2.26	1.00	1.89	6.42	4.390
43.9	54.20	135.7	1.81	0.31	0.33	0.00	1.18	6.09	4.750
44.4	17.80	194.6	1.95	0.34	0.55	1.00	1.30	5.40	4.100
31.2	11.90	195.8	1.33	0.89	0.59	1.00	0.25	1.50	0.830
27.4	31.10	132.7	3.66	0.68	1.92	1.00	1.43	3.12	1.000
27.8	61.60	143.8	2.69	1.00	0.13	0.00	1.29	5.88	4.100
30.1	28.20	78.4	3.26	0.34	1.87	1.00	1.55	2.42	1.700
15.4	2.30	23.7	1.03	0.17	0.34	0.00	0.61	8.83	6.360
49.4	165.10	157.9	2.40	0.56	0.49	0.00	1.14	6.10	3.500
36.0	30.40	113.9	1.82	0.47	0.77	1.00	0.65	1.95	1.650
25.3	39.60	47.3	2.16	0.57	0.44	0.00	1.27	3.02	2.190
29.8	48.50	95.3	4.35	2.90	0.81	1.00	1.91	5.13	3.660
22.4	24.90	74.1	1.01	0.17	0.13	0.00	0.52	34.02	27.590
27.2	39.10	47.4	2.25	0.14	0.90	1.00	1.06	10.40	9.100
18.6	1429.40	46.3	3.76	2.38	0.26	0.00	2.07	3.32	3.000
28.1	10.50	55.4	2.62	1.01	0.59	1.00	1.08	7.80	5.900
37.8	228.10	95.2	3.56	0.90	0.85	1.00	2.20	2.30	1.320
65.3	89.00	156.7	3.44	0.63	0.39	0.00	2.68	5.18	3.990
33.0	59.90	142.5	3.60	0.42	1.45	1.00	2.02	6.00	4.600
32.2	14.90	79.2	1.95	0.43	0.67	1.00	1.25	6.98	5.080
24.6	17.20	83.7	2.18	0.34	0.80	1.00	1.27	18.11	15.300
27.6	14.80	80.1	2.23	0.22	1.06	1.00	1.23	5.40	4.100
29.7	47.85	99.0	2.40	0.56	0.49	0.00	1.14	2.82	2.100

30.7	24.20	76.6	4.13	0.75	1.34	1.00	2.52	9.60	6.900
19.7	32.30	87.9	2.15	0.30	0.52	1.00	1.32	10.70	7.600
33.0	47.85	99.0	2.40	0.56	0.49	0.00	1.14	4.20	2.600
39.3	12.30	96.6	1.85	0.28	0.88	1.00	0.91	8.30	6.500
21.0	17.00	41.5	1.55	0.24	0.98	1.00	0.53	12.40	9.000
29.7	47.85	99.0	2.40	0.56	0.49	0.00	1.14	3.12	1.700
38.0	9.00	118.8	3.36	0.51	1.30	1.00	1.80	3.52	2.020
27.2	104.90	92.1	2.38	0.74	0.42	0.00	1.79	6.60	5.400
26.6	44.90	84.2	2.65	0.85	0.94	1.00	1.62	4.30	3.100
21.6	55.50	49.6	3.24	0.64	1.40	1.00	1.51	5.40	4.400
39.8	31.60	156.8	1.72	0.59	0.40	0.00	1.04	6.70	3.600
30.0	33.00	93.9	4.00	0.52	1.18	1.00	3.09	3.80	1.600
23.7	24.70	53.0	1.77	0.21	1.08	1.00	0.75	6.00	4.300
45.9	11.70	111.7	4.20	0.55	1.23	1.00	2.28	3.00	1.200
34.4	47.85	99.0	2.40	0.56	0.49	0.00	1.14	7.87	6.930
21.6	880.80	110.5	1.78	0.96	0.15	0.00	0.85	10.08	8.940
31.8	47.85	99.0	2.40	0.56	0.49	0.00	1.14	18.13	15.310
19.3	13.20	59.3	2.69	1.62	0.71	1.00	1.44	10.84	7.780
14.0	37.10	268.6	0.73	0.30	0.10	0.00	0.27	13.12	10.350
20.0	47.85	99.0	2.40	0.56	0.49	0.00	1.14	30.14	27.650
27.2	47.85	99.0	2.40	0.56	0.49	0.00	1.14	13.63	12.070
13.7	42.30	34.5	2.40	0.56	0.49	0.00	1.14	19.70	16.800
36.6	40.80	99.9	1.15	0.34	0.12	0.00	0.20	10.42	7.220
37.8	19.20	106.8	3.13	0.45	0.35	0.00	0.73	5.74	3.760
26.1	47.85	99.0	2.40	0.56	0.49	0.00	1.14	5.26	4.130
30.4	23.70	71.5	1.60	0.32	0.53	1.00	0.85	3.82	2.670
35.1	67.90	107.2	1.12	0.29	0.08	0.00	0.07	8.57	6.750
24.6	125.40	67.4	5.06	1.23	0.20	0.00	2.81	10.87	9.440
32.5	18.90	42.3	1.60	0.40	0.56	1.00	0.59	17.71	16.630
25.6	184.70	41.5	1.30	0.39	0.29	0.00	0.61	7.40	6.500
29.8	162.70	112.1	1.83	0.82	0.13	0.00	0.59	8.87	7.090
16.9	47.85	99.0	2.40	0.56	0.49	0.00	1.14	4.76	3.650
29.3	58.10	145.4	1.53	0.71	0.10	0.00	0.70	14.51	12.400
19.4	31.10	136.1	1.54	0.33	0.84	1.00	0.41	4.00	2.900
37.8	24.20	105.9	2.55	0.53	0.56	1.00	1.71	7.03	5.970
23.5	47.85	99.0	2.40	0.56	0.49	0.00	1.14	11.61	7.920
24.6	12.50	85.4	1.00	0.39	0.10	0.00	0.55	7.41	6.430
55.4	96.30	61.8	1.57	1.86	0.09	0.00	0.40	21.17	20.180
39.2	31.90	115.4	2.45	0.59	0.15	0.00	1.45	7.18	4.920
35.1	241.00	178.3	2.89	0.68	0.14	0.00	1.89	12.94	10.360
30.5	90.80	102.9	1.33	0.78	0.65	1.00	0.52	15.40	13.200
29.7	445.10	69.4	2.42	0.78	0.11	0.00	0.96	12.69	11.380
18.2	34.90	51.1	1.16	0.69	0.13	0.00	0.53	23.08	18.710
16.5	35.40	46.1	1.72	0.70	0.11	0.00	0.92	2.49	2.310
20.3	22.30	44.3	0.98	0.45	0.07	0.00	0.14	17.29	15.520

31.5	22.70	172.6	1.24	0.43	0.08	0.00	0.28	4.87	3.790
19.6	21.90	168.0	0.91	0.31	0.07	0.00	0.34	3.03	2.690
31.2	74.70	53.3	1.58	0.63	0.09	0.00	0.97	6.88	4.840
34.4	129.10	380.5	2.30	0.94	0.31	0.00	1.73	24.00	21.300
30.4	75.20	173.3	1.98	0.92	0.32	0.00	0.88	13.60	10.300
38.7	122.70	174.0	1.46	0.45	0.12	0.00	0.46	5.76	3.630
23.3	83.10	120.4	1.51	0.53	0.09	0.00	0.26	9.74	7.530
28.2	90.30	133.3	1.50	0.75	0.22	0.00	0.75	29.23	25.540
24.6	114.80	56.6	1.85	1.16	0.07	0.00	0.92	10.92	8.570
40.2	50.70	41.9	0.95	0.09	0.20	0.00	0.57	26.67	23.730
18.5	507.30	127.1	2.36	1.27	0.11	0.00	1.07	15.03	11.720
43.6	84.00	82.3	1.92	0.45	0.16	0.00	0.69	15.87	14.690
39.3	25.70	76.6	2.41	0.32	0.88	1.00	1.20	5.66	4.890
28.2	87.20	209.9	2.32	0.78	0.13	0.00	0.92	4.70	2.860
34.2	57.80	120.4	2.01	0.38	1.25	1.00	0.90	7.30	6.200
33.3	38.70	151.7	1.38	0.42	0.33	0.00	0.86	6.26	5.900
34.8	32.50	222.0	1.83	0.50	0.38	0.00	1.10	6.60	3.690
33.1	42.00	97.0	2.03	0.36	0.73	1.00	0.84	6.17	3.160
30.1	31.10	96.4	0.89	0.29	0.16	0.00	0.37	6.38	5.560
47.2	15.20	128.7	2.03	0.27	0.34	0.00	0.78	4.57	3.080
42.0	7.20	50.1	1.69	0.52	0.44	0.00	0.61	2.50	2.000
32.7	101.80	341.0	1.60	0.69	0.10	0.00	0.48	7.08	4.830
27.6	8.70	94.0	3.08	0.47	0.67	1.00	0.99	9.38	5.120
48.5	30.50	111.1	0.89	0.30	0.32	0.00	0.41	6.80	5.300
24.1	18.30	51.4	2.40	0.56	0.49	0.00	1.14	9.27	6.820
33.1	52.90	563.4	2.18	0.66	0.17	0.00	0.65	7.92	6.000
22.3	18.30	86.2	1.17	0.45	0.29	0.00	0.25	2.12	1.600
29.5	75.10	90.5	1.49	0.77	0.30	0.00	0.60	17.83	14.980
20.7	33.40	115.6	2.40	0.56	0.49	0.00	1.14	7.40	6.380
47.0	118.60	102.8	2.22	0.76	0.15	0.00	1.05	6.90	4.700
26.6	120.20	96.4	2.23	0.23	0.35	0.00	0.95	9.24	6.920
43.7	34.30	200.8	1.87	0.55	0.67	1.00	0.52	4.70	3.600
43.0	312.10	124.0	2.40	0.56	0.49	0.00	1.14	10.94	8.070
28.8	52.90	122.0	1.65	1.23	0.13	0.00	0.67	6.18	4.620
18.0	19.60	136.1	1.94	0.24	0.34	0.00	0.60	8.76	6.550
32.3	104.60	170.8	2.05	1.00	0.09	0.00	0.65	6.99	5.400
48.8	323.70	145.6	1.86	0.64	0.50	0.00	0.72	13.70	11.080
38.8	20.50	69.7	2.24	0.29	0.39	0.00	1.14	6.61	5.370
18.6	225.10	135.0	3.55	1.06	0.16	0.00	2.13	11.97	8.430
12.4	17.60	38.5	1.50	0.51	0.33	0.00	0.93	21.57	18.170
23.7	13.40	83.4	3.48	0.68	1.08	1.00	2.03	1.84	1.140
28.4	45.60	81.0	3.64	1.31	0.63	1.00	2.20	1.94	1.100

LY#	NLR	RBC	HGB	HCT	PLT	PT	PTA	INR	K
1.30	10.00	4.00	104.0	28.80	190.0	17.2	49.5	1.39	4.82
0.50	3.80	1.38	44.2	12.92	34.4	17.2	49.5	1.39	2.86
0.80	6.13	3.48	96.2	31.20	78.0	15.1	60.6	1.23	3.27
0.70	6.43	2.42	79.2	23.62	112.0	16.5	52.8	1.42	4.28
3.22	0.68	3.13	109.0	36.90	38.4	23.0	30.7	1.97	4.35
3.16	1.94	0.80	25.2	7.72	75.0	14.6	65.2	1.26	3.87
1.06	20.99	3.09	70.2	22.62	64.4	24.3	28.0	2.08	4.72
1.36	6.87	1.83	64.2	20.22	79.0	23.3	30.0	2.00	4.12
1.90	5.73	0.77	25.2	7.22	40.4	30.7	19.0	2.62	3.77
1.44	11.96	1.21	35.2	11.02	148.0	23.7	30.2	2.03	4.52
1.57	3.48	1.33	38.2	12.22	86.0	16.2	55.0	1.39	4.48
0.67	6.13	2.94	103.0	29.10	40.4	22.4	33.1	1.92	2.86
0.65	2.03	2.64	100.0	28.20	19.4	51.8	8.8	4.40	5.05
1.98	3.92	3.40	105.0	29.40	201.0	53.3	8.4	4.53	4.10
0.70	28.61	2.86	102.0	28.40	59.4	33.4	17.6	2.85	4.35
1.93	4.56	2.86	93.4	24.62	102.0	18.5	51.0	1.71	4.35
0.54	13.72	1.05	36.2	11.12	57.0	37.6	22.0	3.48	5.95
3.21	2.72	1.79	67.2	19.92	139.0	27.6	32.0	2.56	5.79
0.52	15.42	3.97	123.0	34.70	146.0	14.2	70.0	1.31	4.30
0.59	17.66	4.20	138.0	38.70	56.4	42.3	18.0	3.92	3.26
0.10	36.00	1.57	66.2	18.62	38.2	27.9	23.0	2.21	3.69
1.20	10.67	2.71	81.2	24.42	58.0	39.8	13.1	3.11	6.15
0.90	4.00	1.70	65.0	18.10	33.0	33.1	17.9	2.61	4.03
0.70	8.86	3.25	92.2	28.50	161.0	26.0	28.3	2.22	3.15
0.43	8.81	1.24	51.2	14.92	35.4	18.5	44.0	1.59	4.51
0.40	32.78	2.56	102.0	29.50	54.0	18.7	43.9	1.60	3.14
0.22	18.50	3.58	108.0	31.30	21.4	15.9	56.7	1.37	3.79
1.67	8.34	3.24	111.4	28.80	124.0	24.4	28.9	2.09	4.53
0.52	10.00	2.98	94.0	28.40	170.0	18.5	50.0	1.61	3.68
4.82	5.75	3.31	109.0	31.50	86.0	24.8	28.2	2.12	4.96
0.67	9.21	2.86	87.0	27.60	46.4	20.9	36.9	1.79	3.26
1.07	7.67	2.62	92.0	25.62	63.4	23.6	30.4	2.02	4.47
1.00	28.61	2.99	102.4	27.20	40.4	28.7	30.0	2.66	4.14
0.72	9.71	1.48	56.2	16.42	42.2	27.4	30.0	2.51	4.65
1.45	4.61	1.71	67.2	19.42	64.0	29.7	28.0	2.61	4.42
2.79	1.32	3.26	95.0	27.40	54.0	21.2	40.0	1.96	4.77
0.73	3.71	4.25	129.3	37.46	54.0	46.0	13.0	3.57	3.43
0.25	17.92	2.27	78.7	22.82	22.6	26.7	24.7	2.12	3.84
0.51	1.84	3.23	113.7	32.72	40.8	17.0	50.4	1.38	3.86
1.20	0.75	3.34	109.0	30.40	49.0	25.3	20.5	2.05	3.38
1.60	1.94	4.21	126.0	37.40	69.0	17.5	48.2	1.41	3.77
0.80	4.38	3.43	116.0	32.60	34.2	35.5	15.7	2.79	4.21
1.90	3.00	3.70	122.0	35.10	140.0	17.2	49.5	1.39	3.41
1.12	1.98	3.84	126.6	38.22	70.5	16.5	53.0	1.28	3.78

1.50	6.87	4.21	132.0	36.30	81.0	23.7	30.1	2.03	3.95
0.60	5.33	2.95	80.0	22.70	55.0	18.0	46.1	1.45	4.08
0.70	13.57	2.33	91.0	23.60	99.6	23.2	30.8	1.85	4.35
1.25	1.58	3.87	127.8	37.63	32.6	17.0	50.4	1.38	4.01
0.50	4.00	3.09	89.2	28.10	211.4	19.4	40.9	1.56	4.43
1.40	3.29	3.07	78.0	22.70	83.0	18.7	43.4	1.51	2.94
0.71	21.18	3.89	117.0	33.30	115.0	35.4	18.3	3.02	4.18
0.44	5.30	2.52	88.2	26.09	69.6	12.2	87.0	0.95	3.86
0.30	26.33	2.17	61.2	18.82	47.2	27.2	24.0	2.16	4.72
1.41	5.14	3.81	116.4	30.90	198.0	17.5	48.3	1.50	1.95
0.74	17.43	3.63	122.0	35.80	48.4	15.5	59.0	1.33	4.70
0.45	11.33	1.70	66.4	18.58	56.3	30.2	20.3	2.39	2.26
1.40	3.00	2.58	81.0	22.20	88.0	17.2	49.5	1.39	5.61
0.90	15.44	3.00	108.2	31.10	78.0	22.5	32.4	1.80	3.63
1.60	7.75	3.36	122.0	35.50	42.2	18.4	47.0	1.58	4.96
0.70	9.29	2.73	88.2	24.62	45.2	46.7	10.5	3.63	4.11
0.90	3.56	2.91	101.0	26.90	26.0	25.3	27.2	2.01	4.15
0.63	24.81	3.23	125.3	34.88	19.6	48.9	15.0	3.79	4.18
0.40	17.00	2.25	84.2	22.32	40.2	26.4	25.5	2.10	3.75
2.00	1.35	3.75	130.0	37.60	17.2	18.0	53.0	1.40	2.61
0.36	20.11	2.13	81.1	23.54	26.7	39.9	13.4	3.12	4.19
0.20	24.00	4.02	125.0	36.40	49.2	18.0	46.1	1.45	4.28
1.20	3.42	3.36	123.0	35.20	64.0	14.5	64.5	1.18	4.39
1.04	8.46	2.65	94.4	25.82	43.4	23.5	30.5	1.88	5.39
0.17	21.18	0.91	35.5	9.98	24.7	18.4	47.0	1.58	6.50
0.30	10.00	4.03	113.0	33.90	65.0	14.9	61.8	1.21	3.88
0.40	9.00	3.76	115.0	35.10	14.2	25.9	26.2	2.06	3.84
0.60	3.83	3.02	96.2	28.60	50.0	18.7	43.5	1.51	3.97
0.70	7.71	3.18	101.2	30.30	51.0	19.5	40.8	1.57	4.43
0.60	1.67	5.22	174.1	50.31	47.2	15.4	58.8	1.25	3.30
1.10	11.73	1.93	69.2	20.52	81.0	35.5	16.1	2.75	4.19
0.50	9.20	4.23	127.0	37.40	37.2	21.9	34.0	1.75	4.34
0.50	4.00	1.25	40.0	12.70	9.0	26.1	25.9	2.08	3.05
0.50	4.60	2.91	78.2	24.52	59.0	17.2	49.5	1.39	4.62
0.30	3.67	2.75	81.2	26.00	45.2	14.3	76.0	1.09	3.20
1.10	11.36	3.84	122.0	35.00	224.0	19.6	40.4	1.68	3.03
1.37	6.35	3.22	106.3	31.50	109.2	15.7	57.0	1.35	4.07
1.51	6.21	1.46	57.0	16.80	88.0	20.5	41.0	1.79	3.18
1.30	3.31	4.72	138.0	41.20	97.0	28.6	22.5	2.44	2.89
0.50	31.80	1.92	60.2	19.02	45.2	22.5	32.6	1.93	4.39
0.40	7.75	2.18	92.2	24.92	53.0	27.6	26.0	2.36	3.97
0.21	14.81	2.85	86.0	27.00	146.0	15.7	58.1	1.35	4.40
1.47	2.43	3.55	124.9	35.82	60.9	15.0	67.0	1.13	3.49
0.60	7.83	3.88	127.0	36.40	120.0	22.9	39.0	1.58	3.98
0.43	3.88	3.22	114.2	34.06	61.3	12.0	93.0	0.94	3.94

1.07	4.36	3.99	121.0	36.30	59.0	15.5	59.2	1.33	3.69
0.52	3.94	2.88	95.0	27.80	96.0	15.0	62.0	1.29	4.16
1.30	6.92	4.44	140.4	37.90	172.0	26.6	27.4	2.27	4.23
0.38	1.92	1.13	35.2	12.22	23.4	21.4	37.4	1.83	4.05
1.05	1.11	2.95	99.1	28.34	41.9	13.4	79.0	1.03	3.87
1.32	1.65	2.84	106.9	30.88	50.7	17.3	50.6	1.49	4.37
0.63	3.78	3.81	99.0	31.60	95.4	16.6	53.7	1.43	4.40
0.30	4.53	2.47	56.2	19.42	131.0	17.9	48.2	1.54	3.83
0.24	23.13	2.22	76.2	22.32	42.2	25.8	34.0	1.74	5.21
0.40	3.58	2.87	89.4	25.55	42.4	20.2	40.6	1.73	3.81
0.45	6.93	3.82	123.0	36.20	44.2	14.9	62.6	1.28	3.89
1.18	8.40	3.18	104.0	29.50	62.0	30.2	19.5	2.58	3.68
0.91	2.78	2.97	91.9	26.83	107.8	17.8	54.0	1.29	2.69
0.88	9.59	2.02	59.2	18.12	75.0	20.1	38.4	1.72	4.57
1.15	5.69	4.28	138.7	37.25	173.5	20.8	44.0	1.47	3.42
0.68	1.88	3.46	119.4	32.50	44.4	17.5	48.3	1.50	2.78
0.54	21.30	3.40	117.0	32.30	29.4	26.9	23.7	2.30	2.80
1.91	2.00	2.43	67.2	19.08	78.1	17.3	49.2	1.49	4.83
0.48	9.21	3.70	118.4	34.14	51.8	23.3	30.0	2.00	4.17
0.64	13.80	3.03	105.0	30.00	33.4	18.2	45.2	1.56	4.07
1.93	2.70	3.29	122.8	32.31	112.3	49.4	16.0	2.93	2.80
1.37	3.47	3.82	121.0	34.00	68.4	54.1	7.4	4.59	3.89
0.96	4.53	4.65	147.4	39.90	55.4	19.9	39.0	1.71	2.85
1.00	1.36	2.49	93.4	25.81	99.8	19.8	47.0	1.41	3.93
1.45	2.11	3.70	118.4	31.70	168.0	21.7	33.8	1.86	3.79
0.43	4.37	2.72	91.0	26.20	27.4	26.8	32.0	2.08	4.25
0.58	3.86	3.87	94.0	30.20	32.4	15.2	61.0	1.31	3.42
1.47	4.77	4.75	157.0	45.30	154.0	40.1	12.2	3.41	5.01
0.77	7.94	2.54	96.0	27.50	52.0	37.7	13.5	3.21	3.25
0.55	5.60	2.90	81.0	23.52	22.4	18.1	45.7	1.55	3.64
0.71	14.03	1.41	52.2	16.02	164.0	23.5	29.6	3.01	5.19
1.68	4.33	2.94	111.0	33.30	78.0	15.0	62.3	1.29	4.44
0.73	8.16	4.81	166.0	46.20	53.4	28.6	21.4	2.44	4.63
0.64	7.88	4.32	125.0	35.30	84.0	14.9	63.0	1.28	2.69
0.76	1.21	2.73	101.0	28.34	38.2	19.9	39.0	1.71	3.41
2.66	7.76	1.87	58.2	16.72	153.0	16.4	53.8	1.41	5.86
1.11	3.30	3.64	113.0	32.30	46.4	20.6	36.8	1.77	3.32
0.76	6.43	2.59	88.0	26.20	54.0	16.9	51.2	1.45	4.61
1.26	1.05	3.32	119.0	34.00	106.0	18.4	47.0	1.58	3.41
0.77	5.39	2.81	107.0	31.20	39.4	20.7	36.5	1.77	3.91
2.10	2.55	3.13	103.4	28.00	79.4	19.8	39.3	1.70	4.06
1.21	2.47	3.38	132.0	36.90	81.0	17.1	50.6	1.47	3.90
0.44	13.64	4.85	163.0	44.90	78.0	30.3	20.5	2.59	4.12
1.25	3.85	2.88	85.0	25.02	119.0	18.1	46.2	1.55	3.60
2.51	1.77	3.16	113.0	31.10	55.0	34.0	24.0	2.17	4.51

2.91	1.81	2.13	82.0	23.62	84.4	14.5	65.5	1.25	2.27
1.42	1.76	3.47	120.9	35.15	48.8	29.0	29.0	1.91	3.46
1.47	1.44	3.35	65.2	21.62	135.4	19.5	48.0	1.39	2.88
1.89	1.28	2.78	102.4	27.50	56.0	18.5	44.7	1.59	3.33
1.02	2.81	2.27	85.0	24.52	25.4	23.1	31.5	1.98	4.71
1.04	3.61	4.28	102.0	31.10	155.4	15.5	59.0	1.33	4.31
2.60	2.03	3.77	129.2	35.76	59.7	27.1	31.0	1.99	2.79
0.48	3.02	2.52	90.0	25.72	46.4	17.6	53.0	1.41	4.47
1.02	11.08	4.62	143.0	39.40	81.4	16.0	56.1	1.38	3.33
0.91	5.30	2.17	83.0	24.12	27.4	26.3	25.7	2.25	3.63
1.54	2.33	5.28	156.0	45.90	116.0	16.5	53.5	1.42	4.21
1.84	1.96	4.10	106.0	34.40	108.4	12.8	79.7	1.10	5.43
0.16	24.75	3.65	127.0	35.60	26.4	17.3	49.6	1.49	4.59
0.69	1.74	2.43	77.5	22.71	44.0	14.0	69.3	1.21	3.36
0.13	14.00	3.14	103.0	30.00	35.4	15.6	58.4	1.34	4.45
1.00	1.52	3.72	129.3	38.25	47.0	14.9	54.0	1.23	3.78
0.80	2.91	1.74	59.2	18.82	86.0	15.3	60.2	1.32	3.77
1.80	4.62	2.51	74.7	23.40	196.1	14.1	68.5	1.21	3.79
1.43	1.66	2.47	86.0	25.42	72.4	12.8	79.7	1.10	3.39
0.58	2.14	3.35	122.0	36.00	52.0	14.1	68.5	1.21	3.47
1.03	1.77	2.01	58.2	18.02	34.4	13.8	70.8	1.19	3.41
0.73	5.18	3.31	109.0	32.40	33.4	28.2	23.0	2.41	4.30
0.70	23.40	2.82	101.0	30.00	91.0	17.0	51.0	1.46	3.84
0.49	3.41	1.32	36.2	12.92	64.0	30.7	20.1	2.62	3.82
2.44	2.11	4.28	146.0	41.60	113.4	14.9	62.8	1.28	3.31
1.45	1.74	2.98	113.9	34.55	74.5	17.5	43.0	1.40	3.85
1.19	2.30	3.00	108.0	31.80	51.0	15.0	62.1	1.29	5.25
1.20	1.37	4.12	135.0	38.40	48.4	18.6	44.3	1.60	3.93
1.57	6.61	2.65	104.4	28.30	102.0	23.7	30.2	2.03	3.04
2.72	1.11	3.08	99.0	30.10	152.0	10.9	106.0	0.94	4.47
1.36	2.54	1.95	72.2	20.92	57.0	36.7	15.2	3.13	2.29
1.15	5.09	4.61	147.0	41.80	139.0	21.2	36.0	1.82	4.12
0.53	8.09	1.53	52.2	17.22	80.0	13.7	71.7	1.18	5.58
1.93	1.08	3.27	115.0	33.20	43.4	22.5	32.8	1.93	4.63
0.78	2.27	2.39	82.0	23.42	36.4	15.9	56.7	1.37	2.37
1.67	3.25	3.92	137.3	39.25	108.4	46.1	17.0	4.09	4.38
0.37	4.14	2.53	83.9	24.60	18.0	16.5	58.0	1.43	4.09
1.17	1.20	3.40	120.0	34.70	38.2	15.0	66.0	1.30	3.70
0.80	3.64	2.42	89.0	24.92	38.4	17.7	47.9	1.52	2.78
0.61	6.30	4.31	133.0	38.00	34.4	31.2	19.6	2.66	4.37
0.74	9.54	1.68	60.5	17.34	36.3	19.4	47.0	1.69	3.94
0.89	19.27	4.86	147.0	44.50	155.0	24.3	29.1	2.08	3.65
0.80	11.08	2.52	105.0	30.10	64.0	19.9	39.8	1.71	4.27
0.87	9.33	3.08	109.0	32.30	28.4	25.3	27.3	2.16	5.47
0.64	3.59	2.26	58.0	18.19	61.8	14.8	67.0	1.28	4.74

1.11	8.71	3.07	94.0	28.10	13.4	22.7	32.4	1.94	4.20
3.07	0.91	3.28	113.9	35.05	132.4	16.1	60.0	1.40	4.25
0.49	5.14	2.15	72.2	21.92	69.0	19.2	42.1	1.65	4.17
0.68	1.87	1.93	83.0	23.42	52.0	14.6	64.8	1.26	4.21
0.61	6.85	4.20	135.4	36.00	101.4	20.6	37.7	1.77	3.28
0.26	13.58	3.26	114.0	33.60	46.4	17.5	48.7	1.50	5.60
0.59	5.08	3.87	117.3	35.43	22.2	29.1	21.9	2.49	4.24
0.63	3.75	2.94	119.0	32.80	23.4	17.1	50.6	1.47	4.23
0.87	4.28	2.87	91.7	26.17	56.3	16.2	61.0	1.39	3.59
0.28	18.32	4.52	148.0	42.30	59.0	37.6	14.6	3.20	4.17
2.21	3.91	3.79	133.0	36.40	62.4	51.9	8.8	4.41	4.07
0.61	7.85	2.23	78.2	23.29	150.7	14.2	72.0	1.22	2.73
1.68	2.21	4.09	126.8	37.88	210.7	13.0	82.0	1.12	4.49
1.23	2.50	2.55	54.2	18.62	118.4	21.7	34.7	1.86	5.81
1.28	4.91	4.18	142.0	41.20	207.0	15.2	60.8	1.31	3.84
2.93	1.47	3.52	133.0	37.20	39.4	17.4	49.2	1.49	3.39
0.88	4.23	2.13	67.2	19.92	96.0	14.9	62.8	1.28	3.86
1.00	2.98	2.01	69.2	20.42	85.0	13.9	70.0	1.20	4.17
0.63	3.67	1.83	62.3	18.32	42.4	23.0	37.0	1.93	3.54
1.16	2.43	3.47	120.4	32.80	34.4	18.2	50.0	1.54	3.26
2.27	3.27	2.17	67.2	21.12	172.0	14.7	64.1	1.26	3.30
1.09	1.49	3.96	121.0	35.70	150.0	19.7	40.5	1.69	3.87
0.84	2.01	3.17	106.0	32.40	82.0	36.0	15.7	3.07	3.99
0.78	13.44	1.94	68.2	18.82	58.0	17.5	48.7	1.50	5.18
1.34	5.35	3.26	113.0	32.90	47.4	23.3	31.1	2.00	3.67
0.61	11.64	3.05	94.0	29.20	62.4	13.3	75.1	1.15	4.43
0.75	11.84	3.55	117.0	34.30	39.4	14.2	63.0	1.31	3.21
0.53	29.55	3.93	129.0	36.10	72.4	18.5	44.7	1.59	2.65
0.44	0.89	2.19	84.9	25.94	44.8	12.7	75.0	1.17	3.58
1.23	5.03	4.21	142.6	42.93	90.4	40.3	17.0	3.63	3.91
0.82	1.77	3.08	114.6	33.45	30.2	15.2	57.0	1.40	3.81
0.90	1.07	2.87	95.0	29.90	90.0	19.6	40.8	1.68	3.08
1.12	8.34	3.96	127.4	33.00	266.0	20.8	37.1	1.78	5.84
0.48	3.50	2.40	70.2	20.82	7.4	14.6	61.0	1.34	3.80
0.38	26.71	4.63	141.0	38.70	51.4	19.9	39.8	1.71	3.11
0.67	7.24	1.82	62.2	19.52	88.0	16.5	53.5	1.42	4.43
1.43	3.97	4.08	138.4	37.30	113.0	38.2	14.3	3.25	3.86
1.42	5.63	1.60	61.2	18.04	58.0	40.9	12.8	3.48	5.06
0.62	7.95	3.61	91.0	28.00	85.4	17.8	47.5	1.53	3.93
0.44	9.20	3.75	116.0	33.10	133.0	23.8	30.0	2.04	4.01
1.02	4.61	2.27	88.0	25.52	19.4	22.8	34.0	2.08	2.33
0.92	5.92	1.75	68.3	20.06	43.9	18.0	46.6	1.55	5.41
1.00	4.43	2.19	96.4	25.82	41.4	23.6	30.4	2.02	4.63
1.23	7.34	3.26	105.4	28.70	90.0	22.7	34.0	2.07	4.47
0.69	3.71	1.34	50.2	14.32	36.4	19.5	41.1	1.67	3.17

1.66	10.20	4.13	151.4	41.20	79.0	19.1	42.0	1.75	3.19
0.70	9.83	1.73	39.4	12.86	48.6	20.0	39.5	1.72	4.59
0.93	6.16	3.24	82.0	25.62	54.4	23.6	30.4	2.03	3.52
1.34	3.58	3.44	117.0	33.40	119.0	19.0	42.8	1.63	3.85
0.59	1.59	0.93	50.2	13.82	63.0	20.7	37.4	1.77	3.80
0.57	2.14	2.34	81.1	24.18	21.8	14.3	63.0	1.32	4.93
0.81	4.26	3.96	125.2	37.41	82.7	41.5	12.5	3.53	5.01
0.58	4.16	2.96	106.0	29.40	63.0	19.0	42.8	1.63	4.02
2.00	15.77	2.82	89.0	25.62	36.4	24.1	29.5	2.06	4.25
0.34	24.91	3.33	122.0	34.50	62.0	22.8	34.0	2.08	4.47
3.23	2.75	4.47	142.0	39.50	329.4	17.4	52.0	1.61	2.82
0.44	21.00	1.87	62.2	18.12	50.0	22.6	32.6	1.94	4.84
1.36	1.28	2.34	74.2	22.52	65.0	17.9	47.0	1.54	3.98
1.57	1.45	4.71	147.0	43.10	45.4	14.0	69.3	1.21	3.33
0.38	28.13	3.31	99.4	27.00	180.4	18.0	52.0	1.67	4.48
1.71	1.91	2.72	102.0	29.10	64.0	17.1	53.0	1.58	3.00
4.40	0.75	3.90	136.4	34.90	168.0	43.7	18.0	4.05	4.14
1.43	3.05	4.16	130.0	38.70	90.6	45.0	17.0	4.17	3.14
1.31	5.02	1.85	69.2	19.62	93.0	18.3	45.4	1.57	5.42
1.18	2.57	2.22	77.5	23.63	42.4	15.1	61.5	1.30	4.29
0.74	5.39	2.32	91.0	25.42	29.4	20.5	38.0	1.76	3.79
0.66	2.38	3.36	110.0	33.10	52.0	14.0	69.3	1.21	3.80
0.78	3.21	3.13	110.0	32.10	51.0	15.0	62.1	1.29	4.97
2.08	1.46	3.53	114.4	30.40	94.0	44.2	18.0	4.09	4.20
0.54	23.78	1.99	73.2	20.72	321.0	18.5	44.7	1.59	3.72
0.30	31.10	1.93	78.2	23.72	48.4	21.0	36.6	1.80	5.14
0.91	7.27	4.69	152.0	41.80	107.0	13.4	74.2	1.15	3.85
1.32	3.26	3.52	120.9	37.05	135.4	13.7	71.0	1.27	3.53
0.22	5.05	1.79	50.2	15.22	21.4	14.1	68.5	1.21	5.02
0.66	24.32	2.75	96.4	25.82	37.4	21.8	34.5	1.87	1.41
0.27	6.37	2.17	77.2	23.32	35.4	18.1	49.0	1.68	3.46
0.35	12.37	3.54	105.0	31.70	49.4	18.4	47.0	1.58	3.93
1.74	5.99	1.98	70.2	20.12	65.0	18.3	45.4	1.57	4.18
1.22	2.66	3.98	128.4	34.90	33.4	25.5	33.0	2.36	3.98
0.51	6.71	2.25	54.2	17.02	105.0	14.5	65.5	1.25	2.46
1.66	2.95	4.91	149.0	41.90	86.0	24.1	29.5	2.06	3.59
1.23	0.65	1.79	73.2	20.12	76.0	16.9	51.5	1.45	3.84
0.78	0.94	4.21	143.0	40.70	80.0	16.3	56.0	1.51	3.46
0.25	22.28	2.73	84.0	26.80	24.2	24.7	28.3	2.11	6.73
0.35	6.40	1.86	69.4	18.52	24.4	44.3	17.0	4.10	3.03
1.12	4.69	2.21	60.2	18.02	44.4	15.0	62.1	1.29	3.63
0.66	9.94	2.59	83.0	23.72	55.0	22.9	31.9	1.96	4.27
1.38	11.14	2.51	83.0	23.52	214.0	14.6	64.8	1.26	3.87
1.87	4.25	3.59	120.0	34.30	133.0	29.0	22.0	2.48	4.57
0.25	8.20	2.04	66.4	17.02	66.0	27.7	23.7	2.37	3.31

1.44	6.52	2.67	91.4	24.32	92.0	17.5	48.7	1.50	5.72
0.58	2.00	3.87	114.0	37.20	20.0	17.8	50.0	1.65	2.56
0.45	26.02	3.18	119.4	32.10	155.0	23.5	36.0	2.18	1.71
0.41	11.05	1.67	55.2	16.02	65.0	19.0	48.0	1.76	4.24
1.00	6.35	3.74	123.0	36.60	69.0	29.2	28.0	2.70	3.08
1.17	14.55	2.21	78.4	21.32	84.0	16.4	60.0	1.52	4.90
1.40	2.71	2.64	71.0	21.90	205.0	14.3	67.0	1.32	3.62
0.67	4.25	1.52	52.4	13.92	73.0	31.4	27.0	2.91	3.80
1.04	10.05	3.52	105.0	30.30	37.4	30.2	29.0	2.80	4.05
0.82	5.67	2.28	83.0	25.12	32.4	22.1	41.0	2.05	6.06
0.95	1.85	3.31	110.0	32.50	54.0	20.8	44.0	1.93	3.29
1.21	7.40	1.17	47.2	14.62	18.4	31.8	27.0	2.94	3.31
0.77	1.51	1.38	46.2	14.22	44.4	42.1	20.0	3.90	4.04
3.55	2.40	2.91	102.4	27.40	70.0	50.5	16.0	4.68	4.37
0.95	2.51	2.50	89.0	25.22	35.4	24.3	37.0	2.25	3.76
1.31	3.67	4.75	163.4	43.80	141.0	37.9	22.0	3.51	2.95
0.62	4.19	2.66	99.4	26.50	72.0	28.5	31.0	2.64	3.46
1.22	4.29	5.05	162.4	44.30	127.0	33.2	24.0	3.04	4.27
1.55	4.66	3.02	111.0	31.00	52.0	28.6	30.0	2.65	3.56
0.60	12.15	4.12	135.4	36.90	122.0	62.8	13.0	5.81	4.21
1.64	1.43	2.70	65.2	19.52	134.0	14.8	69.0	1.37	3.58
0.79	7.13	1.74	66.4	17.72	43.4	36.7	23.0	3.40	5.09
1.34	6.54	3.00	117.4	30.20	212.0	29.7	29.0	2.75	2.47
1.12	4.50	5.61	187.0	53.60	77.0	21.4	41.0	1.97	3.50
1.25	0.74	3.83	122.0	35.50	67.0	19.6	48.0	1.81	3.55
1.08	3.70	3.43	110.0	29.90	67.0	23.8	38.0	2.20	4.16
0.99	5.51	3.27	114.0	31.70	84.0	21.1	44.0	1.95	4.05
0.69	3.04	3.83	133.0	36.80	28.4	21.0	45.0	1.94	3.26
0.67	20.72	2.65	84.0	23.62	73.4	15.2	66.0	1.33	4.13
1.66	4.35	4.59	152.0	43.10	123.0	26.1	34.0	2.42	4.00
0.72	8.71	2.67	95.0	27.00	70.0	20.6	46.0	1.91	2.58
1.14	4.06	3.40	104.0	29.50	119.0	17.2	58.0	1.59	3.21
0.44	2.11	1.79	61.2	18.62	23.2	28.5	31.0	2.64	3.01
0.59	3.32	2.29	74.2	21.52	48.1	28.8	31.0	2.67	3.56
1.95	1.03	2.95	108.0	30.50	64.0	19.6	46.0	1.71	4.19
0.79	27.92	1.94	77.4	20.32	125.0	48.8	17.0	4.52	4.01
0.72	9.67	2.11	71.2	20.62	41.4	16.1	64.0	1.49	4.29
0.83	11.33	1.82	52.0	16.70	260.0	20.4	44.0	1.78	6.67
3.38	3.45	3.85	137.0	37.70	131.0	25.5	33.0	2.24	4.39
0.88	3.94	3.07	112.0	32.20	74.0	20.3	47.0	1.88	2.68
0.52	3.62	2.49	91.0	26.60	65.0	19.5	49.0	1.81	4.17
0.30	7.57	2.94	96.0	25.96	14.0	18.8	52.0	1.74	3.15
0.69	1.90	2.41	93.0	26.00	45.4	18.4	53.0	1.70	4.09
0.76	5.39	4.06	143.0	39.20	64.4	11.9	99.0	1.10	4.14
1.20	2.92	2.42	56.2	17.72	96.4	16.8	60.0	1.56	4.23

3.19	0.65	2.35	88.6	25.83	81.5	21.9	40.0	1.92	2.80
1.27	4.35	4.17	134.0	37.30	70.4	66.1	11.0	5.85	3.64
0.56	19.20	4.15	143.0	42.80	34.0	29.2	28.0	2.56	4.27
1.45	17.33	2.19	86.0	24.52	264.0	15.8	65.0	1.46	5.63
0.47	22.77	1.58	50.2	14.82	108.0	21.8	43.0	2.02	4.81
0.61	10.85	1.99	81.0	23.30	41.0	24.6	35.0	2.16	3.00
0.56	8.84	3.32	127.0	35.50	27.4	32.0	27.0	2.96	2.92
3.21	5.84	1.62	57.1	15.32	130.6	19.3	47.0	1.69	4.30
1.13	3.81	4.71	149.0	41.00	81.4	56.9	14.0	5.27	4.54
1.45	15.81	4.08	128.0	37.10	124.4	17.4	57.0	1.61	4.99
1.80	7.34	1.19	37.2	11.52	145.0	15.2	69.0	1.41	3.53
0.63	4.54	2.65	91.0	26.00	84.0	17.6	56.0	1.63	3.34
0.23	2.78	2.16	47.2	15.52	27.4	19.5	49.0	1.81	4.27
0.75	4.13	2.88	104.0	30.00	45.4	12.9	82.0	1.19	3.17
0.79	3.35	3.87	131.0	38.00	148.0	22.7	37.0	2.10	3.75
0.45	8.02	2.98	108.0	31.90	55.0	21.1	39.0	1.85	4.00
1.17	3.85	4.59	143.0	40.50	98.4	26.3	31.0	2.44	3.39
0.76	4.34	3.24	101.0	29.30	86.4	14.7	67.0	1.36	5.08
1.07	2.43	2.02	60.2	19.02	87.0	14.7	67.0	1.36	2.78
0.45	11.78	3.24	113.0	31.30	131.0	15.9	60.0	1.47	4.57
1.28	1.88	3.59	120.9	35.14	67.8	15.0	64.0	1.31	4.28
1.96	2.05	3.76	114.0	34.90	330.4	10.0	132.0	0.87	3.77
1.96	3.37	4.80	146.0	42.20	106.0	20.9	40.0	1.83	5.27
1.72	8.80	2.43	81.0	23.72	51.0	35.6	22.0	3.30	4.75
1.18	1.10	3.06	92.0	26.20	34.4	21.0	41.0	1.94	3.12
0.91	2.80	2.71	86.9	25.27	72.1	16.5	57.0	1.53	4.20
0.37	33.49	1.99	54.2	18.02	97.0	26.4	31.0	2.44	4.26
1.01	11.38	3.00	92.4	25.02	43.4	29.6	27.0	2.74	3.99
0.12	17.75	1.84	66.2	20.82	30.0	51.5	14.0	4.77	3.19
0.81	1.62	2.32	81.0	23.80	41.0	33.7	22.0	2.96	3.49
0.86	8.87	2.35	66.2	21.32	71.0	27.2	33.0	2.52	4.09
3.43	1.75	3.50	110.4	29.30	201.0	14.4	73.0	1.33	4.64
0.91	3.34	4.26	152.0	42.90	69.0	19.4	50.0	1.80	5.04
0.74	3.54	4.12	126.0	36.30	74.0	16.0	63.0	1.48	3.81
0.51	33.31	3.62	133.0	38.70	90.4	23.9	38.0	2.21	5.39
1.05	2.03	3.86	136.0	37.80	46.0	19.1	50.0	1.77	4.05
1.43	7.57	2.84	90.0	26.60	193.0	13.7	78.0	1.27	3.11
0.66	5.09	3.77	127.0	36.00	30.0	22.7	41.0	2.10	3.47
2.37	0.88	3.03	70.0	23.20	24.2	13.5	78.0	1.17	2.99
1.86	7.89	2.72	81.0	23.90	96.0	17.5	56.0	1.62	4.51
0.82	4.77	1.83	68.2	19.22	50.0	29.1	31.0	2.69	4.18
1.39	3.05	1.49	45.0	13.70	75.0	22.8	41.0	2.11	3.76
1.18	12.35	3.85	99.0	29.60	127.0	14.3	74.0	1.34	3.50
2.65	0.89	2.55	89.0	25.02	134.0	22.4	42.0	2.07	3.74
0.89	3.02	3.21	74.2	24.52	80.4	18.6	48.0	1.72	3.25

1.60	3.41	1.29	49.2	14.02	52.0	43.9	18.0	3.72	4.37
2.26	2.41	1.34	50.2	14.75	61.6	17.2	55.0	1.48	3.78
1.89	1.24	3.31	105.0	30.10	109.0	42.0	18.0	3.89	3.49
3.14	0.36	3.80	93.0	28.70	273.0	24.4	34.0	2.26	3.53
1.38	5.22	2.61	94.0	27.00	95.0	23.7	35.0	2.19	4.68
1.19	6.50	6.00	199.1	56.91	24.4	19.6	45.0	1.81	4.75
0.85	0.76	3.27	122.0	34.80	29.0	15.3	65.0	1.32	3.78
0.57	5.56	2.90	103.4	27.30	77.0	26.7	32.0	2.28	4.64
0.44	7.86	3.38	103.0	30.40	244.0	11.0	108.0	0.96	5.10
0.86	4.05	3.47	137.0	38.40	49.4	21.9	39.0	2.03	3.89
0.66	12.95	3.50	103.0	35.00	79.0	16.0	59.0	1.48	4.13
1.20	12.33	3.22	117.0	30.70	99.0	15.5	58.3	1.26	4.41
2.30	4.10	1.37	57.2	16.22	86.0	19.4	41.4	1.66	6.29
0.36	30.86	2.10	72.2	20.82	49.0	21.4	35.5	1.83	5.27
1.26	10.19	1.38	39.2	12.92	37.4	19.8	40.0	1.81	4.26
0.40	23.03	3.84	96.5	31.53	95.8	16.2	57.0	1.49	2.85
0.41	4.20	1.87	65.2	17.82	14.4	21.9	42.0	2.03	3.26
1.00	6.20	3.05	98.0	27.30	57.0	19.8	39.8	1.59	3.40
1.36	7.82	4.29	155.4	41.50	73.0	15.3	60.2	1.32	3.78
0.54	7.91	1.88	68.4	18.62	70.0	18.4	45.0	1.58	5.08
1.79	5.25	4.08	124.0	34.90	159.0	15.0	64.0	1.31	4.93
1.45	2.03	2.49	76.2	22.82	170.0	14.8	66.0	1.37	4.32
0.82	5.90	3.41	75.2	23.80	47.6	26.4	25.1	2.10	4.50
0.26	3.27	2.64	71.2	22.92	24.4	21.4	35.5	1.83	4.13
1.75	5.31	5.03	162.0	48.40	106.0	27.4	24.1	2.34	4.66
2.10	2.62	3.43	125.0	36.50	61.0	26.0	32.0	2.41	2.37
0.87	10.55	2.18	56.2	17.22	60.0	14.9	66.0	1.38	4.17
0.25	4.20	1.12	34.2	10.32	8.4	20.1	39.2	1.72	4.95
0.22	3.00	4.06	98.0	30.40	30.4	17.1	54.0	1.58	4.15
1.39	2.65	2.34	74.1	21.51	107.0	13.5	80.0	1.25	4.73
0.55	8.38	2.23	86.0	24.70	57.0	30.4	29.0	2.81	4.55
0.50	24.80	2.44	91.2	26.62	30.2	24.2	28.9	1.93	4.16
0.30	14.67	2.34	90.2	24.02	33.2	21.5	42.0	1.67	5.89
0.63	1.94	3.70	128.0	36.50	56.0	20.6	37.7	1.77	3.62
0.38	9.26	2.43	90.0	26.92	28.2	39.0	20.0	3.50	3.75
0.92	10.91	2.49	71.2	21.32	264.0	14.7	67.0	1.36	3.26
1.50	1.07	3.26	106.0	28.80	190.0	15.0	61.2	1.22	3.52
1.12	10.32	2.46	87.0	24.82	98.0	16.8	51.7	1.44	4.11
0.33	8.18	1.65	65.2	19.32	20.4	20.1	39.2	1.72	4.39
0.65	18.22	2.72	96.4	26.10	138.0	22.2	38.0	2.06	2.93
1.90	5.05	1.87	76.0	22.22	163.0	18.6	44.3	1.60	3.97
0.60	7.58	2.48	80.0	23.32	29.4	16.5	60.0	1.53	3.58
0.21	12.71	1.55	57.0	16.80	20.0	29.6	29.0	2.53	4.41
0.80	1.75	2.53	82.2	24.52	51.0	18.3	45.0	0.48	3.97
3.91	0.95	4.19	137.5	38.93	143.9	32.4	17.4	2.77	3.21

1.33	2.35	3.48	116.0	32.30	160.0	34.3	25.0	3.18	3.15
1.05	2.74	2.77	102.0	28.60	200.0	16.4	62.0	1.52	5.11
1.88	2.05	3.58	119.5	35.45	86.4	20.5	37.1	1.76	4.11
1.64	3.63	3.75	124.4	33.00	101.4	20.8	37.1	1.78	4.71
1.16	8.31	1.82	79.2	22.52	111.0	15.0	62.1	1.29	2.82
0.97	1.70	3.25	102.0	30.50	83.4	18.4	45.0	1.58	3.06
0.92	3.89	4.35	142.4	37.80	72.4	51.9	16.0	4.81	3.66
1.00	3.99	2.23	71.2	20.12	94.0	16.3	59.0	1.42	3.06
0.53	4.49	1.99	53.0	17.40	55.0	27.7	32.0	2.56	3.54
0.67	2.10	2.30	75.0	22.26	48.8	19.5	45.0	1.65	4.05
0.81	2.89	2.34	84.0	25.80	57.0	21.8	38.0	1.91	2.62
1.57	2.15	4.42	137.7	40.63	121.0	19.3	48.0	1.66	3.64
0.48	4.35	2.89	92.0	31.90	49.4	15.9	59.0	1.47	4.26
0.49	15.33	3.13	113.0	32.60	57.0	22.3	33.3	1.92	3.71
0.88	5.53	2.69	105.7	31.40	16.7	42.1	19.0	2.58	3.84
1.93	3.80	4.89	164.8	47.30	87.3	17.6	52.0	1.49	3.57
0.90	4.94	3.08	82.0	25.72	57.4	14.4	66.2	1.24	3.98
1.00	1.85	4.00	120.0	36.30	45.0	14.3	67.0	1.32	3.80
0.41	4.44	2.31	80.0	22.32	56.4	13.6	81.0	1.26	4.53
0.92	4.71	4.59	141.0	38.70	113.0	25.1	33.0	2.32	2.83
0.90	2.89	3.44	96.1	28.94	90.8	15.5	58.3	1.26	4.59
0.84	3.14	3.52	120.0	33.00	42.4	25.3	27.3	2.16	3.07
0.60	8.67	2.48	94.2	28.00	12.4	23.7	29.8	1.89	3.84
0.54	2.22	2.30	99.2	26.50	48.2	20.2	38.6	1.62	3.63
2.42	6.17	3.63	128.0	35.60	98.0	17.4	49.2	1.49	4.15
0.74	12.72	4.53	138.4	37.70	207.0	27.3	24.2	2.33	3.68
0.81	6.86	1.42	35.2	12.22	62.0	15.1	62.0	1.40	3.82
0.41	8.66	3.63	84.0	25.42	54.4	20.4	43.0	1.88	3.73
0.52	21.04	3.00	112.0	31.70	41.4	14.5	65.9	1.25	4.51
0.73	11.34	3.41	123.4	33.00	27.4	22.9	31.9	1.96	4.44
0.66	8.58	3.14	104.0	29.60	145.0	15.8	57.2	1.36	4.32
1.34	2.19	1.67	60.0	17.90	61.0	14.4	71.0	1.25	3.49
1.20	3.00	3.20	96.0	25.80	61.0	17.8	46.9	1.44	3.32
0.75	8.77	2.79	104.4	31.26	109.0	16.1	54.9	1.31	3.18
2.65	4.88	1.66	53.8	16.56	68.1	41.3	12.7	3.22	5.41
0.41	9.59	2.49	93.3	27.35	64.2	17.9	46.5	1.44	4.33
0.58	4.83	3.89	118.0	32.30	52.3	14.0	74.0	1.07	3.89
0.67	2.03	3.40	113.0	33.80	70.0	15.4	59.7	1.32	4.61
1.41	1.91	3.29	112.0	30.90	42.0	16.9	57.0	1.45	3.63
0.20	15.00	3.45	118.0	35.20	39.2	19.6	40.4	1.58	5.37
0.32	5.03	3.38	91.0	28.60	32.4	17.7	54.0	1.29	4.68
0.82	15.21	2.89	110.0	30.80	79.0	25.3	27.3	2.16	4.05
1.12	5.42	3.63	120.6	35.30	101.4	39.4	13.7	3.08	4.16
0.32	20.38	2.54	107.5	30.03	79.2	14.9	68.0	1.12	5.02
0.86	4.33	2.14	81.0	23.02	91.0	25.8	25.4	2.21	3.86

1.33	4.64	3.34	89.5	27.37	60.8	22.2	40.0	1.54	2.98
0.90	3.91	3.74	117.4	30.80	52.4	35.3	21.0	3.10	4.08
2.54	3.05	1.64	65.0	18.40	79.0	25.5	33.0	2.36	2.55
1.10	1.09	2.65	94.2	27.10	91.4	16.3	47.2	0.31	3.14
1.01	5.02	3.32	125.4	32.40	57.0	21.4	35.5	1.83	3.15
0.77	1.44	3.96	96.0	29.70	138.0	15.5	61.0	1.35	3.12
0.80	8.13	2.25	80.0	20.90	88.0	17.6	40.8	1.42	3.61
1.07	2.36	2.31	83.0	24.32	73.0	25.3	26.2	2.16	3.86
0.59	14.10	2.99	102.0	28.00	148.0	23.2	40.0	2.15	3.67
0.70	2.43	3.15	106.2	31.80	41.2	19.8	39.8	1.59	4.41
0.90	24.33	3.70	100.0	28.40	178.0	14.2	66.6	1.16	5.01
1.20	2.08	3.09	118.0	34.30	61.0	19.1	42.1	1.54	4.47
0.78	13.26	4.05	136.6	38.84	84.4	28.1	31.0	1.86	4.75
0.29	3.31	3.35	63.1	20.89	40.7	14.7	64.1	1.26	3.84
1.11	2.13	2.99	67.2	22.12	287.0	14.1	72.0	1.31	3.70
1.00	4.19	3.21	95.0	28.70	172.0	18.7	48.0	1.73	3.43
0.61	6.49	1.49	62.2	17.89	45.7	54.4	8.0	4.20	4.63
0.90	2.22	3.95	107.2	34.80	75.0	23.6	30.3	1.88	3.36
0.31	18.65	2.74	87.1	27.23	66.3	13.3	74.0	1.23	3.88
0.85	3.92	2.80	111.4	30.20	76.0	17.9	47.0	1.54	3.61
0.92	5.68	3.69	127.0	35.50	127.0	24.3	29.1	2.08	3.50
0.26	13.31	2.10	46.2	15.82	55.4	22.4	38.0	2.07	4.10
1.28	2.53	4.10	129.3	36.20	52.8	26.6	24.1	2.27	3.39
0.20	5.50	2.92	89.0	26.40	23.0	14.7	68.0	1.36	3.67
1.52	11.34	2.74	100.0	28.50	268.0	28.7	30.0	2.66	4.56
2.40	1.49	1.47	47.2	14.82	93.0	22.0	34.0	1.88	3.47
1.36	3.43	4.17	128.0	35.20	182.0	30.6	28.0	2.83	4.20
0.31	9.13	2.08	59.7	17.31	48.7	14.2	73.0	1.23	4.14
0.47	19.36	2.91	90.4	24.42	47.4	15.9	56.7	1.37	3.50
1.56	2.79	3.54	123.4	32.90	107.0	45.4	17.0	4.20	4.37
1.51	6.30	3.40	117.0	32.46	75.4	29.7	20.1	2.54	4.74
3.70	1.09	3.91	135.0	38.40	135.0	16.9	51.2	1.45	4.99
0.75	1.87	2.90	98.0	28.00	86.0	16.6	53.0	1.43	3.09
0.76	5.86	2.87	104.0	29.40	73.0	17.1	54.0	1.58	4.32
0.90	3.78	1.54	60.2	17.02	53.0	30.2	24.0	2.34	4.68
1.00	5.00	3.99	123.0	35.30	115.0	20.8	36.7	1.67	5.28
0.67	13.85	1.14	48.2	13.45	42.3	29.2	21.8	2.49	2.65
1.10	2.64	2.65	87.6	26.25	29.2	23.5	30.7	2.01	5.54
1.86	4.85	3.98	131.4	34.20	107.4	29.8	27.0	2.76	4.10
1.49	2.84	2.50	104.0	28.70	67.0	32.7	25.0	2.88	4.33
0.68	2.49	4.61	103.0	31.10	154.4	16.3	54.5	1.40	3.39
0.17	3.00	2.04	53.5	17.38	45.6	15.3	60.2	1.32	3.97
0.91	3.47	2.20	74.3	21.98	61.3	25.3	30.0	2.30	3.96
0.80	3.38	3.72	114.0	33.70	40.2	27.4	23.7	2.17	3.52
1.90	4.63	1.54	64.2	19.12	178.0	15.7	57.0	1.27	3.37

0.98	2.53	2.82	97.0	27.30	35.4	28.0	22.1	2.39	3.71
1.22	4.89	1.76	54.6	15.92	138.6	13.4	74.2	1.15	4.12
0.82	7.30	2.92	98.0	27.00	16.4	27.1	30.0	2.51	4.07
0.49	1.18	3.62	85.0	28.10	54.4	16.1	55.4	1.38	3.58
1.76	5.63	1.45	47.2	13.62	54.0	23.1	35.0	2.02	3.95
1.23	10.52	1.60	50.2	14.22	64.0	21.9	39.0	2.03	4.26
0.88	1.42	3.46	84.0	26.60	59.4	15.1	64.0	1.40	4.21
0.93	9.04	1.67	65.2	18.62	26.4	41.7	19.0	2.80	4.12
0.78	6.53	3.06	97.0	28.10	71.0	14.1	71.0	1.31	3.74
1.06	2.47	3.02	105.1	31.51	41.6	18.3	44.8	1.57	3.90
0.18	11.89	2.50	88.0	26.10	24.7	16.2	55.0	1.39	4.76
0.70	1.71	3.17	105.2	30.20	51.0	20.2	38.4	1.62	3.45
0.52	3.44	2.60	96.7	26.95	11.8	24.8	28.1	1.98	3.92
0.78	3.27	3.20	108.0	32.20	90.0	14.6	64.8	1.26	4.40
0.80	5.75	3.52	118.0	33.60	29.2	14.9	62.1	1.21	4.19
0.53	4.21	4.57	104.0	32.70	67.4	16.3	54.5	1.40	4.31
0.14	26.29	1.92	78.5	22.57	25.1	27.2	24.3	2.16	2.61
0.86	2.95	3.50	113.0	32.00	81.0	23.3	33.1	2.00	3.33
1.26	1.85	3.52	123.4	32.30	54.0	16.4	53.8	1.41	4.09
1.21	1.31	3.55	101.0	33.96	48.8	15.2	57.0	1.40	3.43
0.80	8.63	2.86	112.0	31.80	54.0	23.5	30.5	2.01	2.55
0.59	5.66	4.53	134.0	39.10	79.4	21.8	39.0	2.02	4.83
2.53	1.06	2.74	97.6	28.91	150.6	11.2	104.0	1.04	4.07
2.10	1.95	4.52	158.4	42.80	58.0	15.1	66.0	1.32	3.13
2.42	0.50	2.91	101.0	28.80	149.0	18.0	54.0	1.67	3.54
0.70	4.00	2.92	96.0	24.70	98.0	14.8	56.6	1.19	3.29
1.10	1.27	4.08	130.0	35.20	59.0	30.2	20.3	2.39	3.39
0.20	14.70	2.12	76.9	22.24	75.0	25.8	33.0	2.00	3.57
1.34	2.01	2.95	107.0	30.80	65.0	22.5	40.0	1.93	3.62
1.05	1.92	4.93	155.0	43.50	98.0	34.7	24.0	3.21	3.16
0.84	5.82	4.59	137.4	35.70	148.0	40.3	21.0	3.73	2.59
1.23	2.34	3.04	109.0	30.60	76.0	27.1	31.0	2.51	3.26
1.80	2.94	4.73	139.0	41.30	96.0	35.1	16.0	2.76	3.37
1.76	0.81	3.47	124.0	35.70	88.4	15.0	62.1	1.29	3.72
0.70	9.43	2.82	101.2	28.40	159.0	20.0	39.0	1.61	3.78
2.41	0.66	2.72	107.4	29.10	50.0	23.3	39.0	2.16	4.12
1.00	7.46	2.08	84.4	21.32	56.0	21.0	44.0	1.94	5.94
0.40	7.43	2.82	87.0	25.32	41.4	14.1	68.5	1.21	4.22
1.48	3.14	3.48	122.4	35.74	61.2	14.5	68.0	1.34	3.57
1.10	6.36	3.43	107.0	28.70	93.0	26.8	18.4	2.18	3.42
1.03	5.54	1.76	46.0	15.50	51.0	15.6	63.0	1.36	4.30
2.50	1.74	3.96	141.7	40.59	120.1	12.4	88.0	0.97	2.97
1.06	6.66	3.44	108.0	31.60	123.0	13.4	74.2	1.15	4.73
0.97	1.35	3.18	115.0	34.40	63.0	18.9	49.0	1.65	3.89
0.56	21.96	4.55	130.0	38.70	94.0	14.6	64.8	1.26	4.92

1.12	4.04	1.58	64.2	18.22	35.2	30.5	26.0	2.82	3.53
0.76	3.54	3.07	114.0	33.50	42.4	23.6	30.4	2.02	3.55
0.56	1.93	3.02	107.0	30.60	56.0	19.6	45.0	1.81	3.81
0.97	3.46	3.12	106.0	29.60	29.4	21.1	36.3	1.81	4.26
0.40	4.75	2.93	69.2	22.92	36.2	20.7	37.2	1.66	3.37
1.08	1.63	4.01	138.0	40.20	73.0	16.3	54.5	1.40	3.80
0.87	2.29	2.13	62.2	18.42	84.0	18.4	47.0	1.58	3.44
1.20	2.25	2.79	92.2	27.00	80.0	15.3	59.4	1.24	4.10
0.26	8.65	2.39	61.2	19.12	59.4	24.5	37.0	2.27	6.10
1.10	1.56	3.19	112.0	31.80	42.4	23.5	30.7	2.01	5.43
1.12	1.69	3.97	132.0	37.70	98.0	18.7	48.0	1.73	4.25
0.61	4.43	0.95	31.6	9.19	46.2	22.2	40.0	1.69	4.21
0.30	4.33	2.20	62.0	18.70	13.0	25.5	33.0	2.36	3.27
1.58	2.17	3.32	119.0	33.70	107.0	20.9	44.0	1.94	3.70
0.82	7.55	1.53	51.2	15.52	49.4	18.5	44.7	1.59	3.53
0.86	4.76	2.80	110.4	28.80	96.0	15.7	57.8	1.35	3.35
0.86	4.76	2.80	110.4	28.80	96.0	15.7	57.8	1.35	3.35
0.53	3.42	2.30	73.2	23.12	23.4	19.9	39.8	1.72	5.11
0.49	8.71	3.02	113.0	32.80	28.4	24.7	36.0	2.29	4.35
1.18	1.46	3.18	110.5	33.17	131.5	14.0	72.0	1.21	4.14
0.73	3.18	1.88	68.2	20.12	109.0	14.6	61.0	1.34	4.69
1.16	7.12	4.16	143.0	39.80	51.0	18.7	48.0	1.73	3.66
0.83	2.46	2.20	50.2	19.52	106.0	14.1	68.5	1.21	3.72
0.63	3.38	3.41	120.0	34.90	88.0	14.6	64.8	1.26	3.66
0.92	1.83	4.05	146.6	41.72	32.1	16.6	52.7	1.43	3.95
1.27	3.81	4.41	139.0	40.10	51.0	10.2	128.0	0.94	3.83
1.04	3.35	3.12	118.4	32.10	87.0	23.9	33.0	2.09	3.00
0.62	4.85	3.13	112.0	32.90	102.0	17.8	47.5	1.53	3.67
1.08	2.96	3.15	115.2	33.74	122.9	15.6	66.0	1.21	4.38
0.56	6.70	2.72	96.0	30.00	219.0	14.5	65.5	1.25	6.03
0.59	4.90	2.30	78.2	23.52	36.4	18.5	49.0	1.71	4.61
0.90	7.50	3.77	117.0	33.20	211.0	16.9	59.0	1.56	3.42
0.51	2.75	2.70	87.0	25.50	69.0	15.7	62.0	1.36	3.98
2.70	3.67	3.27	105.2	31.80	86.0	19.3	41.4	1.66	3.85
0.80	6.75	2.88	107.0	29.90	24.0	20.7	42.0	1.92	2.80
1.10	4.18	2.72	108.2	29.80	23.2	20.9	36.6	1.68	3.38
0.69	3.58	2.56	96.0	28.10	55.0	20.1	39.2	1.72	4.00
3.52	0.92	4.85	156.0	45.00	213.0	12.2	90.0	1.13	4.27
1.87	5.63	2.53	88.0	25.12	131.0	23.6	35.0	2.19	3.92
0.73	4.89	3.20	113.0	32.10	89.0	18.6	48.0	1.72	3.11
0.86	2.88	1.58	54.0	17.30	33.0	18.1	49.0	1.68	5.59
0.78	3.63	2.89	95.4	25.82	70.0	17.2	57.0	1.59	4.76
1.05	2.36	4.51	144.6	43.09	72.0	13.7	76.0	1.19	3.59
0.93	5.25	2.70	98.9	29.06	78.8	22.9	31.9	1.96	2.88
0.81	8.70	2.78	65.2	21.12	110.0	15.7	62.0	1.45	3.04

1.17	4.57	3.03	107.0	30.20	99.0	21.6	40.0	2.00	4.95
1.29	2.70	2.71	106.0	29.70	74.0	15.3	63.0	1.42	4.06
0.63	2.68	2.91	97.0	27.40	47.4	23.6	30.4	2.02	2.55
0.61	5.59	3.09	102.0	28.50	49.4	14.5	69.0	1.34	5.83
1.27	6.00	2.65	102.4	27.40	55.0	22.4	33.1	1.92	4.54
1.56	4.30	2.22	64.2	19.72	144.0	16.4	57.0	1.52	3.66
1.70	2.94	2.93	106.2	28.90	146.0	23.5	30.5	1.88	2.54
0.58	7.14	3.40	84.9	26.77	54.2	17.3	49.6	1.49	3.64
1.45	13.16	2.81	74.2	21.72	91.4	20.9	42.0	1.94	6.93
1.15	14.50	4.30	128.0	35.80	195.0	28.7	22.4	2.45	3.95
0.60	3.30	3.01	94.6	27.80	94.0	13.5	76.0	1.25	4.01
0.68	6.16	4.37	134.0	38.50	35.4	15.2	63.0	1.41	3.96
0.47	7.98	2.06	65.0	19.30	34.0	17.5	54.0	1.51	4.79
0.64	3.25	2.31	75.2	21.12	65.4	27.3	23.1	2.33	3.68
0.31	7.13	2.14	58.0	19.10	51.0	16.5	58.0	1.53	5.58
1.70	3.88	3.44	126.0	32.20	55.0	24.6	28.1	1.96	3.68
0.71	7.45	2.43	84.0	24.92	68.0	13.1	76.9	1.13	3.33
1.10	1.46	1.72	42.0	13.70	60.0	14.2	71.0	1.31	3.58
1.29	4.81	3.31	115.0	33.50	75.0	13.1	76.9	1.13	2.55
0.93	12.49	2.96	111.8	32.09	140.7	22.2	33.1	1.78	2.62
1.98	1.28	3.30	113.0	32.60	107.0	17.2	57.0	1.59	3.36
0.46	6.78	3.19	115.0	33.70	149.0	13.2	85.0	1.22	3.84
0.77	11.68	1.64	58.2	17.32	71.0	19.0	42.1	1.63	4.15
0.22	30.18	2.95	95.7	29.36	30.7	23.0	31.0	1.74	3.98
0.98	2.65	2.81	98.0	28.70	46.0	22.6	36.0	1.98	3.66
0.34	4.03	3.11	107.3	30.22	37.7	14.4	71.0	1.09	3.62
0.40	12.00	1.23	44.2	13.52	62.0	22.3	40.0	1.58	3.84
1.18	2.60	3.10	102.0	28.20	49.4	15.6	58.4	1.34	5.07
0.38	13.87	2.65	96.0	29.10	20.4	32.1	21.0	2.74	3.98
0.80	11.00	2.27	77.2	23.32	47.2	21.0	36.3	1.68	3.62
1.24	1.79	2.69	96.0	27.90	81.0	22.2	37.0	1.94	3.74
1.04	2.90	3.39	104.0	31.30	38.4	13.3	70.0	1.23	4.77
0.32	7.00	1.75	65.2	17.82	24.4	24.1	35.0	2.23	3.75
3.43	0.78	4.70	148.7	41.92	122.3	13.6	73.3	1.17	3.66
0.55	14.75	4.38	151.0	43.40	92.0	15.0	69.0	1.39	4.44
0.39	11.28	1.93	48.1	15.70	94.3	16.7	52.5	1.43	3.60
1.03	1.86	2.33	92.4	22.82	88.0	18.9	47.0	1.74	3.54
1.47	9.03	4.74	134.0	38.70	148.0	35.0	22.0	3.24	4.06
0.96	5.58	1.92	76.7	21.90	22.8	39.7	20.0	3.37	4.43
0.80	2.25	1.79	63.0	18.00	31.0	21.2	35.6	1.70	4.15
0.62	3.53	4.78	169.0	46.80	43.4	16.9	59.0	1.56	3.65
1.39	1.65	5.09	129.0	39.30	85.0	15.3	6.2	1.32	3.64
1.05	1.94	4.89	87.0	31.30	132.4	15.9	64.0	1.47	3.47
1.10	1.82	1.90	56.0	18.30	71.0	17.2	49.5	1.39	3.65
2.34	3.51	2.39	87.4	23.62	30.4	16.1	55.6	1.38	3.78

0.54	7.67	3.36	107.0	30.80	47.4	15.6	51.0	1.28	5.27
0.92	2.01	3.53	129.0	37.00	72.0	15.5	62.0	1.44	4.99
0.56	2.95	2.36	64.2	20.22	43.4	15.6	61.0	1.44	4.75
0.77	6.48	4.02	143.4	38.90	52.0	14.3	72.0	1.09	3.32
0.63	5.71	2.40	83.0	24.22	114.0	13.1	76.9	1.13	4.77
0.79	2.24	3.08	107.0	30.74	36.6	13.2	75.2	1.08	4.00
1.69	0.62	1.66	59.2	17.52	83.4	15.4	61.0	1.43	4.00
1.77	1.05	3.29	119.0	34.10	44.4	14.3	69.0	1.32	4.83
1.16	2.81	3.35	81.0	27.10	59.4	16.0	61.0	1.38	3.76
1.16	3.78	4.40	141.0	39.60	137.0	29.0	31.0	2.69	3.92
0.58	8.47	2.63	76.2	23.42	27.4	19.7	44.0	1.82	4.29
0.95	3.38	2.13	63.3	18.28	75.1	14.0	74.0	1.21	4.01
0.42	16.50	2.65	96.0	26.30	59.0	18.5	44.7	1.59	4.70
0.46	3.17	3.27	78.2	26.90	27.4	17.5	48.7	1.50	3.62
1.53	0.73	3.48	126.0	35.30	17.4	15.4	61.0	1.43	3.05
0.48	4.94	3.42	91.0	28.00	72.4	17.3	53.0	1.60	3.48
1.49	3.60	3.17	102.0	30.30	84.0	16.4	57.0	1.52	2.84
0.87	6.31	3.25	85.0	25.72	86.0	13.3	77.0	1.23	3.25
0.27	5.37	2.16	52.9	16.54	32.5	15.7	61.0	1.45	3.72
0.80	5.63	2.47	94.0	26.40	47.0	28.8	22.3	2.28	3.47
1.06	4.15	3.86	127.0	35.00	67.0	13.6	75.0	1.26	3.63
1.40	1.36	2.87	101.0	27.80	49.0	26.6	24.8	2.11	3.36
1.08	10.94	2.75	83.0	23.42	292.0	16.1	55.6	1.38	6.47
0.79	2.11	3.32	127.8	37.11	37.5	15.7	63.0	1.37	3.70
0.65	19.66	2.02	45.2	15.92	129.0	18.3	51.0	1.58	4.78
1.89	2.06	3.26	89.0	26.20	61.0	15.2	67.0	1.41	3.92
0.72	2.93	3.10	110.0	31.30	52.0	13.6	72.5	1.17	3.67
0.50	4.72	3.92	101.0	33.10	95.4	14.4	57.0	1.20	3.65
2.52	2.02	4.08	132.0	36.80	112.0	32.8	25.0	2.79	3.51
0.56	4.68	2.92	104.0	28.90	20.4	15.2	60.8	1.31	3.80
0.88	2.18	2.60	72.0	21.50	160.0	15.7	63.0	1.36	5.18
0.60	4.17	3.22	113.0	32.00	16.2	16.1	55.0	1.25	3.31
0.70	1.57	2.74	80.0	23.90	132.0	18.4	47.0	1.58	5.13
0.80	3.63	2.02	57.0	16.60	76.0	13.6	71.7	1.11	3.86
0.30	28.67	3.10	122.0	32.20	147.0	19.4	41.1	1.56	4.56
0.61	2.18	1.59	55.2	16.92	36.4	20.7	36.5	1.77	2.18
0.41	5.00	3.35	112.0	33.90	58.4	15.8	57.2	1.36	3.93
0.50	8.56	1.93	50.2	16.22	60.0	15.8	57.2	1.36	3.88
0.31	5.65	1.82	73.6	21.40	37.5	18.6	39.0	1.44	3.60
1.90	1.16	2.57	79.0	22.50	39.0	13.4	74.0	1.04	2.89
0.75	2.76	3.88	140.0	39.20	90.0	13.3	80.0	1.15	2.72
0.39	2.54	3.01	89.5	26.08	36.9	14.2	70.0	1.24	3.68
0.61	1.62	2.65	56.2	19.72	47.4	20.7	42.0	1.92	3.40
1.32	5.41	2.11	66.7	19.84	87.7	13.5	76.0	1.25	4.26
0.70	2.63	1.97	55.2	17.52	69.0	17.5	47.0	1.60	4.08

0.78	7.82	2.55	98.4	24.92	58.0	17.5	57.0	1.62	5.98
1.01	5.44	2.57	90.0	26.50	216.0	14.5	74.0	1.34	7.69
1.75	3.47	1.71	45.2	14.26	84.2	17.0	51.0	1.46	5.99
1.90	1.05	3.33	97.0	27.20	72.0	17.6	48.0	1.36	4.35
1.24	2.31	2.22	92.0	26.80	59.0	17.4	48.0	1.60	3.94
0.67	1.94	1.83	47.2	16.82	88.0	14.7	64.1	1.26	4.55
0.59	4.85	2.95	113.4	30.20	46.4	14.6	69.0	1.35	3.63
0.46	2.57	3.51	96.0	29.00	35.4	15.7	61.0	1.45	3.77
0.76	17.11	3.72	70.2	24.72	128.4	15.2	63.0	1.41	4.85
1.30	2.62	2.88	106.0	30.30	46.0	15.4	64.0	1.34	3.83
1.29	0.99	3.45	110.0	31.90	106.0	10.4	117.0	0.96	4.73
0.64	2.36	3.32	86.5	25.11	81.4	17.7	54.0	1.29	3.78
0.48	11.31	1.94	63.2	18.92	187.0	15.5	62.0	1.44	3.91
0.41	4.98	1.85	56.2	15.82	33.4	16.4	60.0	1.52	3.87
1.52	3.09	3.49	118.0	33.30	147.0	15.4	63.0	1.43	4.44
0.59	3.02	2.45	51.2	18.02	40.4	19.1	50.0	1.77	3.76
1.51	1.63	2.47	91.8	25.97	147.0	15.8	62.0	1.29	3.89
0.39	2.77	3.16	102.0	29.90	34.4	39.2	22.0	3.63	4.07
1.74	7.76	2.59	106.4	28.60	58.0	19.4	41.4	1.66	5.01
0.80	3.85	2.93	95.0	28.50	109.0	13.8	76.0	1.20	4.47
0.25	5.84	2.87	73.2	24.42	72.0	17.5	55.0	1.62	3.93
0.39	5.87	1.73	45.2	13.82	80.0	15.0	69.0	1.39	3.78
0.58	3.47	2.74	95.9	26.84	75.4	37.1	21.0	3.44	3.78
0.57	3.49	3.68	133.0	38.10	48.4	18.0	55.0	1.67	3.60
0.66	5.21	3.72	128.0	37.20	45.4	15.8	60.0	1.46	3.92
3.97	3.29	3.14	105.0	30.50	111.0	16.4	61.0	1.52	4.12
0.36	6.67	3.04	93.3	28.95	71.0	11.5	88.0	1.06	4.02
1.22	1.18	1.45	49.0	14.80	28.0	15.2	66.0	1.33	3.99
0.59	13.95	3.38	110.0	31.50	92.0	16.8	57.0	1.47	4.89
1.12	4.95	3.91	119.0	34.00	103.4	16.7	60.0	1.55	3.62
1.31	5.88	4.08	148.4	40.30	99.0	15.5	59.0	1.33	6.35
0.80	8.38	3.02	87.0	26.10	67.0	14.1	77.0	1.31	4.02
0.83	2.27	3.04	93.0	28.40	61.4	17.2	50.1	1.48	4.89
0.45	1.84	3.34	87.0	26.30	26.4	17.6	54.0	1.52	5.75
0.71	2.58	2.96	113.0	32.00	49.0	16.7	58.0	1.44	3.87
1.12	5.65	2.70	105.0	29.60	34.4	27.1	33.0	2.51	2.32
0.22	3.50	3.82	94.0	29.60	43.4	27.3	30.0	2.53	4.20
0.82	7.39	3.32	110.0	31.60	46.4	16.8	54.0	1.47	4.21
0.99	8.34	2.12	77.2	22.22	56.0	14.4	66.7	1.24	4.78
1.21	2.06	1.86	59.2	17.72	77.0	13.8	75.0	1.20	3.96
0.60	7.62	3.46	124.0	34.90	42.4	16.1	60.0	1.40	4.16
0.53	11.75	4.31	126.9	37.34	54.4	13.1	82.0	1.14	3.50
0.81	1.73	3.41	112.0	33.20	46.4	18.8	50.0	1.62	5.08
1.40	3.79	3.70	110.0	31.70	89.0	16.4	54.0	1.41	3.72
2.29	1.29	2.21	69.4	21.15	144.9	13.5	76.0	1.18	3.78

1.02	4.33	3.11	94.0	28.40	50.4	18.4	47.0	1.58	3.19
1.32	5.30	2.25	65.0	20.20	69.0	18.2	52.0	1.57	5.05
1.43	5.79	4.60	145.0	41.20	194.0	29.3	21.7	2.50	3.68
0.95	6.92	2.48	93.4	25.22	76.0	17.6	56.0	1.63	6.03
0.51	11.90	2.09	82.6	23.68	50.9	22.5	32.8	1.93	4.54
0.86	1.86	2.15	79.8	24.06	27.7	31.7	26.0	2.90	3.96
0.60	6.05	2.95	107.0	30.80	32.4	18.6	43.6	1.60	3.48
1.33	4.33	3.78	110.9	32.64	76.2	14.1	75.0	1.31	3.90
1.18	2.44	1.38	50.4	14.62	72.2	24.7	32.0	2.16	2.88
1.81	2.44	3.29	115.8	32.94	81.6	16.0	62.0	1.19	3.92
1.13	2.91	2.46	80.1	23.92	92.1	12.6	88.0	1.17	4.41
0.72	5.69	2.55	89.0	25.52	83.0	16.1	64.0	1.49	3.14
1.92	4.22	2.76	77.2	25.52	124.0	15.7	61.0	1.45	4.32
0.84	2.43	2.03	81.2	23.82	52.0	21.3	37.6	1.83	4.64
0.32	5.34	2.26	65.2	19.42	50.0	14.8	67.0	1.37	3.57
0.80	1.69	3.48	122.4	33.20	30.4	21.0	45.0	1.94	5.10
0.81	1.63	2.67	94.0	27.90	68.0	15.2	68.0	1.41	4.52
0.34	24.65	4.57	159.0	45.60	63.0	25.3	36.0	2.34	5.29
1.40	4.71	1.98	83.2	23.62	123.0	19.6	33.3	1.58	4.28
1.25	3.82	2.82	73.0	24.10	118.0	14.8	66.0	1.37	3.38
1.02	3.63	3.31	116.4	30.80	95.0	13.0	87.0	1.20	4.08
1.22	1.35	4.32	150.0	43.10	45.4	17.1	54.0	1.58	4.86
0.50	7.40	2.73	74.2	25.12	156.0	12.9	77.3	1.11	4.24
0.33	5.70	4.62	137.0	37.70	39.4	13.0	84.0	1.20	4.07
1.07	2.54	2.40	86.0	26.60	71.0	16.3	54.5	1.40	3.97
1.18	2.96	2.21	85.0	23.42	78.0	20.1	47.0	1.86	4.98
1.90	0.72	2.95	88.0	26.10	175.0	26.7	30.0	2.47	4.17
1.70	1.24	4.07	128.0	36.30	51.0	15.1	60.6	1.23	3.49
0.48	4.35	3.23	99.0	29.10	71.0	15.7	61.0	1.45	4.04
1.16	1.97	3.37	97.0	29.20	95.4	16.2	54.9	1.39	4.46
1.05	3.86	4.46	144.0	41.10	200.0	16.2	58.0	1.50	4.26
1.20	15.08	1.98	62.2	19.52	59.0	17.5	41.2	1.41	5.29
0.61	2.39	3.59	90.0	27.47	46.7	16.4	54.6	1.41	3.35
0.25	21.96	2.49	69.2	21.92	189.0	12.5	86.0	1.16	3.88
0.33	2.85	2.26	73.6	21.43	15.3	16.5	61.0	1.28	3.86
1.06	3.01	3.10	103.0	29.10	71.0	14.1	68.5	1.21	3.73
1.51	1.40	3.53	124.5	36.35	68.9	15.9	58.0	1.39	3.76
1.40	6.04	3.43	115.0	34.40	129.0	11.6	99.0	1.07	3.43
0.50	4.32	2.91	109.0	32.34	46.9	16.8	51.3	1.36	4.39
0.45	2.22	2.64	93.0	29.00	19.4	17.3	49.2	1.49	4.11
0.60	4.67	3.81	107.2	35.20	47.2	13.6	77.0	1.04	2.82
1.56	7.74	3.10	101.0	30.30	199.0	16.3	58.0	1.51	3.77
0.62	2.31	2.94	58.0	20.70	81.0	14.3	72.0	1.25	3.42
0.73	3.85	3.57	120.0	34.60	68.0	17.2	58.0	1.59	4.56
0.53	5.38	2.22	72.0	21.70	97.7	15.5	64.0	1.35	3.18

0.58	2.28	4.00	139.0	39.70	26.4	25.1	26.5	2.15	2.52
1.85	2.54	3.86	123.0	35.20	77.0	25.8	30.0	2.26	4.01
1.16	2.01	3.73	138.0	38.20	89.0	16.0	59.0	1.48	4.05
1.76	1.73	2.83	68.2	21.02	380.0	12.0	92.0	1.11	6.76
2.26	0.99	5.39	171.0	51.29	122.0	15.2	60.8	1.31	4.49
0.65	2.08	2.66	84.0	23.22	28.4	13.2	79.0	1.22	4.79
0.20	33.55	2.31	84.0	24.82	25.4	21.2	36.0	1.82	4.74
0.89	1.46	3.07	108.9	31.96	26.1	15.9	56.0	1.29	3.44
1.40	1.36	2.76	103.0	26.00	22.0	16.9	58.0	1.31	3.04
0.64	18.55	2.42	80.0	23.92	79.0	20.3	38.6	1.74	5.93
0.76	0.99	2.35	94.0	26.16	48.9	20.3	41.0	1.78	3.57
1.23	2.97	3.03	108.2	31.40	103.0	17.5	48.2	1.50	3.74
1.06	2.79	2.64	94.0	25.82	34.4	13.6	81.0	1.26	5.97
1.81	2.98	3.62	72.2	23.32	39.4	16.9	60.0	1.56	3.76
1.98	1.41	2.99	110.0	31.40	76.0	13.3	75.1	1.15	3.99
1.10	1.82	2.79	120.0	34.60	80.0	21.8	34.3	1.87	3.99
2.28	2.54	5.13	150.0	42.60	110.4	24.3	35.0	2.13	3.52
2.09	0.97	3.27	109.0	32.60	253.0	14.3	67.0	1.23	3.66
0.82	2.91	2.85	111.0	32.70	50.0	18.0	46.6	1.55	4.19
0.67	2.21	3.63	83.0	27.90	60.4	24.0	35.0	2.22	2.81
1.05	4.44	2.99	75.2	22.82	258.0	12.4	89.0	1.15	3.93
0.80	2.88	2.49	92.2	26.20	18.2	20.2	38.6	1.62	4.32
2.28	3.25	3.28	113.0	33.10	114.0	18.7	43.9	1.60	4.47
2.24	0.54	4.30	148.6	43.85	78.9	16.0	58.0	1.40	3.63
0.73	3.01	4.83	129.0	38.40	205.0	12.1	91.0	1.12	4.81
0.26	4.88	1.86	45.2	15.22	76.4	17.5	52.0	1.62	3.80
1.19	10.89	5.14	165.4	43.70	146.0	26.2	25.8	2.24	4.87
0.55	4.22	2.59	65.3	20.40	75.8	15.9	56.0	1.23	4.45
0.98	6.21	4.45	154.0	44.00	111.0	13.3	75.1	1.15	3.93
1.98	1.45	2.95	107.0	30.40	48.0	17.7	53.0	1.55	3.53
1.09	3.06	2.89	102.7	29.93	41.7	22.9	35.0	2.01	2.83
1.60	1.65	3.00	102.0	29.80	126.0	13.0	81.0	1.13	3.63
0.29	6.28	2.68	101.0	28.90	49.4	13.9	70.0	1.20	3.60
0.83	4.99	2.98	99.0	27.10	155.0	16.0	62.0	1.48	4.70
0.92	1.51	3.80	129.0	37.00	33.0	12.8	78.0	1.19	3.62
0.65	14.69	3.42	99.0	29.40	30.4	19.0	42.8	1.63	3.94
0.89	2.71	3.29	112.0	32.10	62.0	15.7	61.0	1.45	3.77
1.50	2.29	5.63	185.1	52.51	116.0	14.4	69.0	1.33	4.03
2.42	2.75	3.28	106.0	30.30	153.0	15.9	60.0	1.47	4.20
0.57	4.30	2.50	66.2	21.32	123.0	14.8	67.0	1.37	4.31
1.63	1.32	3.12	97.0	29.10	36.0	20.8	43.0	1.82	3.77
0.49	4.59	3.36	110.0	32.10	49.0	17.8	51.0	1.64	5.20
0.39	4.87	2.91	69.2	23.82	81.4	16.3	58.0	1.51	3.28
1.71	3.36	3.65	107.0	32.80	112.0	14.8	65.0	1.29	4.29
1.30	4.04	2.55	62.2	20.52	110.0	16.0	58.0	1.48	3.30

0.79	1.49	3.49	122.0	34.90	39.0	25.2	31.0	2.21	3.06
1.15	1.37	2.59	86.0	24.82	45.4	20.8	42.0	1.93	3.09
0.81	3.96	2.45	73.2	22.32	59.0	20.7	37.4	1.77	3.35
1.08	4.90	1.61	49.2	15.02	62.0	14.4	68.0	1.25	4.44
1.02	7.87	2.94	103.0	28.70	42.4	37.1	21.0	3.44	3.81
0.73	2.19	3.68	123.0	35.50	39.4	17.0	50.7	1.46	4.20
0.50	6.22	3.34	85.0	26.60	105.0	14.6	64.8	1.26	4.95
0.34	3.18	3.39	100.4	29.46	43.3	16.0	56.0	1.24	3.69
0.60	3.00	3.33	108.2	31.90	109.0	17.5	48.2	1.41	3.93
1.07	4.96	4.43	147.0	42.60	42.4	15.0	62.1	1.29	3.66
1.33	1.77	3.20	104.5	29.99	83.1	12.4	89.0	1.06	3.66
1.57	2.06	4.38	155.4	42.20	133.0	13.1	80.0	1.21	3.49
0.94	6.23	2.16	42.2	14.52	185.0	16.7	52.5	1.43	4.15
0.76	4.18	3.02	112.0	32.20	48.4	17.5	48.7	1.50	3.78
2.70	3.52	1.20	103.0	28.30	133.0	15.0	61.2	1.22	4.33
1.04	3.24	3.30	121.4	32.10	112.0	12.6	87.0	1.17	4.91
1.00	4.40	2.53	106.2	31.00	89.0	15.9	57.0	1.23	2.33
2.24	2.74	2.17	73.2	21.02	144.0	13.1	86.0	1.21	3.97
0.67	2.54	5.18	156.0	43.00	28.4	15.4	63.0	1.43	3.80
0.66	1.55	3.69	97.0	30.30	30.4	16.5	53.5	1.42	3.50
0.62	4.69	3.31	61.2	22.22	137.4	16.0	64.0	1.48	3.74
0.50	5.40	2.53	91.2	25.82	38.2	17.6	47.8	1.42	5.18
0.97	8.75	2.62	88.0	25.12	58.0	29.6	30.0	2.74	4.33
0.76	2.96	2.19	65.2	18.92	82.0	17.1	59.0	1.58	3.79
0.23	6.78	2.90	75.2	24.32	31.4	15.3	63.0	1.42	4.45
1.00	13.22	2.91	98.0	29.00	59.0	20.1	39.2	1.72	6.05
0.56	3.70	3.21	101.0	29.20	54.0	13.2	76.0	1.14	3.31
1.74	1.81	3.52	129.0	39.17	61.7	18.9	47.0	1.75	4.13
2.31	5.61	2.46	83.6	24.09	159.2	18.2	45.8	1.56	4.86
0.50	12.70	2.79	100.0	29.30	40.4	21.7	34.7	1.86	4.49
0.79	5.63	2.11	65.2	19.62	68.0	15.9	65.0	1.47	3.62
0.56	4.50	2.39	86.0	24.62	53.4	18.0	46.0	1.55	3.84
0.81	7.67	3.42	119.0	35.30	59.0	15.8	57.2	1.36	4.27
2.17	3.59	4.12	138.6	41.20	61.0	38.1	14.3	3.25	4.21
2.80	1.69	2.70	111.4	30.10	80.0	19.3	41.8	1.66	3.79
0.60	5.50	2.39	73.0	21.50	82.0	13.6	77.0	1.18	3.13
0.62	4.53	3.29	86.7	26.93	39.7	16.2	58.0	1.50	4.43
0.68	6.19	3.61	124.0	35.60	52.0	15.0	62.1	1.29	4.06
0.60	5.50	2.85	104.2	30.40	50.0	16.2	62.0	1.26	3.94
0.67	6.93	0.99	30.2	9.02	65.0	17.6	54.0	1.54	4.15
0.28	31.93	4.11	95.0	30.70	42.0	15.2	63.0	1.33	3.97
0.59	7.44	2.59	98.0	27.50	28.4	20.8	45.0	1.93	4.03
0.69	4.20	3.09	108.0	31.30	52.0	16.4	54.0	1.41	4.00
0.84	8.44	2.13	58.2	18.32	108.0	15.4	68.0	1.43	3.88
0.83	6.66	2.24	72.2	22.02	60.0	27.5	29.0	2.55	2.83

1.23	1.76	3.09	117.0	32.40	83.0	16.0	56.1	1.38	4.89
0.37	46.86	1.92	65.2	22.22	51.0	26.9	27.0	2.30	3.87
1.80	4.11	2.08	79.0	21.00	79.0	20.2	38.4	1.62	6.48
0.84	6.71	2.78	110.4	30.00	42.4	23.3	39.0	2.16	4.40
0.53	3.09	4.36	138.0	42.10	46.4	16.5	53.5	1.42	4.50
0.68	4.50	3.23	105.0	30.60	72.0	13.0	77.8	1.12	4.24
1.33	3.30	3.30	110.0	32.00	95.0	15.6	58.4	1.34	3.73
0.90	9.56	2.27	77.0	20.10	62.0	20.9	36.6	1.68	5.63
1.02	3.80	3.66	128.0	36.90	43.4	15.4	59.7	1.32	4.12
1.05	5.76	3.04	102.0	29.90	208.0	11.7	102.0	1.08	2.88
0.92	5.68	1.87	68.0	19.90	93.0	16.5	58.0	1.53	4.91
0.76	4.45	2.34	72.2	22.92	90.0	14.1	74.0	1.31	4.97
2.65	1.74	4.75	161.4	43.30	182.0	13.5	78.0	1.25	2.65
1.38	3.53	4.74	152.0	44.30	120.0	12.2	95.0	1.13	4.40
0.76	3.26	3.62	115.0	34.10	77.0	16.0	56.0	1.38	3.34
1.29	3.49	2.36	74.2	22.42	74.0	19.7	49.0	1.82	4.64
0.69	4.26	2.69	107.3	31.73	43.2	18.2	47.0	1.41	4.09
0.84	9.56	4.15	134.4	35.80	170.4	25.6	35.0	2.37	3.16
0.64	2.88	3.22	114.0	33.00	47.4	19.8	44.0	1.83	3.76
2.46	6.13	2.71	65.2	21.42	125.0	14.6	64.8	1.26	5.73
0.53	21.26	2.92	86.0	25.72	37.4	15.3	68.0	1.42	2.73
1.18	1.63	3.44	123.5	34.40	91.6	19.4	40.7	1.66	4.16
0.99	3.96	3.22	112.0	32.60	62.0	20.6	37.7	1.77	3.56
1.07	3.78	2.28	64.2	19.22	79.0	18.2	54.0	1.69	3.88
1.47	6.89	3.47	119.0	33.40	76.0	22.3	42.0	2.06	4.95
0.94	8.02	2.20	61.0	19.20	91.0	14.7	69.0	1.28	3.89
0.58	5.47	2.82	63.8	20.47	54.1	13.4	73.0	1.24	3.36
0.88	6.70	2.67	79.2	24.42	62.0	15.0	62.1	1.29	3.28
2.30	1.22	3.92	136.0	40.40	107.0	16.0	56.1	1.38	3.13
0.71	3.14	3.05	110.0	32.00	57.4	25.1	34.0	2.20	3.96
1.19	2.66	4.50	148.0	42.20	100.0	13.1	86.0	1.21	3.34
1.41	2.40	2.10	84.0	23.22	106.0	20.2	46.0	1.87	2.75
0.90	4.44	3.18	79.2	25.42	84.0	15.1	61.5	1.30	4.09
1.50	4.67	2.33	60.0	17.70	86.0	18.4	44.5	1.48	4.99
0.88	3.91	3.44	100.6	29.73	39.8	16.6	58.0	1.45	5.60
0.34	9.38	3.80	126.0	37.70	97.0	11.6	99.0	1.01	5.92
0.23	31.96	3.59	116.0	34.40	24.0	16.8	56.0	1.56	3.54
0.96	4.91	2.71	96.0	26.80	41.4	16.2	54.9	1.39	3.84
2.06	0.91	3.25	98.6	29.58	142.3	13.4	79.0	1.17	2.99
0.80	5.83	2.25	63.2	19.42	140.0	14.2	76.0	1.31	3.97
1.29	4.88	3.26	98.0	28.90	254.0	14.1	68.5	1.21	3.55
1.30	2.23	4.52	134.0	40.80	98.0	21.0	45.0	1.94	3.55
1.62	1.93	4.56	134.1	40.60	113.5	19.5	47.0	1.70	3.26
0.70	4.86	3.52	127.0	36.20	144.0	15.2	60.2	1.23	4.03
0.20	8.80	2.57	83.8	24.83	17.7	19.6	45.0	1.81	3.60

1.56	3.20	3.67	116.0	32.90	241.0	12.4	82.0	1.15	3.74
1.33	1.63	4.10	133.7	40.03	54.0	14.9	63.0	1.38	3.34
1.03	3.83	3.75	86.0	28.80	139.4	13.4	83.0	1.24	4.13
0.97	4.18	3.35	109.0	31.10	77.0	17.1	56.0	1.25	4.62
1.21	3.60	4.51	145.4	37.70	209.0	32.7	26.0	3.03	3.45
0.56	3.77	4.00	126.0	39.00	62.0	14.0	78.0	1.30	4.51
0.27	21.78	2.32	80.1	23.06	35.6	15.8	63.0	1.18	4.00
0.75	4.93	2.25	77.2	23.02	27.4	18.7	43.9	1.60	5.10
0.79	6.53	2.47	108.2	32.45	49.8	20.3	38.6	1.74	4.48
0.56	9.39	2.83	94.0	29.40	47.2	20.6	44.0	1.80	3.73
0.47	5.23	3.36	107.0	32.50	36.4	17.6	47.8	1.51	3.85
0.93	1.61	3.92	134.0	38.00	35.0	17.1	54.0	1.58	3.56
0.60	15.48	3.07	97.0	28.30	75.0	15.4	59.7	1.32	3.96
0.87	3.61	2.76	98.0	28.20	48.2	19.4	43.0	1.66	3.38
0.20	18.95	4.03	154.0	43.20	13.4	15.5	64.0	1.35	3.27
0.71	2.25	3.86	135.0	39.10	53.0	14.9	62.8	1.28	3.75
0.79	9.11	2.68	88.0	24.92	186.0	16.2	61.0	1.50	4.69
0.23	5.65	1.27	32.6	10.24	30.8	15.8	63.0	1.18	4.26
0.79	10.49	2.86	110.4	29.90	21.4	33.9	25.0	3.14	4.13
0.73	4.08	3.33	112.0	31.10	63.0	14.5	74.0	1.34	4.37
0.60	2.17	2.96	105.0	29.20	23.0	17.8	47.0	1.31	4.31
1.10	6.40	3.18	107.0	29.70	165.0	17.3	58.0	1.60	3.44
1.04	11.31	3.32	109.0	33.00	187.0	17.3	49.6	1.49	4.12
1.69	2.98	3.21	125.2	36.80	116.3	13.6	71.2	1.11	3.47
1.42	19.15	3.33	111.4	29.90	216.0	18.1	53.0	1.68	4.11
0.33	5.76	1.16	27.2	9.82	42.4	16.3	59.0	1.42	4.28
0.47	13.64	3.37	109.0	30.40	45.4	20.3	47.0	1.88	3.39
0.51	24.53	4.17	129.0	37.80	45.4	47.9	17.0	4.44	3.87
0.44	7.02	2.96	90.0	27.10	66.0	13.2	76.0	1.14	5.02
2.01	1.22	4.23	105.0	32.10	248.0	11.4	106.0	1.06	4.51
0.36	6.58	3.20	79.3	25.61	40.9	13.6	72.5	1.17	4.02
0.99	7.58	1.85	62.2	17.52	77.0	19.9	45.0	1.85	4.28
0.84	4.67	3.65	129.4	34.00	59.0	23.0	39.0	2.13	3.81
0.41	6.17	3.05	90.0	27.00	26.4	18.7	51.0	1.73	3.96
1.03	2.48	3.86	138.0	39.10	46.4	16.2	63.0	1.50	3.21
0.67	15.90	3.55	131.8	39.69	53.6	21.1	37.0	1.93	3.75
1.53	3.58	2.07	68.2	19.32	98.0	16.4	60.0	1.52	4.54
1.43	1.77	3.73	135.0	39.30	85.0	14.2	68.0	1.31	2.89
1.58	6.72	3.17	110.0	32.00	71.0	19.0	42.1	1.63	4.34
0.87	4.71	3.37	111.0	31.30	53.0	15.5	59.0	1.33	3.72
0.51	4.65	3.18	119.4	32.40	44.4	13.3	80.0	1.23	4.17
1.03	2.09	4.28	108.0	34.30	36.4	16.9	51.2	1.45	3.47
0.49	2.67	3.38	76.2	23.82	77.4	15.3	60.2	1.32	3.79
1.20	4.69	4.34	152.0	42.10	103.0	14.7	64.1	1.26	4.70
0.92	11.52	3.46	71.2	23.42	229.4	14.0	74.0	1.30	4.63

0.31	6.71	3.23	84.0	24.82	33.4	11.7	97.0	1.08	4.34
0.60	15.62	2.72	91.0	25.82	137.0	16.1	55.6	1.38	3.54
1.35	1.62	4.68	160.4	43.70	71.0	14.6	64.8	1.26	3.65
1.88	0.99	3.78	124.0	37.70	65.0	14.3	72.0	1.32	3.50
0.38	2.11	3.04	105.6	29.66	38.8	37.7	22.0	2.36	2.87
1.09	11.05	3.13	117.4	30.90	168.0	13.2	81.0	1.22	5.29
1.41	8.72	3.48	121.0	37.20	186.0	21.3	37.0	1.95	4.15
0.45	10.27	1.35	53.2	15.92	61.0	32.0	18.9	2.73	4.27
1.63	1.31	4.14	132.7	39.52	107.2	16.9	50.0	1.55	3.35
0.60	9.00	2.11	71.2	20.82	107.0	15.8	56.6	1.28	4.25
1.63	1.17	3.23	117.2	35.40	86.9	13.6	68.0	1.25	3.63
0.80	5.88	2.35	68.2	21.82	123.0	13.1	75.5	1.07	3.62
1.96	2.87	2.20	50.2	16.02	76.4	21.6	42.0	2.00	4.45
0.83	1.45	2.49	91.9	26.78	21.2	17.4	49.2	1.49	3.91
0.27	9.93	3.79	89.0	29.30	76.4	15.9	56.7	1.37	3.55
0.57	4.58	2.52	81.0	23.22	63.0	15.3	66.0	1.42	4.83
0.27	7.78	2.96	98.0	28.60	59.0	14.0	74.0	1.07	4.13
0.37	5.19	2.16	78.2	21.82	29.4	14.1	68.5	1.21	3.20
0.46	2.37	3.42	112.0	34.90	29.4	16.4	54.0	1.41	4.50
1.88	5.80	2.27	82.0	23.42	100.4	15.7	62.0	1.45	5.58
0.31	11.84	2.16	80.0	23.32	58.0	16.5	53.5	1.42	4.33
0.69	6.54	4.18	124.0	36.60	170.0	13.6	71.3	1.17	5.71
0.70	8.57	1.80	37.2	13.04	185.4	18.2	45.4	1.47	3.87
1.11	3.77	2.08	64.2	19.42	192.0	17.1	50.6	1.47	4.63
1.20	3.14	2.16	82.0	22.70	16.0	20.6	44.0	1.91	4.24
0.88	3.24	2.93	98.0	28.00	77.0	14.5	65.5	1.25	3.75
0.30	14.43	2.79	97.0	27.40	20.2	18.9	47.0	1.74	4.40
0.39	2.82	4.43	107.0	32.90	17.4	17.9	47.0	1.54	4.25
0.98	4.06	4.17	98.0	31.31	195.1	12.1	88.0	0.94	4.08
0.77	3.99	4.58	134.0	39.10	26.4	14.6	70.0	1.35	3.63
0.86	6.67	3.04	93.0	27.20	126.0	14.1	68.5	1.21	4.56
1.04	7.59	3.57	125.7	37.66	93.5	12.4	78.0	1.14	4.18
1.00	5.20	3.52	121.0	34.00	49.2	17.5	49.0	1.36	4.32
0.50	4.40	3.29	66.0	22.10	53.0	16.1	54.9	1.31	3.86
1.62	0.85	3.00	105.4	30.97	42.1	13.9	75.0	1.06	4.41
0.30	4.67	3.42	111.0	32.70	41.2	13.2	76.0	1.02	4.36
0.97	11.55	4.46	122.0	36.10	53.4	18.7	47.0	1.73	4.30
0.99	2.69	3.71	84.0	27.00	37.4	16.5	53.5	1.42	2.81
1.21	4.40	1.15	44.7	12.47	79.7	19.4	40.9	1.56	3.87
2.17	6.92	1.53	57.2	16.42	59.0	50.3	15.0	4.66	4.13
1.75	2.22	2.65	90.0	26.50	199.0	14.3	69.0	1.32	3.30
0.26	4.77	3.12	113.0	31.10	34.4	16.5	53.5	1.42	3.71
0.39	11.59	4.51	150.0	41.60	95.0	24.9	28.0	2.13	3.24
4.19	2.94	1.54	38.2	12.62	229.0	14.9	67.0	1.38	3.66
1.14	1.67	3.44	99.0	30.70	85.0	11.6	90.0	1.07	4.07

2.87	3.93	1.94	60.2	17.42	130.0	19.8	40.1	1.70	5.20
0.40	2.75	3.01	99.0	28.40	25.0	24.4	34.0	2.26	3.59
0.30	7.00	3.10	100.2	29.70	34.2	19.4	40.9	1.56	4.47
1.20	1.08	3.38	119.0	33.30	71.0	19.0	42.3	1.53	3.80
0.98	2.92	2.63	102.0	28.20	78.0	16.2	55.0	1.39	3.80
0.69	3.83	3.24	78.2	27.00	71.0	13.2	75.0	1.22	3.81
1.40	1.50	4.19	146.0	41.60	25.2	13.6	71.2	1.11	3.86
1.21	6.80	4.68	145.0	39.90	323.4	18.4	51.0	1.33	4.25
0.21	10.10	2.62	71.2	21.74	31.4	14.6	65.0	1.35	3.87
0.81	2.05	3.19	107.0	32.50	56.0	17.4	52.0	1.61	3.20
1.32	3.20	3.32	126.1	36.59	101.7	16.5	53.5	1.42	3.50
1.00	1.30	3.55	71.0	21.60	92.0	18.0	39.1	1.45	4.32
1.52	1.84	3.01	96.0	28.80	74.0	14.5	66.0	1.34	3.66
0.90	4.89	4.08	142.0	41.90	77.0	18.8	38.0	1.46	2.73
0.55	6.53	2.49	99.4	26.30	46.4	17.9	47.0	1.54	5.82
0.53	22.17	3.23	112.0	32.10	103.0	19.7	40.5	1.69	4.76
1.30	2.36	3.71	133.0	38.00	45.4	16.1	55.6	1.38	4.41
1.24	1.24	3.74	129.0	37.30	12.4	16.1	54.8	1.31	3.61
2.38	1.91	4.26	141.3	41.94	147.4	22.0	35.0	2.01	4.21
1.21	4.35	1.96	47.0	15.08	71.3	13.6	72.5	1.17	3.85
1.48	3.95	1.77	56.2	16.92	146.0	20.3	38.6	1.74	5.06
1.00	2.90	3.34	118.0	33.00	90.0	18.2	45.4	1.47	3.89
0.60	13.63	3.91	125.0	34.40	52.4	15.0	62.1	1.29	4.05
0.55	8.96	2.17	68.2	20.72	92.0	22.1	39.0	2.05	4.12
0.32	5.69	3.52	84.0	27.20	47.4	16.0	56.1	1.38	3.63
1.15	2.82	3.69	126.0	36.80	82.0	16.5	53.2	1.42	3.51
0.50	3.80	3.03	97.0	27.50	69.0	13.2	75.0	1.22	3.88
0.29	3.03	2.43	81.0	23.12	24.4	16.7	52.5	1.43	5.04
0.54	8.28	2.80	89.0	26.70	49.4	13.6	72.5	1.17	4.82
1.82	1.70	2.68	105.0	30.40	54.0	13.0	81.0	1.12	3.07
1.92	0.99	4.11	148.0	36.00	123.0	13.9	70.0	1.20	4.19
1.48	2.45	2.15	69.2	20.02	89.0	16.3	54.5	1.40	4.54
0.98	8.27	3.19	97.0	29.80	32.4	18.5	44.7	1.59	3.02
0.36	7.94	2.70	89.0	25.22	47.4	16.1	55.6	1.38	3.22
0.21	22.52	2.91	85.0	24.72	44.4	15.0	62.1	1.29	4.18
0.92	11.51	2.61	85.0	24.42	341.0	19.4	45.0	1.80	3.94
1.52	7.03	2.44	60.2	19.22	124.0	14.6	64.8	1.26	5.03
1.38	6.37	3.63	111.0	31.80	87.4	21.9	34.2	1.88	3.23
1.36	5.58	2.50	54.2	18.22	88.0	16.4	54.0	1.41	4.85
1.32	2.73	1.86	63.2	17.42	98.0	15.1	62.0	1.40	3.69
1.53	8.33	1.58	50.2	16.22	187.0	20.4	38.3	1.75	5.82
0.17	27.00	2.20	55.2	17.22	60.0	24.5	34.0	2.05	3.76
1.12	2.89	3.43	125.0	34.40	48.4	13.9	70.0	1.20	5.07
1.56	1.15	4.19	138.0	39.30	69.0	16.3	54.3	1.40	3.91
0.47	4.57	3.21	101.5	29.90	28.6	18.4	47.0	1.58	3.68

1.81	10.43	4.42	121.0	40.90	119.0	16.0	56.1	1.38	3.97
0.51	6.47	1.69	74.2	21.02	37.4	21.4	35.5	1.83	3.11
0.35	8.74	4.28	132.0	36.50	32.4	14.4	66.2	1.24	4.99
1.23	1.21	3.76	129.0	38.50	59.0	12.6	76.0	1.16	4.01
0.42	11.88	2.14	70.2	20.12	49.4	28.5	30.0	2.41	5.01
1.05	6.70	1.41	48.2	14.22	86.0	21.3	43.0	1.49	3.98
2.31	1.52	4.00	139.4	38.00	161.0	17.5	55.0	1.28	4.96
2.83	3.76	2.15	71.2	20.72	157.0	14.9	62.8	1.28	4.28
0.50	4.00	3.19	93.2	28.50	25.4	18.2	46.0	1.41	3.76
0.88	2.85	3.71	80.0	26.20	113.4	17.3	49.6	1.49	4.48
0.78	1.14	2.49	81.0	24.52	46.4	14.7	64.1	1.26	4.18
0.59	2.54	3.74	99.0	30.70	30.4	19.0	42.8	1.63	3.46
1.39	1.81	4.07	139.2	41.54	87.4	14.1	64.0	1.30	3.24
1.26	2.58	4.47	153.0	43.50	121.0	15.5	59.0	1.33	4.77
0.94	2.77	3.14	107.0	29.40	40.4	17.9	47.0	1.54	5.15
1.63	3.31	3.48	121.0	36.00	71.0	25.3	27.3	2.16	4.32
1.53	4.24	3.17	100.0	29.00	77.0	16.5	53.5	1.42	3.55
1.18	9.84	1.73	60.2	18.42	86.4	19.8	40.0	1.81	4.46
1.00	1.80	3.19	110.0	29.90	84.0	25.0	30.0	1.94	4.57
0.29	6.31	2.19	78.2	22.92	21.4	23.5	30.7	2.01	3.85
1.71	2.69	2.70	93.1	27.98	76.7	12.7	75.0	1.17	3.87
1.80	4.33	3.45	119.0	32.50	76.0	16.2	54.3	1.31	3.60
1.19	1.51	3.34	86.4	27.49	75.2	11.6	86.0	1.07	3.97
1.35	3.40	2.56	61.2	19.42	210.0	13.0	77.8	1.12	4.35
1.23	4.59	4.44	133.0	38.90	121.0	10.9	96.0	1.01	3.20
0.48	6.29	2.44	79.2	22.72	48.4	13.4	69.0	1.24	3.47
0.87	11.20	4.20	145.0	40.60	96.0	16.7	52.5	1.43	3.83
0.94	4.60	2.85	89.0	26.00	91.0	16.2	54.9	1.39	6.10
0.81	1.22	2.75	95.4	26.92	22.9	14.0	74.0	1.07	2.94
1.18	1.59	4.03	128.1	39.12	178.7	12.0	82.0	1.11	3.62
1.30	4.75	3.48	130.0	37.50	39.4	18.3	45.4	1.57	3.93
1.35	17.53	2.33	77.2	22.82	95.0	17.0	51.0	1.46	4.80
0.84	6.55	3.86	120.0	33.30	76.0	23.8	30.0	2.04	3.81
0.63	2.44	3.65	118.0	35.30	66.0	18.5	44.7	1.59	4.50
1.10	5.09	3.36	108.0	29.10	107.0	15.2	60.0	1.23	5.10
2.93	1.80	2.48	84.0	24.02	83.0	20.7	36.5	1.77	5.10
0.96	2.09	3.90	132.4	35.60	28.4	20.0	39.5	1.72	3.14
0.54	4.46	2.59	83.0	23.92	111.0	15.9	56.7	1.37	2.89
1.85	1.87	3.55	134.0	38.30	219.0	12.1	87.1	1.04	3.58
0.30	3.80	2.60	77.2	23.02	30.4	14.6	64.8	1.26	3.56
2.32	1.44	2.43	87.7	27.66	230.9	15.0	62.1	1.29	3.80
0.36	8.33	2.35	65.2	20.82	98.0	48.7	14.0	4.35	4.32
0.71	2.10	4.05	126.0	36.30	53.0	14.1	68.5	1.21	3.47
1.04	2.10	4.26	127.0	37.00	127.0	13.6	71.3	1.17	3.48
1.78	1.10	3.37	127.7	35.57	76.0	14.6	67.0	1.25	3.58

1.27	1.13	3.56	125.8	35.98	51.4	15.6	57.0	1.21	3.64
0.76	6.25	4.14	133.0	37.90	72.4	29.2	21.8	2.49	4.01
0.19	19.00	1.68	52.2	15.72	42.4	14.8	63.5	1.27	3.46
0.60	9.17	3.12	118.0	33.40	114.0	18.9	42.8	1.52	2.92
0.94	6.74	4.03	124.4	36.60	142.0	24.4	36.0	1.67	4.19
0.91	2.47	2.81	99.0	29.40	34.4	20.7	37.4	1.77	2.72
0.84	3.33	2.62	81.2	25.82	103.0	14.5	64.5	1.18	3.80
0.80	3.63	2.94	70.0	21.10	86.0	15.3	59.6	1.24	4.37
1.00	5.90	2.45	65.2	20.42	249.0	14.0	68.1	1.14	4.26
0.54	14.83	2.86	89.0	26.30	46.4	19.6	40.8	1.68	3.76
0.51	5.51	3.20	91.0	29.10	32.4	12.9	73.0	1.19	4.22
0.55	8.73	3.31	101.5	30.16	87.8	14.0	68.5	1.14	4.52
0.62	13.31	4.93	155.0	44.60	207.0	40.4	13.1	3.44	4.88
1.10	1.25	4.13	126.3	38.22	93.3	13.7	67.0	1.26	4.46
1.14	3.31	3.27	121.0	34.60	45.4	15.7	57.8	1.35	4.00
0.94	2.32	4.13	141.0	39.10	48.4	17.3	49.6	1.49	3.91
1.30	3.36	4.02	139.0	40.50	48.4	19.4	41.4	1.66	2.29
2.28	0.77	3.81	125.0	35.20	126.4	15.0	62.1	1.29	3.50
0.94	14.43	3.73	121.0	36.10	94.0	12.7	80.7	1.09	5.73
0.36	9.06	3.72	120.0	34.20	54.0	15.5	56.0	1.42	4.39
1.16	7.16	3.39	114.0	33.20	63.0	13.8	66.0	1.27	4.48
2.21	2.14	4.04	137.0	37.80	60.4	32.3	22.0	2.93	3.93
0.34	5.59	2.85	70.2	23.62	47.4	16.0	56.1	1.38	4.05
0.38	10.92	2.18	75.2	21.62	55.0	15.3	60.2	1.32	4.46
0.60	0.88	3.53	93.0	28.28	27.4	18.4	45.0	1.58	4.21
0.65	3.06	2.91	98.8	29.19	41.8	16.3	52.0	1.50	4.29
1.34	6.70	3.17	111.0	33.00	264.0	11.0	85.0	0.97	3.98
1.26	3.70	3.73	123.0	34.30	108.0	25.8	26.5	2.21	4.07
0.49	2.45	4.12	102.0	32.50	62.4	18.6	45.7	1.60	3.17
1.35	1.44	3.38	113.4	30.60	80.0	17.6	49.4	1.51	3.77
2.24	2.28	3.27	118.0	34.10	122.0	16.4	53.3	1.33	3.49
0.83	2.10	3.98	126.0	36.20	44.4	15.9	56.7	1.37	3.78
0.62	11.68	3.44	84.0	25.72	220.0	13.6	77.0	1.17	5.59
0.90	5.44	4.40	143.0	41.20	398.0	15.0	61.2	1.22	2.87
0.65	2.29	2.50	52.2	17.72	106.0	13.2	76.0	1.14	3.71
1.30	2.31	3.25	104.2	29.30	47.2	13.6	71.2	1.11	4.06
1.42	1.19	2.85	92.0	26.40	111.0	19.9	39.8	1.71	4.11
0.70	8.51	2.10	68.2	21.22	24.4	17.0	51.0	1.46	3.99
0.78	4.13	2.57	77.2	23.54	111.2	14.1	68.5	1.21	4.40
0.88	2.58	1.27	42.2	12.42	44.4	22.1	41.0	1.88	4.31
0.89	4.66	3.90	127.0	35.50	55.0	14.0	69.3	1.21	4.02
0.36	2.44	3.09	101.3	30.57	19.6	19.4	46.0	1.64	3.50
0.28	13.39	3.96	125.0	36.00	111.0	19.4	41.4	1.66	4.42
1.20	3.02	1.65	58.2	16.82	29.4	22.9	39.0	1.95	3.55
2.59	1.05	3.75	126.4	37.81	106.9	13.2	81.0	1.02	3.75

0.68	17.51	3.26	87.0	25.22	103.0	15.4	59.6	1.32	5.63
0.89	9.58	3.15	108.8	32.33	82.2	15.0	61.0	1.16	3.24
1.29	3.99	2.01	51.5	16.27	101.9	24.0	37.0	1.64	5.62
0.77	6.18	2.99	102.0	30.50	69.0	15.1	61.5	1.30	4.88
1.02	3.88	3.32	132.7	38.00	59.5	13.0	82.0	1.01	3.63
0.10	41.10	1.25	31.0	9.99	63.5	23.0	31.7	1.97	4.76
0.79	3.62	4.80	149.0	41.90	96.0	14.2	67.7	1.22	3.60
1.12	1.58	3.95	130.0	37.50	49.4	15.8	50.0	1.29	3.82
0.49	5.65	2.42	44.1	14.74	46.2	17.2	56.0	1.26	4.91
1.63	1.96	3.81	119.0	34.90	99.0	16.4	54.0	1.41	3.73
4.90	0.76	3.08	100.0	29.80	225.0	14.0	68.5	1.14	3.98
2.16	3.86	1.82	58.2	17.52	136.0	15.8	57.2	1.36	4.09
2.10	2.05	2.58	83.2	24.42	73.0	17.3	49.1	1.40	3.59
1.78	0.56	4.21	110.0	35.00	359.0	15.9	56.6	1.37	3.63
1.46	7.10	3.64	121.0	33.20	173.0	35.6	15.9	3.04	5.44
0.62	3.81	4.27	150.0	44.00	41.4	17.6	48.3	1.51	3.57
0.60	3.50	3.40	111.0	30.00	51.0	15.5	58.3	1.26	4.60
0.85	1.24	2.93	98.0	28.80	50.0	17.2	50.1	1.48	3.61
0.98	2.67	4.58	142.0	41.40	69.0	14.6	64.8	1.26	4.52
1.20	2.17	2.87	92.2	27.70	102.0	14.0	79.0	1.07	3.89
0.48	5.52	5.01	113.0	36.30	51.4	16.4	53.8	1.41	4.62
1.27	2.64	4.34	133.0	37.80	109.0	21.0	35.7	1.80	3.75
0.96	2.15	2.26	83.1	24.93	95.5	14.4	65.5	1.17	4.60
4.30	2.33	1.79	52.2	16.02	221.0	20.4	38.0	1.64	5.50
0.75	6.92	4.03	135.0	38.40	49.4	18.4	47.0	1.58	4.32
0.90	5.00	3.24	98.0	26.30	78.0	19.8	32.6	1.60	2.54
0.90	3.09	4.13	141.0	40.20	61.0	16.0	56.1	1.38	2.56
2.66	1.16	3.92	129.0	40.19	167.5	11.0	105.0	0.95	5.07
0.94	6.01	1.24	34.2	11.12	106.0	22.5	32.8	1.93	4.66
1.80	5.78	2.26	85.2	23.92	137.0	27.8	17.2	2.26	2.78
3.50	0.43	2.94	108.2	30.60	75.0	15.6	57.6	1.27	3.40
1.57	2.88	2.55	84.0	24.82	95.0	16.4	54.0	1.41	3.91
0.60	2.85	1.05	29.2	8.42	61.0	15.4	59.6	1.32	4.00
1.25	1.66	3.58	121.0	34.80	107.0	13.6	73.3	1.17	3.58
1.95	1.69	2.52	79.3	24.42	54.8	22.6	38.0	1.97	4.01
0.84	4.49	3.07	78.2	24.82	44.2	14.5	65.1	1.25	3.99
1.78	4.20	2.58	71.2	22.12	110.0	15.2	60.8	1.31	3.82
1.66	5.90	4.32	135.0	37.20	182.0	31.3	19.5	2.67	3.99
0.62	3.05	2.64	98.4	26.80	73.0	13.1	76.9	1.13	4.10
1.64	5.73	1.86	59.2	16.52	75.0	24.2	28.2	2.07	5.20
0.88	3.03	2.80	81.0	24.32	58.0	14.4	66.7	1.24	3.93
1.45	2.54	1.06	34.2	9.62	115.0	16.9	51.5	1.45	4.22
1.01	7.35	1.99	53.9	16.52	65.5	17.8	47.5	1.53	5.60
0.87	4.86	2.94	94.2	26.88	75.2	14.8	63.5	1.27	4.29
0.70	13.43	3.37	116.0	33.10	225.0	18.0	47.8	1.55	4.37

0.46	2.43	3.08	76.2	25.02	24.4	14.4	66.2	1.24	3.47
1.25	2.64	4.79	161.0	46.00	119.0	16.5	54.1	1.42	4.41
0.73	2.66	3.58	124.6	35.73	53.8	15.8	63.0	1.18	4.28
1.42	4.04	3.13	102.0	30.90	185.0	14.6	64.8	1.26	5.12
1.17	7.83	2.96	107.4	29.10	85.0	13.0	82.0	1.01	4.67
0.66	2.95	2.09	68.3	19.96	19.0	19.0	49.0	1.38	3.90
0.80	6.13	4.44	112.0	35.50	52.0	15.0	61.4	1.22	4.58
1.38	2.68	3.84	130.0	37.30	22.4	15.1	61.5	1.30	4.18
2.87	1.72	2.74	96.8	29.19	47.0	19.5	48.0	1.39	3.03
2.12	0.87	3.45	121.8	35.56	74.1	13.5	78.0	1.04	3.58
2.24	1.68	3.77	128.0	35.60	99.0	16.0	56.1	1.38	3.01
0.81	7.19	3.07	66.4	21.29	114.1	17.0	57.0	1.25	5.86
0.50	6.00	3.60	107.2	30.60	69.0	14.3	65.9	1.16	4.03
0.73	17.40	4.41	142.0	40.70	205.0	33.7	16.3	2.87	4.31
1.60	3.99	4.31	121.0	37.10	75.0	13.3	76.1	1.15	3.52
1.94	1.02	3.22	123.0	35.20	137.0	11.2	94.0	0.97	3.81
0.95	3.92	3.26	115.4	30.70	24.4	16.1	55.4	1.38	2.06
0.61	7.11	2.02	71.6	21.38	136.8	17.5	48.3	1.50	5.87
1.21	4.29	3.22	96.0	29.40	37.4	16.6	52.7	1.43	4.75
0.66	1.23	3.97	97.0	30.40	59.4	16.0	56.0	1.38	4.01
0.64	5.52	4.22	98.0	31.00	113.4	14.2	68.2	1.22	4.42
0.80	9.03	4.00	135.0	39.10	59.0	15.7	57.8	1.35	3.66
1.56	2.81	3.49	141.0	10.02	103.9	17.1	115.0	0.85	3.52
0.77	6.17	3.23	108.0	31.30	39.2	17.7	49.0	1.52	5.90
0.90	4.56	3.04	106.2	29.70	66.0	19.3	41.4	1.55	6.26
0.50	1.66	2.23	69.2	20.28	66.9	23.0	31.6	1.84	4.36
1.40	0.71	3.38	112.0	32.40	161.0	12.6	94.0	0.98	4.01
0.83	4.94	4.93	159.0	44.30	112.0	24.0	28.6	2.05	3.96
0.50	3.40	4.29	137.0	39.30	45.2	18.4	44.6	1.48	3.46
1.68	3.79	1.92	53.2	18.22	76.0	19.6	40.0	1.68	4.58
2.10	1.67	3.62	142.0	38.00	63.0	16.1	56.1	1.38	5.24
0.15	11.00	3.10	103.9	30.95	31.1	15.1	67.0	1.13	3.32
0.56	3.91	2.28	81.4	22.64	9.4	20.4	40.0	1.75	3.72
0.92	3.98	3.85	125.0	37.40	59.4	14.5	71.0	1.10	4.18
4.05	6.81	3.09	93.0	28.20	71.4	36.8	17.3	3.14	4.94
1.00	9.10	1.82	59.0	16.30	46.0	24.3	29.0	1.94	5.06
0.20	15.00	3.02	111.0	34.30	16.2	17.9	46.5	1.44	4.71
1.20	4.92	1.56	50.2	15.82	95.6	19.7	47.0	1.43	4.64
0.55	2.40	4.44	108.0	34.60	60.0	15.5	59.0	1.33	2.87
0.65	6.14	3.13	115.7	32.79	152.4	17.0	50.4	1.38	3.16
0.60	7.67	3.16	106.2	32.30	112.0	17.5	48.2	1.41	4.69
1.45	3.50	3.97	119.0	35.40	198.0	16.5	57.0	1.53	4.91
1.40	10.93	2.78	74.2	24.32	211.0	16.2	54.3	1.31	4.15
0.80	5.13	2.22	65.2	19.32	67.0	15.7	57.0	1.27	3.65
0.40	5.25	3.04	103.2	30.10	29.2	19.1	49.0	1.39	3.91

2.10	3.29	2.71	59.2	20.12	304.0	15.6	57.6	1.27	3.52
2.50	3.04	1.98	66.0	17.40	85.0	16.1	54.8	1.31	4.18
1.30	2.00	4.69	147.0	44.60	70.0	19.3	41.4	1.55	4.52
1.20	5.42	2.29	71.0	18.70	77.0	19.9	39.5	1.60	5.65
1.40	6.43	2.33	76.0	21.20	46.0	21.4	35.3	1.72	4.67
1.10	1.55	4.07	133.0	38.20	28.2	17.5	48.2	1.41	4.40
0.98	2.06	2.70	91.6	26.89	69.5	17.7	47.4	1.43	3.59
0.70	7.71	3.28	125.0	33.40	40.0	16.9	50.9	1.37	3.49
1.00	3.10	2.90	82.2	24.82	44.0	15.0	61.4	1.22	4.14
0.70	6.29	3.11	98.2	29.10	31.2	17.1	50.0	1.38	4.34
2.50	1.44	3.09	108.2	30.90	65.0	17.9	46.5	1.44	3.68
1.70	0.94	4.85	140.0	38.70	128.0	13.2	75.2	1.08	3.80
1.50	2.87	2.60	72.0	21.10	49.0	20.6	37.2	1.65	3.87
1.40	0.86	3.12	97.0	26.40	154.0	17.3	49.0	1.34	3.76
0.44	15.75	3.22	110.0	31.40	43.4	16.0	56.1	1.38	2.57
0.79	11.32	2.74	89.0	28.30	102.0	29.8	21.1	2.55	5.06
1.07	14.31	3.37	109.4	28.40	74.0	21.7	34.7	1.86	4.86
2.33	3.34	2.13	59.4	18.49	164.5	12.7	80.7	1.09	4.54
1.32	7.84	2.65	90.0	26.60	26.4	21.4	35.5	1.83	4.47
2.20	12.57	2.32	82.0	25.52	40.4	30.3	28.0	2.81	3.49
0.78	15.47	2.55	88.0	26.90	30.4	37.3	21.0	3.45	3.80
1.30	12.92	4.70	147.0	38.70	93.0	23.3	30.9	1.86	4.94
1.48	4.88	2.81	87.0	25.82	101.0	35.6	18.1	3.04	3.42
0.90	4.18	1.36	54.2	15.72	43.4	25.8	26.5	2.21	5.06
0.63	6.56	2.52	85.0	25.02	34.4	21.9	34.2	1.88	3.19
0.80	3.34	2.51	59.3	18.54	119.7	21.4	37.4	1.83	4.66
1.24	5.44	3.93	124.0	34.40	51.0	71.0	5.4	6.01	5.04
0.83	11.37	3.08	91.4	26.30	85.4	40.1	19.0	3.71	4.66
0.63	26.40	3.08	85.0	25.92	76.4	18.7	48.0	1.73	4.72
0.60	10.83	2.06	76.2	22.62	33.2	29.8	21.1	2.36	3.91
0.76	9.33	4.87	149.0	41.80	129.4	40.0	20.0	3.66	5.08
0.69	5.29	2.11	75.2	21.82	52.0	32.0	27.0	2.96	3.59
1.00	12.40	5.21	158.0	47.00	109.0	20.4	38.0	1.64	5.53
0.70	4.14	1.48	52.0	13.20	44.0	44.3	11.4	3.45	6.68
0.66	9.05	0.87	33.2	10.82	86.0	15.5	59.2	1.33	3.94
2.68	2.96	1.37	50.2	15.22	86.0	29.0	20.9	2.48	6.04
0.90	7.14	2.74	97.0	27.70	61.0	21.4	34.6	1.83	7.01
0.35	57.66	2.68	91.4	24.82	253.0	21.6	35.0	1.85	3.40
1.68	2.93	3.41	127.0	35.20	85.4	17.6	48.3	1.51	5.53
1.61	6.43	4.32	133.0	38.80	189.0	45.0	17.0	3.97	5.02
1.10	12.00	2.70	84.0	22.40	19.0	28.3	16.6	2.30	5.31
0.58	19.62	2.21	87.0	24.52	96.0	26.8	27.1	2.29	3.97
2.17	8.62	1.91	63.2	17.62	62.0	39.5	12.5	3.36	5.18
0.15	15.40	1.37	58.2	17.52	56.0	17.2	50.1	1.48	2.13
0.87	17.84	2.38	83.0	23.12	28.4	30.9	19.9	2.64	3.72

0.53	7.15	2.55	93.0	26.50	49.4	23.1	31.5	1.98	4.73
0.28	9.61	2.10	73.2	21.72	58.0	31.7	27.0	2.94	4.42
0.95	5.09	2.37	79.0	22.90	82.0	22.1	40.0	1.93	4.09
1.60	13.31	3.01	106.0	27.90	156.0	19.0	42.3	1.53	3.69
2.40	4.29	5.28	149.0	42.50	126.0	20.4	38.0	1.64	4.04
1.53	2.37	5.16	166.3	48.62	90.8	30.0	28.0	1.96	3.53
1.08	6.97	1.60	60.1	16.76	167.6	65.2	12.0	3.66	4.53
1.91	13.37	3.74	127.0	36.40	342.0	16.9	55.0	1.56	3.45
1.12	7.65	1.90	77.4	20.92	129.0	28.8	31.0	2.67	3.20
1.59	14.92	1.88	64.2	18.62	54.4	22.4	39.0	1.70	6.53
1.45	8.08	3.78	120.4	31.30	93.4	37.0	21.0	3.43	4.14
0.76	19.33	2.31	85.4	21.62	55.0	20.0	39.5	1.72	3.29
0.52	9.40	3.79	131.0	36.00	101.4	15.6	51.0	1.28	6.44
1.25	2.29	3.95	132.3	40.22	73.1	26.7	25.1	2.28	4.15
0.90	6.89	4.66	104.2	34.50	285.0	48.8	5.9	4.01	4.24
0.18	32.78	2.14	81.6	24.28	37.6	24.9	27.6	1.98	5.88
2.00	1.85	4.96	132.0	41.00	156.0	18.8	43.5	1.61	3.35
1.94	1.63	3.78	145.0	41.10	153.0	12.6	81.7	1.09	4.15
0.15	37.07	2.34	95.7	26.59	24.2	16.2	61.0	1.20	4.34
0.83	3.71	1.80	75.0	20.67	54.8	23.9	36.0	1.80	4.09
0.40	5.00	2.78	110.0	28.60	35.0	29.5	21.5	2.33	8.23
1.25	3.86	4.41	137.6	39.49	109.1	24.1	37.0	1.65	2.90
2.63	1.95	2.46	89.0	24.82	90.0	19.1	42.5	1.64	4.15
1.20	4.42	2.05	83.2	23.42	54.0	28.3	22.5	2.24	3.98
1.52	4.49	1.48	37.2	12.22	116.0	25.2	33.0	2.11	4.56
1.10	5.45	1.87	64.4	17.32	179.4	14.8	63.5	1.27	6.63
0.20	8.00	0.95	40.2	11.32	26.2	30.2	20.7	2.39	3.88
1.73	8.66	2.52	70.2	23.72	151.0	17.3	49.6	1.49	3.57
0.34	18.76	2.39	74.7	22.85	56.1	18.0	50.0	1.67	5.36
1.11	4.23	5.13	167.0	46.30	225.0	21.4	35.5	1.83	2.64
1.58	4.38	1.68	59.9	17.69	38.1	27.9	31.0	2.36	5.90
0.70	5.14	3.63	129.0	33.80	19.0	23.2	31.2	1.85	4.15
1.29	6.26	3.07	131.4	34.30	194.0	27.0	33.0	2.50	2.73
1.06	4.36	3.57	126.6	35.49	145.4	19.9	39.5	1.60	3.27
0.95	6.89	2.70	98.0	28.10	49.4	19.4	41.4	1.66	6.02
0.83	6.51	4.32	135.0	39.50	144.0	28.1	23.1	2.40	3.81
1.23	9.01	2.35	87.3	26.69	31.8	25.5	33.0	1.98	3.74
0.67	8.01	2.65	93.0	26.50	61.0	20.2	38.9	1.73	3.88
1.82	4.63	5.05	152.4	46.61	164.2	23.5	33.0	2.14	3.57
1.99	9.13	1.72	48.2	15.32	45.4	18.5	44.7	1.59	4.26
0.58	1.97	3.91	106.0	31.60	62.0	12.7	80.0	1.18	2.75
0.58	1.90	4.18	133.0	38.91	43.8	13.4	79.0	1.03	3.64

Na	Cl	BUN	Cr	GLU	NH4	fushui	MELD	group
138.7	111.1	10.87	102.2	8.790	70.00	2	10.7	0
136.3	105.4	11.31	68.3	8.870	19.00	2	17.34	0
133.4	98.2	8.43	96.0	5.840	24.00	2	13.02	1
143.5	116.0	14.41	78.3	10.480	29.00	0	4.63	1
150.3	123.4	33.70	218.3	3.590	131.00	0	26.97	0
143.5	118.0	9.84	61.0	10.910	23.00	2	9.02	1
128.4	95.8	25.49	180.8	5.440	25.00	2	29.67	0
130.2	91.1	11.59	155.8	8.260	125.00	2	25.7	1
129.3	85.2	54.10	538.2	8.190	42.00	2	38.09	0
137.8	104.0	16.61	67.1	20.450	59.00	2	14.57	0
139.7	105.4	26.70	184.8	7.070	44.00	0	10.82	0
132.3	79.4	3.61	207.4	18.300	96.00	1	21.1	0
117.4	76.2	12.12	218.4	9.420	84.30	2	42.05	0
144.9	101.6	4.94	174.5	25.350	179.00	1	41.03	0
125.6	99.1	10.00	150.4	3.390	27.00	1	32.09	1
138.4	104.8	17.33	48.9	21.240	43.00	0	8.04	0
124.4	98.9	25.40	200.1	6.040	43.00	2	33.13	1
136.5	105.7	15.63	107.9	7.020	26.00	2	20.33	1
137.5	107.5	9.74	32.4	22.270	41.00	0	9.45	0
137.4	93.6	13.33	105.7	9.410	70.00	1	35.09	1
132.9	102.2	15.13	80.0	6.770	33.00	2	27.81	0
128.7	99.7	21.66	73.4	9.400	75.00	2	28.12	1
129.8	93.4	18.71	129.2	6.420	43.00	2	24.9	1
132.7	100.8	27.82	97.7	5.990	31.00	0	22.34	0
132.0	99.6	29.65	98.7	27.050	48.00	2	20.17	1
148.7	108.4	22.99	96.8	17.690	26.00	2	17.2	1
129.1	99.5	14.49	157.4	10.280	7.00	0	15.48	1
118.8	83.2	28.09	199.4	4.390	34.00	2	24.45	1
137.1	101.3	19.03	265.2	8.130	72.00	2	22.28	1
142.0	100.2	21.34	125.9	11.260	26.00	1	31.11	0
150.5	109.6	7.68	37.6	22.230	34.00	2	23.42	1
132.5	101.7	8.40	52.0	27.100	58.00	2	24.48	0
128.0	97.8	16.74	135.8	9.250	76.00	0	29.9	0
118.0	82.8	10.08	115.0	5.840	13.00	2	22.76	1
139.5	99.0	8.17	103.0	12.520	13.00	2	19.86	1
123.0	79.1	11.64	71.8	4.852	42.00	0	18.41	1
141.3	106.7	1.66	66.3	6.170	6.00	1	33.43	1
129.8	85.0	19.07	91.3	12.520	93.00	1	24.5	0
143.6	113.6	6.88	55.0	5.300	35.00	0	11.07	0
134.9	104.4	4.61	40.3	8.530	8.70	2	17.2	1
136.2	109.0	3.75	69.3	10.070	19.00	0	21.63	1
132.7	102.1	4.37	63.2	5.240	24.50	2	30.44	0
140.9	110.1	7.28	112.4	4.160	4.00	0	18.43	0
133.3	103.1	5.69	60.0	4.040	43.00	2	13.65	1

127.2	88.4	8.25	62.0	11.860	37.00	1	26.7	0
139.1	105.6	6.71	74.2	5.900	46.00	2	12.23	1
124.7	89.2	29.84	198.9	5.940	82.00	2	25.56	0
142.4	112.7	6.15	55.0	4.080	28.00	2	13.73	0
139.3	113.0	10.11	63.0	7.570	15.00	2	15.61	1
141.8	104.6	3.18	55.7	4.640	46.00	1	11.11	0
128.1	88.8	9.79	131.1	6.410	36.00	2	35.56	1
140.5	109.8	4.91	62.0	5.200	40.00	1	8.58	0
120.6	85.4	28.25	344.4	5.790	29.00	1	34.57	0
135.8	99.5	4.08	72.1	7.770	68.00	1	23.19	0
133.3	97.9	9.47	68.6	11.570	28.00	0	13.45	1
126.5	87.6	10.11	145.6	7.710	40.00	2	27.73	0
109.6	80.7	17.15	122.6	6.710	86.00	1	17.52	0
137.7	105.2	7.31	95.5	5.490	35.00	2	15.31	1
135.0	91.3	21.22	280.0	11.480	98.00	1	35.65	1
129.9	93.8	10.25	63.1	5.690	30.00	1	33.32	1
128.4	101.2	13.79	93.0	8.910	56.00	2	16.07	0
137.7	102.7	7.42	57.0	3.300	70.00	2	32.96	0
126.3	86.8	21.70	374.7	6.070	34.00	2	35.15	0
132.3	97.4	12.66	118.3	13.910	41.00	2	17.31	1
125.2	98.4	14.67	214.0	2.200	37.00	2	33.3	0
126.0	96.0	5.84	55.8	8.580	92.00	2	19.28	1
129.4	106.7	14.71	98.3	7.800	57.00	2	12.45	0
130.4	102.3	16.19	139.4	6.650	316.00	2	26.23	1
122.9	88.2	29.74	259.2	4.300	27.00	2	34.07	1
135.8	103.3	5.51	50.5	9.960	46.00	0	14.17	0
129.7	97.0	10.09	97.7	6.930	38.00	2	24.58	0
128.2	104.7	9.41	149.3	7.140	19.00	1	11.92	1
130.6	100.8	5.91	61.1	8.590	10.00	0	24.67	0
139.1	105.8	5.27	55.6	6.670	40.00	0	15.29	0
131.5	101.5	43.72	509.0	7.290	23.00	2	41.43	0
135.1	101.0	4.64	55.6	6.210	30.00	2	27.9	0
128.2	96.0	27.74	197.0	13.490	5.00	2	27.76	0
136.0	103.1	7.47	62.4	21.330	54.00	0	13.16	0
137.2	109.3	7.29	106.0	5.550	86.00	2	11.33	0
126.4	91.2	10.15	147.8	6.040	17.00	2	23.95	1
136.8	109.1	8.05	66.0	6.850	38.00	0	19.15	0
130.2	92.6	10.95	161.3	5.880	43.00	2	22.66	1
139.0	105.5	2.77	35.6	9.000	37.00	0	29.54	1
139.8	108.2	18.64	62.9	8.070	17.00	2	17.37	1
122.1	82.4	7.83	106.4	6.100	20.00	2	26.82	0
127.1	90.2	13.15	521.4	7.620	15.00	2	26.77	0
155.0	127.4	4.31	76.0	5.270	22.00	0	12.61	0
133.7	102.9	4.07	54.0	6.650	14.00	2	23.52	0
143.9	104.2	3.75	39.0	5.670	36.00	0	2.77	0

141.3	113.4	6.62	56.3	13.670	44.00	0	12.39	0
134.7	102.1	17.69	165.4	6.630	57.00	1	15.85	1
128.2	98.2	3.58	27.2	11.630	54.00	1	26.27	0
135.0	110.4	3.71	52.9	8.830	25.00	2	13.29	0
143.8	111.3	4.12	60.0	5.290	20.00	0	7.91	0
139.7	106.5	11.21	103.0	5.550	16.00	0	16.57	0
136.1	106.2	3.18	67.9	6.570	53.00	1	6.02	1
127.3	102.3	7.17	57.2	8.890	19.00	2	6.45	0
126.1	95.4	19.71	69.6	36.940	83.00	2	9.7	1
139.6	105.5	4.93	38.0	6.390	66.00	2	15.85	0
139.2	104.9	9.82	84.8	11.790	71.00	0	9.33	0
135.7	98.7	7.38	113.1	5.620	19.00	1	26.46	1
129.7	90.6	7.51	99.6	6.510	7.00	2	15.96	0
142.0	112.9	9.86	104.5	15.110	36.00	0	9.28	1
133.4	97.1	5.73	105.0	5.940	4.00	2	17.5	0
141.2	104.8	4.67	53.3	14.260	56.00	0	17.13	0
119.5	83.4	24.91	109.3	30.240	37.00	2	31.62	0
143.6	111.8	11.97	31.4	7.840	42.00	0	13.23	1
139.9	106.4	9.01	92.0	5.530	69.00	2	23.31	0
134.3	105.8	6.75	57.8	7.020	25.00	0	14.23	0
140.0	98.9	2.16	59.0	3.940	58.00	1	28.72	0
132.9	95.3	5.20	100.3	7.650	26.00	1	38	1
136.3	101.3	4.96	82.0	6.470	13.00	2	26.66	0
135.1	107.9	8.22	84.0	4.650	21.00	2	8.52	0
139.2	104.3	5.18	89.0	4.250	16.00	1	24.21	0
140.9	110.7	5.38	57.5	9.310	21.00	2	13.53	0
139.2	108.0	5.48	63.4	6.020	37.00	2	10.23	0
134.1	96.9	5.18	70.9	8.670	68.00	2	32.74	1
125.6	82.1	28.75	252.0	2.790	20.00	2	43.11	1
132.7	91.9	13.45	158.8	19.910	36.00	2	18.06	0
143.1	110.0	20.36	112.3	6.740	79.00	2	19.17	1
137.2	105.1	11.24	64.6	7.680	53.00	0	6.6	1
130.6	92.6	11.09	173.3	1.730	58.00	2	29.63	0
129.3	86.1	7.31	58.9	7.360	27.00	2	7.3	0
145.8	114.3	2.97	58.0	6.920	22.00	1	16.46	0
133.7	105.5	13.29	50.6	9.920	53.00	2	19.67	0
133.2	99.5	3.13	28.7	7.370	27.00	0	23.02	0
150.2	116.7	18.16	78.9	22.590	63.00	2	11.98	0
140.5	107.8	6.44	76.5	4.910	46.00	0	14.1	1
135.8	105.2	9.42	79.9	7.790	29.00	0	20.5	0
131.5	103.9	12.89	61.6	16.400	23.00	0	10.07	0
137.2	106.9	3.75	56.8	7.900	22.00	0	15.17	0
136.6	101.5	6.21	64.7	6.660	43.00	2	28.37	1
140.9	112.1	6.40	89.0	6.640	14.00	2	18.22	1
129.5	97.7	6.68	93.0	5.960	27.00	0	17.27	0

135.2	103.8	9.52	68.1	6.320	36.00	2	23.59	0
139.0	106.7	4.36	70.0	3.310	22.00	2	23.94	1
142.3	108.1	6.15	53.0	4.050	9.00	1	14.41	1
123.6	96.0	8.14	64.5	5.610	21.00	2	16.3	0
129.9	100.4	34.15	162.8	6.260	40.00	2	25.77	1
135.8	109.0	5.96	58.0	7.970	33.00	2	3.19	1
135.3	96.9	3.06	68.0	6.950	24.00	2	19.63	0
132.7	100.7	6.61	89.9	11.330	36.00	2	13.99	1
137.2	93.8	14.03	159.5	10.890	44.00	2	28.37	1
132.3	94.0	6.88	66.0	7.100	11.00	2	22.84	0
137.9	102.0	4.60	65.1	6.000	17.00	0	17.52	0
132.3	101.7	7.29	83.9	6.000	67.00	0	10.78	0
128.4	97.0	6.80	37.7	20.530	20.00	2	17.39	0
136.6	108.0	7.11	56.0	13.670	48.00	0	8.56	1
131.9	102.6	10.37	88.6	8.190	22.00	2	10.7	1
138.4	108.1	2.60	56.0	10.940	29.00	0	12.21	1
131.0	99.8	8.91	89.3	16.910	52.00	2	12.02	1
143.1	105.8	14.12	93.0	12.990	243.00	1	2.63	0
133.3	98.7	6.98	72.0	5.990	19.00	1	7.95	1
142.1	110.0	3.87	58.1	6.620	36.00	1	15.82	0
144.0	109.2	5.47	35.2	10.870	30.00	0	8.38	0
140.2	108.9	6.83	58.9	11.430	34.00	2	23.37	0
143.6	107.9	13.89	685.7	5.890	32.00	1	32.31	0
142.1	106.6	21.47	65.2	16.100	57.00	2	10.79	1
132.1	103.2	3.37	49.0	19.260	63.00	0	3.43	0
128.2	102.1	6.33	46.0	6.080	47.00	0	7.59	1
142.4	113.1	5.87	47.7	13.760	47.00	0	9.08	0
139.6	111.3	4.23	55.3	11.200	55.00	0	15.85	0
125.4	90.8	5.49	102.2	5.220	25.00	2	19.12	0
136.9	109.2	16.25	108.0	4.950	12.00	2	1.94	0
127.2	87.4	5.40	55.5	11.270	44.00	2	25.07	0
139.5	105.6	4.57	65.4	6.440	29.00	2	25.67	0
127.4	99.0	25.55	206.5	8.170	24.00	2	19.33	0
140.1	106.9	3.34	51.4	6.760	16.00	0	14.37	0
129.8	93.5	3.53	49.0	5.340	24.00	2	14.52	1
131.0	95.2	4.22	58.0	4.250	62.00	2	27.44	0
138.0	108.0	7.15	59.0	5.700	29.00	0	13.04	0
141.6	110.6	3.45	57.0	4.730	56.00	0	10.25	0
126.0	93.5	5.43	51.9	5.260	23.00	0	7.34	1
135.7	99.1	3.46	66.9	7.670	28.00	1	31.35	0
136.8	107.5	6.56	79.0	13.660	40.00	1	14.43	0
142.4	99.7	7.35	59.5	5.890	51.00	1	25.81	0
128.9	95.3	8.88	167.9	7.700	24.00	1	19.37	0
121.4	86.3	14.28	252.8	7.930	59.80	1	26	1
132.7	101.2	15.19	119.0	8.890	29.00	2	13.13	0

135.4	103.6	25.11	122.0	20.770	37.00	2	24.83	0
136.4	107.7	7.42	52.0	4.940	47.00	0	10.2	0
128.4	93.3	15.78	91.1	7.890	45.90	2	20.67	0
133.0	99.5	6.84	116.0	6.880	37.00	2	15.36	1
140.5	104.5	3.39	52.6	4.340	51.00	1	25.18	0
137.4	99.8	7.23	119.7	6.650	6.00	2	18.25	1
131.4	100.8	16.94	175.0	7.510	34.00	2	36.32	0
134.8	108.9	16.31	79.5	8.130	25.00	2	17.61	0
138.1	107.2	5.39	64.0	7.000	33.00	2	10.12	1
136.1	99.6	5.14	68.1	5.290	36.00	1	24.8	1
133.8	95.9	20.84	290.0	9.290	29.70	2	45.95	1
139.8	105.1	7.17	77.0	7.510	35.00	0	8.66	0
140.6	109.6	6.53	47.0	8.150	72.00	2	11.27	0
133.7	104.7	14.30	67.7	7.790	77.80	1	11.18	1
145.4	106.0	9.74	111.0	7.150	38.70	1	19.74	1
133.5	99.5	7.54	86.0	5.920	26.00	2	10.48	0
145.3	108.7	11.73	369.3	20.300	18.00	0	16.59	0
138.0	111.6	9.39	70.6	9.780	77.00	0	9.74	0
139.7	111.2	7.36	84.0	5.560	40.00	2	16.31	0
138.5	107.0	5.18	62.5	5.990	40.50	1	14.19	0
154.3	126.4	16.17	130.5	6.810	44.00	2	12.75	0
131.5	98.6	4.52	44.3	21.450	64.20	0	7.19	1
133.4	98.9	15.32	200.0	5.270	62.00	2	33.64	1
127.1	92.2	15.37	47.0	6.120	38.20	1	13.97	1
142.9	108.3	7.72	50.0	18.650	42.00	2	20.08	0
131.5	97.6	13.52	129.8	9.980	31.70	2	13.23	1
141.5	106.5	3.05	55.0	5.530	49.90	2	11.32	1
128.9	83.4	9.88	44.3	7.570	31.00	0	24.58	0
144.3	108.1	8.49	81.0	5.680	60.00	0	8.19	0
138.2	101.4	9.06	222.0	15.730	71.00	0	34.65	0
137.8	109.3	6.87	87.0	10.370	38.00	0	12.74	0
143.5	102.6	2.54	238.9	8.670	960.00	2	21.75	0
130.7	94.7	15.88	142.9	7.190	64.50	0	28.03	0
130.9	105.6	13.76	113.0	7.020	21.00	2	12.06	0
129.7	91.8	5.00	61.8	5.300	39.00	2	20.19	0
133.7	102.2	26.48	187.0	7.530	29.00	2	17.91	1
130.8	98.1	3.44	48.7	10.500	53.00	1	31.18	0
130.9	101.8	18.78	105.0	6.750	38.00	1	24.53	1
139.7	120.3	4.72	65.5	5.310	20.20	1	13.88	1
139.2	104.2	3.75	70.5	5.810	34.70	2	27.27	0
142.6	103.3	2.94	60.0	13.660	55.00	2	18.24	0
129.2	98.1	9.42	90.0	6.010	23.00	2	13.48	0
133.3	97.1	26.56	281.2	6.930	10.00	2	34.8	1
135.6	104.7	12.32	90.0	19.540	44.00	0	18.36	1
133.4	102.0	10.83	74.6	6.100	15.89	2	18.69	1

133.7	96.5	9.48	65.0	7.690	44.00	0	27.39	1
138.3	106.6	17.47	162.7	35.410	65.00	2	18.49	0
133.7	99.9	10.58	86.0	14.290	26.00	2	20.78	1
133.6	104.0	5.88	69.8	7.670	37.60	2	9.45	0
128.1	93.8	16.67	116.0	7.920	34.00	1	17.72	0
144.8	113.1	21.65	194.0	31.320	28.00	2	20.54	0
126.9	93.4	6.42	70.7	9.160	51.00	0	32.45	0
131.1	100.4	6.69	80.0	7.550	26.00	2	20.87	1
148.2	109.3	15.14	66.4	13.140	46.30	0	16.98	0
129.1	98.1	7.15	94.6	9.240	42.90	2	14.38	1
129.6	89.4	6.34	66.0	7.360	46.00	1	17.85	0
136.1	98.4	4.50	20.6	13.850	65.40	0	13.36	0
140.2	108.3	5.19	40.0	8.220	13.80	0	11.27	1
138.4	107.4	4.04	51.7	9.020	39.40	0	11.11	0
122.5	93.0	12.57	102.0	5.330	23.00	2	20.2	0
151.7	117.9	4.22	34.0	5.980	52.00	0	12.8	0
137.1	105.8	3.47	62.0	4.320	33.00	1	33.09	1
138.8	103.8	1.78	52.0	4.740	37.00	0	34.32	1
124.3	84.7	22.40	209.8	9.490	55.50	2	18.91	0
134.0	104.7	8.16	82.8	37.000	37.00	0	13.17	0
127.5	93.6	5.39	74.9	8.980	28.90	2	13.63	0
145.2	112.1	9.88	68.1	6.690	22.90	1	12.23	0
136.7	108.7	5.01	63.7	17.240	34.70	2	13.14	0
139.0	105.3	2.94	52.0	5.890	44.00	1	34.13	0
129.3	98.0	11.52	147.7	15.620	43.00	2	19.21	1
132.0	97.6	20.56	288.4	11.050	70.00	2	27.66	1
127.7	84.8	4.25	62.3	11.670	28.00	0	8.14	0
138.1	105.4	7.14	61.8	8.380	23.00	0	11.49	0
138.5	106.9	53.65	471.9	6.170	51.00	2	24.59	1
103.7	71.3	6.31	66.5	6.380	80.00	2	18.97	1
136.7	102.7	10.32	70.3	17.250	35.00	1	11.91	0
132.6	102.4	14.23	78.4	5.130	8.00	2	11.55	0
136.4	106.6	15.82	59.7	10.120	88.00	1	9.21	1
129.2	100.4	12.37	118.0	11.490	87.00	2	20.32	1
138.6	109.6	9.27	113.0	10.480	21.00	2	11.28	0
134.7	105.5	5.92	88.9	5.230	36.00	2	24.43	0
140.9	107.3	4.35	58.7	14.600	14.00	0	12.89	0
141.6	110.6	2.89	59.0	6.020	33.00	0	14.57	1
130.9	103.6	13.64	229.4	4.820	23.00	1	21.74	0
123.8	90.3	4.33	47.1	14.650	20.00	2	28.64	0
131.1	100.8	34.23	326.0	11.820	7.00	2	28.69	1
122.0	91.3	8.46	65.3	10.390	13.00	2	24.01	0
140.1	109.6	21.74	95.7	7.650	25.00	0	5.89	0
137.3	105.1	6.48	94.8	7.630	15.00	2	26.95	0
128.1	84.4	10.94	132.9	8.740	27.00	1	32.68	0

134.6	108.5	27.22	198.1	4.890	42.00	1	27.98	1
141.2	101.2	5.74	42.0	7.650	22.00	2	13.9	0
137.9	96.1	4.42	58.0	8.090	20.00	1	20.23	1
132.1	103.4	17.85	85.0	12.450	48.00	1	6.85	1
146.5	114.8	2.88	74.6	7.000	20.00	0	23.54	0
145.9	112.3	20.85	78.1	8.140	54.00	2	11.27	0
130.5	95.1	7.88	74.0	18.930	39.00	1	3.11	1
131.0	96.8	6.30	45.6	11.360	37.00	2	22.13	1
130.3	96.6	12.71	112.0	4.880	31.00	2	27.09	1
136.5	105.9	22.03	418.0	3.660	38.00	0	34.4	0
141.6	109.4	4.60	35.1	8.510	51.00	0	19.02	1
135.9	95.2	6.36	133.9	2.880	48.00	2	24.22	1
139.0	100.3	6.23	64.6	14.570	36.00	2	23.08	1
136.5	97.6	6.51	135.5	3.900	33.00	2	32.99	1
136.1	107.0	3.43	57.5	6.490	39.00	1	21.68	1
137.2	101.2	3.68	64.1	14.620	48.00	0	32.63	1
138.7	100.8	4.18	60.5	5.360	25.00	2	29.33	1
139.2	103.3	4.30	55.1	8.540	27.00	2	30.12	1
138.5	106.6	3.49	42.0	6.840	26.00	0	21.26	0
124.4	93.0	5.44	65.3	12.420	55.00	2	37.3	0
144.0	112.4	7.33	42.6	10.320	69.00	2	9.96	0
131.5	102.9	32.96	253.5	4.440	20.00	2	40.05	0
129.1	91.4	6.34	90.5	6.010	32.00	2	23.97	1
139.4	94.1	7.85	124.0	4.640	28.00	2	23.51	1
143.4	105.4	5.02	89.5	12.100	30.00	0	18.08	1
133.7	96.2	7.25	58.0	5.380	14.00	0	24.6	1
130.9	95.6	2.29	41.5	8.830	21.00	2	14.87	0
137.2	103.1	2.51	57.9	7.120	40.00	0	16.35	0
134.5	103.4	9.73	65.4	11.710	19.00	1	12	0
136.5	103.6	8.46	74.9	22.120	39.00	1	23.06	0
133.0	97.0	6.91	48.0	6.070	39.00	2	10.92	0
135.2	90.7	4.02	61.5	7.990	42.00	2	11.9	0
140.2	107.7	5.73	56.5	7.750	41.00	0	24.45	0
142.8	102.7	3.84	35.0	8.520	39.00	0	27.56	1
139.0	106.8	5.39	70.0	4.300	47.00	2	11.1	1
118.5	77.2	41.21	884.0	7.250	44.00	1	52.32	0
137.6	109.1	15.00	71.5	13.120	58.00	0	13.75	0
126.5	96.3	7.11	59.0	41.380	52.00	1	9.36	0
135.0	101.2	8.61	61.0	9.000	77.00	1	24.36	0
137.1	98.1	7.98	58.1	13.630	45.00	2	12.72	0
137.5	99.6	5.85	56.2	5.570	34.00	2	17.07	0
137.8	104.5	6.80	42.0	7.100	11.00	2	12.63	0
136.8	105.0	4.51	40.9	21.040	66.00	0	19.12	0
137.1	101.2	10.51	153.0	24.300	33.00	1	14.56	1
142.9	114.1	11.39	43.4	6.340	36.00	0	11.43	0

146.1	110.8	5.13	58.0	5.170	21.00	0	15.92	0
133.3	93.7	3.94	58.3	11.900	32.00	2	36.37	1
137.2	101.3	5.22	71.0	7.890	22.00	1	20.07	1
133.4	105.1	7.88	55.1	6.640	21.00	2	8.76	0
136.6	107.7	14.29	139.9	25.380	21.00	2	13.13	0
133.2	94.3	13.97	333.0	7.760	41.00	2	34.05	1
140.8	105.2	6.44	33.4	7.970	49.00	0	26.15	0
147.9	122.1	21.05	98.0	9.860	92.00	2	9.72	0
140.4	100.5	9.85	142.0	5.910	48.00	1	36.27	0
128.6	96.2	10.08	61.3	6.860	53.00	0	25.49	0
140.5	110.1	14.88	45.4	20.490	22.00	2	17.77	0
142.5	103.6	17.03	81.0	5.610	25.00	2	19.97	1
140.2	106.6	13.95	124.6	8.520	15.00	0	16.36	1
131.6	83.0	2.44	42.5	4.470	49.00	2	9.64	1
134.9	105.5	5.50	59.9	15.860	48.00	2	18.31	0
127.5	83.9	8.24	75.0	6.090	29.00	2	13.29	0
139.6	99.1	2.79	64.0	4.000	49.00	1	25.59	0
128.8	91.1	11.45	100.2	13.030	31.00	0	14.67	0
141.7	110.1	7.57	54.5	7.590	27.00	0	9.87	1
121.6	91.6	7.44	83.0	7.720	32.00	2	21.83	0
137.1	105.1	5.25	53.3	5.640	33.00	0	11.4	0
135.3	97.8	5.06	43.9	15.370	61.00	0	13.1	1
130.7	94.6	20.78	147.4	9.320	36.00	1	28.68	0
130.5	94.5	13.94	53.6	9.870	26.00	2	26.56	0
142.5	108.6	1.61	62.8	5.460	27.00	0	15.94	0
141.8	109.5	8.97	74.5	9.490	13.00	1	11.19	0
137.6	99.5	5.18	84.4	9.850	15.00	2	10.37	0
125.3	92.6	3.11	15.0	10.780	15.00	2	26.28	1
137.5	96.8	4.06	201.5	7.020	38.00	2	39.78	0
135.9	98.5	7.02	107.1	3.720	36.00	0	18.63	0
134.5	103.6	4.30	67.3	10.860	34.00	2	24.38	1
125.3	81.4	25.06	201.4	5.470	32.00	0	22.56	0
134.2	98.8	8.80	89.6	5.530	39.00	2	16.71	0
132.4	97.2	13.99	148.1	6.910	31.00	2	18.33	0
133.5	93.4	8.16	142.6	2.880	32.00	2	29.12	1
134.7	102.4	3.59	59.3	17.720	45.00	0	12.58	1
144.4	114.1	22.42	209.3	7.930	57.00	2	12.29	1
136.5	99.6	13.73	87.4	8.680	19.00	2	18.88	0
133.3	100.4	11.29	89.4	8.960	39.00	0	8.36	1
141.4	107.4	11.49	76.0	8.360	39.00	0	12.21	0
125.3	92.1	29.88	154.6	28.490	38.00	2	28.34	0
138.4	109.4	9.55	56.1	11.660	40.00	2	15.05	1
122.7	83.5	19.16	133.2	12.140	40.00	0	8.98	0
136.3	100.3	8.25	45.1	12.590	17.00	0	18.35	0
137.6	103.6	6.82	53.6	6.720	41.00	2	14.51	1

120.0	89.6	11.24	71.6	7.360	16.00	1	28.22	0
145.0	111.6	22.49	90.7	11.910	30.00	2	4.73	0
139.0	98.9	3.45	66.8	9.960	28.00	2	22.56	0
144.0	109.5	4.71	63.4	5.160	27.00	0	15.56	0
133.9	95.8	7.30	70.5	7.070	30.00	2	17.06	0
136.4	96.3	14.24	120.5	4.910	26.00	1	16.98	0
138.0	104.5	5.87	53.3	5.920	39.00	0	9.57	0
118.7	82.4	3.97	60.9	4.840	13.00	0	24.44	0
135.5	102.8	15.13	123.8	6.930	40.00	2	3.26	0
130.7	97.8	4.36	78.5	5.890	15.00	0	19.11	1
154.5	118.9	41.62	238.2	11.470	54.00	0	20.44	1
118.4	87.0	11.39	80.6	5.220	37.00	1	21.95	0
128.9	90.1	4.44	55.7	8.040	63.40	2	12.97	0
129.2	102.8	35.98	301.5	14.900	47.50	1	29.31	0
151.1	123.6	15.84	84.4	8.450	20.60	2	13.47	0
145.7	112.3	6.58	30.0	5.660	39.00	0	15.63	0
135.5	99.1	18.29	133.4	7.160	19.00	2	19.56	0
129.9	100.3	13.93	136.0	5.420	37.00	2	30.02	1
119.2	84.3	9.24	94.9	7.950	27.00	2	16.62	0
116.3	88.9	30.26	159.0	8.650	30.00	2	14.4	1
135.8	104.2	8.70	61.1	18.510	31.00	0	12.6	1
141.0	114.0	12.32	67.1	5.760	33.00	0	9.96	0
130.9	101.9	8.91	105.9	6.520	7.20	2	22.76	0
139.0	110.1	5.17	66.2	6.780	39.40	0	19.89	0
132.6	94.6	3.84	55.4	7.560	45.00	0	25.33	1
148.4	99.9	14.28	69.0	4.770	29.00	2	27.62	0
126.2	100.6	36.45	163.1	8.850	26.00	2	9.53	1
131.0	103.0	21.88	724.0	5.280	12.00	2	29.55	0
143.6	108.5	2.95	63.0	5.280	12.00	1	13.92	0
136.6	106.3	18.38	104.7	4.850	23.00	2	10.55	0
132.3	95.0	4.40	64.8	4.830	24.00	0	20.35	0
126.4	95.4	36.35	201.4	7.930	97.00	2	33.84	0
127.5	97.0	15.28	116.4	5.370	34.00	2	22.92	1
136.4	105.7	3.28	147.6	13.370	28.60	0	23.85	0
132.3	102.7	6.84	52.5	8.230	26.00	1	25.51	0
143.5	106.1	5.12	65.8	8.360	115.00	2	3.44	0
138.1	110.6	5.88	60.7	12.600	50.00	0	10.3	0
129.8	91.6	28.26	329.0	4.990	28.00	2	30.3	0
136.4	102.6	8.07	78.0	6.180	18.00	2	16.36	1
128.0	92.4	10.17	209.2	5.920	38.00	2	29.06	0
129.2	97.9	4.45	59.6	13.210	23.00	2	10.11	0
144.9	110.0	22.15	148.1	5.650	18.00	2	17.76	0
126.7	90.3	22.02	199.9	9.990	12.00	2	37.55	0
130.2	100.6	6.58	79.6	7.430	70.00	1	6.53	0
138.5	105.1	2.58	46.7	17.700	32.00	0	26.79	0

139.0	103.3	3.14	56.7	5.860	28.00	0	30.42	0
137.6	100.0	5.20	72.1	6.930	48.00	2	13.18	0
137.9	99.6	9.37	83.0	12.450	12.00	2	19.06	0
125.8	96.6	7.47	67.3	5.910	26.00	1	20.66	0
137.1	99.6	6.87	55.8	15.970	19.00	2	7.21	1
141.7	107.3	3.30	65.0	6.420	33.00	0	14.88	0
140.6	106.2	2.93	56.6	8.070	35.00	0	33.07	1
138.6	105.3	8.05	47.4	19.600	32.00	1	13.83	1
142.2	106.7	9.13	58.0	5.540	77.00	2	17.41	0
130.1	98.0	6.22	76.0	5.030	18.00	2	17.33	1
140.5	96.7	5.55	82.0	6.760	39.00	2	12.4	1
141.8	103.5	4.91	60.6	4.570	33.00	0	20.39	0
144.9	111.4	8.05	62.0	5.610	12.00	1	18.6	1
137.9	96.6	3.73	68.4	8.480	48.00	0	15.34	0
139.8	102.1	11.85	82.0	4.890	10.00	1	28.52	1
139.8	104.9	4.40	50.0	4.040	39.00	1	21.6	0
139.2	111.0	6.76	30.7	7.720	50.30	1	2.41	1
140.2	107.5	5.16	77.0	4.120	29.00	2	10.09	0
139.7	109.7	18.13	122.9	11.580	54.00	0	21.65	0
138.1	100.0	2.71	49.9	7.180	26.00	2	26.99	0
137.8	107.4	9.29	136.0	5.530	26.00	2	18.15	0
140.9	103.1	4.03	48.0	9.100	42.00	1	26.86	0
137.0	103.1	10.14	234.2	4.100	104.00	2	27.01	1
128.9	92.5	5.26	55.7	10.700	35.00	0	21.67	1
129.0	97.5	18.19	175.3	9.620	59.00	1	18.77	0
127.9	88.4	2.06	36.4	7.610	55.30	2	26.98	0
138.3	110.6	18.36	67.6	21.430	42.90	0	4.92	1
137.0	102.6	5.42	71.6	9.500	57.00	0	12.78	0
125.9	96.5	10.74	53.0	8.050	20.00	1	10.24	1
122.1	89.5	25.69	184.5	20.350	20.00	2	29.49	0
128.6	96.2	11.78	42.2	6.710	48.50	2	11.88	1
141.2	107.7	6.49	71.0	11.210	24.00	0	2.5	0
134.3	101.5	2.86	59.6	9.810	41.00	1	23.26	0
138.5	102.2	4.90	60.0	8.540	67.00	1	12.33	0
124.5	93.9	33.43	318.2	9.380	45.00	2	31.39	0
116.9	86.3	22.87	152.0	7.250	15.00	2	17.4	1
136.6	103.3	10.18	152.6	11.320	20.00	0	25.96	1
134.9	104.3	6.17	35.0	32.330	76.00	0	12.72	0
140.5	101.9	2.48	54.5	3.700	36.00	0	12.98	0
128.5	90.9	4.57	74.9	7.440	18.00	2	19.41	0
141.7	112.2	8.10	89.0	5.380	21.00	1	6.55	0
136.4	105.4	11.48	409.9	7.000	34.00	1	36.52	1
131.7	102.9	9.98	120.0	5.170	70.00	2	34.49	0
120.4	85.1	13.07	95.0	10.590	22.00	2	16.03	0
126.0	96.5	6.28	53.0	12.210	31.00	2	18.41	1

133.1	92.6	2.54	46.0	6.070	54.00	2	15.26	0
131.3	92.4	6.56	68.1	5.650	52.00	0	32.21	1
137.7	102.9	7.24	75.0	9.150	25.00	2	14.69	0
135.7	106.5	12.14	76.7	8.590	13.70	0	9.67	0
133.1	97.0	11.54	156.7	7.200	35.00	2	29.07	0
135.5	94.6	3.29	63.9	1.700	61.00	0	11.29	0
140.5	103.1	4.88	94.9	10.170	51.20	2	14.01	0
134.1	98.4	13.03	137.6	5.960	15.00	2	27.6	1
131.6	93.7	8.40	82.7	12.420	66.00	1	19.05	1
131.7	106.6	3.98	37.2	5.490	41.00	2	17.54	0
128.0	85.2	23.15	246.4	17.050	25.00	0	20.42	0
136.7	107.7	5.56	53.8	6.690	27.00	0	17.1	1
122.4	90.8	8.85	87.0	7.400	18.00	2	25.86	0
137.4	107.2	9.08	81.6	5.960	40.00	2	9.65	0
133.8	95.3	7.51	136.6	8.310	20.00	2	13.62	0
137.1	107.3	6.91	53.0	7.160	45.00	2	10.8	0
125.8	96.2	12.67	150.7	5.910	45.00	0	33.21	0
134.5	107.2	4.51	44.1	4.960	24.00	1	25.06	1
142.4	108.2	11.49	72.0	9.110	24.00	2	9.77	0
130.7	98.4	13.75	124.7	8.730	49.00	1	18.17	0
135.7	101.2	5.09	81.0	5.770	35.00	2	26.88	0
141.7	115.1	15.49	105.0	15.560	6.00	2	19.85	0
131.7	97.6	3.78	72.0	6.310	41.00	2	27.55	1
129.5	98.8	6.50	80.0	17.780	42.00	1	10.13	0
130.7	97.0	2.77	54.5	5.540	17.00	0	19.84	1
135.4	97.8	9.09	78.2	8.110	70.00	2	13.37	0
136.5	101.8	3.53	44.6	6.650	27.00	1	28.99	1
143.0	109.1	3.00	88.7	14.030	23.00	2	9.48	1
133.8	97.7	35.93	668.3	30.140	67.00	2	29.55	0
134.3	98.4	3.51	62.4	8.700	27.00	2	34.99	1
128.1	92.2	6.10	81.2	10.540	42.00	2	23.39	0
133.4	101.4	4.21	47.7	14.810	63.00	0	13.83	0
139.0	107.2	4.50	72.7	6.800	37.70	0	8.74	1
132.7	94.6	11.22	51.3	10.100	47.00	2	17.45	0
125.1	100.4	13.77	96.7	10.210	18.70	2	23.76	0
136.1	100.0	5.61	76.0	5.400	138.00	2	25.31	1
131.6	100.3	18.52	153.0	7.270	10.00	2	27.39	0
133.2	103.6	10.52	62.9	11.590	32.00	0	19.62	1
128.8	95.4	4.24	52.4	5.930	37.00	0	31.21	1
126.7	91.3	8.97	85.6	7.610	12.00	2	29.51	1
138.4	104.2	4.94	33.7	6.200	25.00	0	21.49	0
138.8	105.9	7.67	70.3	10.090	37.00	2	6.03	0
131.1	98.7	6.48	130.5	10.630	64.70	0	24.73	0
136.7	109.1	2.92	45.3	10.600	6.00	1	26.6	1
134.7	106.4	9.81	96.0	7.250	42.00	2	12.04	1

127.4	96.3	7.62	52.7	5.820	24.00	0	26.19	0
141.0	107.9	11.12	79.0	11.040	24.50	1	8	0
133.4	98.3	6.98	80.0	6.540	21.00	2	23.56	0
141.0	110.1	6.19	65.8	6.990	30.00	2	12.01	1
144.6	115.2	25.24	95.0	27.840	46.00	1	8.93	0
143.1	112.4	7.66	45.7	26.250	21.00	2	14.36	0
139.8	103.8	6.34	41.9	7.810	24.00	0	10.2	1
132.4	104.2	7.20	92.5	5.400	18.00	2	21.18	0
146.4	114.1	17.71	129.9	10.720	20.00	2	15	0
143.2	114.9	11.23	76.0	5.230	17.00	2	7.61	0
140.4	112.2	4.86	34.8	13.230	41.00	0	14.61	0
143.2	116.4	3.74	51.7	15.220	67.00	0	16.65	0
126.3	95.8	4.73	51.0	9.320	27.00	0	23.78	1
139.3	108.4	3.60	54.3	5.930	20.00	0	12.67	0
139.7	110.2	7.53	73.3	11.980	68.00	0	14.76	0
135.1	104.4	5.25	47.8	8.230	44.30	2	7.97	1
126.5	97.4	5.77	94.9	6.190	48.00	1	30.71	0
134.3	101.1	2.67	75.4	5.660	11.00	2	25.32	0
127.4	95.8	7.29	77.6	21.500	38.00	0	14.01	0
144.9	110.8	3.90	52.0	6.660	38.00	0	15.05	0
130.2	94.2	25.60	376.2	9.300	23.00	2	34.99	0
133.1	95.7	5.08	56.7	20.600	35.00	1	26.49	0
135.7	101.7	3.91	74.3	5.170	46.00	2	10.51	0
127.0	92.6	16.56	82.9	7.560	24.00	2	5.82	1
139.6	111.6	2.30	54.6	7.130	97.00	1	16.91	0
133.3	99.5	4.22	63.5	8.140	0.60	0	21.19	1
137.2	102.9	1.77	46.6	6.150	22.00	1	27.33	0
127.3	94.1	6.57	96.0	4.140	39.00	2	23.21	1
137.3	99.8	4.03	75.4	4.640	36.00	2	13.87	0
144.7	109.5	2.45	53.0	7.430	41.00	0	29.13	0
135.2	92.6	2.21	52.0	5.010	82.00	2	33.4	1
136.8	103.3	6.01	66.3	6.410	17.00	2	16.67	1
135.7	97.7	2.45	46.0	6.310	43.00	2	31.24	1
140.3	109.9	5.35	90.4	7.030	33.00	0	11.94	1
138.0	108.0	4.47	52.0	4.790	35.00	1	13.12	1
136.0	97.2	11.37	81.3	5.230	16.00	0	21.13	0
113.0	81.8	22.91	172.4	8.530	27.00	1	23.56	1
135.9	103.5	11.18	203.0	12.740	26.00	2	16.82	0
142.6	109.1	4.31	73.4	5.570	49.00	0	9.71	0
133.3	94.4	3.85	65.1	3.880	5.00	2	27.25	0
143.7	110.2	9.35	69.0	7.330	15.00	1	9.87	1
137.7	100.3	4.12	56.0	5.990	28.00	0	9.84	0
132.2	98.8	24.78	106.0	15.420	71.90	2	10.25	0
138.6	104.4	3.83	62.0	4.990	25.00	0	17.49	0
134.7	102.4	13.29	125.7	9.310	14.10	2	13.02	0

135.4	99.8	15.70	187.2	7.930	29.00	2	30.65	1
128.2	92.6	7.85	55.6	8.310	50.00	0	26.51	0
141.6	107.7	4.34	46.1	5.660	30.00	0	17.93	0
138.8	105.7	11.89	102.1	7.130	32.00	0	21.81	0
137.7	106.2	13.26	50.6	5.960	99.00	2	16.04	0
142.7	113.8	9.02	62.6	7.890	58.90	0	12.75	0
142.9	115.8	8.35	33.7	9.820	21.00	2	16.16	0
134.8	107.6	10.50	119.0	9.950	30.00	2	12.72	0
127.6	99.1	14.32	79.3	5.950	32.00	2	13.05	1
137.1	107.8	4.61	63.8	9.580	30.00	0	20.31	1
133.9	99.6	2.39	54.1	17.030	36.00	0	17.29	0
139.0	107.2	23.03	164.0	11.390	31.00	2	12.96	0
130.3	99.4	7.10	55.7	13.190	31.00	2	24.01	1
139.9	108.3	2.97	40.4	5.220	17.00	1	20.4	0
142.0	104.1	6.06	45.7	9.860	63.00	1	11.62	0
112.1	80.5	6.45	81.0	8.630	22.00	2	3.36	0
112.1	80.5	6.45	81.0	8.630	22.00	2	7.36	1
127.1	102.5	27.29	232.1	7.660	22.80	0	26.32	0
134.7	101.3	9.61	111.0	4.480	34.00	2	23.66	0
137.7	104.1	17.01	101.5	5.030	38.00	1	9.23	1
138.5	111.8	14.69	210.0	6.320	30.00	2	11.64	0
141.1	110.9	7.02	54.5	9.260	27.00	0	11.98	1
138.5	103.4	9.47	114.4	7.370	48.00	0	17.82	1
144.8	111.1	6.49	45.9	4.520	48.00	1	13.42	0
141.0	109.8	2.51	51.0	6.980	58.00	0	16.56	0
142.3	103.4	5.35	85.3	5.200	19.00	0	0	1
132.6	96.3	4.88	58.2	5.330	34.00	2	16.47	1
138.2	100.9	5.18	68.9	5.560	22.80	2	19.72	1
144.7	113.6	3.30	56.0	10.860	26.00	1	15.47	0
137.7	114.4	11.76	98.0	7.630	65.00	0	11.23	0
139.8	97.2	8.53	96.8	7.090	30.00	2	9.86	0
137.9	103.7	8.26	61.6	8.350	30.00	0	7.76	1
136.9	104.9	7.66	75.2	7.600	36.00	2	11.37	1
129.3	100.3	16.36	140.3	5.350	24.00	2	18.43	0
135.4	106.0	4.63	62.0	6.500	37.00	0	18.29	0
130.5	102.7	7.66	71.0	18.720	54.00	0	21.28	1
138.7	108.8	6.04	66.2	5.960	58.00	1	21.85	0
149.7	115.2	3.99	72.2	5.520	44.00	0	1.37	0
139.5	105.8	3.16	89.1	8.800	20.00	1	18.3	1
134.1	93.0	7.23	75.1	9.320	29.00	2	14.75	0
151.3	124.5	34.54	314.0	7.160	32.00	2	18.11	0
129.7	99.4	10.60	73.5	11.770	37.00	2	20.2	0
143.6	105.2	4.45	64.0	6.880	31.00	0	4.91	0
133.7	103.1	4.61	70.0	10.960	37.00	0	27.8	1
125.5	87.9	4.49	65.2	7.600	21.00	2	11.6	1

130.5	98.2	8.59	71.1	5.700	36.00	0	13.43	0
134.7	102.9	6.47	69.5	4.720	33.00	1	10.01	1
129.3	80.8	17.11	121.4	28.930	39.00	1	29.35	1
132.2	99.5	16.19	196.1	6.450	28.00	1	19.79	0
127.9	95.6	13.74	98.2	9.430	27.00	2	17.13	0
145.5	112.9	9.86	24.5	11.170	28.00	0	11.12	1
124.4	91.9	3.60	54.0	7.090	40.00	2	24.41	1
130.4	95.8	10.32	115.0	5.790	29.00	0	16.41	0
138.1	107.6	30.26	97.9	18.000	97.00	2	8.41	1
134.8	102.9	3.15	69.2	18.350	45.20	0	29.15	1
136.9	101.5	9.67	118.0	6.010	32.00	2	12.88	1
136.7	105.6	4.85	57.8	10.020	32.00	0	14.39	1
139.9	108.1	7.79	52.6	7.310	51.00	2	11.54	0
132.4	100.7	2.58	48.2	7.440	24.00	0	27.41	1
131.5	101.5	20.66	99.0	7.150	19.00	2	6.45	0
132.6	99.1	3.01	63.2	5.220	59.00	0	19.76	0
141.1	104.7	9.14	58.1	7.950	28.10	2	3.25	1
135.7	107.9	5.10	60.1	5.620	11.00	2	4.74	0
133.6	82.2	5.39	68.7	8.500	52.00	2	9.8	1
132.1	97.1	9.89	112.0	6.340	21.00	0	22.41	1
137.1	105.3	8.13	49.0	4.170	36.00	2	13.93	0
142.4	101.3	10.34	147.0	5.960	30.00	2	14.46	0
137.6	107.2	7.46	65.9	5.930	46.00	2	14.99	0
155.2	117.4	10.51	47.8	11.750	20.00	2	20.67	1
141.7	107.6	5.70	85.4	8.910	52.00	1	12.98	1
140.6	109.8	4.66	71.0	5.480	19.00	0	9.04	0
128.8	97.0	25.02	155.8	7.990	20.00	2	26.51	0
134.5	113.2	10.47	151.7	7.150	64.90	2	16.59	0
135.7	107.5	12.12	107.0	5.850	18.20	1	30.35	0
135.9	108.3	11.78	58.6	10.930	47.00	0	17.46	0
139.4	109.2	4.48	73.5	5.050	27.00	0	21.27	1
133.9	97.2	13.46	163.8	10.010	36.00	0	10.58	1
127.6	96.4	8.84	67.0	5.020	20.00	2	22.72	1
139.2	104.7	3.08	56.6	4.800	29.00	0	3.93	0
135.4	98.2	13.56	93.0	7.830	41.00	2	15.6	0
141.6	114.4	16.38	96.0	4.800	32.00	2	5.65	0
132.7	94.2	4.12	82.0	5.020	25.00	0	12.6	0
134.9	97.5	2.48	54.4	6.210	57.00	0	28.87	1
128.4	94.5	18.04	120.4	6.550	23.00	0	26.01	0
140.4	113.3	10.66	136.5	5.920	60.00	0	21.9	0
141.1	107.6	5.66	75.9	9.620	37.00	0	15.04	0
137.6	98.1	1.49	56.3	6.480	18.00	0	4.48	0
143.1	109.4	4.50	55.2	7.430	53.00	0	4.31	0
135.8	106.4	3.61	52.5	7.520	13.00	2	8.01	1
128.6	112.0	13.60	101.4	9.330	25.00	2	18.97	0

133.9	101.5	11.64	72.4	15.890	46.00	1	14.39	0
138.5	105.2	9.19	61.0	12.490	69.00	0	7.9	0
141.8	116.4	14.27	112.5	5.280	21.00	1	13.74	0
134.0	102.3	3.44	55.0	15.230	25.00	0	4.34	0
133.6	104.6	10.59	97.3	19.690	23.00	2	12.27	0
136.9	104.2	7.66	60.0	4.570	61.00	2	7.31	1
134.2	106.9	5.59	62.0	4.910	52.00	0	13.48	0
134.9	105.3	10.19	110.6	10.990	42.00	0	13.12	1
143.0	111.3	8.86	78.3	6.760	37.00	0	12.53	0
135.1	102.1	4.10	52.5	7.320	27.00	0	22.68	1
142.3	111.8	15.80	54.1	12.280	52.00	0	13.14	1
140.7	109.7	6.14	79.1	8.170	37.00	0	8.56	1
127.7	93.2	20.15	187.8	27.150	69.10	2	21.22	0
137.9	108.9	9.18	55.4	9.180	20.60	2	15.2	1
143.1	102.6	2.79	54.0	5.100	28.00	0	8.83	0
143.8	110.1	5.99	53.7	9.900	67.00	1	13.86	0
142.4	107.2	5.24	51.7	8.620	25.00	0	15.4	0
130.3	96.3	6.87	46.8	6.180	36.00	0	8.75	0
145.7	112.3	6.70	46.4	13.770	36.00	0	10.59	0
133.0	99.6	6.55	66.9	9.640	26.00	1	19.81	0
146.8	114.4	8.46	86.6	6.030	13.00	0	11.78	0
139.7	110.7	2.82	47.2	7.420	33.00	0	21.22	0
120.2	85.5	27.40	328.3	9.280	72.00	0	22.48	1
143.6	109.2	3.38	55.0	30.000	30.00	0	9	0
134.3	106.3	12.61	82.5	8.220	33.00	2	12.49	1
139.7	108.3	11.06	20.2	10.230	60.00	2	10.28	0
142.0	110.2	3.25	73.2	6.500	60.00	0	4.87	0
140.3	103.0	5.43	48.0	8.500	16.00	2	8.47	0
134.8	94.8	5.47	93.2	3.670	33.00	1	26.26	0
138.9	106.6	16.44	102.8	7.940	43.00	0	14.3	1
137.5	107.0	4.57	52.2	20.470	43.00	2	9.87	0
135.8	100.8	2.55	40.3	10.600	36.00	2	8.18	1
130.2	103.3	8.48	88.1	6.930	58.00	0	13.08	0
135.6	100.8	12.31	24.1	26.150	17.00	1	7.6	1
135.9	106.8	3.17	84.7	6.320	51.00	2	11.86	1
136.1	101.2	6.76	51.9	7.540	57.00	2	17.28	0
127.3	98.8	5.53	87.8	5.880	42.00	2	14.31	0
136.9	110.3	12.85	72.3	21.210	8.00	0	3.44	1
139.0	108.0	3.65	58.0	3.790	2.00	2	9.51	0
134.8	100.9	7.32	43.8	14.870	42.00	1	12.88	1
137.2	96.0	2.81	39.7	4.750	50.00	0	10.3	0
145.9	113.2	4.32	55.9	6.750	32.00	0	8.84	0
141.6	109.7	9.02	64.3	9.900	41.00	2	8.71	0
140.3	110.4	15.88	90.2	18.010	33.00	2	9.12	0
138.4	105.0	6.25	118.0	7.530	37.00	1	13.07	0

117.6	78.4	17.63	169.2	8.010	24.00	1	18.58	0
135.1	104.6	19.37	185.2	6.440	33.00	0	13.05	0
136.5	109.1	13.90	86.8	18.050	29.00	1	10.67	0
129.0	100.0	3.81	51.2	12.580	66.00	1	8.96	0
138.3	107.0	6.23	42.0	7.150	45.00	0	15.56	0
138.7	111.0	10.19	154.6	6.680	21.00	2	14.37	1
132.6	95.5	1.74	69.4	16.820	12.00	0	8.92	1
136.5	105.0	3.51	66.1	10.120	52.00	0	6.67	0
131.2	101.4	7.39	71.3	18.540	32.00	0	3.98	0
140.2	106.0	6.47	88.0	5.930	14.00	0	9.59	1
127.1	97.6	14.00	117.1	6.230	39.00	2	9.12	1
137.2	107.1	5.34	44.0	5.520	24.00	1	15.1	0
131.6	99.7	39.40	207.0	12.270	21.00	2	21.78	1
141.3	114.2	13.60	183.9	5.650	26.00	2	19.66	1
133.1	95.3	10.51	94.9	7.840	34.00	0	16.66	1
142.8	112.3	9.66	70.3	7.980	39.00	2	12.82	0
142.1	110.7	5.08	84.0	2.980	12.00	0	4.1	0
142.3	109.3	3.46	36.9	8.800	29.00	0	27.94	1
137.2	105.9	11.37	292.3	5.410	52.00	2	26.88	0
147.2	116.5	13.27	92.9	5.250	30.00	1	10.07	1
134.5	106.1	4.90	67.3	7.970	44.00	1	6.13	1
133.8	101.4	17.11	197.0	10.300	14.00	0	11.91	0
139.9	103.6	4.26	82.0	5.030	31.00	1	29.29	0
133.3	101.5	6.36	69.7	8.740	37.00	0	16.22	0
140.3	105.9	4.17	70.0	11.740	24.00	0	15.51	1
140.7	104.5	21.39	114.9	9.920	37.00	0	13.63	0
133.4	103.4	16.25	230.0	6.150	35.00	2	18.36	0
132.9	101.2	17.94	173.0	5.180	20.00	2	14.55	0
139.9	105.2	14.05	80.0	9.850	40.00	2	6.25	0
135.2	103.5	4.53	66.9	7.580	36.00	1	6.62	1
120.6	88.6	10.15	133.8	12.500	56.00	0	20.47	1
142.6	109.1	9.80	65.8	12.190	49.00	0	3.02	0
132.6	101.7	7.95	122.0	12.830	43.80	0	13.68	1
136.0	105.5	5.70	29.7	6.870	55.00	1	11.97	0
144.3	110.0	4.64	82.2	4.720	28.00	0	14.92	1
137.6	94.6	13.84	103.0	6.870	21.00	2	21.29	0
134.1	99.0	4.45	58.5	23.950	53.00	0	23.09	1
138.0	103.2	4.42	81.2	6.020	10.00	0	16.32	0
129.5	93.2	11.99	99.7	8.130	30.00	1	16.84	0
140.8	108.2	3.32	71.5	13.630	48.00	2	8.47	1
139.7	108.6	5.41	66.6	6.490	13.00	2	16.05	1
145.2	109.8	8.21	99.3	9.730	26.00	0	10.54	0
134.7	103.0	4.12	35.6	5.640	49.00	2	15.44	0
135.5	100.8	6.34	61.0	5.660	33.00	0	10.34	0
133.9	104.0	3.12	71.5	9.570	28.00	2	3.18	0

143.7	114.9	10.41	73.7	5.100	44.00	0	16.27	1
142.0	113.1	15.23	113.7	6.350	36.00	1	8.91	0
137.0	97.2	3.32	73.7	7.510	40.00	1	28.91	1
137.2	105.9	10.69	44.8	16.910	28.00	2	12.87	1
137.1	107.6	18.09	189.0	6.600	22.00	2	20.89	1
140.5	105.0	4.14	71.0	5.420	54.00	1	19.14	0
133.7	94.3	2.09	56.9	13.140	45.00	2	16.23	0
143.2	112.0	8.66	89.4	9.220	22.00	0	12.76	0
140.6	112.2	15.39	66.0	5.380	25.00	2	18.36	0
133.3	103.1	16.61	84.0	6.900	24.00	0	14.77	0
145.1	110.2	13.86	72.7	20.020	40.00	0	1.82	1
133.5	97.4	16.10	109.6	8.300	60.00	2	8.88	0
141.8	108.7	10.61	71.8	11.810	22.00	0	10.59	1
131.3	105.3	5.25	66.0	23.540	38.00	0	12.22	1
138.9	108.7	8.27	58.0	11.650	32.00	1	9.96	1
125.4	93.8	4.46	61.1	6.730	48.00	0	20.19	0
141.2	108.8	5.36	67.0	5.280	41.00	0	13.61	1
134.7	95.8	7.77	145.6	5.180	67.00	0	20.92	0
142.2	111.6	13.30	81.3	7.310	2.40	0	15.99	0
134.7	99.5	5.21	70.0	5.020	30.00	2	13.38	1
127.3	92.1	5.74	78.0	14.910	26.00	2	5.02	1
139.5	105.6	4.57	69.3	6.950	38.00	0	7.72	0
140.4	110.3	8.39	79.7	7.090	21.00	2	10.64	1
140.6	108.1	8.86	106.0	5.560	25.00	0	10.59	0
142.4	111.2	2.86	79.7	9.550	48.00	0	14.99	0
134.5	98.4	6.08	50.1	8.820	27.00	0	15.87	0
139.7	110.4	5.77	66.1	7.070	26.00	2	16.56	0
137.8	109.1	5.15	53.6	7.750	61.00	0	7.15	0
143.2	115.1	5.36	66.7	9.520	39.00	2	10.7	0
138.6	108.5	5.70	67.8	6.420	35.00	0	13.98	1
139.7	104.5	17.62	180.0	5.760	41.00	0	22.75	1
138.3	109.6	13.48	48.3	11.990	35.30	0	11.92	1
142.1	112.9	5.07	57.0	5.350	31.00	0	3.85	1
142.3	109.1	15.96	70.9	9.020	25.00	2	8.09	1
142.1	114.3	8.44	64.0	5.190	32.00	0	9.47	0
134.1	107.5	11.29	99.4	6.880	17.20	2	5.27	1
138.8	103.7	4.19	53.0	4.870	36.00	1	5.99	1
137.4	103.2	29.81	197.3	4.780	18.00	1	8.52	1
134.7	105.7	9.74	85.0	4.920	19.00	0	15.23	0
146.1	119.0	6.82	48.7	9.880	92.00	2	13.47	1
138.0	99.3	9.60	60.6	6.500	10.00	2	4.07	0
146.2	112.5	7.73	51.8	11.310	27.00	0	13.04	1
135.7	100.1	5.25	60.0	8.800	11.00	0	3.45	1
135.3	102.1	8.40	96.0	9.020	46.00	1	15.45	1
146.3	113.2	14.61	86.0	14.240	59.00	0	3.36	0

127.8	87.3	3.76	49.0	7.240	40.00	1	17.62	1
135.1	103.2	4.15	46.1	20.960	47.00	2	18.14	1
142.3	112.1	3.75	60.3	4.990	55.00	0	7.83	1
139.9	106.9	10.81	60.4	14.570	29.00	0	7.6	0
136.6	101.2	7.71	78.0	3.670	43.00	2	14.22	1
137.3	104.2	10.69	90.8	10.570	65.00	1	9.69	0
136.6	100.7	10.83	69.3	6.820	36.00	0	16.77	1
143.2	112.6	2.94	71.0	3.840	34.00	0	12.42	0
136.0	109.8	5.21	67.0	8.380	45.00	2	14.55	1
135.4	108.9	9.47	47.0	12.090	35.00	1	17.88	1
136.0	99.2	6.55	84.5	4.180	48.00	1	13.49	0
133.8	97.4	7.87	55.8	6.720	34.00	2	22.63	0
133.2	103.5	14.42	121.9	22.620	37.00	0	15.79	0
141.7	110.5	14.14	63.5	13.130	37.00	0	12.22	0
142.4	111.6	11.75	113.0	7.710	64.00	0	14.09	0
137.0	107.3	11.62	66.0	6.870	30.00	2	15.29	0
139.2	101.8	4.91	49.0	6.460	53.00	1	26.5	0
145.7	115.1	5.03	59.8	9.990	48.00	2	2.7	0
134.2	106.1	6.99	66.2	6.910	50.00	2	11.08	0
144.8	108.4	5.30	50.0	7.970	33.00	2	18.07	1
124.6	94.8	4.10	32.6	7.410	71.00	2	5.78	0
136.3	108.0	4.30	54.8	5.780	41.00	2	13.96	0
139.0	107.3	8.06	67.6	6.830	36.20	0	10.43	0
144.8	111.6	3.64	57.0	4.180	47.00	0	13.49	0
139.5	102.3	10.59	88.4	13.900	24.00	0	2.58	0
141.1	110.9	5.46	49.6	7.640	27.00	2	13	0
123.6	86.1	9.28	81.1	7.660	33.60	1	19.6	0
129.1	100.2	10.57	64.0	4.350	28.00	2	11.54	0
133.2	91.8	4.47	72.7	9.650	52.70	0	6.84	1
133.2	98.4	6.24	89.0	3.420	26.00	2	14.99	0
143.2	109.4	2.72	63.1	5.080	32.00	0	15.83	0
138.4	105.5	5.94	65.3	7.940	39.00	0	5.48	0
132.5	101.6	8.15	64.0	4.270	34.00	1	10.65	0
121.9	87.2	4.20	41.5	5.670	11.00	1	5.99	0
142.1	106.7	4.42	64.0	5.500	26.00	0	9.43	1
131.5	102.1	14.43	113.6	8.610	18.00	1	11.71	0
142.0	105.1	4.48	62.1	8.970	30.00	0	5.77	1
134.8	99.1	4.68	48.4	14.160	33.00	2	4.67	0
141.3	111.0	9.19	61.2	6.710	48.00	0	13.25	0
134.8	103.2	7.12	71.0	8.400	40.00	1	3.53	0
140.5	104.4	4.14	60.0	5.670	24.00	0	13.44	0
135.9	105.6	5.49	51.0	17.720	59.00	0	15.22	1
144.7	113.0	7.73	86.2	9.730	22.00	0	11.38	0
137.1	104.2	12.72	49.4	9.340	24.00	2	11.02	0
144.8	107.5	9.36	58.1	9.000	60.00	0	6.28	0

141.4	102.1	3.36	89.5	4.220	22.00	1	23.1	0
147.7	116.0	3.73	54.2	4.920	27.00	0	20.27	1
135.6	100.1	3.24	59.0	6.050	17.00	2	15.25	1
141.0	110.6	8.13	66.6	12.500	18.00	2	8.93	0
127.8	95.2	8.81	106.1	9.790	44.00	1	23.4	0
132.2	103.7	7.50	66.9	6.840	35.00	2	14.21	0
137.4	104.8	3.30	50.5	6.550	33.00	0	9.02	0
141.7	112.0	6.11	71.0	4.540	47.00	0	10.78	0
142.0	108.9	3.84	45.3	7.470	37.00	0	14.45	0
143.9	117.7	8.76	68.6	8.760	55.00	0	13.17	0
144.2	109.2	6.95	84.0	4.350	32.00	1	15.25	0
142.2	106.6	6.34	57.1	6.630	21.00	2	11.56	0
140.3	112.7	8.70	57.1	9.410	55.00	1	10.44	0
138.5	108.5	8.37	64.0	8.530	48.00	1	4.54	0
135.9	106.1	7.66	60.7	7.740	63.00	0	6.14	0
130.4	96.0	12.97	143.9	45.570	41.00	0	9.51	0
143.1	102.5	2.61	73.0	11.540	48.00	0	9.27	1
142.5	112.4	12.96	35.3	5.850	23.00	0	8.56	1
137.8	108.3	5.78	53.7	14.930	56.00	0	13.21	0
141.2	109.4	3.72	66.3	5.880	48.80	0	6.63	1
135.6	99.2	3.16	53.0	8.930	52.00	2	4.87	1
122.3	95.0	9.84	68.9	33.120	56.00	0	13.43	0
142.7	101.0	7.91	74.2	15.330	65.00	0	15.91	1
140.4	110.3	4.57	71.1	4.850	23.00	0	11.6	1
129.9	95.9	5.56	58.3	5.960	49.00	2	13.24	1
142.5	116.5	15.34	90.3	7.770	58.00	1	8.1	0
146.7	112.9	6.45	61.0	7.970	40.00	0	9.64	1
137.2	98.4	4.20	85.0	5.420	52.00	1	13.2	1
131.9	101.3	11.32	53.3	9.520	44.00	2	9.65	1
124.7	93.3	13.39	181.5	4.640	25.80	0	24.82	0
139.5	109.3	13.98	28.8	11.940	14.00	1	10.74	0
137.9	105.2	7.88	65.7	11.080	30.00	1	11.16	0
140.5	109.2	3.66	56.9	10.150	33.00	0	8.73	1
131.7	94.9	11.69	145.0	4.950	42.00	0	29.36	1
138.1	101.5	6.52	82.1	7.190	40.00	2	17.3	1
139.1	103.2	4.73	60.0	7.640	19.00	1	9.7	1
138.8	105.6	8.36	67.9	8.300	44.00	0	10.97	0
134.8	107.6	3.74	63.0	7.530	42.60	0	14.44	1
138.7	114.8	6.57	103.6	6.190	33.00	2	8.46	1
136.5	104.1	16.18	49.2	41.750	48.00	1	11.27	1
133.2	100.4	8.66	69.0	12.380	37.00	0	17.4	0
132.6	102.3	10.24	113.3	8.010	44.00	1	19.73	0
138.9	112.4	6.02	66.6	7.830	57.00	2	13.99	0
145.9	117.6	9.75	56.2	8.510	23.00	0	10.44	1
130.2	94.8	7.07	66.7	5.160	16.00	2	22.36	1

142.6	112.6	7.27	119.6	5.320	15.00	0	16.11	1
128.0	93.0	9.98	69.0	22.000	31.00	2	12.79	0
137.1	109.3	15.38	63.8	10.820	19.00	0	15.49	1
141.3	105.9	8.60	70.4	8.400	25.00	0	16.96	1
141.0	108.2	5.81	79.0	8.130	62.00	0	12.19	0
140.1	108.6	12.82	141.1	5.710	54.30	0	12.17	1
136.4	107.7	11.35	75.0	11.380	52.00	2	8.62	0
125.9	79.6	15.76	82.5	23.400	38.00	2	15.81	1
139.6	103.4	6.61	78.0	9.010	33.00	0	9.09	1
137.4	97.8	13.99	484.8	3.910	17.00	0	23.58	0
143.3	111.7	15.27	67.2	9.150	53.00	0	6.17	1
134.6	106.5	15.04	109.8	9.270	32.00	2	11.53	0
138.7	97.0	1.04	51.1	10.580	52.00	0	8.9	0
140.4	104.9	6.53	74.3	5.940	17.00	0	1.37	0
139.8	102.5	8.25	100.7	9.860	26.00	0	16.24	1
141.4	109.6	10.82	30.1	7.900	37.00	0	14.75	0
135.9	108.4	4.49	50.0	4.880	35.00	2	9.18	0
136.4	101.4	4.07	63.7	10.680	33.00	2	26.93	1
144.1	110.1	4.94	76.0	5.640	55.00	0	17.84	1
133.4	102.5	10.93	17.2	7.630	35.30	1	9.02	0
141.8	104.1	9.04	91.5	8.070	23.00	1	11.71	0
136.2	104.4	48.00	139.0	4.780	36.00	0	21.15	0
136.8	102.8	7.94	66.3	6.460	54.66	0	15.64	0
139.7	110.6	12.42	49.7	5.090	21.00	1	13.41	1
131.5	95.8	25.14	206.9	6.780	44.00	2	28.74	1
140.8	107.2	11.22	32.0	20.230	18.00	2	9.19	0
145.5	114.1	7.81	80.0	7.320	35.00	0	3.8	1
136.7	114.1	18.82	60.5	14.870	15.00	2	11.53	1
141.8	106.3	3.94	77.0	4.600	34.00	0	15.6	0
139.6	107.6	3.15	63.0	4.680	10.00	2	14.81	1
143.0	107.3	3.24	62.9	6.140	29.00	0	12.31	1
144.4	110.0	1.59	44.6	9.320	25.00	2	11.9	0
138.2	102.9	11.40	48.2	11.180	63.40	1	9.37	0
138.6	108.0	12.92	23.7	16.830	68.00	0	15.09	0
139.4	105.2	8.99	61.0	16.360	35.00	0	6.15	0
131.8	98.3	5.56	82.0	3.980	18.00	2	1.62	0
137.2	101.5	10.42	69.0	5.340	45.00	1	14.87	0
141.3	109.3	9.78	36.8	9.100	29.00	2	14.24	1
146.1	111.3	3.60	68.0	5.080	34.00	0	8.19	0
141.7	105.1	11.13	73.6	7.720	40.00	2	9.45	1
136.9	104.6	12.09	164.3	7.620	27.00	0	14.77	0
140.9	104.1	2.87	67.0	5.100	42.00	0	22.88	0
141.4	101.6	2.13	72.0	4.280	14.00	2	20.87	0
136.9	109.0	6.63	49.1	6.080	43.00	0	13.05	0
142.7	106.5	3.12	62.0	7.060	42.00	2	11.33	0

135.9	103.9	8.16	94.3	7.400	61.30	1	11.66	1
144.1	113.5	3.66	52.0	7.570	39.00	0	11.64	1
137.5	101.3	9.12	53.8	9.090	77.00	0	8.84	0
129.3	101.3	12.50	146.6	11.070	50.00	0	12.06	1
135.7	99.8	2.38	59.4	10.410	52.00	2	30.12	0
142.9	108.7	6.42	59.1	6.370	31.00	0	2.94	0
129.8	99.0	8.30	67.0	6.880	22.00	1	14.21	0
132.6	107.1	14.67	76.2	7.660	41.10	2	9.75	0
129.8	93.0	6.93	60.7	9.510	67.00	1	15.01	0
149.1	116.9	5.94	37.5	15.960	70.00	2	13.39	0
143.3	111.2	6.13	68.5	6.270	38.00	1	15.03	0
141.9	106.4	4.18	52.0	5.160	48.00	0	9.03	1
139.6	108.8	11.32	58.0	8.690	46.00	1	12.45	0
139.2	108.2	3.71	79.0	6.910	25.00	0	12.01	0
133.3	99.6	5.12	157.8	7.160	42.00	0	19.18	1
140.5	108.3	6.80	56.2	8.520	37.00	0	11.54	0
143.5	111.0	12.13	52.4	10.550	75.00	1	10.97	0
138.5	109.9	11.78	75.0	10.270	12.00	2	8.28	0
132.7	98.4	11.08	94.5	11.610	19.00	2	28.29	0
135.3	97.3	8.96	95.3	10.910	36.00	2	6.31	1
140.2	114.3	9.70	75.4	7.530	14.00	0	10.47	0
133.9	100.0	11.65	92.7	11.550	36.00	2	14.68	1
148.9	120.6	16.33	104.0	8.180	19.00	0	8.26	0
133.6	98.2	13.41	127.0	5.230	20.00	1	15.23	0
137.8	102.0	22.32	72.9	7.990	40.00	2	22.39	0
141.6	114.3	15.40	61.3	17.320	22.00	2	10.36	0
139.1	104.3	4.97	74.0	7.590	51.00	2	9.98	1
135.4	94.4	6.11	126.7	5.670	71.00	1	38.26	0
132.2	100.5	16.55	147.5	9.120	52.70	2	13.01	0
139.2	104.0	8.29	86.3	6.450	18.00	1	7.44	0
140.7	106.3	8.91	84.0	8.440	7.10	0	1.76	0
135.6	107.1	12.23	70.5	18.290	41.00	2	9.8	0
139.6	101.5	4.67	67.0	5.930	18.00	2	15.53	0
144.5	112.4	7.98	61.3	12.590	36.00	2	11.94	0
143.9	107.8	5.72	45.0	6.630	50.00	0	13.24	1
140.8	106.6	9.19	154.0	4.680	45.00	2	19.94	1
140.2	107.4	18.84	52.8	7.790	41.00	0	6.05	0
135.3	93.0	3.86	45.0	4.620	36.00	0	8.53	0
136.7	105.0	27.42	174.8	7.480	32.00	0	15.57	1
136.9	101.3	6.15	65.8	10.360	27.00	1	12.42	0
141.2	111.1	7.07	67.8	10.380	33.00	0	6.27	0
141.7	109.3	5.93	47.5	5.270	36.00	0	11.98	0
136.5	109.6	8.70	76.8	9.280	44.00	2	10.66	0
135.7	100.5	10.07	71.7	5.570	48.50	0	15.47	1
137.6	100.4	32.79	170.0	24.070	49.00	1	15.63	1

138.7	102.0	12.01	139.3	9.050	19.00	0	11.64	0
140.2	106.1	2.82	87.2	8.400	48.00	0	22.02	1
136.5	106.1	3.07	46.8	21.680	43.00	0	4.04	1
143.2	109.7	1.47	38.0	5.090	55.00	0	11.79	0
145.0	112.3	5.90	64.0	4.710	25.00	0	21.36	1
132.4	96.7	16.19	136.0	29.080	40.00	0	11.23	1
131.7	98.7	4.36	41.0	8.940	46.00	2	11.34	0
134.8	104.4	10.88	138.1	5.820	60.00	0	22.88	0
141.6	103.9	5.09	69.0	5.520	15.00	0	17.66	0
148.0	111.4	16.59	92.2	10.480	125.00	0	3.39	0
142.8	110.6	6.52	40.0	6.030	47.00	0	10.56	1
140.1	110.4	10.49	64.9	9.190	34.00	1	7.19	1
136.2	105.1	13.27	28.6	14.720	30.00	2	7.76	0
136.0	109.0	5.03	57.5	8.250	28.00	0	10.07	1
133.4	103.4	7.34	95.6	7.910	34.10	0	14.54	0
140.9	108.1	16.17	73.0	8.430	25.00	1	10.36	0
129.0	95.3	5.91	59.3	14.010	29.00	0	10.92	0
139.7	98.7	10.49	176.3	10.830	75.00	2	20.54	0
135.1	105.3	7.19	154.3	19.680	46.00	0	18.13	0
140.4	103.6	27.48	124.9	11.170	56.00	0	8.61	1
132.3	97.7	7.69	71.2	7.750	57.10	2	15.47	0
120.5	88.1	11.78	74.6	8.200	30.00	1	11.78	0
135.5	106.6	10.52	73.7	7.400	31.00	2	11.12	1
129.1	101.9	8.46	47.5	9.480	34.30	0	6.8	1
125.5	94.9	6.58	57.0	5.990	11.00	2	14.63	0
142.5	112.1	13.95	58.1	7.890	55.50	0	4.28	0
136.7	107.2	11.50	26.0	11.530	31.00	0	17.74	0
135.7	105.9	7.16	56.8	8.890	46.00	0	15.35	0
137.5	107.3	5.41	68.0	6.310	30.00	0	8.97	0
140.2	107.6	5.75	74.7	6.280	41.00	0	6.73	1
133.9	108.2	20.48	123.1	6.710	5.00	1	5.34	0
135.1	106.6	7.86	69.0	5.810	33.00	0	4.19	0
127.1	98.6	8.90	62.2	10.560	49.00	2	8.22	1
139.8	107.9	6.78	49.4	5.300	25.00	0	9.58	1
134.3	105.9	7.12	81.0	5.030	27.00	0	7.69	0
127.7	97.3	3.63	46.0	8.990	11.80	0	10.52	0
136.0	107.7	5.81	57.1	7.540	50.00	0	6.14	0
135.3	94.8	3.73	54.0	6.020	40.00	0	11.03	0
131.4	103.3	13.36	75.0	7.460	129.00	2	11.22	1
133.6	102.0	8.09	60.2	8.160	18.00	1	27.33	1
139.3	103.9	3.50	73.0	5.570	24.00	1	3.78	0
139.3	110.1	3.81	47.1	6.830	42.00	0	15.15	1
134.5	103.2	2.99	61.7	8.100	60.80	0	27.21	0
144.2	114.9	9.76	72.0	16.140	11.00	0	3.61	1
137.2	110.5	8.03	61.0	6.040	23.00	2	0.76	0

142.9	114.4	10.59	87.0	9.000	59.40	2	18.59	0
141.4	112.0	6.25	53.0	5.240	33.00	1	17.57	0
138.5	109.8	12.39	149.3	4.760	62.00	2	20.66	0
139.7	111.5	4.44	52.8	6.500	68.00	0	15.82	0
135.5	104.4	8.46	77.0	6.210	44.00	2	9.13	1
138.4	102.9	5.94	29.0	6.410	36.00	0	4.5	0
140.0	109.7	6.41	64.3	5.680	19.00	0	9.33	0
133.5	105.7	5.95	61.1	7.860	24.00	0	10.71	0
130.6	98.4	8.24	77.9	5.300	16.00	2	13.6	0
142.5	110.8	4.26	39.0	4.090	29.00	1	17.09	0
146.5	115.0	5.19	52.0	7.170	51.00	2	14.02	0
135.1	103.3	4.58	83.5	8.210	21.30	0	16.5	0
136.1	105.1	5.93	93.0	5.180	33.00	2	5.2	0
132.6	92.7	7.32	72.5	10.770	15.00	1	17.98	0
120.8	91.7	18.40	160.8	10.410	14.00	0	23.09	0
129.0	101.0	17.02	106.6	7.160	37.80	2	11.88	0
134.7	102.3	7.92	89.9	8.680	22.00	0	9.36	0
134.1	107.3	1.48	28.2	6.940	35.00	0	14.73	1
141.9	105.7	3.31	66.0	4.890	34.00	1	25.17	0
142.3	113.4	7.31	49.3	7.390	87.10	2	8.19	0
141.2	113.5	14.33	75.4	7.460	71.30	2	16.75	0
135.5	102.0	12.40	96.7	8.260	78.00	2	16.67	1
129.2	99.9	5.29	39.8	21.810	22.90	2	11.57	1
138.1	109.1	5.95	61.1	26.030	92.70	1	13.67	0
139.7	109.0	5.81	64.2	8.470	42.00	2	12.39	0
134.8	100.5	4.60	49.0	19.460	48.00	0	11.62	1
139.6	107.7	9.39	128.0	6.350	47.00	0	12.2	0
124.6	95.7	8.82	72.8	15.590	41.00	0	14.31	1
140.2	111.4	13.84	140.7	9.240	34.10	0	12.64	1
134.1	98.3	7.81	78.8	8.610	24.00	2	5.12	0
142.3	112.1	4.24	46.1	5.310	40.00	0	9.53	1
132.2	103.2	10.54	46.6	10.550	49.00	2	5.96	1
135.5	104.4	7.52	78.9	12.030	36.20	2	16.01	1
134.7	102.9	10.46	90.0	6.780	48.00	0	13.84	1
136.8	103.5	13.01	53.7	17.220	53.00	0	9.28	0
131.6	100.6	3.68	63.8	10.270	13.40	2	16.54	1
141.2	113.9	17.24	60.1	18.970	44.20	1	9.02	1
139.5	102.6	7.18	93.7	9.120	67.70	2	22.82	0
136.9	106.5	22.66	125.8	7.170	26.00	0	9	0
136.3	113.1	12.72	109.6	2.730	65.00	0	14.56	1
136.0	106.4	24.89	95.5	14.040	44.60	0	8.87	0
136.4	108.3	8.15	51.7	28.820	54.20	0	14.47	0
137.4	106.2	6.13	50.9	17.670	19.00	0	11.62	0
135.5	107.9	4.24	75.5	7.210	60.00	1	13.17	0
138.0	108.7	7.81	60.0	16.960	43.00	1	17.03	0

133.3	90.4	1.78	82.0	3.570	92.70	2	9.68	0
130.4	94.1	10.24	76.8	12.940	19.00	2	21.3	0
123.6	95.8	11.66	86.2	9.790	42.00	0	11.63	1
143.6	111.8	6.11	81.5	8.950	61.20	1	10.96	0
137.6	106.8	14.33	102.2	8.710	28.00	2	25.7	1
135.3	103.8	10.03	124.5	8.810	49.00	2	9.84	1
135.4	106.5	16.20	89.6	8.230	21.00	0	13.56	0
140.9	107.6	18.38	102.8	7.800	47.30	0	4.87	0
137.3	110.2	3.17	47.5	8.480	82.00	1	16.28	0
138.6	108.0	9.04	62.0	19.380	84.00	2	15.2	0
139.9	113.5	3.49	57.5	16.090	25.90	0	10.96	0
142.2	111.2	3.94	62.9	10.240	74.40	0	15.99	0
142.3	108.3	2.93	54.0	10.260	32.00	0	12.02	0
136.8	106.8	6.29	65.7	6.500	37.00	0	12.19	0
134.4	104.7	7.53	99.5	5.080	71.40	1	18.55	0
141.8	106.9	4.13	75.9	15.520	48.00	0	11.35	0
145.7	115.5	10.59	64.2	9.610	46.74	0	11.75	0
146.7	115.4	19.68	105.0	9.470	49.00	0	17.11	0
138.2	103.9	6.18	90.0	7.680	15.30	2	21.11	0
141.0	107.5	8.35	85.3	4.600	21.00	2	21.1	0
141.1	100.2	5.83	81.0	5.710	33.00	2	8.27	0
134.6	91.8	12.38	79.6	4.880	27.00	0	16.4	0
137.6	105.2	9.91	53.0	16.670	56.00	0	7.19	0
137.5	106.6	7.30	84.0	6.380	48.00	0	7.7	0
139.8	106.3	6.07	58.3	5.800	36.00	0	6.54	0
140.3	104.2	8.92	158.0	6.760	31.50	2	15.85	0
136.2	94.7	5.15	94.5	8.310	33.60	0	13.76	0
130.4	101.8	19.56	86.9	12.330	65.00	0	6.42	0
141.2	107.2	6.30	68.0	5.140	19.00	0	11.14	1
137.8	106.1	4.18	56.0	6.000	10.00	0	7.6	0
137.8	105.5	6.09	72.6	5.640	51.80	1	18.9	0
135.4	109.6	15.57	41.0	12.610	42.00	1	11.74	1
137.6	104.5	5.22	81.4	9.100	57.10	2	24.84	0
139.1	106.4	7.18	70.9	8.940	36.00	0	9.16	1
136.3	111.4	13.36	43.8	8.060	43.00	0	10.65	1
137.5	111.0	5.84	68.0	11.170	48.00	0	6.59	0
136.2	105.5	8.89	68.9	3.910	42.10	0	18.14	0
138.2	101.2	7.08	47.5	6.570	28.00	2	14.05	0
133.5	94.9	3.76	61.8	10.090	48.00	0	0.44	0
142.0	110.4	4.23	84.4	6.720	78.30	0	9.02	0
138.3	104.6	7.16	60.0	4.760	25.00	2	9.28	1
135.5	100.5	8.33	86.0	4.930	34.00	0	23.38	0
142.8	109.8	6.64	66.5	6.970	56.30	0	3.42	0
139.6	107.4	6.47	88.1	15.390	57.00	0	9.91	1
141.7	113.0	4.95	51.0	12.700	53.00	0	8.09	0

145.5	114.4	4.32	69.0	4.810	35.00	0	12.82	1
137.8	100.1	2.94	54.3	6.720	37.80	1	28.37	1
138.3	111.9	12.79	95.9	8.100	13.00	2	9.89	0
134.5	101.7	5.64	124.0	6.920	43.00	2	21.61	1
134.9	104.8	3.58	65.4	12.750	43.00	2	21.71	0
137.6	97.1	4.55	79.0	4.320	25.00	2	18.65	0
131.9	107.2	8.02	98.0	4.910	16.00	0	5.48	0
136.0	105.8	12.71	73.6	8.400	31.00	1	10.66	0
135.4	106.6	8.23	84.0	7.830	52.00	1	8.35	1
144.5	108.4	5.88	46.0	13.060	51.00	0	12.29	0
136.5	99.9	3.88	53.3	17.730	91.30	0	1.95	0
131.4	103.6	8.29	84.0	6.280	43.00	0	10.37	0
138.0	100.8	2.77	46.7	21.290	76.00	0	31.88	0
139.5	106.9	3.52	44.0	4.160	54.00	0	5.23	1
137.4	102.7	7.96	105.0	8.740	41.00	0	7.66	1
131.2	99.3	5.88	88.0	10.050	17.10	0	17.12	0
142.7	107.7	2.78	39.7	12.770	100.00	0	12.51	1
142.3	113.5	5.15	85.8	6.910	26.00	0	10.18	1
131.7	99.0	6.89	52.5	17.200	59.50	2	2.21	1
133.5	105.1	7.39	46.2	11.030	46.00	0	12.48	0
138.6	102.5	16.63	44.3	11.680	52.70	0	10.47	0
133.5	102.1	2.43	47.6	7.020	55.20	2	28.53	0
133.9	105.5	8.35	45.3	8.910	60.00	0	12.12	0
142.1	112.1	7.08	91.0	7.330	26.00	0	10.35	0
136.2	98.6	7.58	95.0	6.290	33.00	2	21.85	1
143.6	114.0	4.79	62.0	5.370	36.00	0	10.97	1
137.7	103.7	3.30	42.8	6.400	20.00	1	2.01	1
140.3	104.0	3.42	77.2	6.130	21.70	2	25.62	0
144.5	109.7	5.84	48.0	5.830	59.00	0	15.34	1
138.3	110.9	4.41	36.8	8.260	75.00	0	9.78	0
137.4	105.5	3.66	61.2	11.340	32.00	0	11.41	0
135.5	105.7	2.96	57.4	5.350	36.00	0	5.27	0
132.5	101.7	14.31	100.8	6.440	44.30	2	3.03	0
134.4	101.3	3.21	49.6	13.200	74.00	0	10.53	1
139.8	110.1	4.20	69.0	13.430	34.00	2	1.47	0
139.1	107.4	4.88	49.2	9.760	24.00	0	9.93	0
138.4	113.6	6.35	86.7	7.540	36.00	2	15.09	1
140.5	109.4	10.36	67.8	8.670	54.00	2	10.67	1
136.7	108.3	11.40	58.0	17.120	36.00	0	2.13	1
134.4	106.4	8.80	102.7	9.650	24.20	2	17.12	0
140.6	109.8	6.24	56.2	9.680	36.00	1	4.55	0
140.6	110.5	5.97	75.0	4.900	30.00	0	16.06	1
133.4	105.1	3.90	56.8	25.090	64.00	0	16.68	0
146.9	116.1	5.71	58.0	13.260	15.70	0	11.52	0
143.1	113.6	10.91	117.0	4.900	44.00	0	3.56	1

126.7	98.0	24.55	67.9	24.370	33.10	0	11.74	0
127.7	87.4	2.69	38.0	8.370	107.00	1	12.64	1
122.5	99.3	12.54	85.0	3.070	15.00	2	11.81	0
137.3	109.4	7.08	35.6	16.460	53.00	1	10.15	1
131.4	100.0	8.24	107.0	4.320	15.00	0	11.43	0
135.6	108.3	11.87	100.0	9.680	32.00	2	15.2	1
142.8	109.4	3.86	65.9	5.900	34.00	0	3.33	1
139.9	108.1	4.36	51.8	5.160	71.00	0	14.94	0
139.7	112.0	12.31	69.0	10.670	34.00	1	2.59	1
137.4	105.7	4.20	51.0	13.320	42.00	0	12.5	0
145.1	121.5	9.04	106.0	4.810	39.00	0	9.64	0
128.1	101.7	12.25	63.0	6.420	148.00	2	8.55	0
139.5	112.6	14.54	86.8	5.590	49.00	0	10.52	1
145.3	112.2	4.26	51.2	5.810	48.00	0	11.81	1
134.7	103.3	6.36	52.3	6.970	21.00	2	30.47	1
137.7	103.6	6.53	52.0	7.920	30.00	0	12.29	0
129.6	101.2	10.06	104.0	10.190	38.10	1	16.1	0
143.0	115.7	4.25	46.0	4.390	35.00	0	13.74	1
139.1	98.3	2.60	44.4	5.530	27.00	0	4.43	1
141.3	109.0	13.10	60.3	6.760	37.00	0	3.52	1
141.4	113.0	6.13	67.4	6.540	32.00	0	16.69	0
135.2	106.7	5.03	52.8	13.660	48.00	0	22.78	1
135.8	112.6	11.16	92.0	4.660	16.00	2	5.12	0
135.0	108.2	14.83	82.5	7.260	62.00	0	7.36	0
144.7	114.0	4.22	73.3	6.640	60.00	0	10.08	0
137.9	109.9	5.12	69.0	4.970	34.40	1	10.26	0
147.7	105.4	4.83	69.2	7.100	68.00	0	12.39	1
123.1	86.6	22.61	102.5	21.260	22.00	0	5.67	1
127.1	88.7	13.67	107.1	13.500	69.00	1	13.82	1
121.3	86.8	5.68	55.5	5.130	24.00	2	21.51	0
136.4	106.3	7.84	65.5	5.410	19.00	2	13.86	1
139.0	110.8	8.86	55.0	11.310	62.00	0	5.63	0
122.5	96.7	7.95	39.3	13.540	16.00	2	9.54	1
133.1	104.2	5.96	62.9	6.780	43.00	0	8.55	1
143.5	109.5	11.84	62.0	14.210	31.00	0	22.84	0
142.7	109.4	8.05	63.5	13.800	24.10	1	8.93	0
141.0	111.2	9.70	81.6	14.040	62.00	1	10.35	0
130.2	96.4	3.84	64.8	11.250	42.00	2	28.19	1
133.7	104.7	6.41	67.7	7.220	50.00	2	8.12	0
128.2	100.6	24.88	80.6	13.050	65.00	0	13.93	0
138.4	109.8	7.15	67.0	9.080	43.00	1	8.84	0
133.9	103.6	12.60	39.9	6.940	22.00	2	6.92	0
138.0	105.7	11.59	89.7	40.590	30.00	1	7.46	1
132.2	101.4	12.49	60.0	23.830	36.00	0	9.11	0
138.9	109.5	7.60	51.2	11.590	52.00	0	6.22	1

138.6	106.1	5.44	66.1	12.370	26.00	2	8.84	0
137.5	105.3	6.16	68.5	5.800	13.00	0	16.11	0
143.3	107.2	3.06	59.0	6.100	34.00	1	12.9	1
139.7	104.1	15.01	63.7	9.470	49.00	0	10.24	0
134.2	106.8	10.10	128.5	7.720	33.00	0	12.36	1
145.0	114.0	7.78	57.0	7.580	20.00	1	14.39	0
134.9	108.6	15.03	88.0	7.420	64.00	1	5.02	1
140.9	108.0	4.38	74.4	6.480	37.00	0	14.02	0
141.1	110.6	5.17	50.0	4.300	30.00	1	9.21	0
143.9	110.3	5.83	53.0	5.170	35.00	0	4.06	1
140.6	108.0	9.68	54.4	5.980	13.00	0	12.61	1
134.6	102.7	13.29	76.2	16.150	34.00	0	10.19	0
130.2	97.3	8.06	74.5	8.820	47.00	0	9.41	1
139.2	103.4	2.20	55.8	5.330	71.00	0	27.91	1
136.5	102.0	7.79	51.9	6.480	22.00	0	8	0
143.9	106.1	4.29	51.7	6.530	39.00	0	0	1
137.4	102.1	3.27	58.0	7.080	31.00	1	15.34	1
134.4	105.5	8.57	52.0	6.930	53.00	0	8.91	1
137.2	108.0	9.87	65.8	9.890	66.00	1	6.05	0
135.1	103.7	3.66	52.0	5.910	32.00	1	10.65	1
136.9	102.1	6.57	61.3	15.770	23.00	0	8.91	1
130.0	93.0	4.12	53.1	8.480	19.00	0	7.96	0
140.5	98.5	8.29	64.0	7.890	24.00	0	6.28	0
130.0	102.8	11.54	101.3	9.920	24.00	0	13.78	0
126.2	96.4	8.60	76.0	9.530	31.00	0	14.76	0
129.9	101.2	5.24	45.0	4.740	20.00	1	15.52	0
142.1	113.8	5.69	69.0	4.260	48.00	0	6.43	0
137.2	101.4	4.08	64.0	4.700	41.00	0	25.38	1
141.5	112.5	3.36	54.0	4.830	50.00	0	15.93	0
142.1	113.6	17.56	20.2	8.310	24.00	0	14.5	0
122.1	91.8	5.04	86.9	9.210	44.00	0	7.69	1
141.9	104.9	5.34	58.0	5.770	17.00	0	12.18	1
136.5	108.3	8.32	47.5	10.640	27.00	2	16.47	1
142.4	114.4	8.58	52.1	5.630	63.00	0	8.22	0
127.4	89.5	15.69	185.9	13.290	63.00	2	32.31	0
132.6	105.6	17.41	98.0	10.270	55.00	2	15.3	1
130.7	80.9	5.92	112.6	3.540	43.00	1	18.47	1
141.0	108.0	12.94	57.3	14.170	20.00	2	12.5	1
130.8	89.4	5.83	63.9	21.320	30.00	0	6.21	1
102.6	71.1	8.27	66.0	4.770	35.00	0	18.62	0
135.9	108.5	11.89	99.3	11.610	54.00	0	8.34	0
137.9	99.3	10.68	51.2	7.800	37.00	0	11.19	0
141.6	112.3	9.20	76.1	7.660	26.00	0	9.45	1
141.8	110.3	7.39	37.6	7.750	44.00	1	9.11	0
134.2	103.9	6.29	47.8	17.290	24.00	0	11.44	1

143.2	109.7	12.24	54.0	8.540	38.00	0	9.11	0
138.2	112.7	10.36	72.1	7.730	18.00	1	9.45	0
132.6	100.2	5.15	68.7	10.350	25.00	0	21.53	1
135.2	106.2	9.60	61.6	7.650	34.00	2	11.69	0
145.3	108.9	5.63	49.8	8.740	40.00	1	15.82	0
137.1	107.3	5.11	64.0	3.660	42.00	0	11.8	1
140.1	110.9	8.20	60.5	7.280	73.00	2	13.63	1
132.0	105.0	3.01	56.5	7.690	44.00	2	13.92	0
142.4	110.9	8.43	58.1	8.670	26.00	0	8.66	0
136.5	111.0	8.52	72.7	7.650	99.00	0	11.97	0
128.0	99.6	4.21	38.7	5.620	10.30	0	16.51	0
143.2	112.1	7.17	80.0	4.740	28.00	0	7.29	0
141.7	113.5	10.47	16.6	7.710	71.00	0	12.04	1
140.8	115.2	4.12	50.2	5.280	33.50	2	10.75	0
134.9	95.1	17.25	103.0	10.220	9.00	2	5.09	0
122.8	76.3	9.99	179.3	9.330	74.00	1	23.24	0
122.6	89.8	9.69	81.1	10.590	56.00	0	25.08	0
130.9	105.1	10.77	78.0	15.230	61.00	0	7.4	1
142.4	108.5	24.60	176.0	3.020	52.00	1	30.21	0
139.5	96.4	9.75	141.4	5.230	54.00	2	24.42	0
133.4	100.2	26.52	425.7	6.540	53.00	2	40.37	1
130.6	96.3	7.07	84.6	6.640	48.00	0	20.5	1
133.8	96.3	9.67	133.0	6.430	13.00	2	35.09	0
120.3	87.3	14.10	79.4	7.100	20.00	0	19.21	0
146.0	100.5	24.84	119.4	16.400	35.10	2	20.03	0
138.8	113.0	13.50	55.1	13.970	35.00	2	7.75	1
131.2	94.0	3.87	61.6	5.270	68.00	0	37.27	0
128.9	95.4	12.43	215.0	3.090	36.00	2	39.17	0
129.8	94.1	8.00	98.3	7.880	40.00	2	9.93	0
126.2	84.5	14.34	273.5	6.660	46.00	2	33.32	0
134.2	90.9	7.78	116.9	5.690	106.00	1	35.87	0
143.3	98.5	25.60	137.5	7.270	45.00	2	31.01	1
123.4	91.2	10.47	59.9	13.790	69.00	2	26.76	1
120.2	94.5	16.50	141.8	7.850	33.00	1	36.16	0
135.7	105.0	16.21	83.7	7.910	26.00	2	5.38	0
140.2	114.7	28.29	272.8	8.850	47.00	2	25.85	1
125.4	90.1	53.35	523.5	9.490	17.00	1	37.13	1
124.3	92.0	9.95	109.0	19.110	20.00	0	19.75	0
128.2	101.6	16.37	202.8	7.130	66.20	0	28.89	1
134.4	95.4	11.05	109.2	9.420	19.00	2	33.04	0
139.6	93.5	7.57	316.3	7.360	157.00	2	35.2	0
130.8	92.0	30.46	542.9	6.680	22.00	2	38.41	1
120.3	88.4	39.15	316.0	8.790	28.00	0	40.61	0
141.8	104.1	7.97	79.9	14.400	28.00	1	21.47	0
112.6	87.2	30.25	275.0	15.140	49.00	1	40.84	1

132.5	100.9	5.63	61.2	6.580	45.00	0	26.23	0
127.2	93.8	16.36	126.9	4.710	26.00	2	31.66	0
131.9	97.3	10.20	60.0	5.890	36.00	1	23.96	0
123.4	93.1	18.81	87.4	5.440	35.00	2	23.28	0
128.2	95.2	7.20	44.1	11.880	35.00	0	19.43	0
134.6	102.1	3.75	56.0	6.660	41.00	1	26.19	0
135.8	108.0	5.83	44.0	6.000	28.00	2	34.26	0
136.3	96.1	21.43	172.2	6.080	34.00	2	18.73	0
135.0	97.7	11.23	59.8	8.410	49.00	1	20	0
124.9	86.8	26.16	196.5	7.610	42.00	2	18.69	0
120.0	84.5	8.09	58.0	3.400	20.00	0	30.21	1
103.9	68.9	8.25	60.1	8.740	24.00	2	17.59	0
138.2	107.6	20.73	99.0	7.350	114.00	0	8.09	0
137.5	104.5	4.87	87.0	6.240	38.00	0	26.3	1
137.3	104.9	0.56	43.8	11.420	30.50	0	32.77	0
131.2	101.8	20.11	116.0	18.300	99.00	2	26.45	0
135.3	105.4	3.61	38.9	7.920	36.00	0	15.63	0
132.7	111.5	8.01	80.7	6.330	44.00	2	4.39	1
120.8	94.8	9.32	82.0	5.940	20.00	2	16.73	0
129.4	97.6	3.67	40.0	6.550	29.00	0	14.53	0
119.8	93.0	18.04	175.0	4.600	122.00	2	31.21	0
143.7	108.2	6.26	96.0	5.040	30.00	0	24.92	0
135.6	98.6	7.70	68.2	13.760	39.00	2	13.67	1
125.5	93.0	17.70	136.4	9.670	2.80	2	29.56	0
137.7	111.0	21.89	128.0	6.530	108.80	2	11.96	1
129.2	98.7	20.21	150.6	5.730	26.10	1	28.39	0
119.8	92.1	8.19	77.3	9.840	15.00	0	28.67	1
136.4	96.8	11.51	74.3	8.670	15.00	2	12.1	1
121.0	88.2	8.48	90.3	4.310	26.00	2	12.38	1
124.8	86.7	3.60	56.8	6.460	44.00	0	19.92	0
126.5	109.4	30.29	200.6	10.230	41.80	1	29.33	0
132.6	99.2	4.42	53.0	11.550	32.00	2	27.37	0
142.7	102.7	19.08	207.8	7.040	36.00	2	31.46	0
137.8	103.5	2.50	52.0	6.490	34.00	2	26.11	1
126.0	92.8	16.87	115.7	29.310	58.00	1	19.36	1
140.6	105.1	5.30	115.0	4.520	34.70	2	24.5	0
137.1	94.2	20.97	168.0	7.810	15.00	2	27.35	0
128.3	91.0	4.93	41.8	8.440	19.00	2	19.65	0
138.9	103.2	3.47	55.0	4.070	16.00	0	26.07	1
151.1	123.6	15.84	84.4	8.450	20.60	2	12.02	0
142.0	109.4	3.09	48.0	5.470	20.00	1	8.28	0
142.1	105.9	5.41	59.0	6.000	23.00	0	6.76	0
